# Supplementary material for: Mental health and help seeking among trauma-exposed emergency service staff: a qualitative evidence synthesis
Source: BMJ Open. 2022 Feb 2;12(2):e047814. doi: 10.1136/bmjopen-2020-047814 (PMC8811562; doi:10.1136/bmjopen-2020-047814)
Supplement: Supplementary data [file bmjopen-2020-047814supp001.pdf]

**Online supplementary file 1: ENTREQ checklist (Enhancing transparency in reporting the synthesis of qualitative research**

| No | Item | Guide and description |
|---|---|---|
| 1 | Aims | -to identify factors and contexts that may contribute to mental health and recovery from psychological difficulties for emergency service workers (ESWs) exposed to occupational trauma<br>-to identify barriers and facilitators to help-seeking behaviour among trauma-exposed ESWs. |
| 2 | Synthesis Methodology | Thematic synthesis |
| 3 | Approach to searching | Pre-planned comprehensive search strategies, combining database searching with manual search methods. |
| 4 | Inclusion criteria | Articles were eligible for inclusion provided they met the following criteria:<br>(1) Study participants were frontline ESWs (studies with mixed populations of eligible participants were included); (2) The study focus was work-related psychological distress*; (3) Data collection included primary qualitative interviews, focus groups or observational methods (this included mixed methods studies with qualitative components); (4) Analysis focussed around participant attitudes towards: a) behaviour aimed at improving or protecting mental health after experiencing a traumatic event OR b) factors which ESWs find helpful or unhelpful for their mental health while experiencing work-related psychological distress; (5) Published in English and peer-reviewed.<br>No limits were applied to publication date or study location.<br>Exclusion criteria:<br>(1) Due to the unique nature of the traumatic events witnesses in this cohort,[40] studies investigating a military cohort were excluded; (2) Volunteer ESWs were not included. |
| 5 | Data sources | OVID MEDLINE, EMBASE, PsycINFO, SCOPUS. To identify articles missed in the electronic database search, the following |

| | | |
|---|---|---|
| | | methods were also employed:<br>-Using 'related article' feature (when available).<br>-Searching the titles of included studies in google scholar for citation tracking purposes.<br>-Manual searching of the references of relevant studies (reference chaining).<br>Grey literature was searched during background research for context but not to locate eligible studies. |
| 6 | Electronic search strategy | The two combinations of search criteria (see Appendix A) were entered into each database. Figure 1 provides an overview of the database search. |
| 7 | Study screening methods | One reviewer (NA) screened the 13381 article titles and/or abstracts identified by the search. The 42 full text articles identified in this process were then independently assessed for their eligibility criteria by two reviewers (NA and RR) (Figure 1). |
| 8 | Study characteristics | Study characteristics are presented in Table 1. |
| 9 | Study selection results | PRISMA guidance was used to construct a flow diagram displaying the database searching process (Figure 1). Of the 13381 records identified once duplicates were removed, the full text of 42 articles were screened, and 24 studies were included in this qualitative synthesis. |
| 10 | Rationale for appraisal | All potentially relevant studies were quality appraised by one reviewer (NA), using the Critical Appraisal Skills Program (CASP) guidelines. Studies meeting less than 5 criteria were then subject to further scrutiny, in the form of five quality appraisal prompts developed by Dixon-Woods et al. (see 'quality appraisal' section). This second stage of appraisal was carried out independently by by RR, MB or JW, and decisions relating to the inclusion of these studies in the review were made following thorough communication between reviewers and referring to pre-determined quality prompts. |
| 11 | Appraisal terms | Critical Appraisal Skills Program (CASP) guidelines and quality appraisal prompts (see 'quality appraisal' section) were used to quality appraise all included studies. |

| | | |
|---|---|---|
| 12 | Appraisal process | Quality assessment was carried out primarily by one reviewer (NA), with independent verification from RR, MB or JW for ambiguous studies. |
| 13 | Appraisal results | CASP scores of included studies are provided in Table 1. Full study quality assessments are available for review if required. |
| 14 | Data extraction | A data extraction template (Appendix B) was created for this review by one reviewer (NA). Sections for first, second and third order constructs are included in the extraction template, which was filled manually by one reviewer (NA). |
| 15 | Software | RefWorks reference management software. |
| 16 | Number of reviewers | Two reviewers were involved in the coding and analysis (NA, RR). |
| 17 | Coding | Two reviewers carried out line-by-line coding, grouping of codes and generation of descriptive themes (NA and RR). |
| 18 | Study comparison | During primary readings of the studies, overarching concepts relating to the research aims were noted. The generation of new codes altered pre-existing codes. |
| 19 | Derivation of themes | Initial coding was carried out in correspondence between two reviewers (NA and RR), and was grounded in the extracted data. Thematic synthesis of initial codes was an inductive approach, and involved roundtable discussion between all four authors. |
| 20 | Quotations | Appendix C provides example literature quotations from included studies which were used to construct themes. |
| 21 | Synthesis output | This qualitative review generated 14 descriptive themes. These are grouped into either 'Factors contributing to the protection of mental health' or 'factors influencing mental health help-seeking behaviour'. Table 2 presents a summary of themes. These descriptive themes were applied to the context of UK emergency service organisations to produce implications for practice and research. |

Tong A. Enhancing transparency in reporting the synthesis of qualitative research: ENTREQ. BMC Medical Research Methodology ;12(1):181-182.
