# Supplementary material for: Mental health and help seeking among trauma-exposed emergency service staff: a qualitative evidence synthesis
Source: BMJ Open. 2022 Feb 2;12(2):e047814. doi: 10.1136/bmjopen-2020-047814 (PMC8811562; doi:10.1136/bmjopen-2020-047814)
Supplement: Supplementary data [file bmjopen-2020-047814supp003.pdf]

Appendix A- Abbreviations and definitions

**Abbreviation**	**Meaning**
NHS	National Health Service
PTSD	Post-Traumatic Stress Disorder
ESW	Emergency Service Worker
ESO	Emergency Service Organisation
EMS	Emergency Medical Service
CISD	Critical Incident Stress Debriefing
UK	United Kingdom
TRiM	Trauma Risk Management
GMB Union	General, Municipal, Boilermakers and Allied Trade Union
RTA	Reciprocal Translocation Analysis
CASP	Critical Appraisal Skills Programme
EMT	Emergency Medical Technician

**Term**	**Definition**
Psychological distress	'the unique discomforting, emotional state experienced by an individual in response to a specific stressor or demand that results in harm, either temporary or permanent, to the person'[41]
Critical incident	'any event with sufficient impact to produce significant emotional reactions in people now or later'.[9]
Supervisor	EMS frontline personnel responsible for overseeing a 'crew' of EMTs/paramedics. *Brown University- Roles And Responsibilities Of Supervisory Staff. [internet] 2021[cited 2021 June 1]. Available from: https://www.brown.edu/campus-life/health/ems/roles-and-responsibilities-supervisory-staff#:~:text=EMS%20Supervisors&text=The%20EMS%20Supervisor%20is%20the,and%20appropriate%20emergency%20vehicle%20operation.*
Mental health champion	A member of an organisation raising mental health awareness within the workplace[99]

**Type of stigma**	**Definition**
Anticipated stigma (Perceived stigma)	*'the extent to which people believe they personally will be viewed or treated in a stigmatizing way if their mental health problem or related help-seeking becomes known'* [77]
Treatment stigma	'the stigma associated with seeking or receiving treatment for mental ill health'[76]
Internalized stigma	'holding stigmatizing views about oneself'[76]
Public stigma	'invalidating and unjustified beliefs (i.e., prejudices and endorsed stereotypes) about others'[83]

Auth NM, *et al. BMJ Open* 2022; 12:e047814. doi: 10.1136/bmjopen-2020-047814

Appendix B: Literature search terms

(((mental AND (health OR well?being)) OR (trauma* OR *stress OR recover* OR PTSD OR post?traumatic?stress OR emotion* OR (critical AND incident) OR (traumatic AND incident) OR (traumatic AND event) OR experience* OR support* OR *support) AND (emergency service* OR emergency medical service* OR EMS OR first responder* OR ambulanc* OR paramedic* OR firefighter* OR fire service* fire department* OR police*) AND (qual* OR mixed?method* OR interview* OR focus?group*) NOT (quality OR emergency?department)

((emergency service* OR emergency medical service* OR EMS OR first responder* OR ambulanc* OR paramedic* OR firefighter* OR fire service* fire department* OR police*) AND (qual* OR mixed?method* OR interview* OR focus?group*) NOT (quality OR emergency?department)) AND ((help-seeking* OR stigma* OR mental* OR barriers OR (MeSH terms: help-seeking behaviour, social stigma, mental health, psychiatry, social support, mental disorders))

Appendix C: Data extraction template

Citation
Reviewer
Country
Aims
Ethics – how ethical issues were addressed
Study setting- e.g. type of organisation

Relevant context to study setting

Socio-demographics of the country / region

Recruitment context (e.g. where people were recruited from)

Sampling- what sampling methods, what were inclusion and exclusion criteria,

Data quality rating

Participants- ‘population described’

Participants- ‘characteristics’- age, sex etc

Theoretical background

Proportion of sample exposed to critical incidents/ definition of critical incident/ anything relevant to the study matching my selection criteria

Definition of critical incidents/ something related

Data collection methods- data collection methods, role of researcher within setting...

Data analysis approach- how many researchers, how did they code, how were findings corroborated,

Themes identified in study (1st order interpretations)- Help-seeking

Themes identified in study (1st order interpretations) – Mental Health recovery

Data extracts related to key themes- Help-seeking

Data extracts related to key themes- Mental health recovery

Author explanation/interpretation of key themes (2nd order interpretations)- Help-seeking

Author explanation/interpretation of key themes (2nd order interpretations)- Mental health recovery

Recommendations made by authors (both outcomes; but specify)

Third order interpretations:

Other potentially relevant information

Appendix D: Example literature quotations used to construct themes

**Factors contributing to mental health recovery post traumatic incident exposure**	**Organisational**	**Time out/Downtime**	*They prefer support to be offered immediately after the call and find that downtime after a stressful call allows them to decompress and prepare for the rest of their shift: '…we knew we weren't going to get a call right, so we knew we had the two hours, so we watched a funny show and had a nap…But…like for me personally… that's exactly…what I needed.' Participant 4 from focus group 4.* Douglas et al. 2013<br><br>*. . . our supervisor took us out of service for a couple of hours and let us go have lunch, sat down and had lunch and just kind of relax and talk amongst ourselves, not even about the call, just about whatever, just to kind of relax. Before we went back on shift. Certainly we could have booked off the rest of the day, you know, on stress leave or whatever, but we all, found that just having, just being able to have a couple of hours to, kind of, you know, relax a little bit, that helps us a lot.' (Focus group #520–522)*<br>Halpern et al. 2008<br><br>*I like didn't want to be like I need to take a couple of hours off because I did not want to look bad in front of the supervisor.-Participant 2 from focus group 2.*<br>Douglas et al. 2013<br><br>*Unfortunately, paramedics often do not have adequate time to talk. Several participants expressed their frustration over 'calls waiting'. These calls are waiting to be dispatched as no crew is available. When BCEHS paramedics offload their patient(s) at the hospital, dispatch can immediately send them to a waiting call. This can limit paramedics' ability to discuss CIs. James desired:*<br><br>*…to be given the time uninterrupted, unbothered… Give us our time to decompress.*<br>Drewitz-Chesney et al. 2019
		**Supervisor**	*Depending on who it is . . . one supervisor, we took, we did a [critical call], and we took the last hour of the shift off. And he was making us fill out all these forms and, you know, telling us that if we wanted to take the next shift off that we had to go see our doctor and get a note for this and that. And you know, just made it more stress-(Focus group #520–522)*<br>Halpern et al. 2008<br>*'And so our supervisor was really, we have a great supervisor and he's, you know, asked us all specifically, "are you guys okay", you know. And the other crew went off on stress for the rest of the shift. We stayed, because we said, y ou know, we're okay. We just kind of dealt with the aftermath of everything. It was still a pretty stressful call but at least we had that option. And he had no problems, like, he said, go home. Whatever you guys need. So and that's a big thing.- (Focus group #520– 522)*<br>Halpern et al. 2008

			Supervisors, however, were perceived differently. Some paramedics reported feeling unsupported when their supervisors questioned why they were reacting to an event that they did not perceive as traumatic:<br>*I wasn't really involved in a traumatic event because there was no loss of life, or you know what I mean, um, it, there wasn't really much sympathy the, um, I could, not that I really, um, oh, this wasn't, um, a huge accident, you weren't injured, you know, really why are you complaining?*<br>Regehr et al. 2007<br><br>*he's [supervisor] quite an old fashioned sort of police officer, not the bloke you would sort of want to go in and have a chat with about a sudden death you'd just been to … If I went in and said 'Governor, can I have a chat about the sudden death?', he'd look at me as if I'd just asked to kill one of his children!*<br>Evans et al. 2013
		**Peer support network**	*Nurse 1 "Therefore, talking to a person who has the same skills, abilities and knowledge you have would probably be more meaningful".*<br>Carvello et al. 2019<br><br>*The majority of nurses is in favour of peer-supporters. The motivation is based on the fact that they recognise the peer-support as someone that can understand what nurses really mean when relating a traumatic event, being one of their colleagues.* Carvello et al. 2019
	**Infor mal suppo rt**	**Colleague s and family**	*The more you talk about something, the more it becomes something you've told and your telling becomes part of the memory, as opposed to it being a really shiny, vivid thing inside your head*those images.*<br>Evans et al. 2013<br><br>*I don't think talking about it to people at work is the release, the escape I need … it's speaking to people who I care about and who care for me and just having that comfort zone, that's what's important to me. [P16]*<br>Evans et al. 2013<br><br>*… I never tell my wife that, I would never tell her that because I just think that would have really put the frighteners on her. [P15]*<br>Evans et al. 2013
		**Regular partner**	*For me it's just always been that partner, because they're right there with you and they'll know what's going on, and you really want somebody that can understand what's happening.*<br>*Jessica et al. 2009*<br><br>*…for those of us that have regular partners, regular stations, rely on your partner… They're gonna know if something's up with you.*<br>Drewitz-Chesney et al. 2019<br><br>*Paramedics working with different partners may be unable to recognise changes as readily as they would in a regular partner. When paramedics are without a regular partner or are uncomfortable speaking with their*

			current partner, they often don't discuss calls. Instead, some participants said they speak with long-time paramedic friends or coworkers with whom they have similarities, ie. age or training level.<br>Drewitz-Chesney et al. 2019<br><br>*…if I'm working with my regular partner, those communication lines are very, very open and we can talk about the tough calls, how we're feeling, if there's anything we would have done different [sic] (Sean).*<br>Drewitz-Chesney et al. 2019<br><br>*I really envy the paramedics that have regular partners that they know, and they trust, and they can talk to (Krista).*<br>Drewitz-Chesney et al. 2019<br><br>*…I would have a regular partner and that partner would be someone who is just like family to me. We would just talk about everything, without even hesitating…'*<br>Drewitz-Chesney et al. 2019
		**Reassurance and validation**	*I remember going to the hospital and I remember I was disappointed because the parents weren't there at that time...And I don't know what it was. It was just something that made me feel like I just needed to talk to them. But I never ended up talking to them. So anyway that call definitely stands out as being...(Stuck with you.) Yeah.*<br>Halpern et al. 2009<br><br>*And it's weird, I looked in the newspaper. I saw his funeral announcement and I went to the funeral. (Oh okay.) I didn't talk to any of the family. I just went for my own, I don't know why …I, for some reason, I guess I felt I needed to follow up and so I did. And I sent the family a condolence card and then a friend of mine …met a friend of the family's friend and he had said, oh, his [relative] wants to talk to you …So she called me and she wanted to know, like, you know, what happened with her [relative] and who was the last one to talk to him …It's kind of funny because there are so many calls you do and you never think about them again. But this one, I thought about him a lot. I guess because he was so young and again, I really didn't think he was going to die. So I thought, okay, well, you know, he's at the trauma centre now and I had no idea that he was that badly injured. And then he died. So I think that freaked me out. I wasn't prepared for that. And I thought, oh my God, he's so young and his family wasn't there. I felt badly his family wasn't there and then. So that one stuck with me for a while.*<br><br>*Interviewer: "And talking to his [relative], did that help or …?"*<br><br>*It more, I felt it was helping them so it made me feel better, because she really, really was upset. And I felt she wanted, I think she felt a lot better after talking to me and hearing something about what happened. And so it made me feel better to give them some kind of closure.Pg 181-182*<br>Halpern et al. 2009

			. . . the day after that call, my supervisor kind of sensed that I wanted to talk to him and I don't know why, you know, he came to the hospital. But it was the next day and he was kind of like, so pull up a chair, sit down. Let's just talk. And it was like I wanted somebody just to say to me, okay, this is your time and you can talk right now and I'm not going to judge you. I'm not going to talk about anything. I'm going to let you just have your 10 minutes. And that made a huge difference. I have the utmost respect for my particular supervisor . . . Whether it was talking specifi cally about the call or just having a little bit of a "hey you're okay at your job", type comment. (Participant #122)<br>Halpern et al. 2008
**Factors influencing help-seeking behaviour**	**Nature of intervention delivery**	**Mandatory vs non-mandatory**	*We had counselling every six months ... and everybody used to go 'Oh I've got to see the counsellor this week', but I tell you what ... we all quite enjoyed it ... I was so much calmer after speaking to her but it's something I'd never have done had I not been made to do it. [P12]*<br>Evans et al. 2013<br><br>*My emotions are none of your business and if I wanted to share my emotions with you, I'm going to share [them] with someone I trust… Participant 1 from focus group 1.*<br>Douglas et al. 2013
		**Shared experiences with intervention provider**	*FRs in our study preferred a MH professional with experience as an FR or military veteran. Many also approved of a provider that "knew the job," either working with multiple FRs in the past, or even as a family member.*<br>Jones et al. 2020<br><br>*I've been to [therapy] a couple of times. . . . The guy that I got was excellent, but I only think it was excellent because he was prior military (P8, Firefighter ×22 years).*<br>Jones et al. 2020 (study 4)<br>*…someone I think understands what's going on, and has been through what I've done.- Participant 3 from focus group 4.*<br>Douglas et al. 2013

	**Stigma as a help-seeking barrier Specific barriers**	**'Macho culture'**	*I think there's a real element of machismo and masculinity in the police force and it's a bit, sort of a faux pas to admit that things have really affected you ... If I'd have come out and said 'ah you know, that really affected me badly, let's go and sit down and have a cup of tea and talk about it' I think you're straying into pink and fluffy territory there ... saying 'that made me feel sad' is a bit too far. [P3]*<br>Evans et al. 2013<br><br>*Everyone wants to be tough and strong. Maybe that was my downfall or problem at the time and I didn't want to admit that I needed any kind of help. I guess I didn't want to be perceived as weak. (Participant #110)*<br>Halpern et al. 2008<br><br>*… that's so ridiculous. You need to harden up if you want to do this job, and old people die.*<br>Drewitz-Chesney et al. 2019<br><br>*… in metro, there's a lot more...bravado and joking around about things…If you can get one on one with someone, they're usually a lot more receptive and a lot more empathetic- (Dennis).*<br>Drewitz-Chesney et al. 2019<br><br>*Unfortunately there's a massive stigma […] one of my sergeants the other week, there was mental health training coming up and his reaction to being put forward to go on the training was, 'Well what do I want to go and learn how to deal with a load of nutcases for?*<br>Bullock et al. 2018<br><br>*We are many women at the station so its easier for us to talk about what happened…You have to talk about what happened otherwise you can't go on. It's easier than on a station with only men, it's not necessary with any "macho style" so I think it's easier for the men (at our station) to talk about things you must talk about. (A female nurse describes her feelings.)*<br>Jonsson et al. 2003
		**Stigma and shame**	As an institution, they were very very good, in getting me better, at making me understand, but back at work that was a different ball game. You've got your colleagues who are still at work, still running around like idiots and they're like, oh fucking hell, you've had three months off, you know, I should have gone off with stress. (Participant 1).<br>Edwards et al. 2020<br><br>*There have been officers that are doing the shift that have shown that they can't deal with situations like that, and been very open about it*and they haven't got the respect from the shift, because the colleagues go 'well, you're on your own if you're working with her, because she'd back away' or whatever. So you don't want to be considered as one of those. [P7]*<br>Evans et al. 2013
		**Career concerns**	*I think that the stigma is you have to be very careful who you tell that it bothered you or you might get judged as weak or you might get fired" (P2, EMT/paramedic ×20 years).*<br>Jones et al. 2020<br><br>If you've a form of mental health illness you will not get on; you will not be promoted, …people will not want you on their section. (Participant 4).<br>Edwards et al. 2020

			I feel scared to declare anything or do anything about anything because will it bite me later on in life? Will it prevent me from doing something in the police later on? Could it be used against me? … will it be used in a negative way later on?<br>Bullock et al. 2018
		**Confidentiality concerns**	*I know our department's very, very poor at keeping secrets. So if I put a crew out of service, I have to tell the communications center. Communications says, we're sitting this far apart, Hey X, I just put the 22 car out of service in stress. Every dispatcher in there hears it. Every call receiver in there hears it. So they hear it. I don't know if they say anything. I don't know if they go home and tell all their friends and family. But I don't like that system. There's no quiet way of doing it. (Participant #128)*<br>Halpern et al. 2008<br><br>*I think that the stigma is you have to be very careful who you tell that it bothered you or you might get judged as weak or you might get fired" (P2, EMT/paramedic ×20 years).*<br>Jones et al. 2020<br><br>*.. .to whom must I speak in the police if I can't trust anyone. So now I'm seeking professional help outside the police .*<br>Boshoff et al. 2015
	**Mental health literacy**	**Emotional awareness**	*Some appreciated that difficulties in recognizing and admitting to distress pose significant barriers to accessing support. Recognizing the emotional impact of critical incidents may help to address these barriers.*<br>Halpern et al. 2009<br><br>*I didn't recognise it as what it was; I just thought I was grumpy…you don't see them creeping up, and in the end, the thing that tips you over the edge, the thing that makes your bottle overflow if you like can be something quite small because you've got used to dealing with stuff. (Participant 5).*<br>Edwards et al. 2020
		**Education and stigma**	*I think for, for everybody, is, here's my thought towards your process, is giving them the tools. People are going to be very tough and say, yeah, yeah, fine. But you know if you could somehow identify the emotions that go along with these calls that might be starting to put you on tilt...Then you can teach people to be aware of them and say, hey, you know what, it's okay to say, I need to talk to someone.*<br>Halpern et al. 2009<br><br>*In particular, it would appear from this study that teaching ambulance personnel about the emotional aspects surrounding different types of critical incident may diminish their confusion about which incidents they can expect to impact them.*<br>Halpern et al. 2009<br><br>*I guess that's the main two [barriers], pride and then denial. . . . But, it's just education. Just letting [FRs] know, look, these things are normal. It's going to happen to somebody" (P11, Firefighter ×8.5 years).*<br>Jones et al. 2020

Auth NM, *et al. BMJ Open* 2022; 12:e047814. doi: 10.1136/bmjopen-2020-047814

			Knowing that other people are there dealing with that same stuff. You can bounce ideas off each other, see what's worked in their situations and what hasn't . . . you realize you're not crazy (P12, EMT/paramedic ×14 years).<br>Jones et al. 2020<br><br>*If you could actually get people in in front of officers saying, 'I was one of those people that didn't believe stress could ever get to that level and it was ridiculous and you just needed to work harder', maybe officers would accept that from another officer more than just somebody standing in front of you training because it's getting officers to accept that actually it's okay, you're only human and your body and your mind can only take so much, and maybe they'd accept it more then.*<br>Bullock et al. 2018
