# Supplementary material for: Mental health and help seeking among trauma-exposed emergency service staff: a qualitative evidence synthesis
Source: BMJ Open. 2022 Feb 2;12(2):e047814. doi: 10.1136/bmjopen-2020-047814 (PMC8811562; doi:10.1136/bmjopen-2020-047814)
Supplement: Supplementary data [file bmjopen-2020-047814supp002.pdf]

Manuscript online supplementary files

Online Supplementary File 1: ENTREQ checklist (submitted separately)

Online Supplementary File 2: CASP appraisal table

Online Supplementary File 2: CASP appraisal table

Study title (Authors, year)	Aims & Methods	Research design	Sampling	Data collection	Reflexivity	Ethical issues	Data analysis	Discussion of findings	Value
What makes an incident critical for ambulance workers? Emotional outcomes and implications for intervention. Halpern et al. 2009[42]	Research aim clearly stated, introduction uses relevant literature to successfully explain demand for the research. Qualitative methods are appropriate for investigating complicated emotions, which could not be explored in equal depth via quantitative methods.	Interviews and focus groups seem appropriate methods to address the research aims, but the authors don't specify the reasons for their choice to use both methods.	4 supervisors, 54 front-line ambulance workers. Researchers state that participants were recruited from a specific cohort at a mandatory training conference, but no detail is given about what information was provided to the participants to entice them to enter the study. The authors considered representation of all job levels and both genders	The setting of the data collection away from the workplace was stated but no justification was given for the choice of study setting or methods used. Detail was given about the length of interviews and focus groups, and the main topics of questions asked during interviews and focus groups were stated. The researchers stated they had reached saturation by the end of the study. Data obtained was in the form of	Researchers do not acknowledge how their characteristics may have impacted on the results. The researchers acknowledge that self-selection or reporting bias may have contributed to the discrepancy in findings between men and women.	Ethical approval was obtained from a research ethics board, and participants signed a consent form. No further information was given regarding any ethical safeguards in place. Lack of description of how confidentiality was maintained.	The authors described how ethnographic content analysis was used to generate thematic codes, with a constant comparative method throughout. Three authors were involved in the coding process, although only two of these authors listened to the audiotapes. Initial and final codes are described, and contradictory data are taken into account. Sufficient data are	Findings are explicitly stated and are relevant to the researchers' aims. The only measure of credibility described was the use of three analysts during the coding process. Findings are discussed with reference to wider literature, and implications for interventions for critical incident stress are made.	The authors identify promising post-incident interventions which they believe should be further researched. Wider applicability of the study findings appear to not extend beyond the ambulance cohort. The authors provide implications for interventions for critical incident stress.

			during recruitment.	audio recordings, written transcripts and written notes of non-verbal communication information as observed by the researchers.			presented to support the findings. The authors describe how textual data was not analysed for focus groups.		
Police officers' experiences of supportive and unsupportive social interactions following traumatic incidents. Evans et al. 2013[56]	Research aims made clear in objectives section. Overall aim 'was to understand the types of support processes that might promote resilience'. Methods are appropriate as experiences, and other subjective outcomes are best measured qualitatively.	Semi-structured interview design appears reasonable to explore the proposed aims, but choice of qualitative study design not directly justified.	19 Police officers. A snowballing approach was used to recruit officers, which allowed detection of information-rich participants. By limiting participants to those who have served over 2 years, the chance of traumatic incident exposure was increased. Limiting participants to those without diagnosed PTSD was appropriate as the study measured resilience-promoting investigations. Non-participation was not discussed.	An interview schedule was used for the semi-structured interviews, although only the broad focus of the schedule was provided by the authors. Interviews were audio-recorded and transcribed verbatim. No information was given regarding interview setting, and data saturation was not discussed. No justification for choice of methods.	Researchers acknowledge how their role as researchers could have affected the data, giving social desirability bias as an example.	A National Health Service ethics committee granted ethical approval, and participants provided informed consent. No information is provided regarding efforts to maintain confidentiality.	The process of thematic analysis was described in detail. All themes generated can be linked with relevant first order data. Contradictory data is taken into account and present within the themes. Three authors were involved in the analytic process to minimise bias. One author had the main role in the analysis, while two others cross-referenced certain parts of their work in order to increase credibility. All 3 researchers reached a consensus on unclear issues before the final themes were decided upon.	Themes are clearly summarised, with relevant quotations to support each theme. Findings are discussed in relation to the research aims. Three researchers were involved in the analytic process.	The researchers provide detailed recommendations for further quantitative research. Furthermore, the researchers recommend similar qualitative research in a population that has a history of PTSD, so that comparisons can be made.
Interventions for critical incident stress in emergency medical services: a qualitative study	Clear statement of study aims, with overall study purpose ' to facilitate development of interventions that	Explanations were provided for how the focus groups and semi-structured interviews were conducted. E.g	4 supervisors, 54 front-line ambulance workers. Participants were sampled to ensure all job levels and	The setting of the data collection away from the workplace was stated but not explained. Detail was given about	Researchers do not critically examine their role, potential bias and influence of research	Ethical approval was obtained from a research ethics board, and participants signed a consent form. No further	The authors described how ethnographic content analysis was used to generate thematic codes, using a	The findings are clearly presented, using relevant participant quotations to support the authors	The research conclusions appear relevant to all EMS organisations which operate in the format of

Halpern et al. 2008[43]	take into account EMS culture.' Examining experiences adequately requires qualitative methodology.	flexible interview structure ' to permit the elaboration of more in-depth or emotionally significant data' and focus group size of 4-8 members 'to maximize interactive data'. No justification given for choice of interviews and focus groups.	genders were represented. Researchers state that participants were recruited from a specific cohort at a mandatory training conference, but no detail is given about what information was provided to the participants to entice them to enter the study. The sampling process was iterative- preliminary analysis informed subsequent sampling decisions. No further participants were recruited once saturation was reached.	the length of interviews and focus groups, and the main topics of questions asked during interviews and focus groups were stated. The researchers stated they had reached saturation by the end of the study. Data obtained was in the form of audio recordings, written transcripts and written notes of non-verbal communication information as observed by the researchers.	question formulation or data collection.	information was given regarding any ethical safeguards in place.	constant comparative method to categorise themes. Initial and final codes are described. The authors explain why textual data was not analysed for focus groups. Member validation was carried out 6 months post initial data collection. Contradictory data are presented and discussed, and sufficient data are presented to support the findings.	interpretations. 3 analysts were used during the data analysis process. Member validation was carried out 6 months after data was gathered. Contradictory viewpoints are taken into account and discussed.	using supervisors. The authors provide suggestions for interventions which are of relevance to EMS organisations. The authors recommend that the interventions of:<br>1. Supervisor support<br>2. Timeout period post-incident<br><br>should be further researched.
Barriers and Facilitators to Seeking Mental Health Care Among First Responders: "Removing the Darkness" Jones et al, 2020[62]	Aims are clearly stated and the study's importance justified. The authors aims include exploring organisational culture and perceptions of individuals, for which qualitative research is appropriate.	The decision to use individual ethnographic interviews the qualitative method of choice is well explained. The authors also explain why they chose a 'community-based approach'.	32 firefighters and/or EMTs/paramedics .<br><br>This was a convenience sample. The principal investigator facilitated recruitment by developing relationships with community partners who each recruited participants from	Ethnographic ndividual interviews were conducted with participants by the principal investigator. An interview guide was used and attached, which was developed by the PI based on the study aim and input from a qualitative methods expert,	The principal investigator acknowledged how being married to a firefighter/paramedic promoted buy in from the community during the recruitment stage.	Ethical board approval was gained. 'Prior to starting the interview, the PI reviewed the study information sheet and completed the consent process with each participant. ' Resources for further emotional support were provided to participants in case of emotional	Content analysis and constant comparison methods were used during the analytic process. Themes which emerged during data analysis were checked with participants in later interviews. Two researchers independently analysed the transcripts, and discussed the process until	Findings are presented as themes, with participant quotations included for each theme to support the author's interpretations. Credibility was enhanced by the use of more than one analyst, and member validation. Findings are discussed in relation to the	The authors make implications for practice, identifying 'need for improving education and awareness regarding duty-related MH problems.' The authors also make implications for nursing practice, and implications for future research.

			their own organisations. They did this by distributing e-mails on behalf of the principal investigator. Reasons for non-participation: 'individuals not answering or returning calls (all were called twice), scheduling conflicts'	along with feedback from community partners to strengthen trustworthiness and validity. Interviews were audio recorded and transcribed. Field notes were also conducted by the authors. Data collection setting was decided on the basis of convenience for participants. Data saturation was reached.		distress experienced during the study.	consensus was reached. (on 80% of top level codes). Exploration of new themes was stopped when saturation was reached. participant quotations are provided throughout to support the author's interpretations. The researchers acknowledge a bias risk due to the principal investigator conducting all the interviews. Sufficient data are presented to support the findings, and contradictory data are reported (such as positive and negative experiences of therapists).	original research question.	
Exposure to human tragedy, empathy, and trauma in ambulance paramedics. Regehr et al, 2002[40]	Aims of study are clearly stated, and their importance justified. Qualitative methods are appropriate because the authors want to explore experiences in depth.	Choice of qualitative study design not explicitly justified.	18 paramedics. 'Purposive sampling was used to ensure that participants represented a wide range of experiences in terms of length of time with the service and types of events encountered.' Non participation	A semistructured interview guide was used to conduct interview, and the authors provided examples of guide topics. These interviews were audio-recorded and transcribed, and field notes were taken. The authors acknowledge they	Researchers do not critically examine their role, potential bias and influence of research question formulation or data collection. No mention of changing the research design as the study progressed.	No mention of ethical approval, or consent gained from participants. No attempt is made to explain how confidentiality was maintained.	Nvivo was used to aid in data analysis. Open coding was used initially to generate broad categories. Towards the later stages of the process, selective coding was used to develop a meaningful narrative of the experiences of the	Findings are presented in sections headed by themes. Author's comments are supported by first order interpretations. Triangulation with members of emergency service organization was carried out	The authors acknowledge that the study is 'not intended to reflect the views of all paramedics in all organizations; rather, it describes a particular phenomenon experienced and described by one group of paramedics and

			was not discussed.	required a larger than normal sample size to achieve saturation. No information is provided for the setting of data collection.<br><br>'Other sources of data included the notes recording the interviewer's impressions.'			workers. Interviewers also recorded field notes. Triangulation with members of emergency service organization was carried out throughout the research, and analysis of data was discussed. Two members of the research team developed the coding tree together. A third member reviewed the open and selective coding. It is not clear how the themes were derived from the data, but sufficient data are presented to support the findings. Contradictory data are included in the findings and discussed.	throughout the research, and analysis of data was discussed. Two members of the research team developed the coding tree together. A third member reviewed the open and selective coding.	points to some interesting avenues for further consideration. These include future attempts to measure cognitive and emotional empathy in paramedics and assess the impact of these strategies on posttraumatic and depressive symptoms and on social supports.'
The meaning of traumatic events as described by nurse sin ambulance service Jonsson et al. 2003[44]	Aims of study are clearly stated, and their importance justified. Qualitative methodology is appropriate due to nature of the aims, which is to explore subjective experiences in detail.	A phenomenologica l approach was used, and the reason for its us thoroughly explained. The use of self-reports over the use of interviews was not justified (the authors said that interviews and self-reports are	240 medical technicians and 122 registered ambulance nurses. The authors asked 500 participants of another study to write down their experiences with traumatic events. No detail is given how the sample of the	The question the participants were asked to answer was: 'Write down and describe a situation which you experienced as a traumatic event''. From the data returned by the participants, the authors selected 25% of the written	Researchers do not critically examine their role, potential bias and influence of research question formulation or data collection. No mention of changing the research design as the study progressed.	Ethical approval was granted. Consent was implied through willingness to participate in the study, but no information is given regarding a signed consent form. In addition, participants were assured that they could withdraw	Van Kaam's scientific explication was used to analyse the written stories. Detail was provided about the steps of this thematic analytic process. Only 25% of the written stories were further analysed, and	The findings are in the form of themes, supported by first order data and the author's interpretations. The findings are related to the original research question in the discussion. There is no mention of any efforts to increase the	The authors recommend specific areas for further research, limited to the ambulance sector. The organisational interventions recommended by the authors are likewise limited to the ambulance sector.

		the most common methods in empiric-phenomenological studies.	other study was recruited. Non participation is not discussed.	accounts to further analyse. These written accounts were strategically selected based on having the fullest description of a traumatic event. Details of the analytic process are provided. Saturation of data is not discussed.		from the study at any time. The questionnaire was not labelled with the name of the participant. Confidentiality was guaranteed by eliminating names or other identifying characteristics from the essays.	were strategically selected for being more data-rich. There is no information to reveal how many researchers are involved in the analytic process. Sufficient data is presented to support the findings. There is no mention of contradictory data being taken into account. The researcher does not critically appraise their own potential bias.	credibility of the findings.	
Guilt, shame and need for a container: a study of post-traumatic stress among ambulance personnel Jonsson et al. 2004[45]	Aims of study are clearly stated, and their importance justified. Qualitative methodology is appropriate due to nature of the aims, which is to explore subjective experiences in detail.	Descriptive phenomenology was 'chosen for its potential to grasp the meaning of such phenomena through the description of lived experiences.'	10 Ambulance nurses or ambulance technicians. Insufficient information on how participants were The only relevant information is that participants were strategically selected to obtain a 'variation of sex, age, educational background, and experience as ambulance staff.'	Setting for interviews was described but not justified. Interviews were open in structure, beginning with the question "tell about a traumatic event that you have experienced". 'do you mean?" or "How did you feel about that?". The interviews, taking about an hour and a half each, were audio-taped and transcribed word by word.' Reason for choice of interview as method of data collection not	Researchers do not critically examine their role, potential bias and influence of research question formulation or data collection.	The study was described, the extent of participation was explained, and potential risks were explored with the participants, who were assured that they had the right to withdraw from the study at any time. The study was approved by the Ethical committee at Göteborg University. To protect participant confidentiality, no quotations were provided.	The authors provide details about the 5 steps of the analytic process, with the latter steps being repeated once the author was familiar with existing philosophy. Due to the authors not using participant quotations (to protect participant confidentiality), it is not possible to determine whether the author's findings are supported by first order interpretations. Contradictory data are discussed, but	Findings are displayed under relevant sub-headings. In the discussion section, there is some debate for and against the researcher's arguments. No attempts made to demonstrate efforts to enhance credibility.	The results can be applied to emergency medical organisations, however caution should be taken due to the small sample size and lack of information about participant demographics. The authors recommend for the insights gained by this study to be ' distributed to all ambulance managers and other relevant personnel categories. ' The authors make specific

				explained. Saturation of data not discussed.			without the direct participant quotations.		recommendations for management.
Situation Critical: High Demand, Low Control, and Low Support in Paramedic Organizations Regehr et al. 2007[41]	Aims of study are clearly stated, and their importance justified. Qualitative methodology is appropriate due to nature of the aims, which is to explore subjective experiences in detail.	Long interview design was chosen 'to explore in detail the experiences of paramedics in their organizations including their roles, the demands placed on them, the control over the working environment that they experience, and the supports that they receive.'	17 paramedics. Paramedics were purposively sampled from the sample of participants partaking in the quantitative component of the study. Purposive sampling was used to ensure that participants represented a wide range of experiences in terms of length of time with the services and types of work experiences encountered.	A long interview method was used to collect data. The interviews followed an interview guide (not provided), were audio recorded and transcribed verbatim. The interviewer made their own notes during the interview. Setting of data collection not provided, saturation of data not discussed.	Researchers do not critically examine their role, potential bias and influence of research question formulation or data collection.	No mention of ethical approval, or consent gained from participants. No attempt is made to explain how confidentiality was maintained.	Data analysis commenced with open coding that captured a broad range of perspectives, whereas axial and selective coding facilitated the structuring of a coding framework. After the initial coding framework was developed, the transcribed interviews were imported into N*VIVO, a computer generated data analysis system, and the interview data were coded by multiple coders and subjected to detailed thematic analysis. In the final stage of analysis, constant comparative method of data analysis was implemented to compare categories and themes across respondents. Furthermore, paramedic organisations were consulted when creating the	Findings are presented in themed subheadings. Authors claim to have enhanced credibility through ' prolonged engagement and persistent observation'. No mention of more than one analyst, and qualitative findings were triangulated with quantitative results.	The authors make recommendations for EMS organisations, based on a mixture of their quantitative and qualitative results.

							research questions, and throughout the research project to guide it with feedback. Sufficient data are presented to support the findings, and contradictory data taken into account.		
Emergency Medical Services Provider Perspectives on Pediatric Calls: A Qualitative Study Jessica et al. 2019[46]	Aims of study are clearly stated, and their importance justified. Qualitative methodology is appropriate due to nature of the aims, which is to explore subjective experiences in detail	The use of focus groups was not explicitly justified.	17 EMS providers. ' Paramedics and EMTs were recruited for the study through invitations circulated via weekly staff emails, flyers posted at ambulance bases, the agency's social media page, and e-mails from the EMS chaplain. Interested participants were instructed to contact a single study investigator by phone or email 'Selection criteria were applied to the participants in order to sample for the most relevant participants- participants had to be working for over a year in order to increase chance of	The process of focus group conduction was thoroughly described. A semi-structured guide was used by the researcher to conduct the focus groups- the same researchers conducted all the focus groups. At the end of the focus group, themes were fed back to participants, who were invited to comment. Focus groups were conducted at a large ambulance service. Audio recordings were transcribed. A second researcher was present during the focus group to take notes. Thematic saturation was reached.	The researchers reflect on the potential biases of the study in the limitations section. They acknowledge that by using discussion prompts, they may have steered the conversation away from topics that the participants would otherwise have talked about. Furthermore, they acknowledge that the fact that a single coder was responsible for coding 80% of the data alone may be a source of bias.	Participants gave informed consent and ethical approval was gained from an ethics committee. Resources were provided to the participants in case of experiencing psychological distress.	Transcripts were analysed using directed content analysis. Two reviewers independently coded 20% of the transcripts, and a single researcher coded the remaining data. Findings were validated through groups consensus. Quotations are provided for all sub-themes. Authors acknowledge the limitations of focus groups, and the potential for bias with one researcher coding 80% of the data. The authors also acknowledge how the discussion guide prompts may have altered the topic of discussion of participants from what they would otherwise have	Findings are presented as sub-themes, with quotations supporting each sub-theme in a separate table. Credibility of findings was enhanced by reviewing the focus group themes with participants at the end of the focus group. Furthermore, some of the data was reviewed by two researchers.	The findings are relevant to EMS organisations, specifically EMS leaders. Although Desired support mechanisms following difficult paediatric calls can be extrapolated to other types of traumatic events.

			traumatic incident exposure. Non participation was not discussed.				talked about. Contradictory data are taken into account.		
An assessment of the need of police officials for trauma intervention programmes - a qualitative approach. Boshoff et al. 2015[57]	Aims of study are clearly stated, and their importance justified. Qualitative methodology is appropriate due to nature of the aims, which is to explore subjective experiences in detail.	Focus groups were chosen as the method of data collection in order to 'allow the researcher to gain insight into participants' beliefs about and perceptions or accounts of a particular topic'. The researchers state: The focus groups allowed the researcher to interact systematically and simultaneously with several individuals.	40 police officials. Participants were recruited via a purposive sampling method through the health and wellbeing departments, which were used as an intermediary between the researchers and participants. Police officials were purposefully chosen considering the relevance of the topic, specifically referring to their exposure to trauma, resultant symptoms of PTS and their participation in trauma intervention programmes. Non-participation was not discussed, other than reassuring participants that non-participation would not be sanctioned.	Setting for data collection was not outlined. Three focus groups were carried out, and semi-structured interviews were performed within the focus groups. Exact questions asked are provided by the researchers. The sessions were audio recorded, transcribed and analysed. Saturation of the data is not discussed.	Researchers do not critically examine their role, potential bias and influence of research question formulation or data collection.	Ethical approval was gained for this study from the North-West University's ethical committee . Informed consent was taken by the researchers. ' Participants were furthermore encouraged to withdraw from the focus groups at any time should they feel uncomfortable or experience any harm or emotional consequence as a result of participating in the study.'	The authors describe an 8 step method of thematic analysis, but do not explain how they will apply this method. It is not clear how the data is used to arrive at the themes generated. Sufficient data are presented to support the findings. Contradictory data are taken into account.	Findings are presented as sub-themes, supported by sufficient quotations. There is limited discussion for and against the researchers' arguments. No efforts are made to enhance credibility of findings.	The authors give a variety of 'preliminary indicators' for which a ' purposeful psycho-social trauma intervention programme' is indicated. The generalisability of this is limited to police forces.
A preliminary investigation of post-traumatic stress symptoms among firefighters	Aims of study are clearly stated, and their importance justified. Qualitative methodology is	One-to-one interviews were chosen but reasons for their use over focus groups were not	11 Firefighters, 8 Station Officers, 4 Sub Officers, 4 Leading Firefighters, 2 Fire Control	' One-to-one interviews were conducted in a private room with participants. They lasted or up to 90	Researchers do not critically examine their role, potential bias and influence of research	No mention of informed consent, ethical approval or methods to ensure confidentiality.	'The interviews were recorded and fully transcribed and the data were analysed by	Findings are presented as sub-headings of themes, with participant quotations	The authors provide recommendations for fire organisations. These

Haslam et al. 2003[60]	appropriate due to nature of the aims, which is to explore subjective experiences in detail.	explained. The interview schedule was developed in conjunction with psychologists working within the fire service, as well using relevant literature.	Officers and 2 Area Divisional Officers. No information provided on how participants were recruited. 'The sample was selected to cover the range of positions in the service and the proportion of respondents in each position broadly reflects the profile of the service.'	minutes. The interview questions were stated by the researchers. Open-ended questions would be used to explore participants' feelings towards incidents. ;The interviews were recorded and fully transcribed and the data were analysed by sorting verbatim material into emergent themes as described by Dey (1993).'' Choice of data collection methods or study setting was not justified. Saturation of data was not discussed.	question formulation or data collection. The researchers acknowledge that their small sample size may not be representative.		sorting verbatim material into emergent themes as described by Dey (1993). A second researcher independently checked the analysis to ensure analysis reliability' There is little transparency to show how the themes were developed from the primary data. Use of a second researcher enhances credibility. Direct participant quotations are sufficiently used to support the authors' interpretations. Contradictory data are taken into account.	supporting the authors' comments. Credibility is enhanced by the use of two analysts.	recommendations are concerned with efforts to improve staff wellbeing.
A qualitative study about experiences and emotions of emergency medical technicians and out-of-hospital emergency nurses after performing cardiopulmonary resuscitation resulting in death Fernández-Aedo et al. 2017[47]	Aims of study are clearly stated, and their importance justified. Qualitative methodology is appropriate due to nature of the aims, which is to explore subjective experiences in detail.	Both interviews and focus groups were used as methods of data collection, the reason for their use was not explained.	7 EMTs and 6 nurses. Snowball sampling was used to recruit the participants. As a prerequisite for their inclusion in this study, the health workers were required to have carried out at least 5 CPR techniques resulting in death over their entire professional career. No volunteer staff	Questions asked during the interviews were ' developed based on the reviewed literature and experts' opinions', but the authors do not provide a topic guide or examples of other prompts. ' The assignment of participants to' either individual semi-structured interviews or focus groups 'was	Researchers do not critically examine their role, potential bias and influence of research question formulation or data collection.	' The study was approved by the Ethics Committee of the University of the Basque Country.' 'echnique was based on their availability and preferences. Participants gave their written consent to participate in the study and to be recorded in audio and/or video format.'	A detailed description of the analytical process is provided. 'To ensure the quality of the interpretation and guarantee the reliability of the information obtained, a triangulation between all researchers involved in the interviews was also carried out. Any disagreement	Themes are not provided with any participant quotations. 'To ensure the quality of the interpretation and guarantee the reliability of the information obtained, a triangulation between all researchers involved in the interviews was also carried out. Any disagreement	The authors recommend greater training for health professionals when ' notifying bad news and providing psychological support to the family members of the deceased ' No recommendations for research are made.

			was included in the study. ‘ In order to ensure heterogeneity, the study included healthcare professionals of different ages, both genders and varying years of experience, working at different institutions.’ Non participation was not discussed.	based on their availability and preferences.’ ‘A total of 3 health emergency technicians and 3 nurses were interviewed individually for a total of 11---35 min by two of the researchers involved in the study. The focus group was comprised by 4 EMTs and 3 nurses, and it was used to triangulate the information obtained during the individual interviews. The group session lasted 76 min and was carried out by two researchers, one acting as a moderator and the other as an observer.’ Data saturation was reached.			was resolved by consensus.' No participant quotations are provided to support the authors' interpretations. Contradictory data re not taken into account.	was resolved by consensus.' Other than the use of multiple researchers in the analytic process, no other methods to increase credibility are discussed.	
Experiences of and actions towards worries among ambulance nurses in their professional life: A critical incident study Svensson et al. 2008[48]	Aims of study are clearly stated, and their importance justified. Qualitative methodology is appropriate due to nature of the aims, which is to explore subjective experiences in detail.	There is no justification for the choice of semi-structured interviews as a method of data collection.	25 ambulance nurses. Participants were strategically selected from three ambulance services based on ‘socio-demographic and professional characteristics such as age,	The authors explain the structure of the interviews, giving examples of the open-ended questions which they ask to participants at the start of the interview. The authors say the	‘As both the researchers and the nurses were familiar with the environment in which the study was conducted, the risk of misunderstanding during the interviews was minimised.'	‘The managers of the ambulance service involved gave approval for the study to be conducted in their department.’ The authors say ethical board approval is not required in Sweden under	'The interviews were also read through several times before categorizing them into sub-categories, in order to further improve security. The incidents were analysed according to	Findings are arranged into themes and sub-themes, and participant quotations are included for each theme to support the authors' conclusions. Credibility was enhanced via the	The authors recommend further research: 'This phenomenon should be studied more in-depth in order to map how the interaction with colleagues influences the worry among

			education level, sex and years in the profession'. Non participation is not discussed.	interviews took place where it best suited the participants, and three interviews were conducted over the phone. Researchers explain their use of interviews: 'Interviews were chosen as the data collection method, which allowed the respondents to describe their thoughts in more detail with the help of follow-up questions.' The authors also justify the use of the CIT method. Interviews were audio recorded and transcribed verbatim. Saturation of data was not discussed.		certain circumstances. The authors describe how informed consent was taken from participants, including assurances that data will be treated confidentially. 'The study adhered to the principles outlined in the Declaration of Helsinki'	character and content. This step was repeated several times before the end result emerged. The categorizing of incidents into sub-categories was conducted in cooperation with the second researcher, experienced both in theory and practice, which minimised the risk of subjectivity (Andersson and Nilsson, 1964). Direct quotes from the interviews strengthened the accuracy of the study.' Contradictory data were taken into account, and sufficient are presented to support the findings. Saturation of data was not discussed.	use of two researchers when categorising incidents. Authors consider contradictory data in the discussion.	ambulance nurses.' Some recommendations are also made for employers of nurses.
Exploring the nature of resilience in paramedic practice: A psycho-social study Clompus et al. 2016[49]	Aims of study are clearly stated, and their importance justified. Qualitative methodology is appropriate due to nature of the aims, which is to explore subjective	The authors used free association narrative interviewing, a technique which involves a preliminary narrative interview, followed by a semi-structured	'An advert, with a brief study outline, was placed in a regional Paramedic bulletin which was circulated electronically to staff with an invitation to	The researchers justify their use of using Free association narrative interviewing. Saturation was achieved after the 6th interview. Details of the exact two stages	Researchers do not critically examine their role, potential bias and influence of research question formulation or data collection.	' Ethical approval was received from a (NHS) research ethics committee and a university in the SW of England. Confidentiality and anonymity was secured through the use of	Details of the FANI analytic process were given by the researchers. 'Any discrepancies were examined and discussed until consensus was reached. Trustworthiness	Themes are presented as sub-headings, and supported by the direct participant quotations. Member validation was carried out to improve credibility.	The authors relate the findings of the research to the theories of exiting organisational interventions, eg TRiM. The authors conclude that for front-line paramedics, ' applying

	experiences in detail.	interview. 'This enabled a deeper analysis of the affective and often unconscious aspects of paramedics' lives. '	participate.' Participants had to fulfill the following criteria: -Grade of paramedic, technician or emergency care practitioner -Willing to volunteer their time<br><br>Three out of the 10 individuals became unavailable, but the authors do not explain why.	of the interviewing process were provided by the authors. interviews were audio recorded and transcribed, and carried out at a place of the participants' choosing.		pseudonyms for participants, and all data were kept in a password protected personal computer with access limited to SC. Participants were made aware that they could withdraw at any time and that the anonymised data would be disseminated in various ways. Due to the potential distress that participation could inadvertently provoke, information on how to access counselling services was made available to all participants.'	and data credibility were established by several means including participants feeding into the study's aims, keeping contemporaneous notes, and sending interview transcripts and a summary of findings to each participant for verification'. The authors are not clear how many researchers are involved in the analytic process. For each theme, sufficient participant quotes are provided to support the authors' interpretations. Contradictory data were taken into account and discussed.		interventions and reviewing support mechanisms would seem to be a pressing imperative.'
First response emergency care – experiences described by firefighters Abelsson et al. 2019[61]	Aims of study are clearly stated, and their importance justified. Qualitative methodology is appropriate due to nature of the aims, which is to explore subjective experiences in detail.	The authors justify their choice of group interviews, and why limits are set on group interviews: 'The group sizes of eight and nine participants were considered acceptable to moderate and managed by the researcher.	Authors don't explain how participants were recruited into the study.	35 Firefighters. Setting for group interviews was not given. Participation numbers in the group interviews were provided and discussed. Justification for methods chosen was provided. A rough structure to the interviews was provided by	Researchers do not critically examine their role, potential bias and influence of research question formulation or data collection.	No approval has been sought from an ethics committee, which is in accordance with Swedish law. Consent was implied through participation in the study, following the delivery of verbal 'clear information' by the researchers.	The authors describe their method of text-driven, interpretive qualitative content analysis. This involved repeated readings of the transcribed interviews, and then identification of 'meaning units', then codes were derived, and	Findings are presented as four themes, with consistent use of primary quotations to support the researchers' arguments. The findings are discussed in relation to the original research question.	The researchers contrast their findings with other research in the field, such as in the case of spouses being used as sources of support by firefighters. They also recommend areas of future research, eg involving other strands of

		Interaction in the pre-existing group is key to a successful group interview. In this study, all firefighters worked at the same fire station, which promoted interactions optimal for the research purpose'		giving questions which were asked to participants. There is no mention of saturation of the data. The authors reflected in the 'limitations' section that group interviews could lead to participants being uncomfortable.		Methods taken to protect confidentiality are described.	formed into sub-categories and categories. Sufficient data are present to support the findings. Contradictory data are discussed. It is not clear how many researchers were involved in the analytic process. There is no explicit mention of efforts to improve credibility. Authors acknowledge that 'participants in group interviews may experience a pressure within the group, resulting in similar opinions'.		emergency service workers.
Paramedics' experiences with death notification: a qualitative study Douglas et al. 2013[50]	Aims of study are clearly stated, and their importance justified. Qualitative methodology is appropriate due to nature of the aims, which is to explore subjective experiences in detail.	The authors used focus groups as their method of data collection, but did not justify their use over other qualitative methods.	28 paramedics. Participants were self-selected. Recruitment messages were delivered via 'departmental e-mail, flyers, and by word-of-mouth'. Authors don't explain why the participants selected were the most appropriate for the study, and non-participation is not discussed.	The authors provide the locations of the focus groups, and give examples of questions asked during the sessions. There is no explicit justification for why focus groups were chosen over other qualitative methods. Methods were modified during the study as follows: 'The question guide was adapted as	The researchers acknowledged that their choice of focus group location, as well as the presence of the supervisor in the focus group may have affected the answers given by participants. The researchers do not critically examine their own role.	Ethical board approval was gained, and written consent obtained. No information is provided on efforts to maintain participant confidentiality.	The authors describe an inductive approach to data analysis. Two researchers were involved in the analysis process. Themes were discussed with a further two authors, until consensus was reached. Credibility was enhanced via the use of member checking the participant responses with	The findings are presented as themes, supported by participant quotations and authors' interpretations. Credibility was enhanced by distributing results from the study to participants for the purpose of generating feedback.	The findings are discussed in relation to existing literature, and the authors make recommendations for practice, specifically regarding paramedic training.

				required by the author and the facilitator after each session to clarify some questions based on the paramedics' responses. ' Focus groups were audiotaped and transcribed. Saturation was discussed: the authors kept recruiting participants until saturation was reached.			participants at the end of focus groups. Contradictory data are taken into account, and sufficient data are presented to support the findings.		
Peer-support: a coping strategy for nurses working at the Emergency Ambulance Service Carvello et al. 2019[51]	Aims of study are clearly stated, and their importance justified. Qualitative methodology is appropriate due to nature of the aims, which is to explore subjective experiences in detail.	The authors used semi-structured interviews. The authors explain their choice of questions result from pre-existing literature, but do not justify the use of interviews over other qualitative methods eg focus groups	'Participants were recruited on a voluntary basis from an emergency medical service in the north of Italy.' The sampling was 'non-probabilistic', according to the following criteria: nurses working at the emergency ambulance service "118" in a hospital in northern Italy, who had experience in the extra-hospital emergency. Non-participation is not discussed.	Data collection was at an italian emergency service organization called 118. The justification given by the researchers for choosing this location was to choose an environment familiar to the participants so that they would feel at ease. Semi-structured interviews are carried out with participants. The authors explain how they chose their questions, by taking inspiration from similar literature, but choice of	Researchers do not critically examine their role, potential bias and influence of research question formulation or data collection.	Ethical approval was gained from the Bioethical Committee of the University of Bologna. Informed consent was gained from participants before participation. Efforts to protect confidentiality are not discussed.	'The interviews were conducted and analyzed by all the researchers after having been faithfully transcribed on digital text documents, reporting in brackets some relevant non-verbal gestures, and after having evaluated the nodes and relationships generated by the nVivo qualitative research software12.' Although sufficient data are presented to support the findings, it is unclear how the	Findings are presented as themes. Authors discuss arguments for and against certain issues raised. One example of this is the inclusion of quotations of participants who prefer talking to a peer supporter, and those who prefer talking to a professional therapist. No mention is made of efforts to discuss the credibility of findings. Contradictory data are taken into account and discussed.	The authors relate their findings to their aims. They propose that a peer support program should be introduced in the ambulance service. No recommendations for further research are made.

				interviews over other qualitative methods is not explained. 'The interviews were audio-recorded and conducted anonymously'. Saturation of data is not discussed.			themes were generated from the data.		
Experiences among firefighters and police officers of responding to out-of hospital cardiac arrest in a dual dispatch programme in Sweden: an interview study Hasselqvist-Ax et al. 2019[63]	Aims of study are clearly stated, and their importance justified. Qualitative methodology is appropriate due to nature of the aims, which is to explore subjective experiences in detail.	The authors don't justify their use of semi-structured interviews over other qualitative methods.	10 police officers, 12 firefighters. Participants were purposively sampled for knowledge of two or more cardiac arrest situations, and to collect as rich descriptions as possible. Three recruitment approaches were used: '1) an invitation letter from the researchers was presented to the main collaboration group for OHCA alarms in Stockholm County; 2) on the police report for cardiac arrest alarms there was a request to contact the researchers for a voluntary interview; 3) fire stations were directly contacted for recruitment of participants.' Non-participation	The authors provide an interview guide with 7 open-ended questions, which form the basis for the semi-structured interviews. Setting for interviews: all but three took place at regular work places (not known where the others took place). Critical interview technique (CIT) was chosen as the method for data collection. The authors don't justify their use of this technique, but they justify their sample size based on the recommended sample size for CIT. Interviews were recorded and transcribed. The authors state no modification of methods during study were necessary. Saturation was	All interviews were conducted by the same author, to increase the chance that they were conducted in a similar way. The authors list the researchers' relevant strengths and past experiences, demonstrating reflexivity.	Ethical approval was obtained. Written informed consent was obtained from the participants and information was given about the possibility to withdraw from the study without any reprisal. Participants were not entitled to financial remuneration or other benefits.	'This was an interview study where data were analysed by using critical incident technique (CIT) and inductive qualitative content analysis.' The authors explain the CIT process step by step, and provide an example with a piece of interview data. To enhance credibility, the researchers discussed the analysis at each step of the process, ensuring all analyses were supported by data. Occasionally the views of participants are summarised without examples of quotations. Contradictory data are taken into account, but saturation of data is not discussed.	The findings are presented as themes. Although participant quotations were provided, the authors didn't provide quotations for all of their comments. Often, the views of participants were summarised without providing participant quotes.	The authors provide recommendations for swedish emergency organizations, such as giving indications for training of paramedics and firefighters.

			was not discussed.	discussed in relation to the critical incident technique- where 20 interviews (of 2-4 CIs per interview) provide sufficient data. The authors included 22 participants.					
Working in prehospital emergency contexts: Stress, coping and support from the perspective of ambulance personnel Oliveira et al. 2019[52]	Aims of study are clearly stated, and their importance justified. Qualitative methodology is appropriate due to nature of the aims, which is to explore subjective experiences in detail.	The authors don't explain why semi-structured interviews were chosen over other qualitative methods.	14 ambulance personnel. Participants were recruited from seven institutions, mainly on the basis of 'having an active status on a rescue team and being willing to talk and share their experiences'. Details of how participants were recruited are not provided, and non-participation was not discussed.	Interviews took place at the participants' local professional organisation, and were all conducted by the first author. Semi-structured interviews were conducted with participants following an interview guide, details of which are provided by the authors. The choice of semi-structured interviews was justified as it allowed the authors 'to obtain in-depth information regarding participants' prehospital emergency experiences'. Interviews were audio-recorded and transcribed. Saturation of data was achieved.	The researchers acknowledge the possibility of researcher bias during the coding process, and therefore they discuss their analyses with other researchers.	The research project was approved by the Portuguese Red Cross and by the University's Ethics Committee Written consent to participate in the study and to audio-record the interview was obtained, as follows: 'We sent a cover letter to all selected structures, explaining the aim of the study, the procedures, the ethical issues guaranteed, the voluntary character of the participation and the possibility to withdraw at any time'.	The authors use Braun and Clark's analytic approach, and outline the process in detail. This includes explaining how themes were derived from the data. Data collection was performed until saturation was reached. Data was discussed between the researchers during coding, enhancing credibility. The researchers made efforts to reduce researcher bias: 'Furthermore, to reduce research bias, there was a concern to engage with other researchers to discuss the process of data analysis and to illustrate themes with verbatim descriptions from participants.' Sufficient data are	Findings are presented as themes. The researchers provide participant quotations to support their findings. Multiple analysts discussed the analytic process to reduce researcher bias.	The researchers make recommendations to ambulance organisations to improve the psychological wellbeing of their staff. The authors also provide multiple avenues of recommended further research.

							presented to support the findings, and contradictory data are taken into account.		
Exploring paramedic communication and emotional expression in the workplace after responding to emergency calls Drewitz-Chesney et al. 2019[53]	Aims of study are clearly stated, and their importance justified. Qualitative methodology is appropriate due to nature of the aims, which is to explore subjective experiences in detail.	The authors don't explain why semi-structured interviews were chosen over other qualitative methods.	8 paramedics. The recruitment strategy is explained in detail. Participants were recruited over facebook groups. 'Participants were sampled using convenience, then purposive sampling. Convenience sampling enabled initial recruitment. Snowball and quota sampling were the two forms of purposive sampling used.' Authors are not clear on which attributes are sampled for. The authors justified their choice of participants by saying that 'when sufficient information is gleaned from participants, a smaller sample size is required '	Semi-structured interviews were performed with participants, but the researchers do not justify why interviews are chosen. Interviews were conducted using Skype, and participants could choose to interview via video or audio only. An interview guide is provided. Interviews were audio recorded and transcribed, and data collection was stopped once saturation was reached.	The recruitment messages mention that the researcher previously worked as a paramedic. The researchers acknowledge the possibility of confusion and bias that can arise from this, although this was minimised by the fact that the researcher didn't work with any of the participants.	'This study received ethical approval from the University of Edinburgh's Usher Research Ethics Group. Each participant provided verbal and written consent. To minimise the risk of psychological impact, participants were never asked to recall specific calls or details. Participants were monitored for signs of distress during each interview (none were noted). BCEHS paramedics have access to three services offering support and counselling. These services were listed on their information letters. At the conclusion of each interview, participants were asked if they wanted a referral to any of the services, which	The authors used constructivist grounded theory in their analytic process. The authors are transparent about how themes are generated from the data.' Credibility was enhanced through triangulation, including interviewing the manager of the BCEHS CIS program, Marsha McCall, and Anonymous, a retired BCEHS paramedic, whom substantiated some participant data . Thick descriptions and a diverse sample contributed to credibility and transferability. An audit trail was maintained throughout the research process which enhanced dependability. Journaling and bracketing enriched reflexivity ' 'Each transcript was read at	Quotations are interspersed among the results which are presented as themes. Credibility was enhanced through the use of triangulation with leading members of the organisation.	The researchers consider their findings in relation to existing literature. They also make various recommendations for emergency service organisations, relating to post-incident organisational interventions that could protect paramedic wellbeing.

						all participants declined.'	least three times to improve accuracy and familiarity'. Sufficient data are presented to support the findings, and contradictory data are taken into account.		
Mental Health in the UK Police Force: a Qualitative Investigation into the Stigma with Mental Illness Edwards et al. 2020[58]	Aims of study are clearly stated, and their importance justified. Qualitative methodology is appropriate due to nature of the aims, which is to explore subjective experiences in detail.	The authors justify their use of open-ended questions in semi-structured interviews to facilitate meaningful discussion, although the choice of interviews other qualitative data collection methods is not justified.	Five police officers. 'Participants were recruited through a referral from a charity, personal network, police forum and contact from within the police force.' The authors outline the criteria for participant inclusion in the study. They do not however justify their choice for these criteria. Non-participation was not discussed.	Semi-structured individual interviews were conducted using a topic guide, of which the authors provide details. The authors justify their use of open-ended questions. Interviews were audio recorded and transcribed. Data saturation was not discussed.	Researchers do not critically examine their role, potential bias and influence of research question formulation or data collection.	Ethical board approval was obtained, as well as written consent from the participants. The researchers also had measures in place to support participants who experience distress due to participation in the study.	The authors used Braun and Clark's approach of thematic analysis. They outlined this process in detail, giving examples of how they arrived at themes from the data. It is unclear whether multiple researchers collaborated to compare coding. Participant quotations are consistently embedded within the authors' interpretations, and contradictory data are taken into account. The authors are transparent about how themes were generated.	Findings are presented as themes and sub-themes. The authors include contradictory data, and discuss this. Discussion takes place throughout the study within the results section, but there is also a separate discussion section.	The authors identify areas where further research would be valuable, such as barriers to help-seeking in male dominated professions. Recommendation s are also made to police organisations, such as highlighting a need to increase mental health awareness.
Living in Critical Times: The Impact of Critical Incidents on Frontline Ambulance Personnel: A Qualitative Perspective	Aims of study are clearly stated, and their importance justified. Qualitative methodology is appropriate due to nature of the aims, which is to	The authors don't justify why they chose individual interviews over other qualitative methods. They do however justify their use of some closed questions (	21 EMTs, 6 EMCs. The qualitative component of this study followed a quantitative Survey. Participants from the earlier study	Setting for data collection is not stated. The authors give examples of the topics discussed during the interview process. The interview	Researchers do not critically examine their role, potential bias and influence of research question formulation or data collection.	No mention of informed consent, ethical approval or methods to ensure confidentiality.	Thematic analysis was undertaken. 'A random sample of transcripts was read and coded by both authors in order in ensure good reliability and	Findings are presented as themes. For each theme, a range of relevant quotations are provided to support this theme. Credibility	The authors make recommendations to police organisations for post critical incident support for staff.

Gallagher et al. 2007[54]	explore subjective experiences in detail.	to elicit background information and to facilitate comparisons across participants.)	were asked if they would be willing to participate in qualitative interviews. Non-participation is not discussed.	schedule used was created with the help of a literature review and the findings from the quantitative component of the study. No justification for the choice interview method is given. The interviews were audio recorded and transcribed.			validity.' It is not clear how the themes were derived from the data. The authors don't critically evaluate their own role in the analytic process. Contradictory data are taken into account- for example when giving examples of participant quotations with conflicting attitudes. The researchers don't critically examine their own role. Sufficient data are presented to support the findings.	of findings is not discussed.	
Police officers, mental (ill-) health and spoiled identity Bullock et al. 2018[59]	Aims of study are clearly stated, and their importance justified. Qualitative methodology is appropriate due to nature of the aims, which is to explore subjective experiences in detail.	Researchers justified their use of telephone interviews: '. While telephone interviews are often depicted as a less attractive alternative to face-to-face interviewing, telephones may allow respondents to feel relaxed, more able to disclose sensitive information and there is little evidence that they produce lower quality data '	52 police officers, two police staff, four Police Community Support Officers (PCSOs) (four) and one special constable. Participants were recruited from six police constabularies in England and Wales. 'Individual participants were identified by virtue of their contribution to an online survey on the nature of work-related injury in which	The researchers explain in detail how the telephone interviews were conducted. Open questions were asked by the participants. Saturation of data is not discussed. 'All interviews were digitally recorded, professionally transcribed and anonymized. '	The researchers acknowledge that the nature of the interview is sensitive, and that the nature of telephone interviews would make participants more comfortable disclosing such information.	' The nature of the interviewing was inevitably sensitive and mechanisms were put in place to mitigate that; for example, interviewees were provided with the contact details of sources of support.' There is no mention of ethical board approval, informed or methods to protect patient confidentiality.	The authors use Braun and Clark's thematic analysis approach, and give a very brief overview of the approach, but they do not give examples of how themes were derived from the data. The researchers didn't critically examine their own role. Sufficient data are presented to support the findings, and contradictory data are taken into account. Data	The findings are presented as themes. For each theme, the authors use participant quotations as well as relevant literature to discuss the context surrounding the quotations. No efforts to enhance credibility are discussed.	The authors make recommendations to police organisations based on their findings surrounding stigma. No recommendations for research are made.

			they identified themselves as willing to participate in a full-length interview'. This study is part of a wider project. Non-participation was not discussed.				saturation is not discussed.		
'You see a baby die and you're not fine:' a case study of stress and coping strategies in volunteer emergency medical technicians Folwell et al. 2018[55]	Aims of study are clearly stated, and their importance justified. Qualitative methodology is appropriate due to nature of the aims, which is to explore subjective experiences in detail.	The authors justify their choice of in-depth interviewing technique as they 'hope to gain a deeper understanding of the lived experiences of voluntary EMTs'.	25 EMTs. Participants were recruited from one county in a Western state. It is not clear how the participants were selected, and non-participation is not discussed.	Interviews were conducted in a private space of the participant's fire department. Justification for choice of interview setting was not provided. In-depth interviews were performed with participants, using an interview guide. This was chosen to "gain a deeper understanding of the lived experiences of voluntary EMTs". Saturation was reached by the end of the study. Interviews were audio recorded and transcribed.	'The research team consisted of a female Caucasian professor who holds a doctorate and a male Caucasian undergraduate student with three years' experience as a volunteer EMT. While some of the interviews were conducted by both members of the research team, most interviews were conducted by the volunteer EMT'. Other than providing these details, Researchers do not critically examine their role, potential bias and influence of research question formulation or data collection.	Confidentiality was guaranteed by the authors as the transcripts were anonymised. There is no mention of ethical board approval or informed consent.	The authors describe the process of constant comparison analysis. Two researchers independently read all the transcripts, and collaborated to discuss findings. The authors are very transparent about the analytic process. Contradictory data were discussed, such as in the example of participants discussing the pros and cons of CISD. Sufficient data are presented to support the findings.	Findings are presented as themes and sub-themes. 'To enhance credibility, member validation was performed in which a summary of findings and initial interpretations of data were given to five participants to confirm the researchers accurately depicted viewpoints and experiences. To enrich transferability, we provided detailed descriptions of participants and research sites. To improve dependability, we used the same protocol for each interview and documented the process of data collection and analysis. To	The authors make four detailed recommendations to the specific EMS organisation with which the study was involved. The authors also make recommendations for future research.

								address confirmability, we used direct quotes from participants to support the finding'	
