# Supplementary material for: Mental health and help seeking among trauma-exposed emergency service staff: a qualitative evidence synthesis
Source: BMJ Open. 2022 Feb 2;12(2):e047814. doi: 10.1136/bmjopen-2020-047814 (PMC8811562; doi:10.1136/bmjopen-2020-047814)
Supplement: Author's manuscript [file bmjopen-2020-047814.draft_revisions.pdf]

BMJ Open

BMJ Open is committed to open peer review. As part of this commitment we make the peer review history of every article we publish publicly available.

When an article is published we post the peer reviewers' comments and the authors' responses online. We also post the versions of the paper that were used during peer review. These are the versions that the peer review comments apply to.

The versions of the paper that follow are the versions that were submitted during the peer review process. They are not the versions of record or the final published versions. They should not be cited or distributed as the published version of this manuscript.

BMJ Open is an open access journal and the full, final, typeset and author-corrected version of record of the manuscript is available on our site with no access controls, subscription charges or pay-per-view fees (http://bmjopen.bmj.com).

If you have any questions on BMJ Open's open peer review process please email info.bmjopen@bmj.com

BMJ Open

# The barriers and facilitators to help-seeking and recovery among psychologically distressed emergency service staff: A thematic synthesis



| | |
|---|---|
| Journal: | *BMJ Open* |
| Manuscript ID | bmjopen-2020-047814 |
| Article Type: | Original research |
| Date Submitted by the Author: | 09-Dec-2020 |



| | |
|---|---|
| Complete List of Authors: | Auth, Niklas; University of Birmingham, College of Medical and Dental Sciences<br>Wild, Jennifer; University of Oxford, Experimental Psychology<br>Booker, Matthew; University of Bristol, School of Social and Community Medicine<br>Riley, Ruth; University of Bristol, Social and Community Medicine; University of Birmingham, of Applied Health Research |



| | |
|---|---|
| Keywords: | QUALITATIVE RESEARCH, MENTAL HEALTH, OCCUPATIONAL & INDUSTRIAL MEDICINE, Health policy < HEALTH SERVICES ADMINISTRATION & MANAGEMENT |


SCHOLARONE™
Manuscripts

*I, the Submitting Author has the right to grant and does grant on behalf of all authors of the Work (as defined in the below author licence), an exclusive licence and/or a non-exclusive licence for contributions from authors who are: i) UK Crown employees; ii) where BMJ has agreed a CC-BY licence shall apply, and/or iii) in accordance with the terms applicable for US Federal Government officers or employees acting as part of their official duties; on a worldwide, perpetual, irrevocable, royalty-free basis to BMJ Publishing Group Ltd ("BMJ") its licensees and where the relevant Journal is co-owned by BMJ to the co-owners of the Journal, to publish the Work in this journal and any other BMJ products and to exploit all rights, as set out in our licence.*

*The Submitting Author accepts and understands that any supply made under these terms is made by BMJ to the Submitting Author unless you are acting as an employee on behalf of your employer or a postgraduate student of an affiliated institution which is paying any applicable article publishing charge ("APC") for Open Access articles. Where the Submitting Author wishes to make the Work available on an Open Access basis (and intends to pay the relevant APC), the terms of reuse of such Open Access shall be governed by a Creative Commons licence – details of these licences and which Creative Commons licence will apply to this Work are set out in our licence referred to above.*

*Other than as permitted in any relevant BMJ Author's Self Archiving Policies, I confirm this Work has not been accepted for publication elsewhere, is not being considered for publication elsewhere and does not duplicate material already published. I confirm all authors consent to publication of this Work and authorise the granting of this licence.*

For peer review only

# The barriers and facilitators to help-seeking and recovery among psychologically distressed emergency service staff: A thematic synthesis

Word count: 5364

**Corresponding Author:**
Niklas Auth
University of Birmingham
Tel: 07475545757
Email: nma676@student.bham.ac.uk

Matthew Booker
GP & NIHR Clinical Lecturer in Primary Care (Urgent Care)
Centre for Academic Primary Care (CAPC)
Bristol Medical School
University of Bristol

Jennifer Wild
Associate Professor
Department of Experimental Psychology
Oxford Centre for Anxiety Disorders & Trauma
University of Oxford

Ruth Riley
Lecturer in Applied Health Research
College of Medical and Dental Sciences
University of Birmingham

Abstract

For peer review only

**Objectives**

To identify factors contributing to the mental health recovery of emergency service workers (ESWs) following exposure to occupational trauma, and the barriers and facilitators to mental health help-seeking behaviour among ESWs who identify as being psychologically distressed.

**Background**

ESWs are vulnerable to mental ill health, and reported occupational exposure to trauma as a major contributing factor. This has implications for workforce sustainability. There is uncertainty around which organisational interventions should be provided and how to engage trauma-exposed staff with supportive interventions.

**Design**

Qualitative thematic synthesis.

**Methodology**

Four databases (OVID MEDLINE, EMBASE, PsycINFO and SCOPUS) were systematically searched from January to March 2020. We also used citation tracking and reference chaining. A combination of the Critical Appraisal Skills Programme tool and quality appraisal prompts were used to exclude studies of inadequate quality. Results were synthesised using a thematic technique.

**Results**

Fourteen descriptive themes emerged from this review, categorised into two groups: factors contributing to mental health recovery, such as the need for downtime, peer support and reassurance, and factors influencing help-seeking behaviour, such as stigma, the nature of intervention delivery (mandatory vs non-mandatory, characteristics of intervention provider), and mental health literacy (emotional awareness, education and stigma).

**Conclusion**

For peer review only

Following traumatic exposure, ESWs appear to benefit from recovery time with access to informal support from trusted colleagues. It may be beneficial for organisations to facilitate ESWs' ability to detect and respond appropriately to emotional reactions resulting from traumatic incidents, and to support supervisors and managers in this process. A culture which encourages help-seeking and open dialogue surrounding mental health, and discourages stigma is pivotal to bolster mental health recovery. The potential of certain organisational initiatives in achieving this aim could be further investigated.

**Keywords:** Qualitative research, mental health, emergency responders, thematic synthesis, psychological trauma

## Article Summary- Strengths and limitations of the study

- This is the first review to qualitatively synthesise the barriers and facilitators to help-seeking in the emergency services, and frontline staff's experiences of mental health recovery following traumatic incident exposure.
- Correspondence with key stakeholders in UK ambulance management helped to ensure the aims of this comprehensive qualitative synthesis are relevant to address the challenge faced by mental ill health among emergency service workers.
- This review's implications for practice and research provide emergency service organisations with insight on how to reduce the mental strain experienced by trauma-exposed staff.
- Studies were not excluded on the basis of age or setting location, although the majority of included studies were published within the last 10 years, and were set in socioeconomically developed western countries.
- This review's findings offer implications for existing interventions within UK emergency service organisations.

For peer review only

## Introduction

Emergency service workers (ESWs), also referred to as first responders, consistently experience poorer mental health outcomes when compared to the general population. While subtle differences exist between occupational groups within emergency service organisations (ESOs), ESWs experience disproportionately higher rates of post-traumatic stress disorder (PTSD), anxiety, depression and psychological distress.[1-5] Exposure to traumatic events accounts for higher PTSD rates in the ESW cohort,[2] and is the second greatest cause for poor mental health among UK ESWs, following excessive workload.[6] This review will consider a 'critical incident' as: 'any event with sufficient impact to produce significant emotional reactions in people now or later', as described by Mitchell and Everly.[7] Suicide attempts by ESWs are considerably more prevalent than the estimated rate of 0.5% in the general population.[8] PTSD is a risk factor for suicidal ideation and risky behaviours in civilian and military populations,[9,10] and increases suicidal risk in ESWs.[11] Mental ill health among ESWs is associated with high rates of absenteeism and presenteeism, resulting in significant costs to emergency service organizations.[12]

Trauma Risk Management (TRiM) is a peer support system in use across many UK ESOs, delivered by trained volunteers belonging to the organisation,[13] who psychologically risk assess individuals exposed to traumatic events for psychological risk.[14,15] The evidence surrounding TRiM's impact on users' mental health outcomes or positively altering mental health stigma is inconclusive. [15-18] Schwartz rounds are a 'cultural change initiative'[19] which allow multidisciplinary healthcare staff to share and discuss 'non-clinical aspects (e.g. psychosocial, ethical and emotional issues),[20] and as of May 2020 have been adopted by four UK ambulance trusts.[19] Schwartz rounds are reported to improve staff psychological wellbeing and increase 'empathy and compassion for colleagues', [21] although have not been formally evaluated. Other interventions provided by UK ESOs to support the emotional wellbeing of ESWs after attending to critical incidents include counselling and 24-hour helplines,[22-26] 'defusing' programmes[27] and peer support networks.[28]



For peer review only - http://bmjopen.bmj.com/site/about/guidelines.xhtml

Despite the availability of interventions, ESWs experience barriers to mental health care with one third experiencing mental health stigma, a rate that is higher than the general population.[29] The purpose of this review is to identify the factors associated with mental health recovery of ESWs experiencing distress linked to critical incidents. This review will also identify the barriers to and facilitators of mental health help-seeking behaviour among psychologically distressed ESWs. Our findings and implications will assist UK ambulance trusts in designing national guidelines for psychological support for their staff following traumatic incident exposure by identifying important contextual factors which help and hinder staff when they access support while illuminating benefits and disbenefits of current organisational interventions. This review was initiated in collaboration with key ambulance management stakeholders who expressed a need for research to guide decisions about wellbeing interventions for frontline staff, therefore the focus is on ambulance trusts, although the findings will also be of relevance to fire and police organisations.

## Methods

Methodology

The interpretive review technique method of thematic synthesis, as described by Thomas and Harden, was used to conduct this qualitative review.[30] This inductive synthesis technique is focussed around: intervention need, appropriateness, acceptability and effectiveness,[31] and is an established method in the field of mental health to investigate barriers to help-seeking, and to synthesise qualitative evidence about participant experiences.[29,32,33] It is therefore appropriate given that this review's purpose is to inform UK emergency service organisational guidelines. The research question and final search terms were ratified by a consensus panel of key stakeholders drawn from UK ambulance services. This group contributed to the development and refinement of the review questions, search parameters and application of the review findings. The methods of this review are reported using the ENTREQ framework (Supplementary File 2).

Inclusion criteria

(* indicates further information below)

(1) Study participants were frontline ESWs*, volunteers were not included; (2) There is a focus on work-related psychological distress*;



For peer review only - http://bmjopen.bmj.com/site/about/guidelines.xhtml

(3) Data collection includes primary qualitative interviews, focus groups or observational methods; (4) Analysis focussed around participant attitudes towards: a) behaviour aimed at improving mental health after experiencing a traumatic event OR b) factors which ESWs find helpful or unhelpful for their mental health while experiencing work-related psychological distress, are explored; (5) Published in English and peer-reviewed.

* Emergency service workers' (ESW) constitute of members of the emergency medical services (EMS), the fire service and the police force.[2-4,8] The term 'psychological distress will be referred to according to Ridner's definition (see Appendix D).[34]

For peer review only

Search strategy

Systematic searches of the following six databases were conducted between January and March 2020: OVID MEDLINE, EMBASE, PsycINFO and SCOPUS. The first three databases were searched together using the MEDLINE database. In order to avoid duplicates, the SCOPUS database was then searched with an additional filter to exclude MEDLINE results. One reviewer (NA) then applied the eligibility criteria against the search results, initially screening the full text for obviously irrelevant studies. (Figure 1)

<<insert Figure 1 here>>

*Figure 1: Based on the PRISMA flow diagram template.*[35]

To identify articles missed in the electronic database search, the following methods were also employed: 1) Using 'related article' feature (when available), 2) Searching the titles of included studies in google scholar for citation tracking purposes, 3) Manual searching of the references of relevant studies (reference mining).

Grey literature was searched during background research for context. Two combinations of search criteria (see appendix A) were entered into each database in order to locate relevant literature relating to help-seeking and mental health recovery.

Quality appraisal

One reviewer (NA) independently assessed study quality using the CASP qualitative checklist.[36] Studies scoring less than five were further appraised independently by one of three reviewers (RR, MB, JW) using five prompts recommended by Dixon-Woods et al. to identify

studies of an inadequate quality for inclusion in the review.[37] One study was removed during this approach.[38] In keeping with this approach, 'signal' (the relevance of papers to the review's aims) was prioritised over 'noise' (*the inverse of methodological quality*).[37]

Data extraction

A customised data extraction template (See Appendix B) was created using qualitative data extraction guidance[39] and that of a similar review as a template.[33] In keeping with thematic synthesis approach,[30] first order constructs (direct participant quotations) as well as the author's interpretations were extracted, in separate sections, to allow for a more comprehensive review.[39]

Data synthesis:

Following Thomas and Harden,[30] all data relevant to the research aims were extracted onto templates, which then underwent line-by-line open coding. This approach allows new codes to emerge from the data, rather than imposing a pre-existing framework onto the extracted data. Subsequently, first-level codes were assimilated according to their meanings, similarities and differences. The codes were then arranged in a 'hierarchical tree structure' during which some codes were renamed as new meanings were generated. This process results in 'descriptive themes'. To enhance transparency, primary quotations used to construct the themes are supplied in Appendix C. In the final stage of thematic synthesis, the descriptive themes were used to address the review questions. This process involves 'inferring' the meaning behind the data; The final result of this process was the translation of the descriptive themes into implications for ESO wellbeing policy practice.

## Results

Overview of study characteristics

24 studies were included in this review (see Table 1). All but two of these studies[40,41] employed qualitative methodology only. For these two studies, only data from sections related to their qualitative methodology were extracted. The majority of studies (16 of 24) employed a sample of ambulance personnel[40-55] followed by police officers[56-59] and lastly firefighters.[60,61] The study participants of one study, in addition to ESWs, included participants who did not match the selection criteria[60], and two studies included a mix of different types of ESWs.[62,63] These studies were still eligible as the participant quotations were labelled with an occupational identifier to make it possible to differentiate between eligible and non-eligible participants. The objectives of studies varied widely in studies in terms of



For peer review only - http://bmjopen.bmj.com/site/about/guidelines.xhtml

relevance to this review's aims. Ten of the 24 studies included data relevant to post-traumatic incident mental health recovery,[41-43,45-48,51,55,63] and one study included data relevant to barriers and facilitators to mental health help-seeking.[62] The remaining 13 studies[40,44, 49,50,52-54,56-61] included data which was extracted for both of these outcomes.

**Study quality**:

Study quality varied significantly, with data collection, data analysis and discussion of findings being adequately explained in the majority of studies. All studies provided adequate research aims, although fewer than half sufficiently justified the research design. [41,43,45, 49, 50,55,56, 57,59,61,62,] The lowest scoring domains were recruitment and demonstrating reflexivity. In few studies the researchers adequately justified their selection of participants[41,45,47,57,62,63] or critically examined their relationship with participants. [46,48,52,53,56,62,63] Four studies failed to present any ethical considerations. [40,41,54,60]

The full CASP appraisal table of all included studies is provided in online supplementary file 1. Following CASP appraisal, six studies[38,45,47,51,54,60] initially identified as weaker quality underwent further independent quality appraisal by one of three reviewers (RR, MB, JW), during which process one study was excluded.[38]

This qualitative review identified 14 descriptive themes, which are arranged in groups of higher order themes. In turn, these are grouped into one of the following two thematic domains: 'factors contributing to mental health recovery after traumatic incident exposure' or 'factors influencing mental health help-seeking behaviour'. Table 2 presents a summary of hierarchical thematic structure.

**Study characteristics** *(Table 1)*

<<insert Table 1 here>>

**Summary of Themes** ***(****Table 2)*

<<insert Table 2 here>>

For peer review only

## 1. Factors contributing to mental health recovery

The following themes describe factors which participants attribute to having a direct influence on their mental health recovery after traumatic incident exposure.

**Organisational**

Three themes were identified as being directly linked with systems put in place by the organisations employing ESWs.

_Time-out/Downtime period_

A 'time-out' or 'downtime' period refers to a period of time following a stressful call in which ESWs are temporarily placed off duty, the availability of which was inconsistent across the studies. ESWs working in organisations in which they were offered downtime by their supervisors following certain calls reported that these breaks, ranging from 30 minutes to two hours,[43,46] were essential in order to allow them to 'decompress' in preparation for the remainder of the shift.[43,44] ESWs found particular comfort in casual conversation with their colleagues during this time, which did not necessarily relate to the previous call.[43] During such discussion, humour could be employed by the group as a method of distraction and off-loading.[49] While the majority of ESWs preferred to be in the company of colleagues during this time,[45,49,50,53] some individuals chose to make use of organisation-provided exercise equipment in order to de-stress.[55]

When time-out opportunities were unavailable, ESWs describe rushing into the next call without having psychologically processed the previous call.[41] In such circumstances, paramedics reported difficulty giving their full attention to the next call, limiting their ability to provide life-saving care.[54] Contrary to the above findings, one ambulance worker preferred to be dispatched to another call immediately following a stressful call, due to the distraction this provided.[43]

_Supervisor_

ESWs have supervisors/line managers, whose roles include acting as a point of contact after a traumatic incident. In an ambulance



For peer review only - http://bmjopen.bmj.com/site/about/guidelines.xhtml

setting, paramedics were appreciative of the 'genuine concern' shown by their managers or supervisors following a traumatic call.[42] Concern was commonly expressed by asking paramedics how they were feeling, and providing them with an opportunity to talk.[42,43] Not all ESWs want to be approached by their supervisor immediately following the call, as illustrated by the following quote from a paramedic:

> *'I don't want you to come up and get in my face and say, are you okay? Just leave me alone. Okay. Ask me in a couple of days, am I okay with the call, sort of thing.'* [43]

Occasionally supervisors were responsible for taking an ambulance crew off duty after a call.[43] Even if downtime opportunities were available upon individual request, paramedics described not making use of the opportunity unless suggested or requested by the supervisor.[50] Conversely, a supervisor's influence may also dissuade paramedics from requesting temporary downtime, especially for newer paramedics who were fearful of any repercussions linked to the perceived inability to cope.[50]

Paramedics described unsupportive supervisor responses, such as not recognising the traumatising effect of an incident, applying disciplinary pressure after complicated calls,[41] or showing a lack of concern for paramedics' mental wellbeing.[43,52]

*Official peer support network*

The majority of ambulance nurses taking part in one study were in favour of the existence of peer supporters within their organisation; who the nurses described as being able to 'understand', due to their common experiences.[51] Despite the apparent popularity of the service in this organisation, peer support networks in other organisations were rarely used,[49,54] with defusing occurring 'naturally within the halls' instead.[49] Participants in two studies expressed a hesitancy to make use of peer support opportunities for fear of being judged by colleagues as 'weak'.[51,54] Other concerns centred around the competence of peer supporters, their ability to maintain confidentliality,[51,54] and fear of overwhelming the colleagues delivering the support.[51]

**Informal support**

In contrast to organisational factors, these three themes describe how informal social factors influence ESWs' mental health recovery.



For peer review only - http://bmjopen.bmj.com/site/about/guidelines.xhtml

*Colleagues and family*

ESWs reported that they found it useful to talk with someone in an informal manner. [43,44,45,47,49,50,51,53,54,56,58,60,61]One firefighter described a need to 'vent a backpack', which fills up after each call;[61] suppression could be harmful in the long term as mental health conditions remain unrecognised.[58] The main providers of such informal support were family members and work colleagues, but there were mixed findings in terms of preferences for support.

Many ESWs reported turning to their family members as a primary source of emotional support following difficult calls, [56,43,62,40,46,60,49] who were capable of 'selfless listening' without offering judgement,[56] and with whom ESWs feel more comfortable sharing emotional vulnerability with family members than with colleagues.[43,56] However, a number of ESWs reported avoiding talking to their family members about stressful calls out of a wish to protect them from the trauma they experienced, [46,48,49,56,60–62] although this did not apply to family members with a first responder/healthcare background, who were judged to be able to 'understand' ESWs' traumatic experiences.[47–49] For similar reasons, ESWs were willing to talk to certain colleagues about traumatic calls; The informal sharing of vulnerability was reserved for colleagues with whom ESWs shared a bond of trust[47,48,53,56] and for those more likely to empathise and understand the emotional impact of the event.[40,43,45–48] Sharing experiences with trusted colleagues provided an opportunity for reflection and to hear different interpretations of the event. The risks of disclosure included reliving distressing events, and potential having feelings invalidated when partners felt differently about the event.[48]

*Regular partner*

ESWs described how having a regular work partner helped their ability to process traumatic events encountered on the job.[41,43,53,62]A trusting relationship between partners facilitated comfortable sharing of vulnerability following traumatic calls. [43,53] Having shared the experience, partners could emotionally support colleagues by allowing them to talk about the call and provide reassurance.[41,62] Having a regular partner could however be a negative influence in the case of an unsympathetic relationship, such as partners who respond insensitively to any disclosure of vulnerability.[53] Due to the potential stigma arising from the disclosure of vulnerability within earshot of colleagues, the process of 'defusing' between partners, following a call, commonly takes place within the private space of the ambulance, when returning to base and while awaiting the next call. [53]

*Reassurance and validation*

Reassurance, provided by colleagues indicating that they would have acted in the same way[42,44,48] or by receiving praise for their actions from their supervisor,[43] were valued by ESWs following traumatic incidents, especially those involving fatalities.[43] Reviewing the technical


For peer review only - http://bmjopen.bmj.com/site/about/guidelines.xhtml

aspects of calls with other ESWs provided reassurance that the 'final outcome' was unavoidable.[61] In cases of suicide, learning about the preceding circumstances could provide closure for some ESWs.[43] Following fatal accidents, paramedics also described needing to visit family members in hospital, or to attend funerals.[42]

## 2. Factors influencing help-seeking behaviour

The following themes reflect the barriers and facilitators to help-seeking.

### Nature of intervention delivery

*Mandatory vs non-mandatory*

The decision to employ optional or mandatory organisational mental health support for ESWs following traumatic calls was raised in several of the studies and often depended on the timing of delivery following the incident. Some participants resisted mandatory organisational mental health support following traumatic calls; police officers expressed a need to *'feel in control of the decision to talk'* due to the stigma surrounding any disclosure of vulnerability.[56] Mandatory interventions for ESWs before they are ready to talk could lead to a rejection of the intervention,[43,50] as illustrated by the following paramedic quote:

> *'My emotions are none of your business and if I wanted to share my emotions with you, I'm going to share [them] with someone I trust...'*[50]

Others however believed mandatory interventions would reduce stigma associated with their use,[56,50] and prevent delays to help-seeking due to the stigma associated with disclosing vulnerability.[43] Police officers who were initially reluctant to participate felt 'calmer' and expressed gratitude after attending a mandatory counselling service.[56] EMS staff in one study suggested limiting mandatory support to certain types of incident, such as those involving children.[43]



For peer review only - http://bmjopen.bmj.com/site/about/guidelines.xhtml

*Shared experiences with intervention provider*

Therapists with a background in the emergency services or the military, or trained peers, were preferred by ESWs,[62] because of the belief that they are more likely to 'understand' their problems and experiences.[50] This finding was observed across the emergency services:

> *'Many [participants] also approved of a provider that "knew the job," either working with multiple FRs in the past, or even as a family member.'*[62]

For peer review only

**Stigma as a help-seeking barrier**

*'Macho' culture*

Stigma associated with the disclosure of emotional vulnerability related to traumatic calls, and mental health issues.[40,43,44,49,50,52,53,54,56,58,60] was identified across the emergency services. A 'macho' attitude and culture acted as a key barrier to disclosure where there was an expectation to *'deal with it'*.[53,58] Disclosure of vulnerability in such a culture was perceived as a weakness and responders were viewed as unable to cope with the demands of the job as first responder.[43,49,50,53,56,59,60] Revealing one's feelings was perceived to be emasculating and prevented ESWs from talking about their feelings and seeking support,[50,56,60] as demonstrated by this quote from a police officer:

> 'I *think there's a real element of machismo and masculinity in the police force and it's a bit, sort of a faux pas to admit that things have really affected you ... If I'd have come out and said ''ah you know, that really affected me badly, let's go and sit down and have a cup of tea and talk about it'' I think you're straying into pink and fluffy territory there ... saying 'that made me feel sad' is a bit too far'.*[56]

Stigmatising attitudes held by senior organisational members were influential as they prevented ESWs from contacting their supervisors to seek support.[56,59] Discussing stress in this culture was described as 'taboo'[54] so ESWs reported often avoiding asking their colleagues about their emotions following traumatic calls;[50,52] police officers described how 'tough' colleagues working in such a culture have died by



For peer review only - http://bmjopen.bmj.com/site/about/guidelines.xhtml

suicide.[58] Some organisations described a contrasting culture in which openness about emotional vulnerability was regarded as a strength.[61]

Two studies identified a connection between elements of the 'macho' culture described, and the gender of ESWs. Swedish ambulance nurses reported that organisations where there was a higher proportion of women fostered a culture of openness with respect to sharing vulnerability.[40] Of a small sample of seven paramedics, Clompus et al. noted that female participants were more likely to have made use of formal mental health support mechanisms.[49] The authors attribute this to *'masculinised paramedic culture'*.[49]

*Stigma and shame*

This review identified that the fear of being shamed by being labelled as '*malingerers*'[58] or '*the lazy and the lame*'[59] resulted in presenteeism when officers remained on active duty although mentally unwell. Another aspect of mental health stigma is the belief that affected individuals are less competent in their responsibilities as an ESW, as well as being unreliable.[59] Police officers described a common belief that *'you're on your own'* working a shift with a colleague who has been open about the emotional impact of traumatic calls.[56] ESWs who have been open about their mental health diagnoses describe being labelled as '*mad*'[57] or a '*crazy guy*'[62]; Such attitudes may result in shame and the avoidance of help-seeking.

*Career concerns*

Four studies report that police officers delay help-seeking for mental illness due to concerns about the perceived impact of disclosure on their careers [56-59] Officers believed that being labelled with a mental health condition would obstruct career progression.[57-59] Officers feared being removed from 'public-facing operational roles', and/or feared a reduction in pay which related to being removed from frontline duities.[58] Fear of involuntary dismissal due to disclosure and help-seeking was also reported by study participants from the fire service.[60]

*Confidentiality concerns*

Concerns regarding confidentiality were a barrier for ESWs to formal and informal help-seeking behaviour. Formal support services were


For peer review only - http://bmjopen.bmj.com/site/about/guidelines.xhtml

viewed with suspicion by police officers [57] while other officers felt they might be monitored or labelled as 'weak' if they sought referral to wellbeing services.[56] Concerns about confidentiality were also raised by firefighters, who therefore requested preference for an anonymous counselling service outside of the brigade.[60] In the emergency medical services, EMTs expressed concerns about loss of confidentiality through the organisation-provided peer support network;[54] similar concerns were expressed by EMTs towards critical incident stress debriefing (CISD).[55] There are also perceived risks of confidentiality breaches in informal settings, as described by one paramedic:

> *I think that the stigma is you have to be very careful who you tell that it bothered you or you might get judged as weak or you might get fired.'* [62]

Confidentiality concerns could also indirectly influence help-seeking; ambulance supervisors wishing to put their crew on a time-out after a traumatic call can be dissuaded by knowing this information could become public knowledge among the dispatchers.[43]

**Mental health literacy**

*Emotional awareness*

Participants and authors of the included studies recognised a need for more training and education about mental health related issues for ESWs and family members and supervisors, who may be in a better position to detect behavioural changes and therefore facilitate help-seeking.[42,43,54,62] Such education should focus on increasing ESWs' ability to detect emotional changes within themselves, which could facilitate help-seeking behaviour.[42,54,54,57,62] ESWs expressed a desire to be informed about the types of emotions which could be triggered by work-related traumatic incidents, [42,54] which may reduce shame associated with help-seeking.[62] Studies revealed that an inability to recognise milder mental health symptoms acted as a barrier to help-seeking, with participants writing them off as being 'grumpy',[58] and not recognising and admitting to emotional distress.[42] Additionally, participants were sometimes unaware of the support services available to them,[53,57,62] and were unaware of the benefits of seeking help.[62]



For peer review only - http://bmjopen.bmj.com/site/about/guidelines.xhtml

*Education and Stigma*

While stigma may indirectly change through improving general mental health awareness, authors also emphasised the value of education to reduce organisational stigma.[43] ESWs recommend such education to be delivered regularly in 'brief and efficient' classes of small groups by a peer from outside of the organisation.[62] Having an awareness of work-related mental health problems among colleagues appeared to be an important facilitator of help-seeking.[59,62] Experienced ESWs were regarded as being influential by giving permission to other responders to 'open up'.[61] Police officers in one organisation therefore advocated for mental health 'champions'; colleagues, preferably leaders, who could model vulnerability by openly disclosing their mental illness and work-related distress .[59]

## Discussion

This review synthesised 24 primary studies investigating ESW attitudes to help-seeking and mental health recovery following trauma exposure. The synthesis generated 14 themes relating to 'factors contributing to mental health recovery after traumatic incident exposure' and 'factors influencing mental health help-seeking behaviour'.

Help-seeking: culture/stigma

The findings of this review support quantitative findings that the fear of a breach in confidentiality in attending mental health services, and the concerns about negative career repercussions, pose significant stigma-related barriers to help-seeking for ESWs.[29] This is important as any delays in help-seeking can compound or exacerbate mental ill health.[64,65] Concerns raised by ESW responders about the impact of mental health disclosure on career progression, professional identity and competence have also been found in military personnel.[29,33,66,67]

Masculinised social norms, similar to those identified by this review as existing in ESOs, have been described in the military,[15,67] which is male dominated with high rates of mental health stigma.[29] There are, however, many other similarities between the two settings which are likely to be influential, such as 'norms and values that place a premium on self-reliance in the face of obstacles'.[29] Occupations dominated by hyper-masculine stereotypes may also disadvantage men who do not identify with these values, therefore discouraging the very demographic who would challenge them.[68] Recruiting a more diverse, emotionally literate and aware workforce, with more women may challenge macho work cultures which prevent ESWs from disclosing their vulnerability.[68]



For peer review only - http://bmjopen.bmj.com/site/about/guidelines.xhtml

This review's finding of anticipated stigma acting as a help-seeking barrier, is supported by the findings from a systematic review which found that *'stigma can potentially lead to delayed presentation in mental health care'* for ESWs,[29] and as well as negatively influencing help-seeking in civilian populations.[66] These findings have also been demonstrated in a qualitative review of a military setting.[33]

The findings of this review in terms of attitudes to mental ill health are consistent with Goffman who argued that stigma is defined in and enacted through socially constructed 'norms'. The 'norms' of what is stigmatising and what isn't are socially constructed within a large variety of contexts and has the potential to 'shift'.[69] An effective way of challenging stigma associated with mental illness and changing negative perceptions is through a strategy of increasing contact .[15,70-72] In order to effectively counter stigmatising attitudes towards mental health, contact should be delivered in the context of the following five factors:[72] equal status, the opportunity for individuals to get to know each other, information which challenges negative stereotypes, active co-operation and pursuit of a mutual goal. Such approaches and factors could be employed to tackle the stigma and culture which prevent ESWs from seeking help. Elements of these factors can be recognised in organisational strategies such as Mental Health Champions and Schwartz rounds (see below).

**Help-seeking: Education**

This review identifies a demand for improving the mental health literacy of ESWs and describes the type of education ESWs believe would be appropriate. Mental health education has been shown to be effective at changing attitudes towards mental health disorders when aimed at large populations, smaller at-risk groups or at the individual level.[73,74] Anti-stigma education was introduced in UK emergency services as part of the 'Blue Light Programme',[6] and demonstrated that achieving anti-stigma change at the employee level requires sustained education efforts over a number of years.[6]

The literature suggests that certain types of events, such as paediatric fatalities, events involving multiple casualties or suicides, have a higher traumatising potential than others.[40,60,75] The evidence suggests however that the process of traumatisation among ESWs is highly individual, relating to 'personal history, situation, and perspective' of the exposed individual,[55] and ESWs can experience trauma in different ways depending on how they contextualise the victim. [40] Questionnaires taking into account individual factors, such as the ESW's 'state of mind' preceding the event, are therefore a useful resource for predicting peritraumatic distress in exposed individuals.[76]


For peer review only - http://bmjopen.bmj.com/site/about/guidelines.xhtml

**Mental health recovery: Social support and downtime**

Review participants identified the importance of social support following exposure to traumatic incidents, in consistence with the wider literature. [17,33,77,79] Downtime was positively valued by ESWs who had experienced it, yet is not commonly granted to ESWs in practice.[79,80] The types of mental health outcome affected by post-incident downtime and extent of its influence is disputed. Carlier et al. identified 'insufficient time allowed by the employer to resolve the trauma' was correlated with higher PTSD scores in police officers three months post-trauma, but not at 12 months.[81] Having insufficient recovery time following traumatic calls has been correlated with higher emotional exhaustion[80] and psychological distress.[82]These results are in contrast with the findings of a cross-sectional quantitative survey of the psychological consequences of downtime in 217 ambulance workers, which revealed increasing periods of downtime, up to and including one day, to be significantly associated with lower depression scores, but not with symptoms of 'post-traumatic stress, burnout and stress-related physical symptoms'.[83]

**Application of review findings to organisational interventions, policy and research**

Our findings have implications for three organisational interventions which are in use or available for use within the emergency service organisations. These interventions have particular relevance in the context of this review due to their shared potential to alter organisational mental health stigma, which this review identified as a barrier to help-seeking.

1) *Trauma risk management (TRiM)*

Consistent with qualitative findings of navy personnel regarding the implementation of TRiM, [84] this review identified a perception among ESWs that peer-support programs are relevant and suitable for their needs, as well as concerns regarding confidentiality and competence of practitioners of peer support programs. Peer supporters are not however regarded as possessing the same professional competence/credibility of a professional mental health professional. Of note, while review participants expressed concerns about being judged for being perceived as 'weak' by peers in a peer-support system, such a concern was not detected among naval officers towards TRiM,[84] despite widespread stigma towards help-seeking and mental health issues which exists in the military.[33]



For peer review only - http://bmjopen.bmj.com/site/about/guidelines.xhtml
For peer review only

2) *Mental Health Champions*

This review identified that awareness of colleagues' mental health challenges could be an important facilitator for help-seeking. The 'Blue light champions' role[85] therefore appears to be a potentially effective method of improving attitudes, although quantitative evaluation linking these roles with ESW mental health outcomes and culture change is lacking. Staff satisfaction surveys of these roles reveal a lack of support from management and not having sufficient time available to dedicate to the role.[86]

3) *Schwartz Rounds*

In terms of challenging organisational culture which prohibits help-seeking due to stigma, Schwartz Rounds offer staff the opportunity to disclose their vulnerability whilst fostering a connectedness to other staff. However, Schwartz Rounds have not been evaluated and it is unclear whether they reduce stigma, facilitate help-seeking behaviour, or alter mental health outcomes. Quantitative evidence describing the effectiveness of Schwartz on the above outcomes would be useful.

## Implications for policy

Following traumatic calls, ESWs will likely benefit from having a period of mental recovery time during which they can access informal support from their colleagues. ESOs should be aware of the therapeutic effects of informal support in this post-incident setting, and facilitate its availability. It should be considered that despite recognising its value, ESWs are unlikely to request downtime themselves, instead relying on it being imposed/offered by supervisors. It may also be useful for supervisors to consider that the manner in which they approach ESWs for welfare concerns, as demonstrated by this review, in addition to their overall attitude regarding mental health, have significant implications for the help-seeking behaviours of ESWs. It may be important to consider that ESWs report trusting professional relationships, such as regular work-partners, as being psychologically protective against occupational trauma experiences, providing vulnerability can be comfortably shared. ESWs may benefit from education enhancing their ability to recognise pathological emotional changes secondary to traumatic



For peer review only - http://bmjopen.bmj.com/site/about/guidelines.xhtml

incident exposure both in themselves and colleagues, and to able to appreciate when it is appropriate to access formal organisational resources and the potential benefit of different types of interventions for distressed ESWs. Additionally, communicating to ESWs that the objective characteristics of an event cannot invalidate their experience of being traumatised, could enhance help-seeking by normalising symptoms of mental ill health. ESOs may wish to consider that mental health champion-type roles, providing staff in such roles are adequately supported, are regarded by ESWs as valuable tools for challenging mental health stigma. This review's findings may provide insights into how engagement between ESWs and official peer support networks can be improved, by focussing on cultural anti-stigma change and by targeting specific barriers to engagement as identified in the results section. Quick fix solutions to the cultural change necessary to increase help-seeking in ESOs should not be prioritised over meaningful sustained efforts to improve mental health literacy.

## Conclusion

The barriers and facilitators to help-seeking identified by this review may assist emergency medical organisations in improving engagement amongst their staff with organisational wellbeing interventions. Findings related to the experiences and attitudes of staff regarding mental health recovery should be considered when implementing occupational support programmes.

For peer review only

Footnotes

**Acknowledgements:** This work was initially written as a dissertation. We would like to thank Justin Waring for being a valued source of support throughout the dissertation. Special thanks to Anna Parry for her vital contributions in the early stages of the project, and involving key ambulance stakeholders during the process of developing the project aims. We would also like to thank Marcus Auth for his assistance provided in the form of advice regarding structure of the paper, and formulating the abstract.

**Contributors:** NA is the main author involved in all stages of the design, analysis and drafting and writing of the paper. RR was involved in the conception of the project's scope, design, and interpretation of results, providing important supervision and mentorship throughout. RR, MB and JW assisted with drafting the article and revising it critically for important intellectual content, providing methodological



For peer review only - http://bmjopen.bmj.com/site/about/guidelines.xhtml

advice, quality appraisal by independently appraising lower CASP scoring studies, and final approval of the version to be published. All authors have approved the final version of the paper ahead of publication.

**Funding:** This research received no specific grant from any funding agency in the public, commercial or not-for-profit sectors.

**Competing interests:** None declared.

**Provenance and peer review:** Not commissioned; externally peer reviewed.

**Data sharing statement:** No additional data are available.

For peer review only

## References

1. Jones S. Describing the Mental Health Profile of First Responders: A Systematic Review. J Am Psychiatr Nurses Assoc 2017 May;23(3):200-214.

2. Lima Ed P, Assuncao AA. Prevalence and factors associated with Posttraumatic Stress Disorder (PTSD) in emergency workers: a systematic literature review. Rev bras epidemiol 2011 Jun;14(2):217-230.



For peer review only - http://bmjopen.bmj.com/site/about/guidelines.xhtml

3. Berger W, Coutinho E, Figueira I, Marques-Portella C, Luz M, Neylan T, et al. Rescuers at risk: a systematic review and meta-regression analysis of the worldwide current prevalence and correlates of PTSD in rescue workers. Soc Psychiatry Psychiatr Epidemiol 1206;47(6):1001-1011

4. Stanley IH, Hom MA, Joiner TE. A systematic review of suicidal thoughts and behaviors among police officers, firefighters, EMTs, and paramedics. Clin Psychol Rev 1603;44:25-44.

5. Petrie K, Milligan-Saville J, Gayed A, Deady M, Phelps A, Dell L, et al. Prevalence of PTSD and common mental disorders amongst ambulance personnel: a systematic review and meta-analysis. Soc Psychiatry Psychiatr Epidemiol 2018 Sep;53(9):897-909.

6. Mind. Wellbeing and mental health support in the emergency services. [internet]. 2019. [cited 2019 November 5]. Available from: https://www.mind.org.uk/media-a/4572/20046_mind-blue-light-programme-legacy-report-v12_online.pdf

7. Mitchell, J.T., & Everly, G.S. (1995). Critical Incident Stress debriefings: An operations manual for the prevention of traumatic stress among emergency service and disaster workers (2nd ed. revised). Ellicott City, MD: Chevron Publishing Company.

8. Substance Abuse and Mental Health Services Administration. First Responders: Behavioral Health Concerns, Emergency Response, and Trauma. [internet]. 2018 May. [cited 2019 Oct 14]. Available from: https://www.samhsa.gov/sites/default/files/dtac/supplementalresearchbulletin-firstresponders-may2018.pdf

9. Jakupcak, M., Cook, J., Imel, Z., Fontana, A., Rosenheck, R., & McFall, M. Posttraumatic stress disorder as a risk factor for suicidal ideation in Iraq and Afghanistan War veterans. Journal Of Traumatic Stress 2009 July;22(4), 303-306. doi: 10.1002/jts.20423

10. Tarrier N, Gregg L. Suicide risk in civilian PTSD patients. Social Psychiatry and Psychiatric Epidemiology. 2004;39(8).

11. Maia DB, Marmar CR, Metzler T, Nóbrega A, Berger W, Mendlowicz MV, et al. Post-traumatic stress symptoms in an elite unit of Brazilian police officers: Prevalence and impact on psychosocial functioning and on physical and mental health. J Affect Disord 2007;97(1):241-245.

12. Mind. The case for support. [internet]. 2017. [cited 2019 November 6]. Available from: https://www.mind.org.uk/media-a/4510/blp-blueprint_case-for-support.pdf

13. Whybrow D, Jones N, Greenberg N. Promoting organizational well-being: a comprehensive review of Trauma Risk Management. Occup Med (Lond) 2015 /06/01;65(4):331-336.

14. Hunt E, Jones N, Hastings V, Greenberg N. TRiM: an organizational response to traumatic events in Cumbria Constabulary. Occup Med (Lond) 2013 /12/01;63(8):549-555.

15. Gould M, Greenberg N, Hetherton J. Stigma and the military: Evaluation of a PTSD psychoeducational program. Journal of Traumatic Stress 2007;20(4):505-515.

16. Jones N, Burdett H, Green K, Greenberg N. Trauma Risk Management (TRiM): Promoting Help Seeking for Mental Health Problems Among Combat-Exposed U.K. Military Personnel. *Psychiatry*. 2017;80(3):236-251. doi:10.1080/00332747.2017.1286894




For peer review only - http://bmjopen.bmj.com/site/about/guidelines.xhtml

17. Shakespeare-Finch J, Rees A, Armstrong D. Social Support, Self-efficacy, Trauma and Well-Being in Emergency Medical Dispatchers. Soc Indic Res 1509;123(2):549-565.

18. Greenberg N. A cluster randomized controlled trial to determine the efficacy of Trauma Risk Management (TRiM) in a military population. J Trauma Stress ;23(4):430-437.

19. The Point of Care Foundation. List of Schwartz Rounds sites. [internet] [cited 2020 May 19]. Available from: https://www.pointofcarefoundation.org.uk/our-work/schwartz-rounds/list-of-sites/

20. Taylor C, Xyrichis A, Leamy M, Reynolds E, Maben J. Can Schwartz Center Rounds support healthcare staff with emotional challenges at work, and how do they compare with other interventions aimed at providing similar support? A systematic review and scoping reviews. BMJ Open. 2018;8(10):e024254.

21. Maben J, Taylor C, Dawson J, Leamy M, Mccarthy I, Reynolds E, et al. A realist informed mixed-methods evaluation of Schwartz Center Rounds® in England. Health Services and Delivery Research 1101;6(37).

22. HMICFRS. Fire and rescue- Staffordshire. [internet] 2019 [cited 2020 April 5]. Available from: https://www.justiceinspectorates.gov.uk/hmicfrs/frs-assessment/frs-2018/staffordshire/people/

23. HMICFRS. Fire and rescue- Buckinghamshire. [internet] 2019 [cited 2020 April 5]. Available from: https://www.justiceinspectorates.gov.uk/hmicfrs/frs-assessment/frs-2018/buckinghamshire/

24. HMICFRS. Fire and rescue- Derbyshire. [internet] 2019 [cited 2020 April 5]. Available from: https://www.justiceinspectorates.gov.uk/hmicfrs/frs-assessment/frs-2018/derbyshire/

25. HMICFRS. Police- Dfyed-Powes. [internet] 2019 [cited 2020 April 5]. Available from: https://www.justiceinspectorates.gov.uk/hmicfrs/peel-assessments/peel-2018/dyfed-powys/legitimacy/detailed-findings/#question-3

26. HMICFRS. Police- Cumbria. [internet] 2019 [cited 2020 April 5]. Available from: https://www.justiceinspectorates.gov.uk/hmicfrs/peel-assessments/peel-2018/cumbria/legitimacy/detailed-findings/#question-1

27. Her Majesty's Inspectorate of Constabulary and Fire & Rescue Services (HMICFRS). Fire and rescue- Gloucestershire. [internet] 2019 [cited 2020 April 5]. Available from: https://www.justiceinspectorates.gov.uk/hmicfrs/frs-assessment/frs-2018/gloucestershire/people/

28. UNISON. Linc Peer Support. [internet] 2016 [cited 2020 April 20]. Available from: https://lasunison.com/linc-peer-support

29. Haugen PT, McCrillis AM, Smid GE, Nijdam MJ. Mental health stigma and barriers to mental health care for first responders: A systematic review and meta-analysis. Journal of Psychiatric Research. 2017(10):125-126.

30. Thomas J. Methods for the thematic synthesis of qualitative research in systematic reviews. BMC Medical Research Methodology ;8:45-46.

31. Barnett-Page E, Thomas J. Methods for the synthesis of qualitative research: a critical review. BMC Medical Research Methodology. 2009;9(1).

32. Thomas J, Sutcliffe K, Harden A, Oakley A, Oliver S, Rees R, Brunton G, Kavanagh J (2003) Children and Healthy Eating: A systematic review of barriers and facilitators. London: EPPI-Centre, Social Science Research Unit, Institute of Education,




For peer review only - http://bmjopen.bmj.com/site/about/guidelines.xhtml

University of London.

33. Coleman SJ, Stevelink SAM, Hatch SL, Denny JA, Greenberg N. Stigma-related barriers and facilitators to help seeking for mental health issues in the armed forces: a systematic review and thematic synthesis of qualitative literature. 1708;47(11):1880-1892.

34. Ridner SH. Psychological distress: concept analysis. J Adv Nurs. 2004 ;45(5):536-546.

35. Moher D, Liberati A, Tetzlaff J, Altman DG, The PRISMA Group (2009). Preferred Reporting Items for Systematic Reviews and MetaAnalyses: The PRISMA Statement. PLoS Med 6(7): e1000097. doi:10.1371/journal.pmed1000097

36. CASP. Qualitative checklist. [internet] 2018 [cited 2020 March 5]. Available from: https://casp-uk.net/wp-content/uploads/2018/03/CASP-Qualitative-Checklist-2018_fillable_form.pdf

37. Dixon-Woods M, Cavers D, Agarwal S, Annandale E, Arthur A, Harvey J, et al. Conducting a critical interpretive review of the literature on access to healthcare by vulnerable groups. 2006.

38. Sommerfeld A, Wagner S, Harder H, Schmidt G. Behavioral Health and Firefighters: An Intervention and Interviews with Canadian Firefighters. Journal of Loss and Trauma. 2017;22(4):307-324.

39. Noyes J & Lewin S. Chapter 5: Extracting qualitative evidence. In: Noyes J, Booth A, Hannes K, Harden A, Harris J, Lewin S, Lockwood C (editors), Supplementary Guidance for Inclusion of Qualitative Research in Cochrane Systematic Reviews of Interventions. Version 1 (updated August 2011). Cochrane Collaboration Qualitative Methods Group, 2011. Available from URL http://cqrmg.cochrane.org/supplemental-handbook-guidance .

40. Regehr C, Goldberg G, Hughes J. Exposure to human tragedy, empathy, and trauma in ambulance paramedics. American Journal of Orthopsychiatry. 2002;72(4):505-513.

41. Regehr C, Millar D. Situation critical: High demand, low control, and low support in paramedic organizations. Traumatology. 2007;13(1):49-58.

42. Halpern J, Gurevich M, Schwartz B, Brazeau P. What makes an incident critical for ambulance workers? Emotional outcomes and implications for intervention. Work & Stress. 2009;23(2):173-189.

43. Halpern J, Gurevich M, Schwartz B, Brazeau P. Interventions for critical incident stress in emergency medical services: a qualitative study. Stress and Health. 2009;25(2):139-149.

44. Jonsson A, Segesten K. The meaning of traumatic events as described by nurses in ambulance service. Accident and Emergency Nursing. 2003;11(3):141-152.

45. Jonsson A, Segesten K. Guilt, shame and need for a container: a study of post-traumatic stress among ambulance personnel. Accident and Emergency Nursing. 2004;12(4):215-223.

46. Jeruzal J, Boland L, Frazer M, Kamrud J, Myers R, Lick C et al. Emergency Medical Services Provider Perspectives on Pediatric Calls: A Qualitative Study. Prehospital Emergency Care. 2019;23(4):501-509.

47. Fernández-Aedo I, Pérez-Urdiales I, Unanue-Arza S, García-Azpiazu Z, Ballesteros-Peña S. A qualitative study about experiences and emotions of emergency medical technicians and out-of-hospital emergency nurses after performing cardiopulmonary resuscitation resulting in death. Enfermería Intensiva (English ed). 2017;28(2):57-63.

48. Svensson A, Fridlund B. Experiences of and actions towards worries among ambulance nurses in their professional life: A critical incident study. International Emergency Nursing. 2008;16(1):35-42.



For peer review only - http://bmjopen.bmj.com/site/about/guidelines.xhtml

49. Clompus S, Albarran J. Exploring the nature of resilience in paramedic practice: A psycho-social study. International Emergency Nursing. 2016;28:1-7.

50. Douglas L, Cheskes S, Feldman M, Ratnapalan S. Paramedics' experiences with death notification: a qualitative study. Journal of Paramedic Practice. 2012;4(9):533-539.

51. Carvello M, Zanotti F, Rubbi I, Bacchetti S, Artioli G, Bonacaro A. Peer-support: a coping strategy for nurses working at the Emergency Ambulance Service. *Acta Biomed*. 2019;90(11-S):29-37

52. Oliveira A, Neto F, Teixeira F, Maia Â. Working in prehospital emergency contexts. International Journal of Workplace Health Management. 2019;12(6):469-482.

53. Drewitz-Chesney C. Exploring paramedic communication and emotional expression in the workplace after responding to emergency calls. Australasian Journal of Paramedicine. 2019;16.

54. Gallagher S, McGilloway S. Living in critical times: The impact of critical incidents on frontline ambulance personnel--a qualitative perspective. *Int J Emerg Ment Health*. 2007;9(3):215-223.

55. Folwell A, Kauer T. 'You see a baby die and you're not fine:' a case study of stress and coping strategies in volunteer emergency medical technicians. Journal of Applied Communication Research. 2018;46(6):723-743.

56. Evans R, Pistrang N, Billings J. Police officers' experiences of supportive and unsupportive social interactions following traumatic incidents. European Journal of Psychotraumatology. 2013;4(1):19696.

57. Strydom H, Botha K, Boshoff P. AN ASSESSMENT OF THE NEED OF POLICE OFFICIALS FOR TRAUMA INTERVENTION PROGRAMMES – A QUALITATIVE APPROACH. Social Work/Maatskaplike Werk. 2015;51(2).

58. Edwards A, Kotera Y. Mental Health in the UK Police Force: a Qualitative Investigation into the Stigma with Mental Illness. International Journal of Mental Health and Addiction. 2020;.

59. Bullock K, Garland J. Police officers, mental (ill-)health and spoiled identity. Criminology & Criminal Justice. 2017;18(2):173-189.

60. Haslam C, Mallon K. A preliminary investigation of post-traumatic stress symptoms among firefighters. Work & Stress. 2003;17(3):277-285.

61. Abelsson A. First response emergency care – experiences described by firefighters. International Journal of Emergency Services. 2019;8(3):247-258.

62. Jones S, Agud K, McSweeney J. Barriers and Facilitators to Seeking Mental Health Care Among First Responders: "Removing the Darkness". Journal of the American Psychiatric Nurses Association. 2019;26(1):43-54.

63. Hasselqvist-Ax I, Nordberg P, Svensson L, Hollenberg J, Joelsson-Alm E. Experiences among firefighters and police officers of responding to out-of-hospital cardiac arrest in a dual dispatch programme in Sweden: an interview study. BMJ Open. 2019;9(11):e030895.

64. Fridgen G, Aston J, Gschwandtner U, Pflueger M, Zimmermann R, Studerus E, et al. Help-seeking and pathways to care in the early stages of psychosis. Soc Psychiatry Psychiatr Epidemiol 1307;48(7):1033-1043.

65. Gronholm PC. Mental health-related stigma and pathways to care for people at risk of psychotic disorders or experiencing first-episode psychosis: a systematic review. Psychol Med ;47(11):1867-1880.



For peer review only - http://bmjopen.bmj.com/site/about/guidelines.xhtml

66. Clement S, Schauman O, Graham T, Maggioni F, Evans-Lacko S, Bezborodovs N, et al. What is the impact of mental health-related stigma on help-seeking? A systematic review of quantitative and qualitative studies. Psychol Med 2015 101 0;45(1):11-27.

67. Sharp M, Fear NT, Rona RJ, Wessely S, Greenberg N, Jones N, et al. Stigma as a Barrier to Seeking Health Care Among Military Personnel With Mental Health Problems. Epidemiol Rev 2015 /01/01;37(1):144-162.

68. Peters K, Ryan M, Haslam S. Marines, medics, and machismo: Lack of fit with masculine occupational stereotypes discourages men's participation. British Journal of Psychology. 2014;106(4):635-655.

69. E. Goffman. Stigma: Notes on the management of spoiled identity. Prentice-Hall, Englewood Cliffs, NJ (1963)

70. Dickstein BD, Vogt DS, Handa S, Litz BT. Targeting Self-Stigma in Returning Military Personnel and Veterans: A Review of Intervention Strategies. Military Psychology 2010 April 2,;22(2):224-236.

71. Norms, Committee on the Science of Changing Behavioral Health Social, Board on Behavioral C, Education, Division of Behavioral and Social Sciences and, National Academies of Sciences, Engineering. Approaches to Reducing Stigma. : National Academies Press (US); 2016.

72. Like Minds. THE POWER OF CONTACT- Project to Counter Stigma and Discrimination Associated with Mental Illness. [internet] 2005 [cited 2020 April 6]. Available from: https://www.likeminds.org.nz/assets/National-Plans/1power-of-contact.pdf

73. Jorm AF, Barney LJ, Christensen H, Highet NJ, Kelly CM, Kitchener BA. Research on Mental Health Literacy: What we know and what we Still Need to know. Aust N Z J Psychiatry 2006 January 1,;40(1):3-5.

74. Jorm AF. Mental Health Literacy: Empowering the Community to Take Action for Better Mental Health. Am Psychol ;67(3):231-244.

75. Jacobsson A, Backteman-Erlanson S, Brulin C, Hörnsten Å. Experiences of critical incidents among female and male firefighters. International Emergency Nursing. 2015;23(2):100-104.

76. Halpern J, Maunder R, Schwartz B, Gurevich M. The critical incident inventory: characteristics of incidents which affect emergency medical technicians and paramedics. BMC Emergency Medicine 101 0;12:10.

77. Brewin CR, Andrews B, Valentine JD. Meta-Analysis of Risk Factors for Posttraumatic Stress Disorder in Trauma-Exposed Adults. J Consult Clin Psychol 10 00;68(5):748-766.

78. Ploeg Evd, Kleber RJ. Acute and chronic job stressors among ambulance personnel: predictors of health symptoms. Occupational and Environmental Medicine 2003 /06/01;60(suppl 1):i40-i46.

79. Gouweloos-Trines J, Tyler MP, Giummarra MJ, Kassam-Adams N, Landolt MA, Kleber RJ, et al. Perceived support at work after critical incidents and its relation to psychological distress: a survey among prehospital providers. Emerg Med J 1221;34(12):816.

80. Alexander D, Klein S. Ambulance personnel and critical incidents: impact of accident and emergency work on mental health and emotional well-being. British Journal of Psychiatry 101 0(178):76-81.

81. CARLIER I, LAMBERTS R, GERSONS B. Risk Factors for Posttraumatic Stress Symptomatology in Police Officers: A Prospective Analysis. The Journal of Nervous &amp Mental Disease. 1997;185(8):498-506.

For peer review only

82. Mitchell AM. Critical incident stress debriefing: implications for best practice. Disaster Management & Response 2003;1(2):46-52.

83. Halpern J. Downtime after critical incidents in emergency medical technicians/paramedics. BioMed Research International ;2014:483140-483141.

84. Greenberg N, Langston V, Iversen AC, Wessely S. The acceptability of 'Trauma Risk Management' within the UK Armed Forces. Occup Med (Lond) 2011 /05/01;61(3):184-189.

85. Mind. Mind Blue light champions. [internet] 2019 [cited 2020 Feb 19]. Available from: https://www.mind.org.uk/bluelightchampions

86. Mind. Blue Light Programme Strand 1 (Part 2). [internet] 2016 [cited 2020 May 5]. Available from: https://www.mind.org.uk/media-a/4864/strand-1-part-2.pdf

For peer review only

## Appendix A: Literature search terms

(((mental AND (health OR well?being)) OR (trauma* OR *stress OR recover* OR PTSD OR post?traumatic?stress OR emotion* OR (critical AND incident) OR (traumatic AND incident) OR (traumatic AND event) OR experience* OR support* OR *support) AND (emergency service* OR emergency medical service* OR EMS OR first responder* OR ambulanc* OR paramedic* OR firefighter* OR fire service* fire department* OR police*) AND (qual* OR mixed?method* OR interview* OR focus?group*) NOT (quality OR emergency?department)

((emergency service* OR emergency medical service* OR EMS OR first responder* OR ambulanc* OR paramedic* OR firefighter* OR fire service* fire department* OR police*) AND (qual* OR mixed?method* OR interview* OR focus?group*) NOT (quality OR emergency?department)) AND ((help-seeking* OR stigma* OR mental* OR barriers OR (MeSH terms: help-seeking behaviour, social stigma, mental health, psychiatry, social support, mental disorders))


For peer review only - http://bmjopen.bmj.com/site/about/guidelines.xhtml

Appendix B: Data extraction template

Citation

Reviewer

Country

Aims

Ethics – how ethical issues were addressed

Study setting– e.g. type of organisation

Relevant context to study setting

Socio-demographics of the country / region

Recruitment context (e.g. where people were recruited from)

Sampling– what sampling methods, what were inclusion and exclusion criteria,

Data quality rating

Participants– 'population described'

Participants– 'characteristics'– age, sex etc



For peer review only - http://bmjopen.bmj.com/site/about/guidelines.xhtml

Theoretical background

Proportion of sample exposed to critical incidents/ definition of critical incident/ anything relevant to the study matching my selection criteria

Definition of critical incidents/ something related

Data collection methods- data collection methods, role of researcher within setting...

Data analysis approach- how many researchers, how did they code, how were findings corroborated,

Themes identified in study (1st order interpretations)- Help-seeking

Themes identified in study (1st order interpretations) – Mental Health recovery

Data extracts related to key themes- Help-seeking

Data extracts related to key themes- Mental health recovery

Author explanation/interpretation of key themes (2nd order interpretations)- Help-seeking

Author explanation/interpretation of key themes (2nd order interpretations)- Mental health recovery

Recommendations made by authors (both outcomes; but specify)

Third order interpretations:

For peer review only

Other potentially relevant information

## Appendix C: Example literature quotations used to construct themes

| | | | |
|---|---|---|---|
| **Factors contributing to mental health recovery post traumatic incident exposure** | **Organisational** | **Time out/Downtime** | *They prefer support to be offered immediately after the call and find that downtime after a stressful call allows them to decompress and prepare for the rest of their shift: '...we knew we weren't going to get a call right, so we knew we had the two hours, so we watched a funny show and had a nap...But...like for me personally... that's exactly...what I needed.' Participant 4 from focus group 4.* Douglas et al. 2013<br><br>*. . . our supervisor took us out of service for a couple of hours and let us go have lunch, sat down and had lunch and just kind of relax and talk amongst ourselves, not even about the call, just about whatever, just to kind of relax. Before we went back on shift. Certainly we could have booked off the rest of the day, you know, on stress leave or whatever, but we all, found that just having, just being able to have a couple of hours to, kind of, you know, relax a little bit, that helps us a lot.' (Focus group #520–522)*<br>Halpern et al. 2008<br><br>*I like didn't want to be like I need to take a couple of hours off because I did not want to look bad in front of the supervisor.-Participant 2 from focus group 2.*<br>Douglas et al. 2013<br><br>*Unfortunately, paramedics often do not have adequate time to talk. Several participants expressed their frustration over 'calls waiting'. These calls are waiting to be dispatched as no crew is available. When BCEHS paramedics offload their patient(s) at the hospital, dispatch can immediately send them to a waiting call. This can limit paramedics' ability to discuss CIs. James desired:*<br><br>*...to be given the time uninterrupted, unbothered... Give us our time to decompress.*<br>Drewitz-Chesney et al. 2019 |
| | | **Supervisor** | *Depending on who it is . . . one supervisor, we took, we did a [critical call], and we took the last hour of the shift off. And he was making us fill out all these forms and, you know, telling us that if we wanted to take the next shift off that we had to go see our doctor and get a note for this and that. And you know, just made it more stress-(Focus group #520–522)*<br>Halpern et al. 2008<br>*'And so our supervisor was really, we have a great supervisor and he's, you know, asked us all specifically, "are you guys okay", you know. And the other crew went off on stress for the rest of the shift. We stayed, because we said, y ou know, we're okay. We just kind of dealt with the aftermath of everything. It was still a pretty stressful call but at least we had that option. And he had no problems, like, he said, go home. Whatever you guys need. So and that's a big thing.- (Focus group #520– 522)*<br>Halpern et al. 2008<br><br>*Supervisors, however, were perceived differently. Some paramedics reported feeling unsupported when their supervisors questioned why they were reacting to an event that they did not perceive as traumatic:*<br>*I wasn't really involved in a traumatic event because there was no loss of life, or you know what I mean, um, it, there wasn't really much sympathy the, um, I could, not that I really, um, oh, this wasn't, um, a huge accident, you weren't injured, you know, really why are you complaining?*<br>Regehr et al. 2007<br><br>*he's [supervisor] quite an old fashioned sort of police officer, not the bloke you would sort of want to go in and have a chat with about a sudden death you'd just been to ... If I went in and said 'Governor, can I have a chat about the sudden death?', he'd look at me as if I'd just asked to kill one of his children!*<br>Evans et al. 2013 |

For peer review only

| | | | |
|---|---|---|---|
| | | | |
| | | **Peer support network** | *Nurse 1 "Therefore, talking to a person who has the same skills, abilities and knowledge you have would probably be more meaningful".*<br>Carvello et al. 2019<br><br>*The majority of nurses is in favour of peer-supporters. The motivation is based on the fact that they recognise the peer-support as someone that can understand what nurses really mean when relating a traumatic event, being one of their colleagues.* Carvello et al. 2019 |
| | **Informal support** | **Colleagues and family** | *The more you talk about something, the more it becomes something you've told and your telling becomes part of the memory, as opposed to it being a really shiny, vivid thing inside your head*those images.*<br>Evans et al. 2013<br><br>*I don't think talking about it to people at work is the release, the escape I need ... it's speaking to people who I care about and who care for me and just having that comfort zone, that's what's important to me. [P16]*<br>Evans et al. 2013<br><br>*... I never tell my wife that, I would never tell her that because I just think that would have really put the frighteners on her. [P15]*<br>Evans et al. 2013 |
| | | **Regular partner** | *For me it's just always been that partner, because they're right there with you and they'll know what's going on, and you really want somebody that can understand what's happening.*<br>*Jessica et al. 2009*<br><br>*...for those of us that have regular partners, regular stations, rely on your partner... They're gonna know if something's up with you.*<br>Drewitz-Chesney et al. 2019<br><br>*Paramedics working with different partners may be unable to recognise changes as readily as they would in a regular partner. When paramedics are without a regular partner or are uncomfortable speaking with their current partner, they often don't discuss calls. Instead, some participants said they speak with long-time paramedic friends or coworkers with whom they have similarities, ie. age or training level.*<br>Drewitz-Chesney et al. 2019<br><br>*...if I'm working with my regular partner, those communication lines are very, very open and we can talk about the tough calls, how we're feeling, if there's anything we would have done different [sic] (Sean).*<br>Drewitz-Chesney et al. 2019<br><br>*I really envy the paramedics that have regular partners that they know, and they trust, and they can talk to (Krista).*<br>Drewitz-Chesney et al. 2019<br><br>*...I would have a regular partner and that partner would be someone who is just like family to me. We would just talk about everything, without even hesitating...'*<br>Drewitz-Chesney et al. 2019 |
| | | **Reassurance and validation** | *I remember going to the hospital and I remember I was disappointed because the parents weren't there at that time...And I don't know what it was. It was just something that made me feel like I just needed to talk to them. But I never ended up talking to them. So anyway that call definitely stands out as being...(Stuck with you.) Yeah.*<br>Halpern et al. 2009 |

For peer review only

| | | | |
|---|---|---|---|
| | | | *And it's weird, I looked in the newspaper. I saw his funeral announcement and I went to the funeral. (Oh okay.) I didn't talk to any of the family. I just went for my own, I don't know why ... I, for some reason, I guess I felt I needed to follow up and so I did. And I sent the family a condolence card and then a friend of mine ... met a friend of the family's friend and he had said, oh, his [relative] wants to talk to you ... So she called me and she wanted to know, like, you know, what happened with her [relative] and who was the last one to talk to him ... It's kind of funny because there are so many calls you do and you never think about them again. But this one, I thought about him a lot. I guess because he was so young and again, I really didn't think he was going to die. So I thought, okay, well, you know, he's at the trauma centre now and I had no idea that he was that badly injured. And then he died. So I think that freaked me out. I wasn't prepared for that. And I thought, oh my God, he's so young and his family wasn't there. I felt badly his family wasn't there and then. So that one stuck with me for a while.*<br><br>*Interviewer: "And talking to his [relative], did that help or ...?"*<br><br>*It more, I felt it was helping them so it made me feel better, because she really, really was upset. And I felt she wanted, I think she felt a lot better after talking to me and hearing something about what happened. And so it made me feel better to give them some kind of closure.Pg 181-182*<br>Halpern et al. 2009<br><br>*. . . the day after that call, my supervisor kind of sensed that I wanted to talk to him and I don't know why, you know, he came to the hospital. But it was the next day and he was kind of like, so pull up a chair, sit down. Let's just talk. And it was like I wanted somebody just to say to me, okay, this is your time and you can talk right now and I'm not going to judge you. I'm not going to talk about anything. I'm going to let you just have your 10 minutes. And that made a huge difference. I have the utmost respect for my particular supervisor . . . Whether it was talking specifi cally about the call or just having a little bit of a "hey you're okay at your job", type comment. (Participant #122)*<br>Halpern et al. 2008 |
| **Factors influencing help-seeking behaviour** | **Nature of intervention delivery** | **Mandatory vs non-mandatory** | *We had counselling every six months ... and everybody used to go 'Oh I've got to see the counsellor this week', but I tell you what ... we all quite enjoyed it ... I was so much calmer after speaking to her but it's something I'd never have done had I not been made to do it. [P12]*<br>Evans et al. 2013<br><br>*My emotions are none of your business and if I wanted to share my emotions with you, I'm going to share [them] with someone I trust... Participant 1 from focus group 1.*<br>Douglas et al. 2013 |
| | | **Shared experiences with intervention provider** | *FRs in our study preferred a MH professional with experience as an FR or military veteran. Many also approved of a provider that "knew the job," either working with multiple FRs in the past, or even as a family member.*<br>Jones et al. 2020<br><br>*I've been to [therapy] a couple of times. . . . The guy that I got was excellent, but I only think it was excellent because he was prior military (P8, Firefighter ×22 years).*<br>Jones et al. 2020 (study 4)<br>*...someone I think understands what's going on, and has been through what I've done.- Participant 3 from focus group 4.*<br>Douglas et al. 2013 |

| | | | |
|---|---|---|---|
| | **Stigma as a help-seekingbarrier Specific barriers** | **'Macho culture'** | *I think there's a real element of machismo and masculinity in the police force and it's a bit, sort of a faux pas to admit that things have really affected you ... If I'd have come out and said 'ah you know, that really affected me badly, let's go and sit down and have a cup of tea and talk about it' I think you're straying into pink and fluffy territory there ... saying 'that made me feel sad' is a bit too far. [P3]*<br>Evans et al. 2013<br><br>*Everyone wants to be tough and strong. Maybe that was my downfall or problem at the time and I didn't want to admit that I needed any kind of help. I guess I didn't want to be perceived as weak. (Participant #110)*<br>Halpern et al. 2008<br><br>*... that's so ridiculous. You need to harden up if you want to do this job, and old people die.*<br>Drewitz-Chesney et al. 2019<br><br>*... in metro, there's a lot more...bravado and joking around about things...If you can get one on one with someone, they're usually a lot more receptive and a lot more empathetic- (Dennis).*<br>Drewitz-Chesney et al. 2019<br><br>*Unfortunately there's a massive stigma [...] one of my sergeants the other week, there was mental health training coming up and his reaction to being put forward to go on the training was, 'Well what do I want to go and learn how to deal with a load of nutcases for?*<br>Bullock et al. 2018<br><br>*We are many women at the station so its easier for us to talk about what happened...You have to talk about what happened otherwise you can't go on. It's easier than on a station with only men, it's not necessary with any "macho style" so I think it's easier for the men (at our station) to talk about things you must talk about. (A female nurse describes her feelings.)*<br>Jonsson et al. 2003 |
| | | **Stigma and shame** | As an institution, they were very very good, in getting me better, at making me understand, but back at work that was a different ball game. You've got your colleagues who are still at work, still running around like idiots and they're like, oh fucking hell, you've had three months off, you know, I should have gone off with stress. (Participant 1).<br>Edwards et al. 2020<br><br>*There have been officers that are doing the shift that have shown that they can't deal with situations like that, and been very open about it*and they haven't got the respect from the shift, because the colleagues go 'well, you're on your own if you're working with her, because she'd back away' or whatever. So you don't want to be considered as one of those. [P7]*<br>Evans et al. 2013 |
| | | **Career concerns** | *I think that the stigma is you have to be very careful who you tell that it bothered you or you might get judged as weak or you might get fired" (P2, EMT/paramedic ×20 years).*<br>Jones et al. 2020<br><br>If you've a form of mental health illness you will not get on; you will not be promoted, ...people will not want you on their section. (Participant 4).<br>Edwards et al. 2020<br><br>I feel scared to declare anything or do anything about anything because will it bite me later on in life? Will it prevent me from doing something in the police later on? Could it be used against me? ... will it be used in a negative way later on?<br>Bullock et al. 2018 |


For peer review only - http://bmjopen.bmj.com/site/about/guidelines.xhtml

| | | | |
|---|---|---|---|
| | | **Confidentiality concerns** | *I know our department's very, very poor at keeping secrets. So if I put a crew out of service, I have to tell the communications center. Communications says, we're sitting this far apart, Hey X, I just put the 22 car out of service in stress. Every dispatcher in there hears it. Every call receiver in there hears it. So they hear it. I don't know if they say anything. I don't know if they go home and tell all their friends and family. But I don't like that system. There's no quiet way of doing it. (Participant #128)*<br>Halpern et al. 2008<br><br>*I think that the stigma is you have to be very careful who you tell that it bothered you or you might get judged as weak or you might get fired" (P2, EMT/paramedic ×20 years).*<br>Jones et al. 2020<br><br>*.. .to whom must I speak in the police if I can't trust anyone. So now I'm seeking professional help outside the police .*<br>Boshoff et al. 2015 |
| | **Mental health literacy** | **Emotional awareness** | *Some appreciated that difficulties in recognizing and admitting to distress pose significant barriers to accessing support. Recognizing the emotional impact of critical incidents may help to address these barriers.*<br>Halpern et al. 2009<br><br>*I didn't recognise it as what it was; I just thought I was grumpy...you don't see them creeping up, and in the end, the thing that tips you over the edge, the thing that makes your bottle overflow if you like can be something quite small because you've got used to dealing with stuff. (Participant 5).*<br>Edwards et al. 2020 |
| | | **Education and stigma** | *I think for, for everybody, is, here's my thought towards your process, is giving them the tools. People are going to be very tough and say, yeah, yeah, fine. But you know if you could somehow identify the emotions that go along with these calls that might be starting to put you on tilt...Then you can teach people to be aware of them and say, hey, you know what, it's okay to say, I need to talk to someone.*<br>Halpern et al. 2009<br><br>*In particular, it would appear from this study that teaching ambulance personnel about the emotional aspects surrounding different types of critical incident may diminish their confusion about which incidents they can expect to impact them.*<br>Halpern et al. 2009<br><br>*I guess that's the main two [barriers], pride and then denial. . . . But, it's just education. Just letting [FRs] know, look, these things are normal. It's going to happen to somebody" (P11, Firefighter ×8.5 years).*<br>Jones et al. 2020<br><br>*Knowing that other people are there dealing with that same stuff. You can bounce ideas off each other, see what's worked in their situations and what hasn't . . . you realize you're not crazy (P12, EMT/paramedic ×14 years).*<br>Jones et al. 2020<br><br>*If you could actually get people in in front of officers saying, 'I was one of those people that didn't believe stress could ever get to that level and it was ridiculous and you just needed to work harder', maybe officers would accept that from another officer more than just somebody standing in front of you training because it's getting officers to accept that actually it's okay, you're only human and your body and your mind can only take so much, and maybe they'd accept it more then.*<br>Bullock et al. 2018 |



For peer review only - http://bmjopen.bmj.com/site/about/guidelines.xhtml

Appendix D– Abbreviations and definitions

| Abbreviation | Meaning |
|---|---|
| NHS | National Health Service |
| PTSD | Post-Traumatic Stress Disorder |
| ESW | Emergency Service Worker |
| ESO | Emergency Service Organisation |
| EMS | Emergency Medical Service |
| CISD | Critical Incident Stress Debriefing |
| UK | United Kindgdom |
| TRiM | Trauma Risk Management |
| GMB Union | General, Municipal, Boilermakers and Allied Trade Union |
| RTA | Reciprocal Translocation Analysis |
| CASP | Critical Appraisal Skills Programme |
| EMT | Emergency Medical Technician |

| Term | Definition |
|---|---|
| Psychological distress | 'the unique discomforting, emotional state experienced by an individual in response to a specific stressor or demand that results in harm, either temporary or permanent, to the person'[7] |
| Critical incident | 'any event with sufficient impact to produce significant emotional reactions in people now or later'.[9] |



For peer review only - http://bmjopen.bmj.com/site/about/guidelines.xhtml

| | |
|---|---|
| | |

| Type of stigma | Definition |
|---|---|
| Anticipated stigma (Perceived stigma) | *'the extent to which people believe they personally will be viewed or treated in a stigmatizing way if their mental health problem or related help-seeking becomes known'* *Sharp M, Fear NT, Rona RJ, Wessely S, Greenberg N, Jones N, et al. Stigma as a Barrier to Seeking Health Care Among Military Personnel With Mental Health Problems. Epidemiol Rev 2015 /01/01;37(1):144-162.* |
| Treatment stigma | 'the stigma associated with seeking or receiving treatment for mental ill health' *Clement S, Schauman O, Graham T, Maggioni F, Evans-Lacko S, Bezborodovs N, et al. What is the impact of mental health-related stigma on help-seeking? A systematic review of quantitative and qualitative studies. Psychological Medicine 2015 /01;45(1):11-27.* |
| Internalized stigma | 'holding stigmatizing views about oneself' *Clement S, Schauman O, Graham T, Maggioni F, Evans-Lacko S, Bezborodovs N, et al. What is the impact of mental health-related stigma on help-seeking? A systematic review of quantitative and qualitative studies. Psychological Medicine 2015 /01;45(1):11-27.* |
| Public stigma | 'invalidating and unjustified beliefs (i.e., prejudices and endorsed stereotypes) about others' *Dickstein BD, Vogt DS, Handa S, Litz BT. Targeting Self-Stigma in Returning Military Personnel and Veterans: A Review of Intervention Strategies. Military Psychology 2010 April 2,;22(2):224-236.* |

For peer review only

## Figures, tables and supplementary files for manuscript

Figure 1: Preferred Reporting Items for Systematic Reviews and Meta-Analyses flowchart describing the process of study selection.

Table 1: Table of study characteristics

Table 2: Summary of themes

Online Supplementary File 1: CASP appraisal table

For peer review only

**Figure 1**:

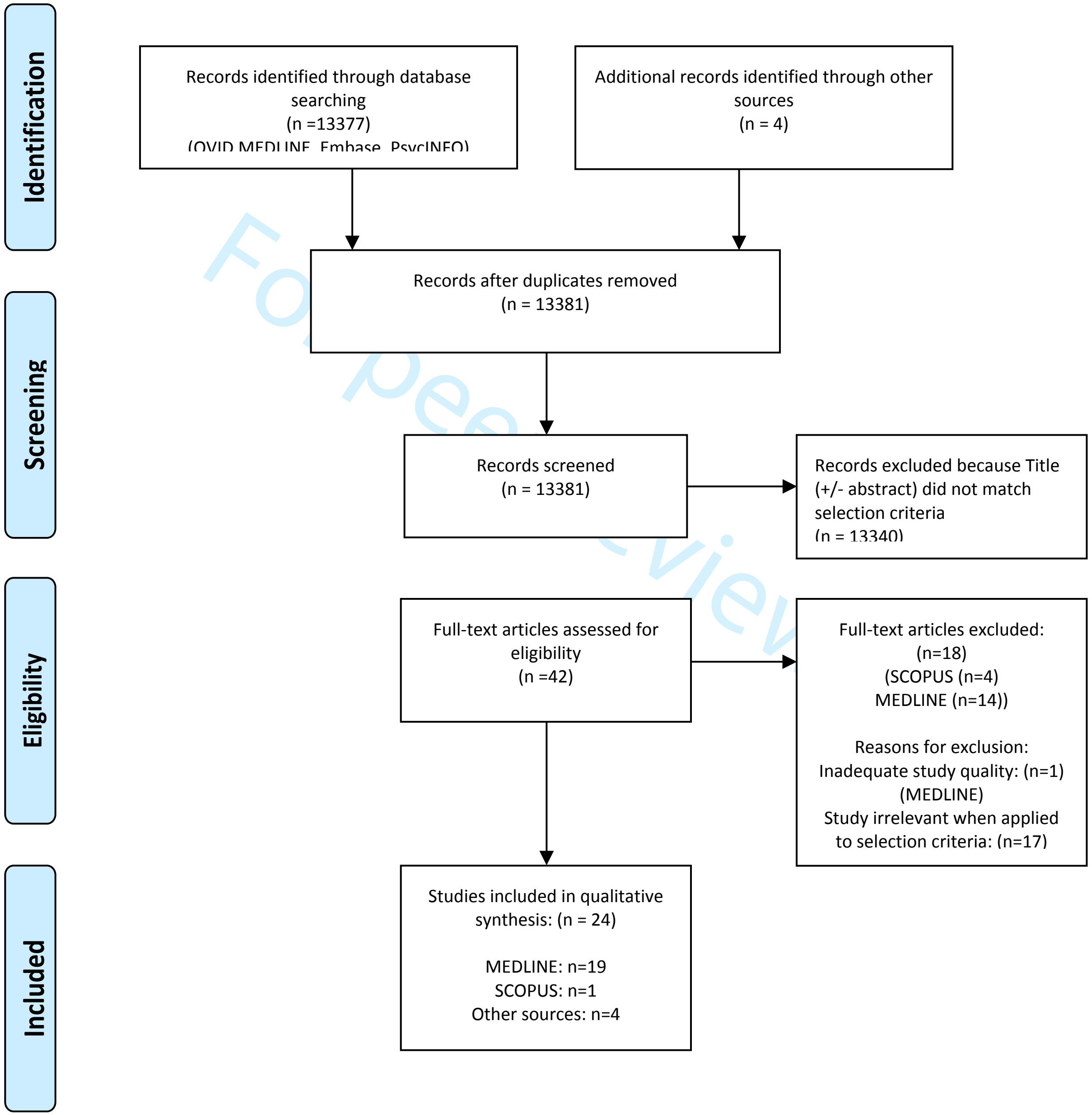
Identification
Screening
Eligibility
Included
Records identified through database searching (n =13377) (OVID MEDLINE, Embase, PsycINFO)
Additional records identified through other sources (n = 4)
Records after duplicates removed (n = 13381)
Records screened (n = 13381)
Records excluded because Title (+/- abstract) did not match selection criteria (n = 13340)
Full-text articles assessed for eligibility (n =42)
Full-text articles excluded: (n=18) (SCOPUS (n=4) MEDLINE (n=14))
Reasons for exclusion: Inadequate study quality: (n=1) (MEDLINE) Study irrelevant when applied to selection criteria: (n=17)
Studies included in qualitative synthesis: (n = 24)
MEDLINE: n=19
SCOPUS: n=1
Other sources: n=4

**Table 1:**

| Title of study (Authors, year) | Quality ranking against CASP criteria | Participants | Study aim(s) | Country | Method of data collection | Analysis method |
|---|---|---|---|---|---|---|
| What makes an incident critical for ambulance workers? Emotional outcomes and implications for intervention. Halpern et al. 2009[93] | 5 | N=60 4 supervisors, 54 front-line ambulance workers | To characterize critical incidents as well as elicit suggestions for interventions | Canada | Focus groups/ individual interviews | Ethnographic content analysis |
| Police officers' experiences of supportive and unsupportive social interactions following | 6 | N=19 Police officers | (1) What are police officers' experiences of supportive and unsupportive interactions following potentially traumatic incidents? | England | Semi-structured interviews | Thematic analysis |

| | | | | | | |
|---|---|---|---|---|---|---|
| traumatic incidents.<br>Evans et al. 2013[94] | | | (2) Do interactions differ on the basis of the context and source of support (i.e., at work with colleagues and supervisors, or outside of work with family and friends)?<br>(3) How do supportive/unsupportive interactions facilitate/hinder the processing of traumatic incidents? | | | |
| Interventions for critical incident stress in emergency medical services: a qualitative study<br>Halpern et al. 2008[95] | 6 | N=60<br>4 supervisors, 54 front-line ambulance workers | To explore and describe Emergency Medical Technicians' (EMTs) experiences of critical incidents and views about potential interventions, in order to facilitate development of interventions that take into account EMS culture | Canada | Focus groups/ individual interviews | Ethnographic content analysis |
| Barriers and Facilitators to Seeking Mental Health Care Among First | 9 | N=32<br>Twenty-five (78%) of the participants were active | To explore factors that influenced FRs' perceptions of mental health problems and | United states | Ethnographic individual interviews | Content analysis |

For peer review only

| | | | | | | |
|---|---|---|---|---|---|---|
| Responders: "Removing the Darkness" Jones et al, 2020[96] | | firefighters, 15 (47%) were certified EMTs, and 11 (34.4%) were certified EMTs/ paramedics. | engagement in MH services. | | | |
| Exposure to human tragedy, empathy, and trauma in ambulance paramedics. Regehr et al, 2002[97] | 6 | N=18 Paramedics | This mixed-methods study attempts to better understand factors that lead to higher levels of distress among paramedics within the theoretical framework of emotional and cognitive empathy. | Canada | Semi-structured interviews | Thematic analysis |
| The meaning of traumatic events as described by nurse sin ambulance service Jonsson et al. 2003[98] | 5 | N=362 240 EMTs, 122 registered nurses | The aim of this phenomenological study is to uncover the essence of traumatic events experienced by Swedish ambulance personnel. | Sweden | Written reports | Phenomenologic al analysis |
| Guilt, shame and need for a container: a study of post-traumatic stress among ambulance personnel | 4 | N=10 Ambulance nurses and ambulance technicians | The phenomenon approached in this study could be described as 'the way ambulance staff experience and handle traumatic events'. | Sweden | Individual interviews | Descriptive phenomenology |

For peer review only

| | | | | | | |
|---|---|---|---|---|---|---|
| Jonsson et al. 2004[99] | | | | | | |
| Situation Critical: High Demand, Low Control, and Low Support in Paramedic Organizations Regehr et al. 2007[100] | 7 | N=17 Paramedics | This mixed-methods study involving survey design and qualitative interviews seeks further to understand the factors related to these high levels of occupational stress. | Canada | Long interviews | Constant comparative method |
| Emergency Medical Services Provider Perspectives on Pediatric Calls: A Qualitative Study Jessica et al. 2019[101] | 8 | N=17 Paramedics and EMTs | This qualitative study was conducted to increase understanding about the difficulties of responding to pediatric calls and to obtain information about how organizations can better support EMS providers in managing potentially difficult calls. | United states | Focus groups | Directed content analysis |
| An assessment of the need of police officials for trauma intervention programmes - a qualitative approach. | 5 | N=40 Police officials | To conduct a qualitative situational analysis by exploring the experience and specific needs with regards to trauma and trauma intervention of police officials within the North- | South Africa | Focus groups | Thematic analysis |

For peer review only

| | | | | | | |
|---|---|---|---|---|---|---|
| Boshoff et al. 2015[102] | | | West Province's specialist units. | | | |
| A preliminary investigation of post-traumatic stress symptoms among firefighters Haslam et al. 2003[103] | 4 | N=31 11 firefighters, 8 station officers, 4 sub officers, 4 leading firefighters, 2 fire control officers, 2 area divisional officers | This preliminary study aimed to conduct an in-depth investigation of symptoms cited by fire service personnel and assess potential risk factors for mental health and PTSD. | England | Individual interviews | Thematic analysis |
| A qualitative study about experiences and emotions of emergency medical technicians and out-of-hospital emergency nurses after performing cardiopulmonary resuscitation resulting in death Fernández-Aedo et al. 2017[104] | 4 | N=13 7EMTs, 6 ambulance nurses | To explore the experiences, emotions and coping skills among emergency medical technicians and emergency nurses after performing out-of-hospital cardiopulmonary resuscitation maneuvers resulting in death. | Spain | Semi-structured individual interviews and focus groups | Thematic analysis |
| Experiences of and actions towards worries among ambulance | 7 | N=25 Ambulance nurses | The purpose of this study was to describe critical incidents in which ambulance nurses experience | Sweden | Semi-structured individual interviews | Structural analysis/Critical incident technique |

For peer review only

| | | | | | | |
|---|---|---|---|---|---|---|
| nurses in their professional life: A critical incident study Svensson et al. 2008[105] | | | worry in their professional life and the actions they take in order to prevent and cope with it. | | | |
| Exploring the nature of resilience in paramedic practice: A psycho-social study Clompus et al. 2016[106] | 5 | N=7 Paramedics or EMTs | The aim of this study was to explore the question of how paramedics 'survive' their work within the current healthcare climate. | England | Biographical narrative interviews and semi-structured interview with all participants | Thematic analysis |
| First response emergency care – experiences described by firefighters Abelsson et al. 2019[107] | 5 | N=35 Firefighters | The purpose of this paper is to describe firefighters' experiences of first response emergency care | Sweden | Group interviews | Interpretive qualitative content analysis |
| Paramedics' experiences with death notification: a qualitative study Douglas et al. 2013[108] | 5 | N=28 Paramedics | To explore paramedics' experiences and coping strategies with death notification in the field. | Canada | Focus groups | Inductive thematic analysis |
| Peer-support: a coping strategy for nurses working at the Emergency | 4 | N=14 Ambulance nurses | The aim of the study is to explore the experiences, the opinions and feelings of emergency medical | Italy | Semi-structured interviews | Not made explicit |

For peer review only

| | | | | | | |
|---|---|---|---|---|---|---|
| Ambulance Service Carvello et al. 2019[109] | | | service nursing staff in relation to the use of the peer supporting model. | | | |
| Experiences among firefighters and police officers of responding to out-of hospital cardiac arrest in a dual dispatch programme in Sweden: an interview study Hasselqvist-Ax et al. 2019[110] | 8 | N=22 10 police officers, 12 firefighters | *The aim of this interview study was to explore firefighters' and police officers' experiences of saving lives in OHCA in a dual dispatch programme.* | Sweden | Individual interviews | Critical incident technique |
| Working in prehospital emergency contexts: Stress, coping and support from the perspective of ambulance personnel Oliveira et al. 2019[111] | 8 | N=14 Ambulance personnel | The purpose of this paper is to explore, from this group perspective, sources of stress, coping strategies and support measures | Portugal | Semi-structured interviews | Thematic analysis |
| Exploring paramedic communication and emotional expression in the workplace after | 7 | N=8 Paramedics | The study aim was to learn about peer communication and emotional expression between paramedics in the workplace, | Canada | Semi-structured individual interviews | Constructivist grounded theory |

For peer review only

| | | | | | | |
|---|---|---|---|---|---|---|
| responding to emergency calls Drewitz-Chesney et al. 2019[112] | | | after they respond to calls. | | | |
| Mental Health in the UK Police Force: a Qualitative Investigation into the Stigma with Mental Illness Edwards et al. 2020[113] | 6 | N=5 Police officers | The study aims to explore institutional negativity and stigma in the police force towards mental ill health | United Kingdom | Semi-structured individual interviews | Thematic analysis |
| Living in Critical Times: The Impact of Critical Incidents on Frontline Ambulance Personnel: A Qualitative Perspective Gallagher et al. 2007[114] | 2 | N=27 21 EMTs, 6 EMCs | The principal aim of this second stage of the study was to ascertain, using qualitative methods, the impact of CIs on frontline staff by allowing them to tell their own stories. | Ireland | Individual interviews | Thematic analysis |
| *Police officers, mental (ill-) health and spoiled identity* Bullock et al. 2018[115] | 5 | N=59 52 police officers, 2 police staff, 4 PCSOs, 1 special constable | The processes through which some police officers with mental ill-health experience stigmatization. | England and Wales | Phone interviews | Thematic analysis |

For peer review only

| | | | | | | |
|---|---|---|---|---|---|---|
| 'You see a baby die and you're not fine:' a case study of stress and coping strategies in volunteer emergency medical technicians Folwell et al. 2018[116] | 6 | N=25 EMTs | This study explores the lived experience of ESP involved in unsuccessful pediatric resuscitation efforts and how this experience affects them professionally and personally. | United States | Individual interviews | Constant comparative analysis |

**Table 2:**

| **Domain** | **Higher level theme** | **Descriptive themes** |
|---|---|---|
| Factors contributing to mental health recovery | Organisational | -Time out/Downtime<br>-Supervisor<br>-Official peer support network |
| | Informal support | -Colleagues and family<br>-Regular partner<br>-Reassurance and validation |
| Factors influencing help-seeking behaviour | Nature of intervention delivery | -Mandatory vs non-mandatory<br>-Shared experiences with intervention provider |

For peer review only

| | | |
|---|---|---|
| | Stigma as a help-seeking barrier | -Macho culture<br>-Stigma and shame<br>-Career concerns<br>-Confidentiality concerns |
| | Mental health literacy | -Emotional awareness<br>-Education and stigma |

For peer review only

## Online Supplementary File 1

| Study title (Authors, year) | Aims & Methods | Research design | Sampling | Data collection | Reflexivity | Ethical issues | Data analysis | Discussion of findings | Value |
|---|---|---|---|---|---|---|---|---|---|
| *What makes an incident critical for ambulance workers? Emotional outcomes and implications for intervention.* Halpern et al. 2009[42] | Research aim clearly stated, introduction uses relevant literature to successfully explain demand for the research. Qualitative methods are appropriate for investigating complicated emotions, which could not be explored in equal depth via quantitative methods. | Interviews and focus groups seem appropriate methods to address the research aims, but the authors don't specify the reasons for their choice to use both methods. | 4 supervisors, 54 front-line ambulance workers. Researchers state that participants were recruited from a specific cohort at a mandatory training conference, but no detail is given about what information was provided to the participants to entice them to enter the study. The authors considered representation of all job levels and both genders during recruitment. | The setting of the data collection away from the workplace was stated but no justification was given for the choice of study setting or methods used. Detail was given about the length of interviews and focus groups, and the main topics of questions asked during interviews and focus groups were stated. The researchers stated they had reached saturation by the end of the study. Data obtained was in the form of audio recordings, written transcripts and written notes of non-verbal communication information as observed by the researchers. | Researchers do not acknowledge how their characteristics may have impacted on the results. The researchers acknowledge that self-selection or reporting bias may have contributed to the discrepancy in findings between men and women. | Ethical approval was obtained from a research ethics board, and participants signed a consent form. No further information was given regarding any ethical safeguards in place. Lack of description of how confidentiality was maintained. | The authors described how ethnographic content analysis was used to generate thematic codes, with a constant comparative method throughout. Three authors were involved in the coding process, although only two of these authors listened to the audiotapes. Initial and final codes are described, and contradictory data are taken into account. Sufficient data are presented to support the findings. The authors describe how textual data was not analysed for focus groups. | Findings are explicitly stated and are relevant to the researchers' aims. The only measure of credibility described was the use of three analysts during the coding process. Findings are discussed with reference to wider literature, and implications for interventions for critical incident stress are made. | The authors identify promising post-incident interventions which they believe should be further researched. Wider applicability of the study findings appear to not extend beyond the ambulance cohort. The authors provide implications for interventions for critical incident stress. |

| | | | | | | | | | |
|---|---|---|---|---|---|---|---|---|---|
| *Police officers' experiences of supportive and unsupportive social interactions following traumatic incidents.* Evans et al. 2013[56] | Research aims made clear in objectives section. Overall aim 'was to understand the types of support processes that might promote resilience'. Methods are appropriate as experiences, and other subjective outcomes are best measured qualitatively. | Semi-structured interview design appears reasonable to explore the proposed aims, but choice of qualitative study design not directly justified. | 19 Police officers. A snowballing approach was used to recruit officers, which allowed detection of information-rich participants. By limiting participants to those who have served over 2 years, the chance of traumatic incident exposure was increased. Limiting participants to those without diagnosed PTSD was appropriate as the study measured resilience-promoting investigations. Non-participation was not discussed. | An interview schedule was used for the semi-structured interviews, although only the broad focus of the schedule was provided by the authors. Interviews were audio-recorded and transcribed verbatim. No information was given regarding interview setting, and data saturation was not discussed. No justification for choice of methods. | Researchers acknowledge how their role as researchers could have affected the data, giving social desirability bias as an example. | A National Health Service ethics committee granted ethical approval, and participants provided informed consent. No information is provided regarding efforts to maintain confidentiality. | The process of thematic analysis was described in detail. All themes generated can be linked with relevant first order data. Contradictory data is taken into account and present within the themes. Three authors were involved in the analytic process to minimise bias. One author had the main role in the analysis, while two others cross-referenced certain parts of their work in order to increase credibility. All 3 researchers reached a consensus on unclear issues before the final themes were decided upon. | Themes are clearly summarised, with relevant quotations to support each theme. Findings are discussed in relation to the research aims. Three researchers were involved in the analytic process. | The researchers provide detailed recommendations for further quantitative research. Furthermore, the researchers recommend similar qualitative research in a population that has a history of PTSD, so that comparisons can be made. |
| *Interventions for critical incident stress in emergency medical services: a qualitative study* Halpern et al. 2008[43] | Clear statement of study aims, with overall study purpose ' to facilitate development of interventions that take into account EMS culture.' Examining experiences adequately requires qualitative methodology. | Explanations were provided for how the focus groups and semi-structured interviews were conducted. E.g flexible interview structure ' to permit the elaboration of more in-depth or emotionally significant data' and focus group size of 4-8 members 'to maximize interactive data'. No justification given for choice of interviews and focus groups. | 4 supervisors, 54 front-line ambulance workers. Participants were sampled to ensure all job levels and genders were represented. Researchers state that participants were recruited from a specific cohort at a mandatory training conference, but no detail is given about what information was provided to the participants to entice them to enter the study. The sampling process was iterative-preliminary analysis informed subsequent sampling decisions. No further participants were recruited once saturation was reached. | The setting of the data collection away from the workplace was stated but not explained. Detail was given about the length of interviews and focus groups, and the main topics of questions asked during interviews and focus groups were stated. The researchers stated they had reached saturation by the end of the study. Data obtained was in the form of audio recordings, written transcripts and written notes of non-verbal communication information as observed by the researchers. | Researchers do not critically examine their role, potential bias and influence of research question formulation or data collection. | Ethical approval was obtained from a research ethics board, and participants signed a consent form. No further information was given regarding any ethical safeguards in place. | The authors described how ethnographic content analysis was used to generate thematic codes, using a constant comparative method to categorise themes. Initial and final codes are described. The authors explain why textual data was not analysed for focus groups. Member validation was carried out 6 months post initial data collection. Contradictory data are presented and discussed, and sufficient data are presented to support the findings. | The findings are clearly presented, using relevant participant quotations to support the authors interpretations. 3 analysts were used during the data analysis process. Member validation was carried out 6 months after data was gathered. Contradictory viewpoints are taken into account and discussed. | The research conclusions appear relevant to all EMS organisations which operate in the format of using supervisors. The authors provide suggestions for interventions which are of relevance to EMS organisations. The authors recommend that the interventions of:<br>1. Supervisor support<br>2. Timeout period post-incident<br><br>should be further researched. |
| *Barriers and Facilitators to Seeking Mental Health Care Among First Responders: "Removing the Darkness"* Jones et al, 2020[62] | Aims are clearly stated and the study's importance justified. The authors aims include exploring organisational culture and perceptions of individuals, for which qualitative research is appropriate. | The decision to use individual ethnographic interviews the qualitative method of choice is well explained. The authors also explain why they chose a 'community-based approach'. | 32 firefighters and/or EMTs/paramedics.<br><br>This was a convenience sample.<br>The principal investigator facilitated recruitment by developing relationships with community partners | Ethnographic ndividual interviews were conducted with participants by the principal investigator. An interview guide was used and attached, which was developed by the PI based on the study aim and input from | The principal investigator acknowledged how being married to a firefighter/paramedic promoted buy in from the community during the recruitment stage. | Ethical board approval was gained. 'Prior to starting the interview, the PI reviewed the study information sheet and completed the consent process with each participant. ' Resources for | Content analysis and constant comparison methods were used during the analytic process. Themes which emerged during data analysis were checked with participants in later interviews. Two researchers independently | Findings are presented as themes, with participant quotations included for each theme to support the author's interpretations. Credibility was enhanced by the use of more than one analyst, and member validation. Findings are | The authors make implications for practice, identifying 'need for improving education and awareness regarding duty-related MH problems.' The authors also make implications for nursing practice, and implications |

For peer review only

| | | | | | | | | | |
|---|---|---|---|---|---|---|---|---|---|
| | | | who each recruited participants from their own organisations. They did this by distributing e-mails on behalf of the principal investigator. Reasons for non-participation: 'individuals not answering or returning calls (all were called twice), scheduling conflicts' | a qualitative methods expert, along with feedback from community partners to strengthen trustworthiness and validity. Interviews were audio recorded and transcribed. Field notes were also conducted by the authors. Data collection setting was decided on the basis of convenience for participants. Data saturation was reached. | | further emotional support were provided to participants in case of emotional distress experienced during the study. | analysed the transcripts, and discussed the process until consensus was reached. (on 80% of top level codes). Exploration of new themes was stopped when saturation was reached. participant quotations are provided throughout to support the author's interpretations. The researchers acknowledge a bias risk due to the principal investigator conducting all the interviews. Sufficient data are presented to support the findings, and contradictory data are reported (such as positive and negative experiences of therapists). | discussed in relation to the original research question. | for future research. |
| *Exposure to human tragedy, empathy, and trauma in ambulance paramedics.* Regehr et al, 2002[40] | Aims of study are clearly stated, and their importance justified. Qualitative methods are appropriate because the authors want to explore experiences in depth. | Choice of qualitative study design not explicitly justified. | 18 paramedics. 'Purposive sampling was used to ensure that participants represented a wide range of experiences in terms of length of time with the service and types of events encountered.' Non participation was not discussed. | A semistructured interview guide was used to conduct interview, and the authors provided examples of guide topics. These interviews were audio-recorded and transcribed, and field notes were taken. The authors acknowledge they required a larger than normal sample size to achieve saturation. No information is provided for the setting of data collection.<br><br>'Other sources of data included the notes recording the interviewer's impressions.' | Researchers do not critically examine their role, potential bias and influence of research question formulation or data collection. No mention of changing the research design as the study progressed. | No mention of ethical approval, or consent gained from participants. No attempt is made to explain how confidentiality was maintained. | Nvivo was used to aid in data analysis. Open coding was used initially to generate broad categories. Towards the later stages of the process, selective coding was used to develop a meaningful narrative of the experiences of the workers. Interviewers also recorded field notes. Triangulation with members of emergency service organization was carried out throughout the research, and analysis of data was discussed. Two members of the research team developed the coding tree together. A third member reviewed the open and selective coding. It is not clear how the themes were derived from the data, but sufficient data are presented to support the findings. Contradictory data are included in the findings and discussed. | Findings are presented in sections headed by themes. Author's comments are supported by first order interpretations. Triangulation with members of emergency service organization was carried out throughout the research, and analysis of data was discussed. Two members of the research team developed the coding tree together. A third member reviewed the open and selective coding. | The authors acknowledge that the study is 'not intended to reflect the views of all paramedics in all organizations; rather, it describes a particular phenomenon experienced and described by one group of paramedics and points to some interesting avenues for further consideration. These include future attempts to measure cognitive and emotional empathy in paramedics and assess the impact of these strategies on posttraumatic and depressive symptoms and on social supports.' |

For peer review only

| | | | | | | | | | |
|---|---|---|---|---|---|---|---|---|---|
| *The meaning of traumatic events as described by nurse sin ambulance service* Jonsson et al. 2003[44] | Aims of study are clearly stated, and their importance justified. Qualitative methodology is appropriate due to nature of the aims, which is to explore subjective experiences in detail. | A phenomenological approach was used, and the reason for its us thoroughly explained. The use of self-reports over the use of interviews was not justified (the authors said that interviews and self-reports are the most common methods in empiric-phenomenological studies. | 240 medical technicians and 122 registered ambulance nurses. The authors asked 500 participants of another study to write down their experiences with traumatic events. No detail is given how the sample of the other study was recruited. Non participation is not discussed. | The question the participants were asked to answer was: 'Write down and describe a situation which you experienced as a traumatic event''. From the data returned by the participants, the authors selected 25% of the written accounts to further analyse. These written accounts were strategically selected based on having the fullest description of a traumatic event. Details of the analytic process are provided. Saturation of data is not discussed. | Researchers do not critically examine their role, potential bias and influence of research question formulation or data collection. No mention of changing the research design as the study progressed. | Ethical approval was granted. Consent was implied through willingness to participate in the study, but no information is given regarding a signed consent form. In addition, participants were assured that they could withdraw from the study at any time. The questionnaire was not labelled with the name of the participant. Confidentiality was guaranteed by eliminating names or other identifying characteristics from the essays. | Van Kaam's scientific explication was used to analyse the written stories. Detail was provided about the steps of this thematic analytic process. Only 25% of the written stories were further analysed, and were strategically selected for being more data-rich. There is no information to reveal how many researchers are involved in the analytic process. Sufficient data is presented to support the findings. There is no mention of contradictory data being taken into account. The researcher does not critically appraise their own potential bias. | The findings are in the form of themes, supported by first order data and the author's interpretations. The findings are related to the original research question in the discussion. There is no mention of any efforts to increase the credibility of the findings. | The authors recommend specific areas for further research, limited to the ambulance sector. The organisational interventions recommended by the authors are likewise limited to the ambulance sector. |
| *Guilt, shame and need for a container: a study of post-traumatic stress among ambulance personnel* Jonsson et al. 2004[45] | Aims of study are clearly stated, and their importance justified. Qualitative methodology is appropriate due to nature of the aims, which is to explore subjective experiences in detail. | Descriptive phenomenology was 'chosen for its potential to grasp the meaning of such phenomena through the description of lived experiences.' | 10 Ambulance nurses or ambulance technicians. Insufficient information on how participants were The only relevant information is that participants were strategically selected to obtain a 'variation of sex, age, educational background, and experience as ambulance staff.' | Setting for interviews was described but not justified. Interviews were open in structure, beginning with the question "tell about a traumatic event that you have experienced". 'do you mean?" or "How did you feel about that?". The interviews, taking about an hour and a half each, were audio-taped and transcribed word by word.' Reason for choice of interview as method of data collection not explained. Saturation of data not discussed. | Researchers do not critically examine their role, potential bias and influence of research question formulation or data collection. | The study was described, the extent of participation was explained, and potential risks were explored with the participants, who were assured that they had the right to withdraw from the study at any time. The study was approved by the Ethical committee at Göteborg University. To protect participant confidentiality, no quotations were provided. | The authors provide details about the 5 steps of the analytic process, with the latter steps being repeated once the author was familiar with existing philosophy. Due to the authors not using participant quotations (to protect participant confidentiality), it is not possible to determine whether the author's findings are supported by first order interpretations. Contradictory data are discussed, but without the direct participant quotations. | Findings are displayed under relevant sub-headings. In the discussion section, there is some debate for and against the researcher's arguments. No attempts made to demonstrate efforts to enhance credibility. | The results can be applied to emergency medical organisations, however caution should be taken due to the small sample size and lack of information about participant demographics. The authors recommend for the insights gained by this study to be ' distributed to all ambulance managers and other relevant personnel categories. ' The authors make specific recommendations for management. |
| *Situation Critical: High Demand, Low Control, and Low Support in Paramedic Organizations* Regehr et al. 2007[41] | Aims of study are clearly stated, and their importance justified. Qualitative methodology is appropriate due to nature of the aims, which is to explore subjective experiences in detail. | Long interview design was chosen 'to explore in detail the experiences of paramedics in their organizations including their roles, the demands placed on them, the control over the working environment that they experience, and the supports that they receive.' | 17 paramedics. Paramedics were purposively sampled from the sample of participants partaking in the quantitative component of the study. Purposive sampling was used to ensure that participants represented a wide range of experiences in terms of | A long interview method was used to collect data. The interviews followed an interview guide (not provided), were audio recorded and transcribed verbatim. The interviewer made their own notes during the interview. Setting of data collection not provided, saturation of | Researchers do not critically examine their role, potential bias and influence of research question formulation or data collection. | No mention of ethical approval, or consent gained from participants. No attempt is made to explain how confidentiality was maintained. | Data analysis commenced with open coding that captured a broad range of perspectives, whereas axial and selective coding facilitated the structuring of a coding framework. After the initial coding framework was developed, the transcribed interviews were imported into | Findings are presented in themed subheadings. Authors claim to have enhanced credibility through ' prolonged engagement and persistent observation'. No mention of more than one analyst, and qualitative findings were triangulated with quantitative results. | The authors make recommendations for EMS organisations, based on a mixture of their quantitative and qualitative results. |

For peer review only

| | | | | | | | | | |
|---|---|---|---|---|---|---|---|---|---|
| | | | length of time with the services and types of work experiences encountered. | data not discussed. | | | N*VIVO, a computer generated data analysis system, and the interview data were coded by multiple coders and subjected to detailed thematic analysis. In the final stage of analysis, constant comparative method of data analysis was implemented to compare categories and themes across respondents. Furthermore, paramedic organisations were consulted when creating the research questions, and throughout the research project to guide it with feedback. Sufficient data are presented to support the findings, and contradictory data taken into account. | | |
| *Emergency Medical Services Provider Perspectives on Pediatric Calls: A Qualitative Study* Jessica et al. 2019[46] | Aims of study are clearly stated, and their importance justified. Qualitative methodology is appropriate due to nature of the aims, which is to explore subjective experiences in detail | The use of focus groups was not explicitly justified. | 17 EMS providers. ' Paramedics and EMTs were recruited for the study through invitations circulated via weekly staff emails, flyers posted at ambulance bases, the agency's social media page, and e-mails from the EMS chaplain. Interested participants were instructed to contact a single study investigator by phone or email 'Selection criteria were applied to the participants in order to sample for the most relevant participants-participants had to be working for over a year in order to increase chance of traumatic incident exposure. Non participation was not discussed. | The process of focus group conduction was thoroughly described. A semi-structured guide was used by the researcher to conduct the focus groups-the same researchers conducted all the focus groups. At the end of the focus group, themes were fed back to participants, who were invited to comment. Focus groups were conducted at a large ambulance service. Audio recordings were transcribed. A second researcher was present during the focus group to take notes. Thematic saturation was reached. | The researchers reflect on the potential biases of the study in the limitations section. They acknowledge that by using discussion prompts, they may have steered the conversation away from topics that the participants would otherwise have talked about. Furthermore, they acknowledge that the fact that a single coder was responsible for coding 80% of the data alone may be a source of bias. | Participants gave informed consent and ethical approval was gained from an ethics committee. Resources were provided to the participants in case of experiencing psychological distress. | Transcripts were analysed using directed content analysis. Two reviewers independently coded 20% of the transcripts, and a single researcher coded the remaining data. Findings were validated through groups consensus. Quotations are provided for all sub-themes. Authors acknowledge the limitations of focus groups, and the potential for bias with one researcher coding 80% of the data. The authors also acknowledge how the discussion guide prompts may have altered the topic of discussion of participants from what they would otherwise have talked about. Contradictory data are taken into account. | Findings are presented as sub-themes, with quotations supporting each sub-theme in a separate table. Credibility of findings was enhanced by reviewing the focus group themes with participants at the end of the focus group. Furthermore, some of the data was reviewed by two researchers. | The findings are relevant to EMS organisations, specifically EMS leaders. Although Desired support mechanisms following difficult paediatric calls can be extrapolated to other types of traumatic events. |
| *An assessment of the need of police officials for* | Aims of study are clearly stated, and their | Focus groups were chosen as the method of data | 40 police officials. Participants were recruited | Setting for data collection was not outlined. Three | Researchers do not critically examine their | Ethical approval was gained for this study from | The authors describe an 8 step method of thematic | Findings are presented as sub-themes, supported by | The authors give a variety of 'preliminary indicators' |

For peer review only

| | | | | | | | | | |
|---|---|---|---|---|---|---|---|---|---|
| *trauma intervention programmes – a qualitative approach.* Boshoff et al. 2015[57] | importance justified. Qualitative methodology is appropriate due to nature of the aims, which is to explore subjective experiences in detail. | collection in order to 'allow the researcher to gain insight into participants' beliefs about and perceptions or accounts of a particular topic'. The researchers state: The focus groups allowed the researcher to interact systematically and simultaneously with several individuals. | via a purposive sampling method through the health and wellbeing departments, which were used as an intermediary between the researchers and participants. Police officials were purposefully chosen considering the relevance of the topic, specifically referring to their exposure to trauma, resultant symptoms of PTS and their participation in trauma intervention programmes. Non-participation was not discussed, other than reassuring participants that non-participation would not be sanctioned. | focus groups were carried out, and semi-structured interviews were performed within the focus groups. Exact questions asked are provided by the researchers. The sessions were audio recorded, transcribed and analysed. Saturation of the data is not discussed. | role, potential bias and influence of research question formulation or data collection. | the North-West University's ethical committee . Informed consent was taken by the researchers. ' Participants were furthermore encouraged to withdraw from the focus groups at any time should they feel uncomfortable or experience any harm or emotional consequence as a result of participating in the study.' | analysis, but do not explain how they will apply this method. It is not clear how the data is used to arrive at the themes generated. Sufficient data are presented to support the findings. Contradictory data are taken into account. | sufficient quotations. There is limited discussion for and against the researchers' arguments. No efforts are made to enhance credibility of findings. | for which a ' purposeful psycho-social trauma intervention programme' is indicated. The generalisability of this is limited to police forces. |
| *A preliminary investigation of post-traumatic stress symptoms among firefighters* Haslam et al. 2003[60] | Aims of study are clearly stated, and their importance justified. Qualitative methodology is appropriate due to nature of the aims, which is to explore subjective experiences in detail. | One-to-one interviews were chosen but reasons for their use over focus groups were not explained. The interview schedule was developed in conjunction with psychologists working within the fire service, as well using relevant literature. | 11 Firefighters, 8 Station Officers, 4 Sub Officers, 4 Leading Firefighters, 2 Fire Control Officers and 2 Area Divisional Officers. No information provided on how participants were recruited. 'The sample was selected to cover the range of positions in the service and the proportion of respondents in each position broadly reflects the profile of the service.' | ' One-to-one interviews were conducted in a private room with participants. They lasted or up to 90 minutes. The interview questions were stated by the researchers. Open-ended questions would be used to explore participants' feelings towards incidents. ;The interviews were recorded and fully transcribed and the data were analysed by sorting verbatim material into emergent themes as described by Dey (1993).'' Choice of data collection methods or study setting was not justified. Saturation of data was not discussed. | Researchers do not critically examine their role, potential bias and influence of research question formulation or data collection. The researchers acknowledge that their small sample size may not be representative. | No mention of informed consent, ethical approval or methods to ensure confidentiality. | 'The interviews were recorded and fully transcribed and the data were analysed by sorting verbatim material into emergent themes as described by Dey (1993). A second researcher independently checked the analysis to ensure analysis reliability' There is little transparency to show how the themes were developed from the primary data. Use of a second researcher enhances credibility. Direct participant quotations are sufficiently used to support the authors' interpretations. Contradictory data are taken into account. | Findings are presented as sub-headings of themes, with participant quotations supporting the authors' comments. Credibility is enhanced by the use of two analysts. | The authors provide recommendations for fire organisations. These recommendations are concerned with efforts to improve staff wellbeing. |
| *A qualitative study about experiences and emotions of emergency medical technicians and out-of-hospital emergency nurses after performing cardiopulmonary resuscitation resulting in death* | Aims of study are clearly stated, and their importance justified. Qualitative methodology is appropriate due to nature of the aims, which is to explore subjective experiences in detail. | Both interviews and focus groups were used as methods of data collection, the reason for their use was not explained. | 7 EMTs and 6 nurses. Snowball sampling was used to recruit the participants. As a prerequisite for their inclusion in this study, the health workers were required to have carried out at least 5 CPR | Questions asked during the interviews were ' developed based on the reviewed literature and experts' opinions', but the authors do not provide a topic guide or examples of other prompts. ' The assignment | Researchers do not critically examine their role, potential bias and influence of research question formulation or data collection. | ' The study was approved by the Ethics Committee of the University of the Basque Country.' 'echnique was based on their availability and preferences. Participants gave their written consent to participate in the study | A detailed description of the analytical process is provided. 'To ensure the quality of the interpretation and guarantee the reliability of the information obtained, a triangulation between all researchers involved in the | Themes are not provided with any participant quotations. 'To ensure the quality of the interpretation and guarantee the reliability of the information obtained, a triangulation between all researchers involved in the | The authors recommend greater training for health professionals when ' notifying bad news and providing psychological support to the family members of the deceased ' No recommendations for research are made. |

For peer review only

| | | | | | | | | | |
|---|---|---|---|---|---|---|---|---|---|
| Fernández-Aedo et al. 2017[47] | | | techniques resulting in death over their entire professional career. No volunteer staff was included in the study. ' In order to ensure heterogeneity, the study included healthcare professionals of different ages, both genders and varying years of experience, working at different institutions.' Non participation was not discussed. | of participants to' either individual semi-structured interviews or focus groups 'was based on their availability and preferences.' 'A total of 3 health emergency technicians and 3 nurses were interviewed individually for a total of 11---35 min by two of the researchers involved in the study. The focus group was comprised by 4 EMTs and 3 nurses, and it was used to triangulate the information obtained during the individual interviews. The group session lasted 76 min and was carried out by two researchers, one acting as a moderator and the other as an observer.' Data saturation was reached. | | and to be recorded in audio and/or video format.' | interviews was also carried out. Any disagreement was resolved by consensus.' No participant quotations are provided to support the authors' interpretations. Contradictory data re not taken into account. | interviews was also carried out. Any disagreement was resolved by consensus.' Other than the use of multiple researchers in the analytic process, no other methods to increase credibility are discussed. | |
| *Experiences of and actions towards worries among ambulance nurses in their professional life: A critical incident study* Svensson et al. 2008[48] | Aims of study are clearly stated, and their importance justified. Qualitative methodology is appropriate due to nature of the aims, which is to explore subjective experiences in detail. | There is no justification for the choice of semi-structured interviews as a method of data collection. | 25 ambulance nurses. Participants were strategically selected from three ambulance services based on 'socio-demographic and professional characteristics such as age, education level, sex and years in the profession'. Non participation is not discussed. | The authors explain the structure of the interviews, giving examples of the open-ended questions which they ask to participants at the start of the interview. The authors say the interviews took place where it best suited the participants, and three interviews were conducted over the phone. Researchers explain their use of interviews: 'Interviews were chosen as the data collection method, which allowed the respondents to describe their thoughts in more detail with the help of follow-up questions.' The authors also justify the use of the CIT method. Interviews were audio recorded and transcribed verbatim. Saturation of data was not discussed. | 'As both the researchers and the nurses were familiar with the environment in which the study was conducted, the risk of misunderstanding during the interviews was minimised.' | 'The managers of the ambulance service involved gave approval for the study to be conducted in their department.' The authors say ethical board approval is not required in Sweden under certain circumstances. The authors describe how informed consent was taken from participants, including assurances that data will be treated confidentially. 'The study adhered to the principles outlined in the Declaration of Helsinki' | 'The interviews were also read through several times before categorizing them into sub-categories, in order to further improve security. The incidents were analysed according to character and content. This step was repeated several times before the end result emerged. The categorizing of incidents into sub-categories was conducted in cooperation with the second researcher, experienced both in theory and practice, which minimised the risk of subjectivity (Andersson and Nilsson, 1964). Direct quotes from the interviews strengthened the accuracy of the study.' Contradictory data were taken into account, and sufficient are presented to | Findings are arranged into themes and sub-themes, and participant quotations are included for each theme to support the authors' conclusions. Credibility was enhanced via the use of two researchers when categorising incidents. Authors consider contradictory data in the discussion. | The authors recommend further research: 'This phenomenon should be studied more in-depth in order to map how the interaction with colleagues influences the worry among ambulance nurses.' Some recommendations are also made for employers of nurses. |

For peer review only

| | | | | | | | | | |
|---|---|---|---|---|---|---|---|---|---|
| | | | | | | | support the findings. Saturation of data was not discussed. | | |
| *Exploring the nature of resilience in paramedic practice: A psycho-social study* Clompus et al. 2016[49] | Aims of study are clearly stated, and their importance justified. Qualitative methodology is appropriate due to nature of the aims, which is to explore subjective experiences in detail. | The authors used free association narrative interviewing, a technique which involves a preliminary narrative interview, followed by a semi-structured interview. 'This enabled a deeper analysis of the affective and often unconscious aspects of paramedics' lives. ' | 'An advert, with a brief study outline, was placed in a regional Paramedic bulletin which was circulated electronically to staff with an invitation to participate.' Participants had to fulfill the following criteria: -Grade of paramedic, technician or emergency care practitioner -Willing to volunteer their time<br><br>Three out of the 10 individuals became unavailable, but the authors do not explain why. | The researchers justify their use of using Free association narrative interviewing. Saturation was achieved after the 6th interview. Details of the exact two stages of the interviewing process were provided by the authors. interviews were audio recorded and transcribed, and carried out at a place of the participants' choosing. | Researchers do not critically examine their role, potential bias and influence of research question formulation or data collection. | ' Ethical approval was received from a (NHS) research ethics committee and a university in the SW of England. Confidentiality and anonymity was secured through the use of pseudonyms for participants, and all data were kept in a password protected personal computer with access limited to SC. Participants were made aware that they could withdraw at any time and that the anonymised data would be disseminated in various ways. Due to the potential distress that participation could inadvertently provoke, information on how to access counselling services was made available to all participants.' | Details of the FANI analytic process were given by the researchers. 'Any discrepancies were examined and discussed until consensus was reached. Trustworthiness and data credibility were established by several means including participants feeding into the study's aims, keeping contemporaneous notes, and sending interview transcripts and a summary of findings to each participant for verification'. The authors are not clear how many researchers are involved in the analytic process. For each theme, sufficient participant quotes are provided to support the authors' interpretations. Contradictory data were taken into account and discussed. | Themes are presented as sub-headings, and supported by the direct participant quotations. Member validation was carried out to improve credibility. | The authors relate the findings of the research to the theories of exiting organisational interventions, eg TRiM. The authors conclude that for front-line paramedics, ' applying interventions and reviewing support mechanisms would seem to be a pressing imperative.' |
| *First response emergency care – experiences described by firefighters* Abelsson et al. 2019[61] | Aims of study are clearly stated, and their importance justified. Qualitative methodology is appropriate due to nature of the aims, which is to explore subjective experiences in detail. | The authors justify their choice of group interviews, and why limits are set on group interviews: 'The group sizes of eight and nine participants were considered acceptable to moderate and managed by the researcher. Interaction in the pre-existing group is key to a successful group interview. In this study, all firefighters worked at the same fire station, which promoted interactions optimal for the research purpose' | Authors don't explain how participants were recruited into the study. | 35 Firefighters. Setting for group interviews was not given. Participation numbers in the group interviews were provided and discussed. Justification for methods chosen was provided. A rough structure to the interviews was provided by giving questions which were asked to participants. There is no mention of saturation of the data. The authors reflected in the 'limitations' section that group interviews could lead to participants being uncomfortable. | Researchers do not critically examine their role, potential bias and influence of research question formulation or data collection. | No approval has been sought from an ethics committee, which is in accordance with Swedish law. Consent was implied through participation in the study, following the delivery of verbal 'clear information' by the researchers. Methods taken to protect confidentiality are described. | The authors describe their method of text-driven, interpretive qualitative content analysis. This involved repeated readings of the transcribed interviews, and then identification of 'meaning units', then codes were derived, and formed into sub-categories and categories. Sufficient data are present to support the findings. Contradictory data are discussed. It is not clear how many researchers were involved in the analytic process. There is no explicit mention of efforts to improve credibility. Authors acknowledge that | Findings are presented as four themes, with consistent use of primary quotations to support the researchers' arguments. The findings are discussed in relation to the original research question. | The researchers contrast their findings with other research in the field, such as in the case of spouses being used as sources of support by firefighters. They also recommend areas of future research, eg involving other strands of emergency service workers. |

For peer review only

| | | | | | | | | | |
|---|---|---|---|---|---|---|---|---|---|
| | | | | | | | 'participants in group interviews may experience a pressure within the group, resulting in similar opinions'. | | |
| *Paramedics' experiences with death notification: a qualitative study*<br>Douglas et al. 2013[50] | Aims of study are clearly stated, and their importance justified. Qualitative methodology is appropriate due to nature of the aims, which is to explore subjective experiences in detail. | The authors used focus groups as their method of data collection, but did not justify their use over other qualitative methods. | 28 paramedics. Participants were self-selected. Recruitment messages were delivered via 'departmental e-mail, flyers,<br>and by word-of-mouth'. Authors don't explain why the participants selected were the most appropriate for the study, and non-participation is not discussed. | The authors provide the locations of the focus groups, and give examples of questions asked during the sessions. There is no explicit justification for why focus groups were chosen over other qualitative methods. Methods were modified during the study as follows: 'The question guide was adapted as required by the author and the facilitator after each session to clarify some questions based on the paramedics' responses.' Focus groups were audiotaped and transcribed. Saturation was discussed: the authors kept recruiting participants until saturation was reached. | The researchers acknowledged that their choice of focus group location, as well as the presence of the supervisor in the focus group may have affected the answers given by participants. The researchers do not critically examine their own role. | Ethical board approval was gained, and written consent obtained. No information is provided on efforts to maintain participant confidentiality. | The authors describe an inductive approach to data analysis. Two researchers were involved in the analysis process. Themes were discussed with a further two authors, until consensus was reached. Credibility was enhanced via the use of member checking the participant responses with participants at the end of focus groups. Contradictory data are taken into account, and sufficient data are presented to support the findings. | The findings are presented as themes, supported by participant quotations and authors' interpretations. Credibility was enhanced by distributing results from the study to participants for the purpose of generating feedback. | The findings are discussed in relation to existing literature, and the authors make recommendations for practice, specifically regarding paramedic training. |
| *Peer-support: a coping strategy for nurses working at the Emergency Ambulance Service*<br>Carvello et al. 2019[51] | Aims of study are clearly stated, and their importance justified. Qualitative methodology is appropriate due to nature of the aims, which is to explore subjective experiences in detail. | The authors used semi-structured interviews. The authors explain their choice of questions result from pre-existing literature, but do not justify the use of interviews over other qualitative methods eg focus groups | 'Participants were recruited on a voluntary basis from an emergency medical service in the north of Italy.' The sampling was 'non-probabilistic', according to the following criteria: nurses working at the emergency ambulance service "118" in a hospital in northern Italy, who had experience in the extra-hospital emergency. Non-participation is not discussed. | Data collection was at an italian emergency service organization called 118. The justification given by the researchers for choosing this location was to choose an environment familiar to the participants so that they would feel at ease. Semi-structured interviews are carried out with participants. The authors explain how they chose their questions, by taking inspiration from similar literature, but choice of interviews over other qualitative methods is not explained. 'The interviews were audio-recorded and conducted | Researchers do not critically examine their role, potential bias and influence of research question formulation or data collection. | Ethical approval was gained from the Bioethical Committee of the University of Bologna. Informed consent was gained from participants before participation. Efforts to protect confidentiality are not discussed. | 'The interviews were conducted and analyzed by all the researchers after having been faithfully transcribed on digital text documents, reporting in brackets some relevant non-verbal gestures, and after having evaluated the nodes and relationships generated by the nVivo qualitative research software12.' Although sufficient data are presented to support the findings, it is unclear how the themes were generated from the data. | Findings are presented as themes. Authors discuss arguments for and against certain issues raised. One example of this is the inclusion of quotations of participants who prefer talking to a peer supporter, and those who prefer talking to a professional therapist. No mention is made of efforts to discuss the credibility of findings. Contradictory data are taken into account and discussed. | The authors relate their findings to their aims. They propose that a peer support program should be introduced in the ambulance service. No recommendations for further research are made. |

| | | | | | | | | | |
|---|---|---|---|---|---|---|---|---|---|
| | | | | anonymously'. Saturation of data is not discussed. | | | | | |
| *Experiences among firefighters and police officers of responding to out-of hospital cardiac arrest in a dual dispatch programme in Sweden: an interview study* Hasselqvist-Ax et al. 2019[63] | Aims of study are clearly stated, and their importance justified. Qualitative methodology is appropriate due to nature of the aims, which is to explore subjective experiences in detail. | The authors don't justify their use of semi-structured interviews over other qualitative methods. | 10 police officers, 12 firefighters. Participants were purposively sampled for knowledge of two or more cardiac arrest situations, and to collect as rich descriptions as possible. Three recruitment approaches were used: '1) an invitation letter from the researchers was presented to the main collaboration group for OHCA alarms in Stockholm County; 2) on the police report for cardiac arrest alarms there was a request to contact the researchers for a voluntary interview; 3) fire stations were directly contacted for recruitment of participants.' Non-participation was not discussed. | The authors provide an interview guide with 7 open-ended questions, which form the basis for the semi-structured interviews. Setting for interviews: all but three took place at regular work places (not known where the others took place). Critical interview technique (CIT) was chosen as the method for data collection. The authors don't justify their use of this technique, but they justify their sample size based on the recommended sample size for CIT. Interviews were recorded and transcribed. The authors state no modification of methods during study were necessary. Saturation was discussed in relation to the critical incident technique- where 20 interviews (of 2-4 CIs per interview) provide sufficient data. The authors included 22 participants. | All interviews were conducted by the same author, to increase the chance that they were conducted in a similar way. The authors list the researchers' relevant strengths and past experiences, demonstrating reflexivity. | Ethical approval was obtained. Written informed consent was obtained from the participants and information was given about the possibility to withdraw from the study without any reprisal. Participants were not entitled to financial remuneration or other benefits. | 'This was an interview study where data were analysed by using critical incident technique (CIT) and inductive qualitative content analysis.' The authors explain the CIT process step by step, and provide an example with a piece of interview data. To enhance credibility, the researchers discussed the analysis at each step of the process, ensuring all analyses were supported by data. Occasionally the views of participants are summarised without examples of quotations. Contradictory data are taken into account, but saturation of data is not discussed. | The findings are presented as themes. Although participant quotations were provided, the authors didn't provide quotations for all of their comments. Often, the views of participants were summarised without providing participant quotes. | The authors provide recommendations for swedish emergency organizations, such as giving indications for training of paramedics and firefighters. |
| *Working in prehospital emergency contexts: Stress, coping and support from the perspective of ambulance personnel* Oliveira et al. 2019[52] | Aims of study are clearly stated, and their importance justified. Qualitative methodology is appropriate due to nature of the aims, which is to explore subjective experiences in detail. | The authors don't explain why semi-structured interviews were chosen over other qualitative methods. | 14 ambulance personnel. Participants were recruited from seven institutions, mainly on the basis of 'having an active status on a rescue team and being willing to talk and share their experiences'. Details of how participants were recruited are not provided, and non-participation was not discussed. | Interviews took place at the participants' local professional organisation, and were all conducted by the first author. Semi-structured interviews were conducted with participants following an interview guide, details of which are provided by the authors. The choice of semi-structured interviews was justified as it allowed the authors 'to obtain in-depth information regarding participants' prehospital emergency experiences'. Interviews were audio-recorded and transcribed. Saturation of | The researchers acknowledge the possibility of researcher bias during the coding process, and therefore they discuss their analyses with other researchers. | The research project was approved by the Portuguese Red Cross and by the University's Ethics Committee Written consent to participate in the study and to audio-record the interview was obtained, as follows: 'We sent a cover letter to all selected structures, explaining the aim of the study, the procedures, the ethical issues guaranteed, the voluntary character of the participation and the possibility to withdraw at any time'. | The authors use Braun and Clark's analytic approach, and outline the process in detail. This includes explaining how themes were derived from the data. Data collection was performed until saturation was reached. Data was discussed between the researchers during coding, enhancing credibility. The researchers made efforts to reduce researcher bias: 'Furthermore, to reduce research bias, there was a concern to engage with other researchers to discuss the process of data | Findings are presented as themes. The researchers provide participant quotations to support their findings. Multiple analysts discussed the analytic process to reduce researcher bias. | The researchers make recommendations to ambulance organisations to improve the psychological wellbeing of their staff. The authors also provide multiple avenues of recommended further research. |

For peer review only

| | | | | | | | | | |
|---|---|---|---|---|---|---|---|---|---|
| | | | | data was achieved. | | | analysis and to illustrate themes with verbatim descriptions from participants.' Sufficient data are presented to support the findings, and contradictory data are taken into account. | | |
| *Exploring paramedic communication and emotional expression in the workplace after responding to emergency calls* Drewitz-Chesney et al. 2019[53] | Aims of study are clearly stated, and their importance justified. Qualitative methodology is appropriate due to nature of the aims, which is to explore subjective experiences in detail. | The authors don't explain why semi-structured interviews were chosen over other qualitative methods. | 8 paramedics. The recruitment strategy is explained in detail. Participants were recruited over facebook groups. 'Participants were sampled using convenience, then purposive sampling. Convenience sampling enabled initial recruitment. Snowball and quota sampling were the two forms of purposive sampling used.' Authors are not clear on which attributes are sampled for. The authors justified their choice of participants by saying that 'when sufficient information is gleaned from participants, a smaller sample size is required ' | Semi-structured interviews were performed with participants, but the researchers do not justify why interviews are chosen. Interviews were conducted using Skype, and participants could choose to interview via video or audio only. An interview guide is provided. Interviews were audio recorded and transcribed, and data collection was stopped once saturation was reached. | The recruitment messages mention that the researcher previously worked as a paramedic. The researchers acknowledge the possibility of confusion and bias that can arise from this, although this was minimised by the fact that the researcher didn't work with any of the participants. | 'This study received ethical approval from the University of Edinburgh's Usher Research Ethics Group. Each participant provided verbal and written consent. To minimise the risk of psychological impact, participants were never asked to recall specific calls or details. Participants were monitored for signs of distress during each interview (none were noted). BCEHS paramedics have access to three services offering support and counselling. These services were listed on their information letters. At the conclusion of each interview, participants were asked if they wanted a referral to any of the services, which all participants declined.' | The authors used constructivist grounded theory in their analytic process. The authors are transparent about how themes are generated from the data.' Credibility was enhanced through triangulation, including interviewing the manager of the BCEHS CIS program, Marsha McCall, and Anonymous, a retired BCEHS paramedic, whom substantiated some participant data . Thick descriptions and a diverse sample contributed to credibility and transferability. An audit trail was maintained throughout the research process which enhanced dependability. Journaling and bracketing enriched reflexivity ' 'Each transcript was read at least three times to improve accuracy and familiarity'. Sufficient data are presented to support the findings, and contradictory data are taken into account. | Quotations are interspersed among the results which are presented as themes. Credibility was enhanced through the use of triangulation with leading members of the organisation. | The researchers consider their findings in relation to existing literature. They also make various recommendations for emergency service organisations, relating to post-incident organisational interventions that could protect paramedic wellbeing. |
| *Mental Health in the UK Police Force: a Qualitative Investigation into the Stigma with Mental Illness* Edwards et al. 2020[58] | Aims of study are clearly stated, and their importance justified. Qualitative methodology is appropriate due to nature of the aims, which is to explore subjective experiences in detail. | The authors justify their use of open-ended questions in semi-structured interviews to facilitate meaningful discussion, although the choice of interviews other qualitative data collection methods is not justified. | Five police officers. 'Participants were recruited through a referral from a charity, personal network, police forum and contact from within the police force.' The authors outline the criteria for participant inclusion in the study. They do not however justify their | Semi-structured individual interviews were conducted using a topic guide, of which the authors provide details. The authors justify their use of open-ended questions. Interviews were audio recorded and transcribed. Data saturation was not discussed. | Researchers do not critically examine their role, potential bias and influence of research question formulation or data collection. | Ethical board approval was obtained, as well as written consent from the participants. The researchers also had measures in place to support participants who experience distress due to participation in the study. | The authors used Braun and Clark's approach of thematic analysis. They outlined this process in detail, giving examples of how they arrived at themes from the data. It is unclear whether multiple researchers collaborated to compare coding. Participant quotations are | Findings are presented as themes and sub-themes. The authors include contradictory data, and discuss this. Discussion takes place throughout the study within the results section, but there is also a separate discussion section. | The authors identify areas where further research would be valuable, such as barriers to help-seeking in male dominated professions. Recommendations are also made to police organisations, such as highlighting a need to increase mental health |

For peer review only

| | | | | | | | | | |
|---|---|---|---|---|---|---|---|---|---|
| | | | choice for these criteria. Non-participation was not discussed. | | | | consistently embedded within the authors' interpretations, and contradictory data are taken into account. The authors are transparent about how themes were generated. | | awareness. |
| *Living in Critical Times: The Impact of Critical Incidents on Frontline Ambulance Personnel: A Qualitative Perspective* Gallagher et al. 2007[54] | Aims of study are clearly stated, and their importance justified. Qualitative methodology is appropriate due to nature of the aims, which is to explore subjective experiences in detail. | The authors don't justify why they chose individual interviews over other qualitative methods. They do however justify their use of some closed questions ( to elicit background information and to facilitate comparisons across participants. ) | 21 EMTs, 6 EMCs. The qualitative component of this study followed a quantitative Survey. Participants from the earlier study were asked if they would be willing to participate in qualitative interviews. Non-participation is not discussed. | Setting for data collection is not stated. The authors give examples of the topics discussed during the interview process. The interview schedule used was created with the help of a literature review and the findings from the quantitative component of the study. No justification for the choice interview method is given. The interviews were audio recorded and transcribed. | Researchers do not critically examine their role, potential bias and influence of research question formulation or data collection. | No mention of informed consent, ethical approval or methods to ensure confidentiality. | Thematic analysis was undertaken. 'A random sample of transcripts was read and coded by both authors in order in ensure good reliability and validity.' It is not clear how the themes were derived from the data. The authors don't critically evaluate their own role in the analytic process. Contradictory data are taken into account- for example when giving examples of participant quotations with conflicting attitudes. The researchers don't critically examine their own role. Sufficient data are presented to support the findings. | Findings are presented as themes. For each theme, a range of relevant quotations are provided to support this theme. Credibility of findings is not discussed. | The authors make recommendations to police organisations for post critical incident support for staff. |
| *Police officers, mental (ill-) health and spoiled identity* Bullock et al. 2018[59] | Aims of study are clearly stated, and their importance justified. Qualitative methodology is appropriate due to nature of the aims, which is to explore subjective experiences in detail. | Researchers justified their use of telephone interviews: '. While telephone interviews are often depicted as a less attractive alternative to face-to-face interviewing, telephones may allow respondents to feel relaxed, more able to disclose sensitive information and there is little evidence that they produce lower quality data ' | 52 police officers, two police staff, four Police Community Support Officers (PCSOs) (four) and one special constable. Participants were recruited from six police constabularies in England and Wales. 'Individual participants were identified by virtue of their contribution to an online survey on the nature of work-related injury in which they identified themselves as willing to participate in a full-length interview'. This study is part of a wider project. Non-participation was not discussed. | The researchers explain in detail how the telephone interviews were conducted. Open questions were asked by the participants. Saturation of data is not discussed. 'All interviews were digitally recorded, professionally transcribed and anonymized. ' | The researchers acknowledge that the nature of the interview is sensitive, and that the nature of telephone interviews would make participants more comfortable disclosing such information. | ' The nature of the interviewing was inevitably sensitive and mechanisms were put in place to mitigate that; for example, interviewees were provided with the contact details of sources of support.' There is no mention of ethical board approval, informed or methods to protect patient confidentiality. | The authors use Braun and Clark's thematic analysis approach, and give a very brief overview of the approach, but they do not give examples of how themes were derived from the data. The researchers didn't critically examine their own role. Sufficient data are presented to support the findings, and contradictory data are taken into account. Data saturation is not discussed. | The findings are presented as themes. For each theme, the authors use participant quotations as well as relevant literature to discuss the context surrounding the quotations. No efforts to enhance credibility are discussed. | The authors make recommendations to police organisations based on their findings surrounding stigma. No recommendations for research are made. |

For peer review only

| | | | | | | | | | |
|---|---|---|---|---|---|---|---|---|---|
| *'You see a baby die and you're not fine:' a case study of stress and coping strategies in volunteer emergency medical technicians* Folwell et al. 2018[55] | Aims of study are clearly stated, and their importance justified. Qualitative methodology is appropriate due to nature of the aims, which is to explore subjective experiences in detail. | The authors justify their choice of in-depth interviewing technique as they 'hope to gain a deeper understanding of the lived experiences of voluntary EMTs'. | 25 EMTs. Participants were recruited from one county in a Western state. It is not clear how the participants were selected, and non-participation is not discussed. | Interviews were conducted in a private space of the participant's fire department. Justification for choice of interview setting was not provided. In-depth interviews were performed with participants, using an interview guide. This was chosen to ''gain a deeper understanding of the lived experiences of voluntary EMTs''. Saturation was reached by the end of the study. Interviews were audio recorded and transcribed. | 'The research team consisted of a female Caucasian professor who holds a doctorate and a male Caucasian undergraduate student with three years' experience as a volunteer EMT. While some of the interviews were conducted by both members of the research team, most interviews were conducted by the volunteer EMT'. Other than providing these details, Researchers do not critically examine their role, potential bias and influence of research question formulation or data collection. | Confidentiality was guaranteed by the authors as the transcripts were anonymised. There is no mention of ethical board approval or informed consent. | The authors describe the process of constant comparison analysis. Two researchers independently read all the transcripts, and collaborated to discuss findings. The authors are very transparent about the analytic process. Contradictory data were discussed, such as in the example of participants discussing the pros and cons of CISD. Sufficient data are presented to support the findings. | Findings are presented as themes and sub-themes. 'To enhance credibility, member validation was performed in which a summary of findings and initial interpretations of data were given to five participants to confirm the researchers accurately depicted viewpoints and experiences. To enrich transferability, we provided detailed descriptions of participants and research sites. To improve dependability, we used the same protocol for each interview and documented the process of data collection and analysis. To address confirmability, we used direct quotes from participants to support the finding' | The authors make four detailed recommendations to the specific EMS organisation with which the study was involved. The authors also make recommendations for future research. |

For peer review only

Supplementary File 2:

ENTREQ checklist (Enhancing transparency in reporting the synthesis of qualitative research)[83]

| No | Item | Guide and description |
|---|---|---|
| 1 | Aims | To synthesise the evidence on:<br>1. The factors that contribute to mental health recovery as expressed by psychologically distressed emergency service staff following occupational critical incident exposure<br>2. The barriers to and facilitators of mental health help-seeking behaviour among psychologically distressed emergency service staff |
| 2 | Synthesis Methodology | Thematic synthesis |
| 3 | Approach to searching | Pre-planned comprehensive search strategies, combining database searching with manual search methods. |
| 4 | Inclusion criteria | Articles were eligible for inclusion provided they met the following criteria:<br>(1) Participants were frontline ESWs; (2) There must be a focus on work-related psychological distress; (3) Include primary qualitative interviews, focus groups or observational methods; (4) Participant attitudes towards: a) pro-active action aimed at improving mental health after experiencing a traumatic event OR b) factors which emergency service workers find helpful or unhelpful for their mental health while experiencing work-related psychological distress, are explored; (5) Published in English and peer-reviewed. |
| 5 | Data sources | OVID MEDLINE, EMBASE, PsycINFO, SCOPUS. To identify articles missed in the electronic database search, the following methods were also employed:<br>-Using 'related article' feature (when available).<br>-Searching the titles of included studies in google scholar for citation tracking purposes.<br>-Manual searching of the references of relevant studies (reference chaining).<br>Grey literature was searched during background research for context but not to locate eligible studies. |
| 6 | Electronic search strategy | The two combinations of search criteria (see Appendix A) were entered into each database. Figure 1 provides an overview of the database search. |
| 7 | Study screening methods | The elegibility criteria were applied against the titles of the database search results by one reviewer (NA). The full text of studies with potential relevance was screened. (Figure 1) |
| 8 | Study characteristics | Study characteristics are presented in Table 1. |
| 9 | Study selection results | PRISMA guidance was used to construct a flow diagram displaying the database searching process (Figure 1). Of the 13381 records identified once duplicates were removed, the full text of 42 articles were screened, and 24 studies were included in this qualitative synthesis. |
| 10 | Rationale for appraisal | All potentially relevant studies were quality appraised by one reviewer (NA), using the Critical Appraisal Skills Program (CASP) guidelines. Studies meeting less than 5 criteria were then subject to further scrutiny, in the form of five quality appraisal prompts developed by Dixon-Woods et al. (see 'quality appraisal' section). This second stage of appraisal was carried out independently by by RR, MB or JW, and decisions relating to the inclusion of these studies in the review were made following thorough communication between reviewers and referring to pre-determined quality prompts. |
| 11 | Appraisal terms | Critical Appraisal Skills Program (CASP) guidelines and quality appraisal prompts (see 'quality appraisal' section) were used to quality appraise all included studies. |
| 12 | Appraisal process | Quality assessment was carried out primarily by one reviewer (NA), with independent verification from RR, MB or JW for ambiguous studies. |
| 13 | Appraisal results | CASP scores of included studies are provided in Table 1. Full study quality assessments are available for review if required. |
| 14 | Data extraction | A data extraction template (Appendix B) was created for this review by one reviewer (NA). Sections for first, second and third order constructs are included in the extraction template, which was filled manually by one reviewer (NA). |

For peer review only

| | | |
|---|---|---|
| 15 | Software | N/A. |
| 16 | Number of reviewers | Two reviewers were involved in the coding and analysis (NA, RR). |
| 17 | Coding | One reviewer carried out line-by-line coding (NA). RR aided with the grouping of codes, and assisted with the generation of descriptive themes. |
| 18 | Study comparison | During primary readings of the studies, overarching concepts relating to the research aims were noted. The generation of new codes altered pre-existing codes. |
| 19 | Derivation of themes | Thematic generation was carried out in correspondence between two reviewers (NA and RR), and was grounded in the extracted data. This was an inductive approach. |
| 20 | Quotations | Appendix C provides example literature quotations from included studies which were used to construct themes. |
| 21 | Synthesis output | This qualitative review generated 14 descriptive themes. These are grouped into either 'factors contributing to mental health recovery post traumatic incident exposure' or 'factors influencing mental health help-seeking behaviour'. Table 2 presents a summary of themes. These descriptive themes were applied to the context of UK emergency service organisations to produce implications for practice and research. |

For peer review only

BMJ Open

# The barriers and facilitators to help-seeking and psychological protection of trauma-exposed emergency service staff: A qualitative evidence synthesis

| | |
|---|---|
| Journal: | *BMJ Open* |
| Manuscript ID | bmjopen-2020-047814.R1 |
| Article Type: | Original research |
| Date Submitted by the Author: | 27-Jun-2021 |
| Complete List of Authors: | Auth, Niklas; University of Birmingham, College of Medical and Dental Sciences<br>Booker, Matthew; University of Bristol, School of Social and Community Medicine<br>Wild, Jennifer; University of Oxford, Experimental Psychology<br>Riley, Ruth; University of Bristol, Social and Community Medicine; University of Birmingham, of Applied Health Research |
| <b>Primary Subject Heading</b>: | Occupational and environmental medicine |
| Secondary Subject Heading: | Mental health, Medical management, Qualitative research |
| Keywords: | QUALITATIVE RESEARCH, MENTAL HEALTH, OCCUPATIONAL & INDUSTRIAL MEDICINE, Health policy < HEALTH SERVICES ADMINISTRATION & MANAGEMENT |
| | |

SCHOLARONE™
Manuscripts

*I, the Submitting Author has the right to grant and does grant on behalf of all authors of the Work (as defined in the below author licence), an exclusive licence and/or a non-exclusive licence for contributions from authors who are: i) UK Crown employees; ii) where BMJ has agreed a CC-BY licence shall apply, and/or iii) in accordance with the terms applicable for US Federal Government officers or employees acting as part of their official duties; on a worldwide, perpetual, irrevocable, royalty-free basis to BMJ Publishing Group Ltd ("BMJ") its licensees and where the relevant Journal is co-owned by BMJ to the co-owners of the Journal, to publish the Work in this journal and any other BMJ products and to exploit all rights, as set out in our licence.*

*The Submitting Author accepts and understands that any supply made under these terms is made by BMJ to the Submitting Author unless you are acting as an employee on behalf of your employer or a postgraduate student of an affiliated institution which is paying any applicable article publishing charge ("APC") for Open Access articles. Where the Submitting Author wishes to make the Work available on an Open Access basis (and intends to pay the relevant APC), the terms of reuse of such Open Access shall be governed by a Creative Commons licence – details of these licences and which Creative Commons licence will apply to this Work are set out in our licence referred to above.*

*Other than as permitted in any relevant BMJ Author's Self Archiving Policies, I confirm this Work has not been accepted for publication elsewhere, is not being considered for publication elsewhere and does not duplicate material already published. I confirm all authors consent to publication of this Work and authorise the granting of this licence.*

For peer review only

# The barriers and facilitators to help-seeking and psychological protection of trauma-exposed emergency service staff: A qualitative evidence synthesis

Word count: 6249

**Corresponding Author:**
Niklas Auth
University of Birmingham
Tel: 07475545757
Email: nma676@student.bham.ac.uk

Matthew Booker
GP & NIHR Clinical Lecturer in Primary Care (Urgent Care)
Centre for Academic Primary Care (CAPC)
Bristol Medical School
University of Bristol

Jennifer Wild
Associate Professor
Department of Experimental Psychology
Oxford Centre for Anxiety Disorders & Trauma
University of Oxford

For peer review only

Ruth Riley

Lecturer in Applied Health Research

College of Medical and Dental Sciences

University of Birmingham

For peer review only

## Abstract

**Objectives**

To identify factors and contexts contributing to mental health protection and recovery of emergency service workers (ESWs) following exposure to occupational trauma, and barriers and facilitators to mental health help-seeking behaviour among trauma-exposed ESWs.

**Background**

ESWs are vulnerable to mental ill health, with occupational exposure to trauma a major contributing factor. This has implications for workforce sustainability. Types of



For peer review only - http://bmjopen.bmj.com/site/about/guidelines.xhtml

organisational interventions offered to trauma-exposed ESWs are inconsistent across the UK, with uncertainty around how to engage staff.

**Design**

Four databases (OVID MEDLINE, EMBASE, PsycINFO and SCOPUS) were systematically searched from January to March 2020., with citation tracking and reference chaining. A qualitative evidence synthesis was carried out on 24 qualitative studies. A modified Critical Appraisal Skills Programme tool and quality appraisal prompts were used to identify fatally flawed studies. The PerSPEcTiF framework was used to define eligibility criteria, and data was extracted using a customised extraction table. Drawing upon the principles of thematic synthesis, a qualitative synthesis was performed based on line-by-line inductive coding of included studies.

**Results**

Fourteen descriptive themes emerged from this review, categorised into two overarching constructs:  factors contributing to mental health recovery (such as the need for downtime, peer support and reassurance) and factors influencing help-seeking behaviour (such as

For peer review only

stigma, the content, form and mandatory nature of interventions, and mental health literacy (i.e. emotional awareness, education).

## Conclusion

The juxtaposition of ESW perceptions and organisational priorities highlighted by this review provide new insights about the applicability and usefulness of trauma interventions. Following traumatic exposure, ESWs appear to benefit from recovery time and informal support from trusted colleagues. Facilitation of ESWs' ability to detect and respond appropriately to trauma-related emotional reactions may have organisational benefits. A culture which encourages help-seeking and open dialogue surrounding mental health, and discourages stigma is pivotal to bolster mental health recovery.

For peer review only



For peer review only - http://bmjopen.bmj.com/site/about/guidelines.xhtml

**Keywords:** Qualitative research, mental health, emergency responders, thematic synthesis, psychological trauma, qualitative evidence synthesis

## Strengths and Limitations

With respect to reflexivity, the analysis constitutes multiple perspectives on the topic, compromising of a primary care and ambulance clinician, a medical student, and two applied health researchers. Only English language studies were included, and despite not excluding studies on the basis of age or setting location, all but one of the studies[67] were set in socioeconomically western countries; therefore findings may not be transferable to all countries. Study quality varied significantly, with research design commonly being inadequately explained. Only one reviewer performed the initial screen of studies, possible allowing for selection bias, however a second reviewer was recruited to independently assess potentially relevant studies against eligibility criteria.

For peer review only

For peer review only

## Introduction

Emergency service workers (ESWs) consisting of members of the emergency medical services (EMS), the fire service and the police force, consistently experience poorer mental health outcomes when compared to the general population. While subtle differences exist



For peer review only - http://bmjopen.bmj.com/site/about/guidelines.xhtml

between occupational groups within emergency service organisations (ESOs), ESWs, also referred to as first responders, experience disproportionately higher rates of post-traumatic stress disorder (PTSD), anxiety, depression and psychological distress.[1-5] Suicide attempts by ESWs are considerably more prevalent than the estimated rate of 0.5% in the general population.[6] Exposure to traumatic events accounts for higher PTSD rates in the ESW cohort,[2] and is the second most commonly reported cause of poor mental health among UK ESWs in a 2019 survey, following excessive workload.[7] This review will consider the terms 'traumatic incident/event' synonymously with the term 'critical incident', which is defined as: 'any event with sufficient impact to produce significant emotional reactions in people now or later', as described by Mitchell and Everly.[8] The increased incidence of PTSD in ESW populations[1,2] is important to appreciate as PTSD is a risk factor for suicidal ideation and risky behaviours in civilian and military populations,[9,10] and increases suicidal risk in ESWs.[11,12] The wider impacts of mental ill health among ESWs include high rates of absenteeism and presenteeism, resulting in significant costs to emergency service organizations.[13]

ESOs employ a variety of programmes to prevent the development of mental ill health in trauma-exposed staff, of which main categories include stress management, psychotherapy

For peer review only

and health promotion.[14] Psychotherapy in the form of single-session session stress debriefing has widespread historical and current use in ESOs[15-17], but by the early 2000s, a substantial body of evidence had emerged highlighting its ineffectiveness at preventing PTSD, as well as potentially exacerbating symptoms by interfering with natural coping mechanisms.[15,16,18-20] The following two interventions are emerging among UK ESOs, and are examples of prevention strategies hoped to replace traditional debriefing methods. Trauma Risk Management (TRiM) is a peer support system, delivered by trained volunteers belonging to the organisation,[21] who psychologically risk assess individuals exposed to traumatic events.[22,23] The evidence surrounding TRiM's impact on users' mental health outcomes or positively altering mental health stigma is inconclusive.[23-26] Schwartz rounds are a 'cultural change initiative' also emerging in the UK,[27] which allow multidisciplinary healthcare staff to share and discuss 'non-clinical aspects (e.g. psychosocial, ethical and emotional issues),[28] and as of May 2020 have been adopted by four UK ambulance trusts.[27] Schwartz rounds are reported to improve staff psychological wellbeing and increase 'empathy and compassion for colleagues',[29] although have not been formally evaluated. Other interventions provided by UK ESOs to support the emotional wellbeing of ESWs after attending to critical incidents



For peer review only - http://bmjopen.bmj.com/site/about/guidelines.xhtml

include counselling and 24-hour helplines,[30-34] ‘defusing’ programmes[35] and peer support networks.[36]

Despite the availability of interventions, ESWs experience barriers to mental health care with one third experiencing mental health stigma, a rate that is higher than the general population.[37] The purpose of this review is to identify the factors associated with psychological protection of trauma-exposed ESWs, and the mental health recovery of those ESWS experiencing distress linked to critical incidents. This review will also identify the barriers to and facilitators of mental health help-seeking behaviour among trauma-exposed and psychologically distressed ESWs.

By identifying important contextual factors which help and hinder staff when they access support, and illuminating benefits and disbenefits of current organisational interventions, this review aims to offer qualitative insights grounded in the perceptions of ESWs, which may help ESOs in decision-making about psychological support for their staff following traumatic incident exposure.

For peer review only

This review was initiated in collaboration with key ambulance management stakeholders who expressed a need for research to guide decisions about wellbeing interventions for frontline staff, therefore the focus is on ambulance trusts, although the findings will also be of relevance to fire and police organisations.

For peer review only

## Methods

Methodology

The analytical technique of thematic synthesis, as described by Thomas and Harden, was used to conduct this qualitative review.[38] This technique used inductive line-by-line coding and is focussed around: intervention need, appropriateness, acceptability and effectiveness,[39] and is an established method in the field of mental health to investigate barriers to help-seeking, and to synthesise qualitative evidence about participant experiences.[37,40,41] It is therefore appropriate given that this review's purpose is to inform UK emergency service organisational guidelines. The research question and final search terms were ratified by a

consensus panel of key stakeholders drawn from UK ambulance services. This group contributed to the development and refinement of the review questions, search parameters and application of the review findings. The methods of this review are reported using the ENTREQ framework (Supplementary File 1).

Eligibility criteria

In keeping with recent qualitative review guidance[42], the PerSPEcTiF framework was used to enhance description of inclusion criteria.[43] (Table 1)

**Table 1: The PerSPEcTiF question formulation framework[43]**

| Perspective | Setting | Phenomenon of interest/Problem | Environment | (Optional comparison, not applicable) | Time/timing | Findings |
|---|---|---|---|---|---|---|
| Emergency service workers | Emergency frontline ambulance, police or fire service work | How does the phenomenon of participant attitudes towards: a) behaviour aimed at improving mental health after experiencing a traumatic event OR b) factors which ESWs find helpful or unhelpful for their mental health while experiencing work-related psychological distress | Poor mental health outcomes and elevated rates of mental health stigma within the emergency services | | Following occupational exposure to traumatic event(s) | In relation to the emergency service workers' perceptions and experiences |


For peer review only - http://bmjopen.bmj.com/site/about/guidelines.xhtml

**Inclusion criteria:**

(* indicates further information below)

(1) Study participants were frontline ESWs* (studies with mixed populations of eligible participants were included); (2) There is a focus on work-related psychological distress*; (3) Data collection includes primary qualitative interviews, focus groups or observational methods (this included mixed methods studies with qualitative components); (4) Analysis focussed around participant attitudes towards: a) behaviour aimed at improving or protecting mental health after experiencing a traumatic event OR b) factors which ESWs find helpful or unhelpful for their mental health while experiencing work-related psychological distress, are explored; (5) Published in English and peer-reviewed.

No limits were applied to publication date or study location.

**Exclusion criteria:**

(1) Due to the unique nature of the traumatic events witnesses in this cohort,[44] studies investigating a military cohort will be excluded; (2) Volunteer ESWs were not included.

For peer review only

* Emergency service workers' (ESW) constitute of members of the emergency medical services (EMS), the fire service and the police force, and have been chosen based on the similar nature of the traumatic events these groups face. [2-4,6] The term 'psychological distress will be referred to according to Ridner's definition (see Appendix A).[45]

Search strategy

Systematic searches of the following four databases were conducted between January and March 2020: OVID MEDLINE, EMBASE, PsycINFO  and SCOPUS. The first three databases were searched together using the MEDLINE database. In order to avoid duplicates, the SCOPUS database was then searched with an additional filter to exclude MEDLINE results. Two reviewers (NA and RR) independently reviewed the 42 full text articles assessed for their eligibility criteria, with the aid of RefWorks reference management software. During this process, the two reviewers agreed to exclude 17 studies which did not meet the eligibility criteria. In cases of uncertainty surrounding eligibility criteria, there was a group discussion between all authors. One additional study was excluded on grounds of insufficient study


For peer review only - http://bmjopen.bmj.com/site/about/guidelines.xhtml

quality; this was agreed upon by all authors. (Figure 1)

To identify articles missed in the electronic database search, the following methods were also employed: 1) Using 'related article' feature (when available), 2) Searching the titles of included studies in google scholar for citation tracking purposes, 3) Manual searching of the references of relevant studies (reference mining).

Grey literature was searched during background research for context. Two combinations of search criteria (see appendix B) were entered into each database in order to locate relevant literature relating to help-seeking and mental health recovery. The search terms were developed during a process of trial and error using qualitative guidance,[46] other reviews in the field,[37,41] and virtual consultations with stakeholders and co-authors.

For peer review only

Quality appraisal

One reviewer (NA) assessed study quality using the CASP qualitative checklist.[47] Studies scoring less than five were further appraised independently by one of three reviewers (RR,

MB, JW) to determine whether they should be included in this review based on their conceptual richness, drawing upon five quality appraisal prompts as recommended by Dixon-Woods et al.[48] One study was removed during this approach.[49] In keeping with this approach, ‘signal’ (the relevance of papers to the review’s aims) was prioritised over ‘noise’ (*the inverse of methodological quality*).[48]

For peer review only

Data extraction

A customised data extraction template (See Appendix C) was created using qualitative data extraction guidance[46] and that of a similar review as a template.[41] In keeping with thematic synthesis approach,[38] first order constructs (direct participant quotations) as well as the author’s interpretations were extracted, in separate sections, to allow for a more comprehensive review.[46] Following Thomas and Harden,[38] all data relevant to the research aims were extracted onto templates by one researcher (NA).

Data synthesis:



For peer review only - http://bmjopen.bmj.com/site/about/guidelines.xhtml

The extracted text underwent line-by-line open coding, which allows new codes to emerge from the data, rather than imposing a pre-existing framework onto the extracted data.[38] Subsequently, first-level codes were assimilated according to their meanings, similarities and differences. The codes were then arranged in a ‘hierarchical tree structure’ during which some codes were renamed as new meanings were generated. For example, some quotations initially coded as ‘stigma’ were later agreed to more appropriately reflect ‘macho culture’ or ‘career concerns’, with ‘stigma and shame’ relating specifically to shaming practices. This inductive process results in ‘descriptive themes’. Two reviewers (NA and RR) performed the initial coding process, but grouping into descriptive themes and interpretation of the data involved discussion between all four authors. To enhance transparency, primary quotations used to construct the themes are supplied in Appendix D. In the final stage of thematic synthesis, the descriptive themes were used to address the review questions. This process involves ‘inferring’ the meaning behind the data; The final result of this process was the translation of the descriptive themes into implications for ESO wellbeing policy practice, which involved roundtable discussion between all four authors.

## Results


For peer review only - http://bmjopen.bmj.com/site/about/guidelines.xhtml

<<insert Figure 1 here>>

*Figure 1: Preferred Reporting Items for Systematic Reviews and Meta-Analyses (PRISMA) flow diagram.*[43]

Overview of study characteristics

24 studies were included in this review (Table 2). All but two of these studies[50,51] employed qualitative methodology only. For these two studies, only data from sections related to their qualitative methodology were extracted. The majority of studies (16 of 24) employed a sample of ambulance personnel[50-65] followed by police officers[66-69] and lastly firefighters.[70,71] The study participants of one study, in addition to ESWs, included participants who did not match the selection criteria[70], and two studies included a mix of different types of ESWs.[72,73] These studies were still eligible as the participant quotations were labelled with an occupational identifier to make it possible to differentiate between eligible and non-eligible participants. The objectives of studies varied widely in studies in terms of relevance to this review's aims. Ten of the 24 studies included data relevant to post-traumatic incident mental health recovery,[51-53,55-58,61,65,73] and one study included data relevant to barriers and

For peer review only

facilitators to mental health help-seeking.[72] The remaining 13 studies[50,54, 59,60,62-64,66-71] included data which was extracted for both of these outcomes.

**Study quality**:

Study quality varied significantly, with data collection, data analysis and discussion of findings being adequately explained in the majority of studies. All studies provided adequate research aims, although fewer than half sufficiently justified the research design. [51,53,55, 59, 60,65,66, 67,69,71,72] The lowest scoring domains were recruitment and demonstrating reflexivity. In few studies the researchers adequately justified their selection of participants[51,55,57,67,72,73] or critically examined their relationship with participants. [66,58,62,63,66,72,73] Four studies failed to present any ethical considerations. [50,51,64,70]

The full CASP appraisal table of all included studies is provided in online supplementary file 2. Following CASP appraisal, six studies[48,55,57,61,64,70] initially identified as weaker quality underwent further independent quality appraisal by one of three reviewers (RR, MB, JW), during which process one study was excluded.[49]

This qualitative review identified 14 descriptive themes, which are arranged in groups of higher order themes. In turn, these are grouped into one of the following two overarching



For peer review only - http://bmjopen.bmj.com/site/about/guidelines.xhtml

constructs: ‘factors contributing to mental health protectoin’ or ‘factors influencing mental health help-seeking behaviour’. Table 3 presents a summary of hierarchical thematic structure.

**Patient and public involvement**

No patient involved

**Table 2: Study characteristics**

| Title of study (Authors, year) | Quality ranking against CASP criteria | Participants | Study aim(s) | Country | Method of data collection | Analysis method |
|---|---|---|---|---|---|---|
| What makes an incident critical for ambulance workers? Emotional outcomes and implications for intervention. Halpern et al. 2009 | 5/9 | N=60 4 supervisors, 54 front-line ambulance workers | To characterize critical incidents as well as elicit suggestions for interventions | Canada | Focus groups/ individual interviews | Ethnographic content analysis |
| Police officers' experiences of supportive and unsupportive social interactions following traumatic incidents. Evans et al. 2013 | 6/9 | N=19 Police officers | (1) What are police officers’ experiences of supportive and unsupportive interactions following potentially traumatic incidents? (2) Do interactions differ on the basis of the context and source of support (i.e., at work with colleagues and supervisors, or outside of work with family and friends)? (3) How do supportive/unsupportive interactions facilitate/hinder the processing of traumatic incidents? | England | Semi-structured interviews | Thematic analysis |
| Interventions for critical incident stress in | 6/9 | N=60 4 supervisors, 54 front-line | To explore and describe Emergency Medical Technicians’ | Canada | Focus groups/ individual interviews | Ethnographic content analysis |



For peer review only - http://bmjopen.bmj.com/site/about/guidelines.xhtml

| | | | | | | |
|---|---|---|---|---|---|---|
| emergency medical services: a qualitative study Halpern et al. 2008 | | ambulance workers | (EMTs) experiences of critical incidents and views about potential interventions, in order to facilitate development of interventions that take into account EMS culture | | | |
| Barriers and Facilitators to Seeking Mental Health Care Among First Responders: "Removing the Darkness" Jones et al, 2020 | 9/9 | N=32 Twenty-five (78%) of the participants were active firefighters, 15 (47%) were certified EMTs, and 11 (34.4%) were certified EMTs/ paramedics. | To explore factors that influenced FRs' perceptions of mental health problems and engagement in MH services. | United states | Ethnographic individual interviews | Content analysis |
| Exposure to human tragedy, empathy, and trauma in ambulance paramedics. Regehr et al, 2002 | 6/9 | N=18 Paramedics | This mixed-methods study attempts to better understand factors that lead to higher levels of distress among paramedics within the theoretical framework of emotional and cognitive empathy. | Canada | Semi-structured interviews | Thematic analysis |
| The meaning of traumatic events as described by nurse sin ambulance service Jonsson et al. 2003 | 5/9 | N=362 240 EMTs, 122 registered nurses | The aim of this phenomenological study is to uncover the essence of traumatic events experienced by Swedish ambulance personnel. | Sweden | Written reports | Phenomenological analysis |
| Guilt, shame and need for a container: a study of post-traumatic stress among ambulance personnel Jonsson et al. 2004 | 4/9 | N=10 Ambulance nurses and ambulance technicians | The phenomenon approached in this study could be described as 'the way ambulance staff experience and handle traumatic events'. | Sweden | Individual interviews | Descriptive phenomenology |
| Situation Critical: High Demand, Low Control, and Low Support in Paramedic Organizations Regehr et al. 2007 | 7/9 | N=17 Paramedics | This mixed-methods study involving survey design and qualitative interviews seeks further to understand the factors related to these high levels of occupational stress. | Canada | Long interviews | Constant comparative method |
| Emergency Medical Services Provider Perspectives on Pediatric Calls: A Qualitative Study Jessica et al. 2019 | 8/9 | N=17 Paramedics and EMTs | This qualitative study was conducted to increase understanding about the difficulties of responding to pediatric calls and to obtain information about how organizations can better support EMS | United states | Focus groups | Directed content analysis |



For peer review only - http://bmjopen.bmj.com/site/about/guidelines.xhtml

| | | | | | | |
|---|---|---|---|---|---|---|
| | | | providers in managing potentially difficult calls. | | | |
| An assessment of the need of police officials for trauma intervention programmes - a qualitative approach. Boshoff et al. 2015 | 5/9 | N=40 Police officials | To conduct a qualitative situational analysis by exploring the experience and specific needs with regards to trauma and trauma intervention of police officials within the North-West Province's specialist units. | South Africa | Focus groups | Thematic analysis |
| A preliminary investigation of post-traumatic stress symptoms among firefighters Haslam et al. 2003 | 4/9 | N=31 11 firefighters, 8 station officers, 4 sub officers, 4 leading firefighters, 2 fire control officers, 2 area divisional officers | This preliminary study aimed to conduct an in-depth investigation of symptoms cited by fire service personnel and assess potential risk factors for mental health and PTSD. | England | Individual interviews | Thematic analysis |
| A qualitative study about experiences and emotions of emergency medical technicians and out-of-hospital emergency nurses after performing cardiopulmonary resuscitation resulting in death Fernández-Aedo et al. 2017 | 4/9 | N=13 7EMTs, 6 ambulance nurses | To explore the experiences, emotions and coping skills among emergency medical technicians and emergency nurses after performing out-of-hospital cardiopulmonary resuscitation maneuvers resulting in death. | Spain | Semi-structured individual interviews and focus groups | Thematic analysis |
| Experiences of and actions towards worries among ambulance nurses in their professional life: A critical incident study Svensson et al. 2008 | 7/9 | N=25 Ambulance nurses | The purpose of this study was to describe critical incidents in which ambulance nurses experience worry in their professional life and the actions they take in order to prevent and cope with it. | Sweden | Semi-structured individual interviews | Structural analysis/Critical incident technique |
| Exploring the nature of resilience in paramedic practice: A psycho-social study Clompus et al. 2016 | 5/9 | N=7 Paramedics or EMTs | The aim of this study was to explore the question of how paramedics 'survive' their work within the current healthcare climate. | England | Biographical narrative interviews and semi-structured interview with all participants | Thematic analysis |
| First response emergency care – experiences described by firefighters Abelsson et al. 2019 | 5/9 | N=35 Firefighters | The purpose of this paper is to describe firefighters' experiences of first response emergency care | Sweden | Group interviews | Interpretive qualitative content analysis |


For peer review only - http://bmjopen.bmj.com/site/about/guidelines.xhtml

| | | | | | | |
|---|---|---|---|---|---|---|
| Paramedics' experiences with death notification: a qualitative study Douglas et al. 2013 | 5/9 | N=28 Paramedics | To explore paramedics' experiences and coping strategies with death notification in the field. | Canada | Focus groups | Inductive thematic analysis |
| Peer-support: a coping strategy for nurses working at the Emergency Ambulance Service Carvello et al. 2019 | 4/9 | N=14 Ambulance nurses | The aim of the study is to explore the experiences, the opinions and feelings of emergency medical service nursing staff in relation to the use of the peer supporting model. | Italy | Semi-structured interviews | Not made explicit |
| Experiences among firefighters and police officers of responding to out-of hospital cardiac arrest in a dual dispatch programme in Sweden: an interview study Hasselqvist-Ax et al. 2019 | 8/9 | N=22 10 police officers, 12 firefighters | The aim of this interview study was to explore firefighters' and police officers' experiences of saving lives in OHCA in a dual dispatch programme. | Sweden | Individual interviews | Critical incident technique |
| Working in prehospital emergency contexts: Stress, coping and support from the perspective of ambulance personnel Oliveira et al. 2019 | 8/9 | N=14 Ambulance personnel | The purpose of this paper is to explore, from this group perspective, sources of stress, coping strategies and support measures | Portugal | Semi-structured interviews | Thematic analysis |
| Exploring paramedic communication and emotional expression in the workplace after responding to emergency calls Drewitz-Chesney et al. 2019 | 7/9 | N=8 Paramedics | The study aim was to learn about peer communication and emotional expression between paramedics in the workplace, after they respond to calls. | Canada | Semi-structured individual interviews | Constructivist grounded theory |
| Mental Health in the UK Police Force: a Qualitative Investigation into the Stigma with Mental Illness Edwards et al. 2020 | 6/9 | N=5 Police officers | The study aims to explore institutional negativity and stigma in the police force towards mental ill health | United Kingdom | Semi-structured individual interviews | Thematic analysis |

For peer review only

| | | | | | | |
|---|---|---|---|---|---|---|
| Living in Critical Times: The Impact of Critical Incidents on Frontline Ambulance Personnel: A Qualitative Perspective Gallagher et al. 2007 | 2/9 | N=27<br>21 EMTs, 6 EMCs | The principal aim of this second stage of the study was to ascertain, using qualitative methods, the impact of CIs on frontline staff by allowing them to tell their own stories. | Ireland | Individual interviews | Thematic analysis |
| *Police officers, mental (ill-) health and spoiled identity* Bullock et al. 2018 | 5/9 | N=59<br>52 police officers,<br>2 police staff,<br>4 PCSOs,<br>1 special constable | The processes through which some police officers with mental ill-health experience stigmatization. | England and Wales | Phone interviews | Thematic analysis |
| 'You see a baby die and you're not fine:' a case study of stress and coping strategies in volunteer emergency medical technicians Folwell et al. 2018 | 6/9 | N=25<br>EMTs | This study explores the lived experience of ESP involved in unsuccessful pediatric resuscitation efforts and how this experience affects them professionally and personally. | United States | Individual interviews | Constant comparative analysis |

For peer review only

**Table 3: Summary of Themes**

| Domain | Higher level theme | Descriptive themes |
|---|---|---|
| Factors contributing to mental health protection | Organisational | -Time out/Downtime<br>-Supervisor<br>-Official peer support network |



For peer review only - http://bmjopen.bmj.com/site/about/guidelines.xhtml

| | | |
|---|---|---|
| | Informal support | -Colleagues and family<br>-Regular partner<br>-Reassurance and validation |
| Factors influencing help-seeking behaviour | Nature of intervention delivery | -Mandatory vs non-mandatory<br>-Shared experiences with intervention provider |
| | Stigma as a help-seeking barrier | -Macho culture<br>-Stigma and shame<br>-Career concerns<br>-Confidentiality concerns |
| | Mental health literacy | -Emotional awareness<br>-Education and stigma |

## 1. Factors contributing to mental health protection

The following themes describe factors which participants attribute to having a direct influence on their mental health protection or recovery following traumatic incident exposure.


For peer review only - http://bmjopen.bmj.com/site/about/guidelines.xhtml

## Organisational

Three themes were identified as being directly linked with systems put in place by the organisations employing ESWs, and represent opportunities for mental health support following trauma exposure over which ESOs have complete control.

For peer review only

*Time-out/Downtime period*

A ‘time-out’ or ‘downtime’ period refers to a period of time following a stressful call in which ESWs are temporarily placed off duty, the availability of which was inconsistent across the studies. ESWs working in organisations in which they were offered downtime by their supervisors following certain calls reported that these breaks, ranging from 30 minutes to two hours,[53,56] were essential in order to allow them to ‘decompress’ in preparation for the remainder of the shift.[53,54] ESWs found particular comfort in casual conversation with their colleagues during this time, which did not necessarily relate to the previous call.[53] During such discussion, humour could be employed by the group as a method of distraction and off-loading.[59] While the majority of ESWs preferred to be in the company of colleagues during



For peer review only - http://bmjopen.bmj.com/site/about/guidelines.xhtml

this time,[55,59,60,63] some individuals chose to make use of organisation-provided exercise equipment in order to de-stress.[65]

When time-out opportunities were unavailable, ESWs describe rushing into the next call without having psychologically processed the previous call.[51] In such circumstances, paramedics reported difficulty giving their full attention to the next call, limiting their ability to provide life-saving care.[64] Contrary to the above findings, one ambulance worker preferred to be dispatched to another call immediately following a stressful call, due to the distraction this provided.[53]

*Supervisor*

ESWs have supervisors/line managers, whose roles include acting as a point of contact after a traumatic incident. In an ambulance setting, paramedics were appreciative of the ‘genuine concern’ shown by their managers or supervisors following a traumatic call.[52] Concern was commonly expressed by asking paramedics how they were feeling, and providing them with an opportunity to talk.[52,53] Not all ESWs want to be approached by their supervisor immediately following the call, as illustrated by the following quote from a paramedic:



For peer review only - http://bmjopen.bmj.com/site/about/guidelines.xhtml

*'I don't want you to come up and get in my face and say, are you okay? Just leave me alone. Okay. Ask me in a couple of days, am I okay with the call, sort of thing.'*[53]

For peer review only

Occasionally supervisors were responsible for taking an ambulance crew off duty after a call.[53] Even if downtime opportunities were available upon individual request, paramedics described not making use of the opportunity unless suggested or requested by the supervisor.[60] Conversely, a supervisor's influence may also dissuade paramedics from requesting temporary downtime, especially for newer paramedics who were fearful of any repercussions linked to the perceived inability to cope.[60]

Paramedics described unsupportive supervisor responses, such as not recognising the traumatising effect of an incident, applying disciplinary pressure after complicated calls,[51] or showing a lack of concern for paramedics' mental wellbeing.[53,62]

*Official peer support network*



For peer review only - http://bmjopen.bmj.com/site/about/guidelines.xhtml

The majority of ambulance nurses taking part in one study were in favour of the existence of peer supporters within their organisation; who the nurses described as being able to 'understand', due to their common experiences.[61] Despite the apparent popularity of the service in this organisation, peer support networks in other organisations were rarely used,[59,64] with defusing occurring 'naturally within the halls' instead.[59] Participants in two studies expressed a hesitancy to make use of peer support opportunities for fear of being judged by colleagues as 'weak'.[61,64] Other concerns centred around the competence of peer supporters, their ability to maintain confidentliality,[61,64] and fear of overwhelming the colleagues delivering the support.[61]

**Informal support**

In contrast to organisational factors, these three themes describe how informal social factors influence ESWs' mental health protection and recovery. While discussion with colleagues is frequently discussed, this represents informal discussion and is therefore beyond the direct control of ESOs.

*Colleagues and family*



For peer review only - http://bmjopen.bmj.com/site/about/guidelines.xhtml

ESWs reported that they found it useful to talk with someone in an informal manner.[53,54,55,57,59,60,61,63,64,66,68,70,71] One firefighter described a need to 'vent a backpack', which fills up after each call;[71] suppression could be harmful in the long term as mental health conditions remain unrecognised.[68] The main providers of such informal support were family members and work colleagues, but there were mixed findings in terms of preferences for support.

Many ESWs reported turning to their family members as a primary source of emotional support following difficult calls,[66,53,72,50,56,70,59] who were capable of 'selfless listening' without offering judgement,[66] and with whom ESWs feel more comfortable sharing emotional vulnerability with family members than with colleagues.[53,66] However, a number of ESWs reported avoiding talking to their family members about stressful calls out of a wish to protect them from the trauma they experienced,[56,58,59,66,70-72] although this did not apply to family members with a first responder/healthcare background, who were judged to be able to 'understand' ESWs' traumatic experiences.[57-59] For similar reasons, ESWs were willing to talk to certain colleagues about traumatic calls; The informal sharing of vulnerability was reserved for colleagues with whom ESWs shared a bond of trust[57,58,63,66] and for those more likely to empathise and understand the emotional impact of the event.[50,53,55-58] Sharing experiences with trusted colleagues provided an opportunity for reflection and to hear


For peer review only - http://bmjopen.bmj.com/site/about/guidelines.xhtml

different interpretations of the event. The risks of disclosure included reliving distressing events, and potential having feelings invalidated when partners felt differently about the event.[58]

*Regular partner*

ESWs described how having a regular work partner helped their ability to process traumatic events encountered on the job.[51,53,63,72]A trusting relationship between partners facilitated comfortable sharing of vulnerability following traumatic calls. [53,63] Having shared the experience, partners could emotionally support colleagues by allowing them to talk about the call and provide reassurance.[51,72] Having a regular partner could however be a negative influence in the case of an unsympathetic relationship, such as partners who respond insensitively to any disclosure of vulnerability.[63] Due to the potential stigma arising from the disclosure of vulnerability within earshot of colleagues, the process of 'defusing' between partners, following a call, commonly takes place within the private space of the ambulance, when returning to base and while awaiting the next call. [63]

*Reassurance and validation*

For peer review only

Reassurance, provided by colleagues indicating that they would have acted in the same way[52,54,58] or by receiving praise for their actions from their supervisor,[53] were valued by ESWs following traumatic incidents, especially those involving fatalities.[53] Reviewing the technical aspects of calls with other ESWs provided reassurance that the ‘final outcome’ was unavoidable.[71] In cases of suicide, learning about the preceding circumstances could provide closure for some ESWs.[53] Following fatal accidents, paramedics also described needing to visit family members in hospital, or to attend funerals.[52]

## 2. Factors influencing help-seeking behaviour

The following themes reflect the barriers and facilitators to help-seeking following occupational traumatic exposure.

**Nature of intervention delivery**

Two themes elaborate on how the method in which a formal intervention is offered can influence attitudes towards engagement.



For peer review only - http://bmjopen.bmj.com/site/about/guidelines.xhtml

*Mandatory vs non-mandatory*

The decision to employ optional or mandatory organisational mental health support for ESWs following traumatic calls was raised in several of the studies and often depended on the timing of delivery following the incident. Some participants resisted mandatory organisational mental health support following traumatic calls; police officers expressed a need to '*feel in control of the decision to talk'* due to the stigma surrounding any disclosure of vulnerability.[66] Mandatory interventions for ESWs before they are ready to talk could lead to a rejection of the intervention,[53,60] as illustrated by the following paramedic quote:

> *'My emotions are none of your business and if I wanted to share my emotions with you, I'm going to share [them] with someone I trust…*[60]

Others however believed mandatory interventions would reduce stigma associated with their use,[60,66] and prevent delays to help-seeking due to the stigma associated with disclosing vulnerability.[53] Police officers who were initially reluctant to participate felt 'calmer' and expressed gratitude after attending a mandatory counselling service.[66] EMS staff in one study

For peer review only

suggested limiting mandatory support to certain types of incident, such as those involving children.[53]

*Shared experiences with intervention provider*

Therapists with a background in the emergency services or the military, or trained peers, were preferred by ESWs,[72] because of the belief that they are more likely to 'understand' their problems and experiences.[60] This finding was observed across the emergency services:

> *'Many [participants] also approved of a provider that "knew the job," either working with multiple FRs in the past, or even as a family member.'*[72]

**Stigma as a help-seeking barrier**

*'Macho' culture*

Stigma associated with the disclosure of emotional vulnerability related to traumatic calls, and mental health issues.[50,53,54,59,60,62,63,64,66,68,70] was identified across the emergency services. A


For peer review only - http://bmjopen.bmj.com/site/about/guidelines.xhtml

'macho' attitude and culture acted as a key barrier to disclosure where there was an expectation to *'deal with it'*.[63,68] Disclosure of vulnerability in such a culture was perceived as a weakness and responders were viewed as unable to cope with the demands of the job as first responder.[53,59,60,63,66,69,70] Revealing one's feelings was perceived to be emasculating and prevented ESWs from talking about their feelings and seeking support,[60,66,70] as demonstrated by this quote from a police officer:

'I *think there's a real element of machismo and masculinity in the police force and it's a bit, sort of a faux pas to admit that things have really affected you ... If I'd have come out and said ''ah you know, that really affected me badly, let's go and sit down and have a cup of tea and talk about it'' I think you're straying into pink and fluffy territory there ... saying 'that made me feel sad' is a bit too far'.*[66]

Stigmatising attitudes held by senior organisational members were influential as they prevented ESWs from contacting their supervisors to seek support.[66,69] Discussing stress in this culture was described as 'taboo'[64] so ESWs reported often avoiding asking their colleagues about their emotions following traumatic calls;[60,62] police officers described how

For peer review only



For peer review only - http://bmjopen.bmj.com/site/about/guidelines.xhtml

'tough' colleagues working in such a culture have died by suicide.[68] Some organisations described a contrasting culture in which openness about emotional vulnerability was regarded as a strength.[71]

Two studies identified a connection between elements of the 'macho' culture described, and the gender of ESWs. Swedish ambulance nurses reported that organisations where there was a higher proportion of women fostered a culture of openness with respect to sharing vulnerability.[50] Of a small sample of seven paramedics, Clompus et al. noted that female participants were more likely to have made use of formal mental health support mechanisms.[59] The authors attribute this to *'masculinised paramedic culture'*.[59]

*Stigma and shame*

This review identified that the fear of being shamed by being labelled as '*malingerers*'[68] or '*the lazy and the lame*'[69] resulted in presenteeism when officers remained on active duty although mentally unwell. Another aspect of mental health stigma is the belief that affected individuals are less competent in their responsibilities as an ESW, as well as being unreliable.[69] Police



For peer review only - http://bmjopen.bmj.com/site/about/guidelines.xhtml

officers described a common belief that *'you're on your own'* working a shift with a colleague who has been open about the emotional impact of traumatic calls.[66] ESWs who have been open about their mental health diagnoses describe being labelled as '*mad*'[67] or a '*crazy guy*'[72]; Such attitudes may result in shame and the avoidance of help-seeking.

*Career concerns*

Four studies report that police officers delay help-seeking for mental illness due to concerns about the perceived impact of disclosure on their careers [66-69] Officers believed that being labelled with a mental health condition would obstruct career progression.[67-69] Officers feared being removed from 'public-facing operational roles', and/or feared a reduction in pay which related to being removed from frontline duities.[68] Fear of involuntary dismissal due to disclosure and help-seeking was also reported by study participants from the fire service.[70]

*Confidentiality concerns*

Concerns regarding confidentiality were a barrier for ESWs to formal and informal help-seeking behaviour. Formal support services were viewed with suspicion by police officers [67] while other officers felt they might be monitored or labelled as 'weak' if they sought referral

For peer review only

to wellbeing services.[66] Concerns about confidentiality were also raised by firefighters, who therefore requested preference for an anonymous counselling service outside of the brigade.[70] In the emergency medical services, EMTs expressed concerns about loss of confidentiality through the organisation-provided peer support network;[64] similar concerns were expressed by EMTs towards critical incident stress debriefing (CISD).[65] There are also perceived risks of confidentiality breaches in informal settings, as described by one paramedic:

> *I think that the stigma is you have to be very careful who you tell that it bothered you or you might get judged as weak or you might get fired.*[72]

Confidentiality concerns could also indirectly influence help-seeking; ambulance supervisors wishing to put their crew on a time-out after a traumatic call can be dissuaded by knowing this information could become public knowledge among the dispatchers.[53]

**Mental health literacy**



For peer review only - http://bmjopen.bmj.com/site/about/guidelines.xhtml

*Emotional awareness*

Participants and authors of the included studies recognised a need for more training and education about mental health related issues for ESWs and family members and supervisors, who may be in a better position to detect behavioural changes and therefore facilitate help-seeking.[52,53,64,72] Such education should focus on increasing ESWs' ability to detect emotional changes within themselves, which could facilitate help-seeking behaviour.[52,64,64,67,72] ESWs expressed a desire to be informed about the types of emotions which could be triggered by work-related traumatic incidents, [52,64] which may reduce shame associated with help-seeking.[72] Studies revealed that an inability to recognise milder mental health symptoms acted as a barrier to help-seeking, with participants writing them off as being 'grumpy',[68] and not recognising and admitting to emotional distress.[52] Additionally, participants were sometimes unaware of the support services available to them,[63,67,72] and were unaware of the benefits of seeking help.[72]

*Education and Stigma*



For peer review only - http://bmjopen.bmj.com/site/about/guidelines.xhtml

While stigma may indirectly change through improving general mental health awareness, authors also emphasised the value of education to reduce organisational stigma.[53] ESWs recommend such education to be delivered regularly in ‘brief and efficient’ classes of small groups by a peer from outside of the organisation.[72] Having an awareness of work-related mental health problems among colleagues appeared to be an important facilitator of help-seeking.[69,72] Experienced ESWs were regarded as being influential by giving permission to other responders to ‘open up’.[71] Police officers in one organisation therefore advocated for mental health ‘champions’; colleagues, preferably leaders, who could model vulnerability by openly disclosing their mental illness and work-related distress .[69]

For peer review only

## Discussion

This review synthesised 24 primary studies investigating ESW attitudes to help-seeking and mental health protection and recovery following trauma exposure. The synthesis generated 14 themes relating to ‘factors contributing to mental health protection after traumatic incident exposure’ and ‘factors influencing mental health help-seeking behaviour’. Despite being grouped separately for increased clarity, these overarching constructs are interconnected.


For peer review only - http://bmjopen.bmj.com/site/about/guidelines.xhtml

Both constructs explore the influence that senior organisational members have on the mental health on ESWs within their organisation. The influence could be positive, such as delivering educational sessions regarding organisational mental health stigma. Contrary to this, ESWs could be discouraged from contacting their supervising officer to seek mental health support, or request temporary downtime, depending on the supervisors' perceived attitude to such issues. Another interconnecting area relates to the themes of 'macho culture' and 'stigma and shame', as these stigmatising attitudes contribute towards a hesitancy to use official peer support networks.

Help-seeking: culture/stigma

The findings of this review support quantitative findings that the fear of a breach in confidentiality in attending mental health services, and the concerns about negative career repercussions, pose significant stigma-related barriers to help-seeking for ESWs.[37] This is important as any delays in help-seeking can compound or exacerbate mental ill health.[74,75] Concerns raised by ESW responders about the impact of mental health disclosure on career progression, professional identity and competence have also been found in military personnel.[37,41,66,76,77]



For peer review only - http://bmjopen.bmj.com/site/about/guidelines.xhtml

Expressing emotional vulnerability, being labelled with a mental health condition and seeking help was equated with a perception of weakness, which contributed to a ‘macho’ culture. Similar masculinised social norms have been described in the military, [23,77] which is male dominated with high rates of mental health stigma.[37] There are, however, many other similarities between the two settings which are likely to be influential, such as ‘norms and values that place a premium on self-reliance in the face of obstacles’.[37] Furthermore, these findings may reflect evidence that females are more likely to seek help about their mental health,[78] and correlates with the literature suggesting that males in male-dominated professions display reduced mental health related help-seeking behaviours,[79] and increased suicide rates.[80] Notably, females had a reduced risk of suicide in male-dominated professions, but a slightly increased risk in female-dominated professions,[80] highlighting the complexity of using occupational gender ratios to predict mental health help-seeking behaviours and mental health outcomes. Occupations dominated by hyper-masculine stereotypes may also disadvantage men who do not identify with these values, therefore discouraging the very demographic who would challenge them.[81] Recruiting a more diverse, emotionally literate and aware workforce, with more women may challenge macho work cultures which prevent ESWs from disclosing their vulnerability.[81]

For peer review only

This review's finding of anticipated stigma acting as a help-seeking barrier, is supported by the findings from a systematic review which found that *'stigma can potentially lead to delayed presentation in mental health care'* for ESWs,[37] and as well as negatively influencing help-seeking in civilian populations.[76] These findings have also been demonstrated in a qualitative review of a military setting.[41]

The findings of this review in terms of attitudes to mental ill health are consistent with Goffman who argued that stigma is defined in and enacted through socially constructed 'norms'. The 'norms' of what is stigmatising and what isn't are socially constructed within a large variety of contexts and has the potential to 'shift'.[82] An effective way of challenging stigma associated with mental illness and changing negative perceptions is through a strategy of increasing contact .[23,83-85] In order to effectively counter stigmatising attitudes towards mental health, contact should be delivered in the context of the following five factors:[85] equal status, the opportunity for individuals to get to know each other, information which challenges negative stereotypes, active co-operation and pursuit of a mutual goal. Such approaches and factors could be employed to tackle the stigma and culture which prevent



For peer review only - http://bmjopen.bmj.com/site/about/guidelines.xhtml

ESWs from seeking help. Elements of these factors can be recognised in organisational strategies such as Mental Health Champions and Schwartz rounds (see below).

**Help-seeking: Education**

This review identifies a demand for improving the mental health literacy of ESWs and describes the type of education ESWs believe would be appropriate. Mental health education has been shown to be effective at changing attitudes towards mental health disorders when aimed at large populations, smaller at-risk groups or at the individual level.[86,87] Anti-stigma education was introduced in UK emergency services as part of the 'Blue Light Programme',[7] and demonstrated that achieving anti-stigma change at the employee level requires sustained education efforts over a number of years.[7]

The literature suggests that certain types of events, such as paediatric fatalities, events involving multiple casualties or suicides, have a higher traumatising potential than others.[50,70,88] The evidence suggests however that the process of traumatisation among ESWs is highly individual, relating to 'personal history, situation, and perspective' of the exposed individual,[65] and ESWs can experience trauma in different ways depending on how they contextualise the victim. [50] Questionnaires taking into account individual factors, such as the

For peer review only

ESW's 'state of mind' preceding the event, are therefore a useful resource for predicting peritraumatic distress in exposed individuals.[89]

**Mental health protection: Social support and downtime**

Review participants identified the importance of social support following exposure to traumatic incidents, in consistence with the wider literature. [25,41,90,91] Downtime was positively valued by ESWs who had experienced it, yet is not commonly granted to ESWs in practice.[91,92] The types of mental health outcome affected by post-incident downtime and extent of its influence is disputed. Carlier et al. identified 'insufficient time allowed by the employer to resolve the trauma' was correlated with higher PTSD scores in police officers three months post-trauma, but not at 12 months.[93] Having insufficient recovery time following traumatic calls has been correlated with higher emotional exhaustion[94] and psychological distress.[95] These results are in contrast with the findings of a cross-sectional quantitative survey of the psychological consequences of downtime in 217 ambulance workers, which revealed increasing periods of downtime, up to and including one day, to be



For peer review only - http://bmjopen.bmj.com/site/about/guidelines.xhtml

significantly associated with lower depression scores, but not with symptoms of 'post-traumatic stress, burnout and stress-related physical symptoms'.[96]

**Application of review findings to organisational interventions, policy and research**

Our findings have implications for three organisational interventions which are in use or available for use within the emergency service organisations. These interventions have particular relevance in the context of this review due to their shared potential to alter organisational mental health stigma, which this review identified as a barrier to help-seeking.

1) *Trauma risk management (TRiM)*

Consistent with qualitative findings of navy personnel regarding the implementation of TRiM, [97] this review identified a perception among ESWs that peer-support programs are relevant and suitable for their needs, as well as concerns regarding confidentiality and competence of practitioners of peer support programs. Peer supporters are not however regarded as possessing the same professional



For peer review only - http://bmjopen.bmj.com/site/about/guidelines.xhtml

competence/credibility of a professional mental health professional. Of note, while review participants expressed concerns about being judged for being perceived as ‘weak’ by peers in a peer-support system, such a concern was not detected among naval officers towards TRiM,[97] despite widespread stigma towards help-seeking and mental health issues which exists in the military.[41]

2) *Mental Health Champions*

This review identified that awareness of colleagues’ mental health challenges could be an important facilitator for help-seeking. The ‘Blue light champions’ role[98] therefore appears to be a potentially effective method of improving attitudes, although quantitative evaluation linking these roles with ESW mental health outcomes and culture change is lacking. Staff satisfaction surveys of these roles reveal a lack of support from management and not having sufficient time available to dedicate to the role.[99]

3) *Schwartz Rounds*



For peer review only - http://bmjopen.bmj.com/site/about/guidelines.xhtml

In terms of challenging organisational culture which prohibits help-seeking due to stigma, Schwartz Rounds offer staff the opportunity to disclose their vulnerability whilst fostering a connectedness to other staff. However, Schwartz Rounds have not been evaluated and it is unclear whether they reduce stigma, facilitate help-seeking behaviour, or alter mental health outcomes. Quantitative evidence describing the effectiveness of Schwartz on the above outcomes would be useful.

For peer review only

**A note about Covid**

This is the first review to qualitatively synthesis the barriers and facilitators to help-seeking in the emergency services, and frontline staff's experience of mental health protection and recovery following traumatic incident exposure. The search was conducted in March 2020, before the emergence of qualitative literature relating to the Covid pandemic, offering a summary of evidence preceding this significant historical benchmark. Qualitative literature relating to ESW mental health in the context of Covid-19 necessitate analysis in their own right, due to the distinct psychological stressors brought on by a pandemic, such as fear of infecting family members.[100] Healthcare workers experience different mental health pressures


For peer review only - http://bmjopen.bmj.com/site/about/guidelines.xhtml

in this unique context,[101] and should therefore be studied in a separate context to police and fire workers.

## Implications for further research to help inform policy

Following traumatic calls, ESWs will likely benefit from having a period of mental recovery time when they can access informal support from their colleagues. ESOs should be aware of the therapeutic effects of informal support in this post-incident setting, and facilitate its availability. It should be considered that despite recognising its value, ESWs are unlikely to request downtime themselves, instead relying on it being imposed/offered by supervisors. It may also be useful for supervisors to consider that the manner in which they approach ESWs for welfare concerns, as demonstrated by this review, in addition to their overall attitude regarding mental health, have significant implications for the help-seeking behaviours of ESWs. It may be important to consider that ESWs report trusting professional relationships, such as regular work-partners, as being psychologically protective against occupational trauma experiences, providing vulnerability can be comfortably

For peer review only

shared. ESWs may benefit from education enhancing their ability to recognise pathological emotional changes secondary to traumatic incident exposure both in themselves and colleagues, and to able to appreciate when it is appropriate to access formal organisational resources and the potential benefit of different types of interventions for trauma exposed ESWs. Additionally, communicating to ESWs that the objective characteristics of an event cannot invalidate their experience of being traumatised, could enhance help-seeking by normalising symptoms of mental ill health. ESOs may wish to consider that mental health champion-type roles, providing staff in such roles are adequately supported, are regarded by ESWs as valuable tools for challenging mental health stigma. This review's findings may provide insights into how engagement between ESWs and official peer support networks can be improved, by focussing on cultural anti-stigma change and by targeting specific barriers to engagement as identified in the results section. Quick fix solutions to the cultural change necessary to increase help-seeking in ESOs should not be prioritised over meaningful sustained efforts to improve mental health literacy.



For peer review only - http://bmjopen.bmj.com/site/about/guidelines.xhtml

# Conclusion

This review explores the role of mental health stigma on help-seeking behaviours of trauma-exposed ESWs, who often access informal defusing opportunities in preference to formal interventions. The barriers and facilitators to help-seeking identified by this review may assist emergency medical organisations in improving engagement amongst their staff with organisational wellbeing interventions, in keeping with the organisation's responsibility to dismantle barriers to help-seeking and destigmatising mental ill health and vulnerability through role modelling and supportive leadership. Findings related to the experiences and attitudes of staff regarding mental health protection and recovery should be considered when implementing occupational support programmes.

For peer review only



For peer review only - http://bmjopen.bmj.com/site/about/guidelines.xhtml

For peer review only


For peer review only - http://bmjopen.bmj.com/site/about/guidelines.xhtml

## Footnotes

**Acknowledgements:** This work was initially written as a dissertation. We would like to thank Justin Waring for being a valued source of support throughout the dissertation. Special thanks to Anna Parry for her vital contributions in the early stages of the project, and involving key ambulance stakeholders during the process of developing the project aims. We would also like to thank Marcus Auth for his assistance provided in the form of advice regarding structure of the paper, and formulating the abstract.

**Contributors:** NA is the main author involved in all stages of the design, analysis and drafting and writing of the paper. RR was involved in the conception of the project's scope, design,



For peer review only - http://bmjopen.bmj.com/site/about/guidelines.xhtml

and interpretation of results, providing important supervision and mentorship throughout. RR, MB and JW assisted with drafting the article and revising it critically for important intellectual content, providing methodological advice, quality appraisal by independently appraising lower CASP scoring studies, and final approval of the version to be published. All authors have approved the final version of the paper ahead of publication.

**Funding:** This research received no specific grant from any funding agency in the public, commercial or not-for-profit sectors.

**Competing interests:** None declared.

**Provenance and peer review:** Not commissioned; externally peer reviewed.

**Data sharing statement:** No additional data are available.

**Figure legend:** Supplementary Figure 1. Preferred Reporting Items for Systematic Reviews and Meta-Analyses (PRISMA) flow diagram.

only

## References


1. Jones S. Describing the Mental Health Profile of First Responders: A Systematic Review. J Am Psychiatr Nurses Assoc 2017 May;23(3):200-214.
2. Lima Ed P, Assuncao AA. Prevalence and factors associated with Posttraumatic Stress Disorder (PTSD) in emergency workers: a systematic literature review. Rev bras epidemiol 2011 Jun;14(2):217-230.
3. Berger W, Coutinho E, Figueira I, Marques-Portella C, Luz M, Neylan T, et al. Rescuers at risk: a systematic review and meta-regression analysis of the worldwide current prevalence and correlates of PTSD in rescue workers. Soc Psychiatry Psychiatr Epidemiol 1206;47(6):1001-1011
4. Stanley IH, Hom MA, Joiner TE. A systematic review of suicidal thoughts and behaviors among police officers, firefighters, EMTs, and paramedics. Clin Psychol Rev 1603;44:25-44.
5. Petrie K, Milligan-Saville J, Gayed A, Deady M, Phelps A, Dell L, et al. Prevalence of PTSD and common mental disorders amongst ambulance personnel: a systematic review and meta-analysis. Soc Psychiatry Psychiatr Epidemiol 2018 Sep;53(9):897-909.
6. Substance Abuse and Mental Health Services Administration. First Responders: Behavioral Health Concerns, Emergency Response, and Trauma. [internet]. 2018 May. [cited 2019 Oct 14]. Available from: https://www.samhsa.gov/sites/default/files/dtac/supplementalresearchbulletin-firstresponders-may2018.pdf


For peer review only

7. Mind. Wellbeing and mental health support in the emergency services. [internet]. 2019. [cited 2019 November 5]. Available from: https://www.mind.org.uk/media-a/4572/20046_mind-blue-light-programme-legacy-report-v12_online.pdf
8. Mitchell, J.T., & Everly, G.S. (1995). Critical Incident Stress debriefings: An operations manual for the prevention of traumatic stress among emergency service and disaster workers (2nd ed. revised). Ellicott City, MD: Chevron Publishing Company.
9. Jakupcak, M., Cook, J., Imel, Z., Fontana, A., Rosenheck, R., & McFall, M. Posttraumatic stress disorder as a risk factor for suicidal ideation in Iraq and Afghanistan War veterans. Journal Of Traumatic Stress 2009 July;22(4), 303-306. doi: 10.1002/jts.20423
10. Tarrier N, Gregg L. Suicide risk in civilian PTSD patients. Social Psychiatry and Psychiatric Epidemiology. 2004;39(8).
11. Maia DB, Marmar CR, Metzler T, Nóbrega A, Berger W, Mendlowicz MV, et al. Post-traumatic stress symptoms in an elite unit of Brazilian police officers: Prevalence and impact on psychosocial functioning and on physical and mental health. J Affect Disord 2007;97(1):241-245.
12. Case R, Alabakis J, Bowles K-A, Smith K: High-risk occupations—Suicide: an Evidence Check rapid review brokered by the Sax Institute (www.saxinstitute.org.au) for the NSW Ministry of Health, 2020.
13. Mind. The case for support. [internet]. 2017. [cited 2019 November 6]. Available from: https://www.mind.org.uk/media-a/4510/blp-blueprint_case-for-support.pdf
14. Antony J, Brar R, Khan P, Ghassemi M, Nincic V, Sharpe J et al. Interventions for the prevention and management of occupational stress injury in first responders: a rapid overview of reviews. Systematic Reviews. 2020;9(1).
15. Smith A, Roberts K. Interventions for post-traumatic stress disorder and psychological distress in emergency ambulance personnel: a review of the literature. Emergency Medicine Journal 2003 /01/01;20(1):75-78.
16. Carlier IVE, Lamberts RD, Van Uchelen AJ, Gersons BPR. Disaster-related post-traumatic stress in police officers: a field study of the impact of debriefing. Stress Med 1998 9807 9807;14(3):143-148.
17. Sattler DN, Boyd B, Kirsch J. Trauma-exposed Firefighters: Relationships among Posttraumatic Growth, Posttraumatic Stress, Resource Availability, Coping and Critical Incident Stress Debriefing Experience. Stress Health 2014;30(5):356-365.



For peer review only - http://bmjopen.bmj.com/site/about/guidelines.xhtml

18. Tuckey MR. Group critical incident stress debriefing with emergency services personnel: a randomized controlled trial. Anxiety, Stress & Coping 2014;27(1):38-55.
19. Regel S. Post-trauma support in the workplace: the current status and practice of critical incident stress management (CISM) and psychological debriefing (PD) within organizations in the UK. Occupational Medicine 2007 709 0;57(6):411-416.
20. Rose S, Bisson J, Churchill R, Wessely S. Psychological debriefing for preventing post traumatic stress disorder (PTSD). Cochrane Database of Systematic Reviews. 2002;.
21. Whybrow D, Jones N, Greenberg N. Promoting organizational well-being: a comprehensive review of Trauma Risk Management. Occup Med (Lond) 2015 /06/01;65(4):331-336.
22. Hunt E, Jones N, Hastings V, Greenberg N. TRiM: an organizational response to traumatic events in Cumbria Constabulary. Occup Med (Lond) 2013 /12/01;63(8):549-555.
23. Gould M, Greenberg N, Hetherton J. Stigma and the military: Evaluation of a PTSD psychoeducational program. Journal of Traumatic Stress 2007;20(4):505-515.
24. Jones N, Burdett H, Green K, Greenberg N. Trauma Risk Management (TRiM): Promoting Help Seeking for Mental Health Problems Among Combat-Exposed U.K. Military Personnel. *Psychiatry*. 2017;80(3):236-251. doi:10.1080/00332747.2017.1286894
25. Shakespeare-Finch J, Rees A, Armstrong D. Social Support, Self-efficacy, Trauma and Well-Being in Emergency Medical Dispatchers. Soc Indic Res 1509;123(2):549-565.
26. Greenberg N. A cluster randomized controlled trial to determine the efficacy of Trauma Risk Management (TRiM) in a military population. J Trauma Stress ;23(4):430-437.
27. The Point of Care Foundation. List of Schwartz Rounds sites. [internet] [cited 2020 May 19]. Available from: https://www.pointofcarefoundation.org.uk/our-work/schwartz-rounds/list-of-sites/
28. Taylor C, Xyrichis A, Leamy M, Reynolds E, Maben J. Can Schwartz Center Rounds support healthcare staff with emotional challenges at work, and how do they compare with other interventions aimed at providing similar support? A systematic review and scoping reviews. BMJ Open. 2018;8(10):e024254.


For peer review only



For peer review only - http://bmjopen.bmj.com/site/about/guidelines.xhtml

29. Maben J, Taylor C, Dawson J, Leamy M, Mccarthy I, Reynolds E, et al. A realist informed mixed-methods evaluation of Schwartz Center Rounds® in England. Health Services and Delivery Research 1101;6(37).
30. HMICFRS. Fire and rescue- Staffordshire. [internet] 2019 [cited 2020 April 5]. Available from: https://www.justiceinspectorates.gov.uk/hmicfrs/frs-assessment/frs-2018/staffordshire/people/
31. HMICFRS. Fire and rescue- Buckinghamshire. [internet] 2019 [cited 2020 April 5]. Available from: https://www.justiceinspectorates.gov.uk/hmicfrs/frs-assessment/frs-2018/buckinghamshire/
32. HMICFRS. Fire and rescue- Derbyshire. [internet] 2019 [cited 2020 April 5]. Available from: https://www.justiceinspectorates.gov.uk/hmicfrs/frs-assessment/frs-2018/derbyshire/
33. HMICFRS. Police- Dfyed-Powes. [internet] 2019 [cited 2020 April 5]. Available from: https://www.justiceinspectorates.gov.uk/hmicfrs/peel-assessments/peel-2018/dyfed-powys/legitimacy/detailed-findings/#question-3
34. HMICFRS. Police- Cumbria. [internet] 2019 [cited 2020 April 5]. Available from: https://www.justiceinspectorates.gov.uk/hmicfrs/peel-assessments/peel-2018/cumbria/legitimacy/detailed-findings/#question-1
35. Her Majesty's Inspectorate of Constabulary and Fire & Rescue Services (HMICFRS). Fire and rescue- Gloucestershire. [internet] 2019 [cited 2020 April 5]. Available from: https://www.justiceinspectorates.gov.uk/hmicfrs/frs-assessment/frs-2018/gloucestershire/people/
36. UNISON. Linc Peer Support. [internet] 2016 [cited 2020 April 20]. Available from: https://lasunison.com/linc-peer-support
37. Haugen PT, McCrillis AM, Smid GE, Nijdam MJ. Mental health stigma and barriers to mental health care for first responders: A systematic review and meta-analysis. Journal of Psychiatric Research. 2017(10):125-126.
38. Thomas J. Methods for the thematic synthesis of qualitative research in systematic reviews. BMC Medical Research Methodology ;8:45-46.
39. Barnett-Page E, Thomas J. Methods for the synthesis of qualitative research: a critical review. BMC Medical Research Methodology. 2009;9(1).
40. Thomas J, Sutcliffe K, Harden A, Oakley A, Oliver S, Rees R, Brunton G, Kavanagh J (2003) Children and Healthy Eating: A systematic review of barriers and facilitators.



For peer review only - http://bmjopen.bmj.com/site/about/guidelines.xhtml

London: EPPI-Centre, Social Science Research Unit, Institute of Education, University of London.

41. Coleman SJ, Stevelink SAM, Hatch SL, Denny JA, Greenberg N. Stigma-related barriers and facilitators to help seeking for mental health issues in the armed forces: a systematic review and thematic synthesis of qualitative literature. 1708;47(11):1880-1892.
42. Flemming K, Noyes J. Qualitative Evidence Synthesis: Where Are We at?. International Journal of Qualitative Methods. 2021;20:160940692199327.
43. Booth A, Noyes J, Flemming K, Moore G, Tunçalp Ö, Shakibazadeh E. Formulating questions to explore complex interventions within qualitative evidence synthesis. BMJ Global Health. 2019;4(Suppl 1):e001107.
44. Litz BT. Distinct Trauma Types in Military Service Members Seeking Treatment for Posttraumatic Stress Disorder. J Trauma Stress 2018;31(2):286-296.
45. Ridner SH. Psychological distress: concept analysis. J Adv Nurs. 2004 ;45(5):536-546.
46. Noyes J & Lewin S. Chapter 5: Extracting qualitative evidence. In: Noyes J, Booth A, Hannes K, Harden A, Harris J, Lewin S, Lockwood C (editors), Supplementary Guidance for Inclusion of Qualitative Research in Cochrane Systematic Reviews of Interventions. Version 1 (updated August 2011). Cochrane Collaboration Qualitative Methods Group, 2011. Available from URL http://cqrmg.cochrane.org/supplemental-handbook-guidance .
47. CASP. Qualitative checklist. [internet] 2018 [cited 2020 March 5]. Available from: https://casp-uk.net/wp-content/uploads/2018/03/CASP-Qualitative-Checklist-2018_fillable_form.pdf
48. Dixon-Woods M, Cavers D, Agarwal S, Annandale E, Arthur A, Harvey J, et al. Conducting a critical interpretive review of the literature on access to healthcare by vulnerable groups. 2006.
49. Sommerfeld A, Wagner S, Harder H, Schmidt G. Behavioral Health and Firefighters: An Intervention and Interviews with Canadian Firefighters. Journal of Loss and Trauma. 2017;22(4):307-324.
50. Regehr C, Goldberg G, Hughes J. Exposure to human tragedy, empathy, and trauma in ambulance paramedics. American Journal of Orthopsychiatry. 2002;72(4):505-513.
51. Regehr C, Millar D. Situation critical: High demand, low control, and low support in paramedic organizations. Traumatology. 2007;13(1):49-58.



For peer review only - http://bmjopen.bmj.com/site/about/guidelines.xhtml

52. Halpern J, Gurevich M, Schwartz B, Brazeau P. What makes an incident critical for ambulance workers? Emotional outcomes and implications for intervention. Work & Stress. 2009;23(2):173-189.
53. Halpern J, Gurevich M, Schwartz B, Brazeau P. Interventions for critical incident stress in emergency medical services: a qualitative study. Stress and Health. 2009;25(2):139-149.
54. Jonsson A, Segesten K. The meaning of traumatic events as described by nurses in ambulance service. Accident and Emergency Nursing. 2003;11(3):141-152.
55. Jonsson A, Segesten K. Guilt, shame and need for a container: a study of post-traumatic stress among ambulance personnel. Accident and Emergency Nursing. 2004;12(4):215-223.
56. Jeruzal J, Boland L, Frazer M, Kamrud J, Myers R, Lick C et al. Emergency Medical Services Provider Perspectives on Pediatric Calls: A Qualitative Study. Prehospital Emergency Care. 2019;23(4):501-509.
57. Fernández-Aedo I, Pérez-Urdiales I, Unanue-Arza S, García-Azpiazu Z, Ballesteros-Peña S. A qualitative study about experiences and emotions of emergency medical technicians and out-of-hospital emergency nurses after performing cardiopulmonary resuscitation resulting in death. Enfermería Intensiva (English ed). 2017;28(2):57-63.
58. Svensson A, Fridlund B. Experiences of and actions towards worries among ambulance nurses in their professional life: A critical incident study. International Emergency Nursing. 2008;16(1):35-42.
59. Clompus S, Albarran J. Exploring the nature of resilience in paramedic practice: A psycho-social study. International Emergency Nursing. 2016;28:1-7.
60. Douglas L, Cheskes S, Feldman M, Ratnapalan S. Paramedics' experiences with death notification: a qualitative study. Journal of Paramedic Practice. 2012;4(9):533-539.
61. Carvello M, Zanotti F, Rubbi I, Bacchetti S, Artioli G, Bonacaro A. Peer-support: a coping strategy for nurses working at the Emergency Ambulance Service. *Acta Biomed*. 2019;90(11-S):29-37
62. Oliveira A, Neto F, Teixeira F, Maia Â. Working in prehospital emergency contexts. International Journal of Workplace Health Management. 2019;12(6):469-482.
63. Drewitz-Chesney C. Exploring paramedic communication and emotional expression in the workplace after responding to emergency calls. Australasian Journal of Paramedicine. 2019;16.




For peer review only - http://bmjopen.bmj.com/site/about/guidelines.xhtml

64. Gallagher S, McGilloway S. Living in critical times: The impact of critical incidents on frontline ambulance personnel--a qualitative perspective. *Int J Emerg Ment Health*. 2007;9(3):215-223.
65. Folwell A, Kauer T. 'You see a baby die and you're not fine:' a case study of stress and coping strategies in volunteer emergency medical technicians. Journal of Applied Communication Research. 2018;46(6):723-743.
66. Evans R, Pistrang N, Billings J. Police officers' experiences of supportive and unsupportive social interactions following traumatic incidents. European Journal of Psychotraumatology. 2013;4(1):19696.
67. Strydom H, Botha K, Boshoff P. AN ASSESSMENT OF THE NEED OF POLICE OFFICIALS FOR TRAUMA INTERVENTION PROGRAMMES – A QUALITATIVE APPROACH. Social Work/Maatskaplike Werk. 2015;51(2).
68. Edwards A, Kotera Y. Mental Health in the UK Police Force: a Qualitative Investigation into the Stigma with Mental Illness. International Journal of Mental Health and Addiction. 2020;.
69. Bullock K, Garland J. Police officers, mental (ill-)health and spoiled identity. Criminology & Criminal Justice. 2017;18(2):173-189.
70. Haslam C, Mallon K. A preliminary investigation of post-traumatic stress symptoms among firefighters. Work & Stress. 2003;17(3):277-285.
71. Abelsson A. First response emergency care – experiences described by firefighters. International Journal of Emergency Services. 2019;8(3):247-258.
72. Jones S, Agud K, McSweeney J. Barriers and Facilitators to Seeking Mental Health Care Among First Responders: "Removing the Darkness". Journal of the American Psychiatric Nurses Association. 2019;26(1):43-54.
73. Hasselqvist-Ax I, Nordberg P, Svensson L, Hollenberg J, Joelsson-Alm E. Experiences among firefighters and police officers of responding to out-of-hospital cardiac arrest in a dual dispatch programme in Sweden: an interview study. BMJ Open. 2019;9(11):e030895.
74. Fridgen G, Aston J, Gschwandtner U, Pflueger M, Zimmermann R, Studerus E, et al. Help-seeking and pathways to care in the early stages of psychosis. Soc Psychiatry Psychiatr Epidemiol 1307;48(7):1033-1043.
75. Gronholm PC. Mental health-related stigma and pathways to care for people at risk of psychotic disorders or experiencing first-episode psychosis: a systematic review. Psychol Med ;47(11):1867-1880.



For peer review only - http://bmjopen.bmj.com/site/about/guidelines.xhtml

76. Clement S, Schauman O, Graham T, Maggioni F, Evans-Lacko S, Bezborodovs N, et al. What is the impact of mental health-related stigma on help-seeking? A systematic review of quantitative and qualitative studies. Psychol Med 2015 101 0;45(1):11-27.
77. Sharp M, Fear NT, Rona RJ, Wessely S, Greenberg N, Jones N, et al. Stigma as a Barrier to Seeking Health Care Among Military Personnel With Mental Health Problems. Epidemiol Rev 2015 /01/01;37(1):144-162.
78. Australian Bureau of Statistics. National Survey of Mental Health and Wellbeing: Summary of Results, Australia, 2007. ABS cat. no. 4326.0. Canberra: ABS; 2008.
79. Milner A, Scovelle A, King T. Treatment-seeking differences for mental health problems in male- and non-male-dominated occupations: evidence from the HILDA cohort. Epidemiology and Psychiatric Sciences. 2018;28(6):630-637.
80. Milner A, King T. Men's work, women's work and suicide: a retrospective mortality study in Australia. Australian and New Zealand Journal of Public Health. 2018;43(1):27-32.
81. Peters K, Ryan M, Haslam S. Marines, medics, and machismo: Lack of fit with masculine occupational stereotypes discourages men's participation. British Journal of Psychology. 2014;106(4):635-655.
82. E. Goffman. Stigma: Notes on the management of spoiled identity. Prentice-Hall, Englewood Cliffs, NJ (1963)
83. Dickstein BD, Vogt DS, Handa S, Litz BT. Targeting Self-Stigma in Returning Military Personnel and Veterans: A Review of Intervention Strategies. Military Psychology 2010 April 2,;22(2):224-236.
84. Norms, Committee on the Science of Changing Behavioral Health Social, Board on Behavioral C, Education, Division of Behavioral and Social Sciences and, National Academies of Sciences, Engineering. Approaches to Reducing Stigma. : National Academies Press (US); 2016.
85. Like Minds. THE POWER OF CONTACT- Project to Counter Stigma and Discrimination Associated with Mental Illness. [internet] 2005 [cited 2020 April 6]. Available from: https://www.likeminds.org.nz/assets/National-Plans/1power-of-contact.pdf
86. Jorm AF, Barney LJ, Christensen H, Highet NJ, Kelly CM, Kitchener BA. Research on Mental Health Literacy: What we know and what we Still Need to know. Aust N Z J Psychiatry 2006 January 1,;40(1):3-5.


For peer review only - http://bmjopen.bmj.com/site/about/guidelines.xhtml

87. Jorm AF. Mental Health Literacy: Empowering the Community to Take Action for Better Mental Health. Am Psychol ;67(3):231-244.
88. Jacobsson A, Backteman-Erlanson S, Brulin C, Hörnsten Å. Experiences of critical incidents among female and male firefighters. International Emergency Nursing. 2015;23(2):100-104.
89. Halpern J, Maunder R, Schwartz B, Gurevich M. The critical incident inventory: characteristics of incidents which affect emergency medical technicians and paramedics. BMC Emergency Medicine 101 0;12:10.
90. Brewin CR, Andrews B, Valentine JD. Meta-Analysis of Risk Factors for Posttraumatic Stress Disorder in Trauma-Exposed Adults. J Consult Clin Psychol 10 00;68(5):748-766.
91. Gouweloos-Trines J, Tyler MP, Giummarra MJ, Kassam-Adams N, Landolt MA, Kleber RJ, et al. Perceived support at work after critical incidents and its relation to psychological distress: a survey among prehospital providers. Emerg Med J 1221;34(12):816
92. Alexander D, Klein S. Ambulance personnel and critical incidents: impact of accident and emergency work on mental health and emotional well-being. British Journal of Psychiatry 101 0(178):76-81.
93. CARLIER I, LAMBERTS R, GERSONS B. Risk Factors for Posttraumatic Stress Symptomatology in Police Officers: A Prospective Analysis. The Journal of Nervous &amp Mental Disease. 1997;185(8):498-506.
94. Alexander D, Klein S. Ambulance personnel and critical incidents: impact of accident and emergency work on mental health and emotional well-being. British Journal of Psychiatry 101 0(178):76-81.
95. Mitchell AM. Critical incident stress debriefing: implications for best practice. Disaster Management & Response 2003;1(2):46-52.
96. Halpern J. Downtime after critical incidents in emergency medical technicians/paramedics. BioMed Research International ;2014:483140-483141.
97. Greenberg N, Langston V, Iversen AC, Wessely S. The acceptability of 'Trauma Risk Management' within the UK Armed Forces. Occup Med (Lond) 2011 /05/01;61(3):184-189.
98. Mind. Mind Blue light champions. [internet] 2019 [cited 2020 Feb 19]. Available from: https://www.mind.org.uk/media-a/4843/blp-role-description.pdf



For peer review only - http://bmjopen.bmj.com/site/about/guidelines.xhtml

99. Mind. Blue Light Programme Strand 1 (Part 2). [internet] 2016 [cited 2020 May 5]. Available from: https://www.mind.org.uk/media-a/4864/strand-1-part-2.pdf

100. Cai H, Tu B, Ma J, Chen L, Fu L, Jiang Y et al. Psychological impacts and coping strategies of front-line medical staff during COVID-19 outbreak in Hunan, China. Medical Science Monitor. 2020;26.

101. Vagni M, Maiorano T, Giostra V, Pajardi D. Coping With COVID-19: Emergency Stress, Secondary Trauma and Self-Efficacy in Healthcare and Emergency Workers in Italy. Frontiers in Psychology. 2020;11.

For peer review only

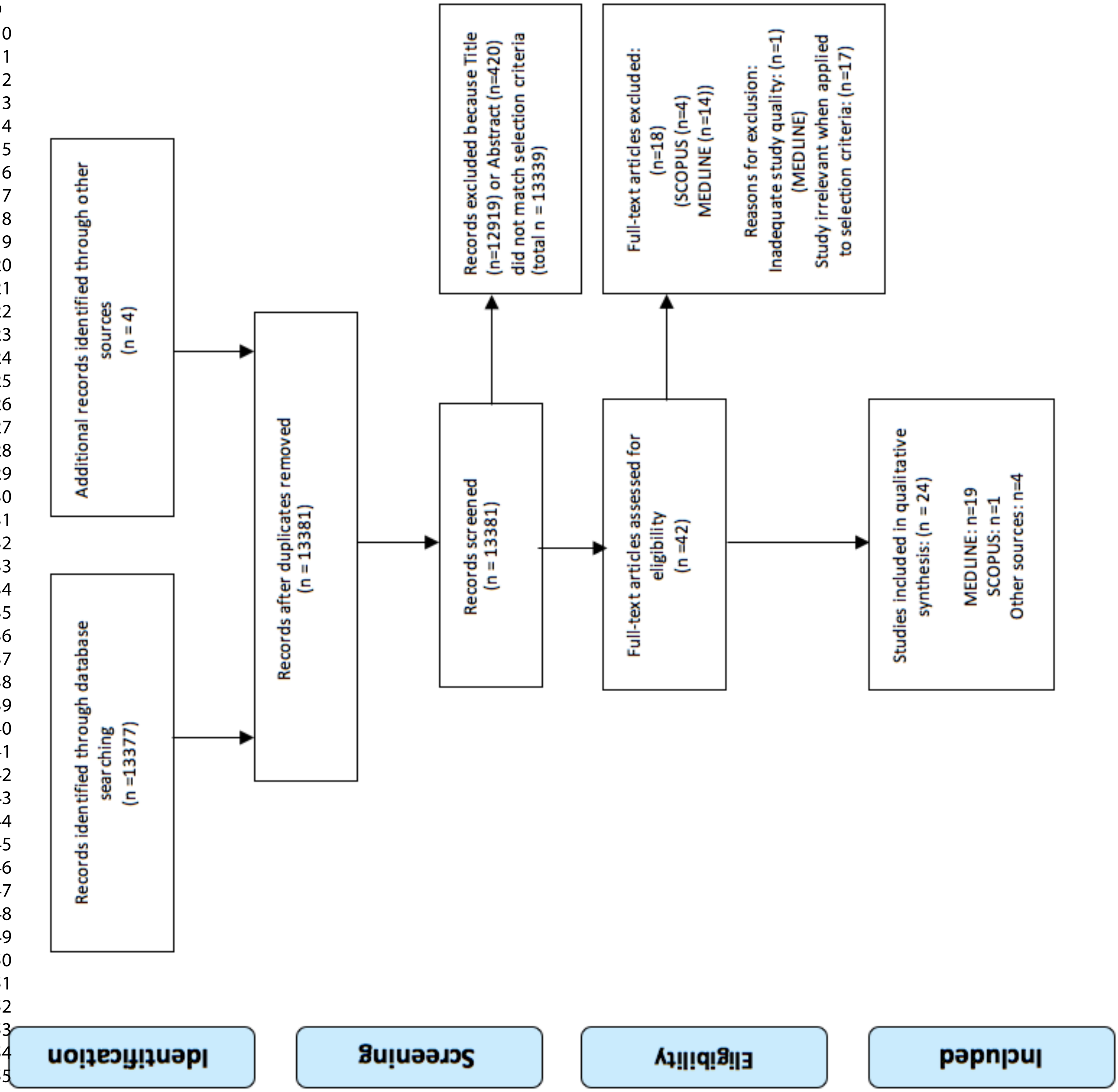
Identification
Screening
Eligibility
Included
Records identified through database searching (n =13377)
Additional records identified through other sources (n = 4)
Records after duplicates removed (n = 13381)
Records screened (n = 13381)
Records excluded because Title (n=12919) or Abstract (n=420) did not match selection criteria (total n = 13339)
Full-text articles assessed for eligibility (n =42)
Full-text articles excluded: (n=18) (SCOPUS (n=4) MEDLINE (n=14))
Reasons for exclusion: Inadequate study quality: (n=1) (MEDLINE)
Study irrelevant when applied to selection criteria: (n=17)
Studies included in qualitative synthesis: (n = 24)
MEDLINE: n=19
SCOPUS: n=1
Other sources: n=4

**Online supplementary file 1: ENTREQ checklist (Enhancing transparency in reporting the synthesis of qualitative research**

| No | Item | Guide and description |
|---|---|---|
| 1 | Aims | To synthesise the evidence on:<br>1. The factors that contribute to mental health protection and recovery as expressed by emergency service staff following occupational critical incident exposure<br>2. The barriers to and facilitators of mental health help-seeking behaviour among psychologically distressed emergency service staff |
| 2 | Synthesis Methodology | Thematic synthesis |
| 3 | Approach to searching | Pre-planned comprehensive search strategies, combining database searching with manual search methods. |
| 4 | Inclusion criteria | Articles were eligible for inclusion provided they met the following criteria:<br>(1) Study participants were frontline ESWs* (studies with mixed populations of eligible participants were included); (2) There is a focus on work-related psychological distress*; (3) Data collection includes primary qualitative interviews, focus groups or observational methods (this included mixed methods studies with qualitative components); (4) Analysis focussed around participant attitudes towards: a) behaviour aimed at improving or protecting mental health after experiencing a traumatic event OR b) factors which ESWs find helpful or unhelpful for their mental health while experiencing work-related psychological distress, are explored; (5) Published in English and peer-reviewed.<br>Exclusion criteria: (1) Due to the unique nature of the traumatic events witnesses in this cohort, studies investigating a military cohort will be excluded; (2) Volunteer ESWs were not included. |
| 5 | Data sources | OVID MEDLINE, EMBASE, PsycINFO, SCOPUS. To identify articles missed in the electronic database search, the following methods were also employed:<br>-Using ‘related article’ feature (when available). |

For peer review only

| | | |
|---|---|---|
| | | -Searching the titles of included studies in google scholar for citation tracking purposes.<br>-Manual searching of the references of relevant studies (reference chaining).<br>Grey literature was searched during background research for context but not to locate eligible studies. |
| 6 | Electronic search strategy | The two combinations of search criteria (see Appendix A) were entered into each database. Figure 1 provides an overview of the database search. |
| 7 | Study screening methods | The eligibility criteria were applied against the titles of the database search results by one reviewer (NA). The full text of studies with potential relevance was screened. (Figure 1) |
| 8 | Study characteristics | Study characteristics are presented in Table 1. |
| 9 | Study selection results | PRISMA guidance was used to construct a flow diagram displaying the database searching process (Figure 1). Of the 13381 records identified once duplicates were removed, the full text of 42 articles were screened, and 24 studies were included in this qualitative synthesis. |
| 10 | Rationale for appraisal | All potentially relevant studies were quality appraised by one reviewer (NA), using the Critical Appraisal Skills Program (CASP) guidelines. Studies meeting less than 5 criteria were then subject to further scrutiny, in the form of five quality appraisal prompts developed by Dixon-Woods et al. (see 'quality appraisal' section). This second stage of appraisal was carried out independently by by RR, MB or JW, and decisions relating to the inclusion of these studies in the review were made following thorough communication between reviewers and referring to pre-determined quality prompts. |
| 11 | Appraisal terms | Critical Appraisal Skills Program (CASP) guidelines and quality appraisal prompts (see 'quality appraisal' section) were used to quality appraise all included studies. |
| 12 | Appraisal process | Quality assessment was carried out primarily by one reviewer (NA), with independent verification from RR, MB or JW for ambiguous studies. |

For peer review only

| | | |
|---|---|---|
| 13 | Appraisal results | CASP scores of included studies are provided in Table 1. Full study quality assessments are available for review if required. |
| 14 | Data extraction | A data extraction template (Appendix B) was created for this review by one reviewer (NA). Sections for first, second and third order constructs are included in the extraction template, which was filled manually by one reviewer (NA). |
| 15 | Software | RefWorks reference management software. |
| 16 | Number of reviewers | Two reviewers were involved in the coding and analysis (NA, RR). |
| 17 | Coding | One reviewer carried out line-by-line coding (NA). RR aided with the grouping of codes, and assisted with the generation of descriptive themes. |
| 18 | Study comparison | During primary readings of the studies, overarching concepts relating to the research aims were noted. The generation of new codes altered pre-existing codes. |
| 19 | Derivation of themes | Initial coding was carried out in correspondence between two reviewers (NA and RR), and was grounded in the extracted data. Thematic synthesis of initial codes was an inductive approach, and involved roundtable discussion between all four authors. |
| 20 | Quotations | Appendix C provides example literature quotations from included studies which were used to construct themes. |
| 21 | Synthesis output | This qualitative review generated 14 descriptive themes. These are grouped into either 'factors contributing to mental health recovery post traumatic incident exposure' or 'factors influencing mental health help-seeking behaviour'. Table 2 presents a summary of themes. These descriptive themes were applied to the context of UK emergency service organisations to produce implications for practice and research. |

For peer review only

Tong A. Enhancing transparency in reporting the synthesis of qualitative research: ENTREQ. BMC Medical Research Methodology ;12(1):181-182.

# Manuscript online supplementary files

Online Supplementary File 1: ENTREQ checklist (submitted separately)

**Online Supplementary File 2: CASP appraisal table**

**Online Supplementary File 2: CASP appraisal table**

| Study title (Authors, year) | Aims & Methods | Research design | Sampling | Data collection | Reflexivity | Ethical issues | Data analysis | Discussion of findings | Value |
|---|---|---|---|---|---|---|---|---|---|
| *What makes an incident critical for ambulance workers? Emotional outcomes and implications for intervention.* Halpern et al. 2009[42] | Research aim clearly stated, introduction uses relevant literature to successfully explain demand for the research. Qualitative methods are appropriate for investigating complicated emotions, which could not be explored in equal depth via quantitative methods. | Interviews and focus groups seem appropriate methods to address the research aims, but the authors don't specify the reasons for their choice to use both methods. | 4 supervisors, 54 front-line ambulance workers. Researchers state that participants were recruited from a specific cohort at a mandatory training conference, but no detail is given about what information was provided to the participants to entice them to enter the study. The authors considered representation of all job levels and both genders | The setting of the data collection away from the workplace was stated but no justification was given for the choice of study setting or methods used. Detail was given about the length of interviews and focus groups, and the main topics of questions asked during interviews and focus groups were stated. The researchers stated they had reached saturation by the end of the study. Data obtained was in the form of | Researchers do not acknowledge how their characteristics may have impacted on the results. The researchers acknowledge that self-selection or reporting bias may have contributed to the discrepancy in findings between men and women. | Ethical approval was obtained from a research ethics board, and participants signed a consent form. No further information was given regarding any ethical safeguards in place. Lack of description of how confidentiality was maintained. | The authors described how ethnographic content analysis was used to generate thematic codes, with a constant comparative method throughout. Three authors were involved in the coding process, although only two of these authors listened to the audiotapes. Initial and final codes are described, and contradictory data are taken into account. Sufficient data are | Findings are explicitly stated and are relevant to the researchers' aims. The only measure of credibility described was the use of three analysts during the coding process. Findings are discussed with reference to wider literature, and implications for interventions for critical incident stress are made. | The authors identify promising post-incident interventions which they believe should be further researched. Wider applicability of the study findings appear to not extend beyond the ambulance cohort. The authors provide implications for interventions for critical incident stress. |

For peer review only

| | | | | | | | | | |
|---|---|---|---|---|---|---|---|---|---|
| | | | during recruitment. | audio recordings, written transcripts and written notes of non-verbal communication information as observed by the researchers. | | | presented to support the findings. The authors describe how textual data was not analysed for focus groups. | | |
| *Police officers' experiences of supportive and unsupportive social interactions following traumatic incidents.* Evans et al. 2013[56] | Research aims made clear in objectives section. Overall aim 'was to understand the types of support processes that might promote resilience'. Methods are appropriate as experiences, and other subjective outcomes are best measured qualitatively. | Semi-structured interview design appears reasonable to explore the proposed aims, but choice of qualitative study design not directly justified. | 19 Police officers. A snowballing approach was used to recruit officers, which allowed detection of information-rich participants. By limiting participants to those who have served over 2 years, the chance of traumatic incident exposure was increased. Limiting participants to those without diagnosed PTSD was appropriate as the study measured resilience-promoting investigations. Non-participation was not discussed. | An interview schedule was used for the semi-structured interviews, although only the broad focus of the schedule was provided by the authors. Interviews were audio-recorded and transcribed verbatim. No information was given regarding interview setting, and data saturation was not discussed. No justification for choice of methods. | Researchers acknowledge how their role as researchers could have affected the data, giving social desirability bias as an example. | A National Health Service ethics committee granted ethical approval, and participants provided informed consent. No information is provided regarding efforts to maintain confidentiality. | The process of thematic analysis was described in detail. All themes generated can be linked with relevant first order data. Contradictory data is taken into account and present within the themes. Three authors were involved in the analytic process to minimise bias. One author had the main role in the analysis, while two others cross-referenced certain parts of their work in order to increase credibility. All 3 researchers reached a consensus on unclear issues before the final themes were decided upon. | Themes are clearly summarised, with relevant quotations to support each theme. Findings are discussed in relation to the research aims. Three researchers were involved in the analytic process. | The researchers provide detailed recommendations for further quantitative research. Furthermore, the researchers recommend similar qualitative research in a population that has a history of PTSD, so that comparisons can be made. |
| *Interventions for critical incident stress in emergency medical services: a qualitative study* | Clear statement of study aims, with overall study purpose ' to facilitate development of interventions that | Explanations were provided for how the focus groups and semi-structured interviews were conducted. E.g | 4 supervisors, 54 front-line ambulance workers. Participants were sampled to ensure all job levels and | The setting of the data collection away from the workplace was stated but not explained. Detail was given about | Researchers do not critically examine their role, potential bias and influence of research | Ethical approval was obtained from a research ethics board, and participants signed a consent form. No further | The authors described how ethnographic content analysis was used to generate thematic codes, using a | The findings are clearly presented, using relevant participant quotations to support the authors | The research conclusions appear relevant to all EMS organisations which operate in the format of |

| | | | | | | | | | |
|---|---|---|---|---|---|---|---|---|---|
| Halpern et al. 2008[43] | take into account EMS culture.' Examining experiences adequately requires qualitative methodology. | flexible interview structure ' to permit the elaboration of more in-depth or emotionally significant data' and focus group size of 4-8 members 'to maximize interactive data'. No justification given for choice of interviews and focus groups. | genders were represented. Researchers state that participants were recruited from a specific cohort at a mandatory training conference, but no detail is given about what information was provided to the participants to entice them to enter the study. The sampling process was iterative-preliminary analysis informed subsequent sampling decisions. No further participants were recruited once saturation was reached. | the length of interviews and focus groups, and the main topics of questions asked during interviews and focus groups were stated. The researchers stated they had reached saturation by the end of the study. Data obtained was in the form of audio recordings, written transcripts and written notes of non-verbal communication information as observed by the researchers. | question formulation or data collection. | information was given regarding any ethical safeguards in place. | constant comparative method to categorise themes. Initial and final codes are described. The authors explain why textual data was not analysed for focus groups. Member validation was carried out 6 months post initial data collection. Contradictory data are presented and discussed, and sufficient data are presented to support the findings. | interpretations. 3 analysts were used during the data analysis process. Member validation was carried out 6 months after data was gathered. Contradictory viewpoints are taken into account and discussed. | using supervisors. The authors provide suggestions for interventions which are of relevance to EMS organisations. The authors recommend that the interventions of:<br>1. Supervisor support<br>2. Timeout period post-incident<br><br>should be further researched. |
| *Barriers and Facilitators to Seeking Mental Health Care Among First Responders: "Removing the Darkness"* Jones et al, 2020[62] | Aims are clearly stated and the study's importance justified. The authors aims include exploring organisational culture and perceptions of individuals, for which qualitative research is appropriate. | The decision to use individual ethnographic interviews the qualitative method of choice is well explained. The authors also explain why they chose a 'community-based approach'. | 32 firefighters and/or EMTs/paramedics.<br><br>This was a convenience sample.<br>The principal investigator facilitated recruitment by developing relationships with community partners who each recruited participants from | Ethnographic ndividual interviews were conducted with participants by the principal investigator. An interview guide was used and attached, which was developed by the PI based on the study aim and input from a qualitative methods expert, | The principal investigator acknowledged how being married to a firefighter/paramedic promoted buy in from the community during the recruitment stage. | Ethical board approval was gained. 'Prior to starting the interview, the PI reviewed the study information sheet and completed the consent process with each participant. ' Resources for further emotional support were provided to participants in case of emotional | Content analysis and constant comparison methods were used during the analytic process. Themes which emerged during data analysis were checked with participants in later interviews. Two researchers independently analysed the transcripts, and discussed the process until | Findings are presented as themes, with participant quotations included for each theme to support the author's interpretations. Credibility was enhanced by the use of more than one analyst, and member validation. Findings are discussed in relation to the | The authors make implications for practice, identifying 'need for improving education and awareness regarding duty-related MH problems.' The authors also make implications for nursing practice, and implications for future research. |

| | | | | | | | | | |
|---|---|---|---|---|---|---|---|---|---|
| | | | their own organisations. They did this by distributing e-mails on behalf of the principal investigator. Reasons for non-participation: 'individuals not answering or returning calls (all were called twice), scheduling conflicts' | along with feedback from community partners to strengthen trustworthiness and validity. Interviews were audio recorded and transcribed. Field notes were also conducted by the authors. Data collection setting was decided on the basis of convenience for participants. Data saturation was reached. | | distress experienced during the study. | consensus was reached. (on 80% of top level codes). Exploration of new themes was stopped when saturation was reached. participant quotations are provided throughout to support the author's interpretations. The researchers acknowledge a bias risk due to the principal investigator conducting all the interviews. Sufficient data are presented to support the findings, and contradictory data are reported (such as positive and negative experiences of therapists). | original research question. | |
| *Exposure to human tragedy, empathy, and trauma in ambulance paramedics.* Regehr et al, 2002[40] | Aims of study are clearly stated, and their importance justified. Qualitative methods are appropriate because the authors want to explore experiences in depth. | Choice of qualitative study design not explicitly justified. | 18 paramedics. 'Purposive sampling was used to ensure that participants represented a wide range of experiences in terms of length of time with the service and types of events encountered.' Non participation | A semistructured interview guide was used to conduct interview, and the authors provided examples of guide topics. These interviews were audio-recorded and transcribed, and field notes were taken. The authors acknowledge they | Researchers do not critically examine their role, potential bias and influence of research question formulation or data collection. No mention of changing the research design as the study progressed. | No mention of ethical approval, or consent gained from participants. No attempt is made to explain how confidentiality was maintained. | Nvivo was used to aid in data analysis. Open coding was used initially to generate broad categories. Towards the later stages of the process, selective coding was used to develop a meaningful narrative of the experiences of the | Findings are presented in sections headed by themes. Author's comments are supported by first order interpretations. Triangulation with members of emergency service organization was carried out | The authors acknowledge that the study is 'not intended to reflect the views of all paramedics in all organizations; rather, it describes a particular phenomenon experienced and described by one group of paramedics and |

For peer review only

| | | | | | | | | | |
|---|---|---|---|---|---|---|---|---|---|
| | | | was not discussed. | required a larger than normal sample size to achieve saturation. No information is provided for the setting of data collection.<br><br>‘Other sources of data included the notes recording the interviewer’s impressions.’ | | | workers. Interviewers also recorded field notes. Triangulation with members of emergency service organization was carried out throughout the research, and analysis of data was discussed. Two members of the research team developed the coding tree together. A third member reviewed the open and selective coding. It is not clear how the themes were derived from the data, but sufficient data are presented to support the findings. Contradictory data are included in the findings and discussed. | throughout the research, and analysis of data was discussed. Two members of the research team developed the coding tree together. A third member reviewed the open and selective coding. | points to some interesting avenues for further consideration. These include future attempts to measure cognitive and emotional empathy in paramedics and assess the impact of these strategies on posttraumatic and depressive symptoms and on social supports.’ |
| *The meaning of traumatic events as described by nurse sin ambulance service*<br>Jonsson et al. 2003[44] | Aims of study are clearly stated, and their importance justified. Qualitative methodology is appropriate due to nature of the aims, which is to explore subjective experiences in detail. | A phenomenological approach was used, and the reason for its us thoroughly explained. The use of self-reports over the use of interviews was not justified (the authors said that interviews and self-reports are | 240 medical technicians and 122 registered ambulance nurses.<br>The authors asked 500 participants of another study to write down their experiences with traumatic events. No detail is given how the sample of the | The question the participants were asked to answer was: ‘Write down and describe a situation which you experienced as a traumatic event’’. From the data returned by the participants, the authors selected 25% of the written | Researchers do not critically examine their role, potential bias and influence of research question formulation or data collection. No mention of changing the research design as the study progressed. | Ethical approval was granted. Consent was implied through willingness to participate in the study, but no information is given regarding a signed consent form. In addition, participants were assured that they could withdraw | Van Kaam's scientific explication was used to analyse the written stories. Detail was provided about the steps of this thematic analytic process. Only 25% of the written stories were further analysed, and | The findings are in the form of themes, supported by first order data and the author's interpretations. The findings are related to the original research question in the discussion. There is no mention of any efforts to increase the | The authors recommend specific areas for further research, limited to the ambulance sector. The organisational interventions recommended by the authors are likewise limited to the ambulance sector. |

For peer review only

| | | | | | | | | | |
|---|---|---|---|---|---|---|---|---|---|
| | | the most common methods in empiric-phenomenological studies. | other study was recruited. Non participation is not discussed. | accounts to further analyse. These written accounts were strategically selected based on having the fullest description of a traumatic event. Details of the analytic process are provided. Saturation of data is not discussed. | | from the study at any time. The questionnaire was not labelled with the name of the participant. Confidentiality was guaranteed by eliminating names or other identifying characteristics from the essays. | were strategically selected for being more data-rich. There is no information to reveal how many researchers are involved in the analytic process. Sufficient data is presented to support the findings. There is no mention of contradictory data being taken into account. The researcher does not critically appraise their own potential bias. | credibility of the findings. | |
| *Guilt, shame and need for a container: a study of post-traumatic stress among ambulance personnel* Jonsson et al. 2004[45] | Aims of study are clearly stated, and their importance justified. Qualitative methodology is appropriate due to nature of the aims, which is to explore subjective experiences in detail. | Descriptive phenomenology was 'chosen for its potential to grasp the meaning of such phenomena through the description of lived experiences.' | 10 Ambulance nurses or ambulance technicians. Insufficient information on how participants were The only relevant information is that participants were strategically selected to obtain a 'variation of sex, age, educational background, and experience as ambulance staff.' | Setting for interviews was described but not justified. Interviews were open in structure, beginning with the question "tell about a traumatic event that you have experienced". 'do you mean?" or "How did you feel about that?". The interviews, taking about an hour and a half each, were audio-taped and transcribed word by word.' Reason for choice of interview as method of data collection not | Researchers do not critically examine their role, potential bias and influence of research question formulation or data collection. | The study was described, the extent of participation was explained, and potential risks were explored with the participants, who were assured that they had the right to withdraw from the study at any time. The study was approved by the Ethical committee at Göteborg University. To protect participant confidentiality, no quotations were provided. | The authors provide details about the 5 steps of the analytic process, with the latter steps being repeated once the author was familiar with existing philosophy. Due to the authors not using participant quotations (to protect participant confidentiality), it is not possible to determine whether the author's findings are supported by first order interpretations. Contradictory data are discussed, but | Findings are displayed under relevant sub-headings. In the discussion section, there is some debate for and against the researcher's arguments. No attempts made to demonstrate efforts to enhance credibility. | The results can be applied to emergency medical organisations, however caution should be taken due to the small sample size and lack of information about participant demographics. The authors recommend for the insights gained by this study to be ' distributed to all ambulance managers and other relevant personnel categories. ' The authors make specific |

For peer review only

| | | | | | | | | | |
|---|---|---|---|---|---|---|---|---|---|
| | | | | explained. Saturation of data not discussed. | | | without the direct participant quotations. | | recommendations for management. |
| *Situation Critical: High Demand, Low Control, and Low Support in Paramedic Organizations* Regehr et al. 2007[41] | Aims of study are clearly stated, and their importance justified. Qualitative methodology is appropriate due to nature of the aims, which is to explore subjective experiences in detail. | Long interview design was chosen 'to explore in detail the experiences of paramedics in their organizations including their roles, the demands placed on them, the control over the working environment that they experience, and the supports that they receive.' | 17 paramedics. Paramedics were purposively sampled from the sample of participants partaking in the quantitative component of the study. Purposive sampling was used to ensure that participants represented a wide range of experiences in terms of length of time with the services and types of work experiences encountered. | A long interview method was used to collect data. The interviews followed an interview guide (not provided), were audio recorded and transcribed verbatim. The interviewer made their own notes during the interview. Setting of data collection not provided, saturation of data not discussed. | Researchers do not critically examine their role, potential bias and influence of research question formulation or data collection. | No mention of ethical approval, or consent gained from participants. No attempt is made to explain how confidentiality was maintained. | Data analysis commenced with open coding that captured a broad range of perspectives, whereas axial and selective coding facilitated the structuring of a coding framework. After the initial coding framework was developed, the transcribed interviews were imported into N*VIVO, a computer generated data analysis system, and the interview data were coded by multiple coders and subjected to detailed thematic analysis. In the final stage of analysis, constant comparative method of data analysis was implemented to compare categories and themes across respondents. Furthermore, paramedic organisations were consulted when creating the | Findings are presented in themed subheadings. Authors claim to have enhanced credibility through ' prolonged engagement and persistent observation'. No mention of more than one analyst, and qualitative findings were triangulated with quantitative results. | The authors make recommendations for EMS organisations, based on a mixture of their quantitative and qualitative results. |

For peer review only

| | | | | | | | | | |
|---|---|---|---|---|---|---|---|---|---|
| | | | | | | | research questions, and throughout the research project to guide it with feedback. Sufficient data are presented to support the findings, and contradictory data taken into account. | | |
| *Emergency Medical Services Provider Perspectives on Pediatric Calls: A Qualitative Study* Jessica et al. 2019[46] | Aims of study are clearly stated, and their importance justified. Qualitative methodology is appropriate due to nature of the aims, which is to explore subjective experiences in detail | The use of focus groups was not explicitly justified. | 17 EMS providers. ' Paramedics and EMTs were recruited for the study through invitations circulated via weekly staff emails, flyers posted at ambulance bases, the agency's social media page, and e-mails from the EMS chaplain. Interested participants were instructed to contact a single study investigator by phone or email 'Selection criteria were applied to the participants in order to sample for the most relevant participants-participants had to be working for over a year in order to increase chance of | The process of focus group conduction was thoroughly described. A semi-structured guide was used by the researcher to conduct the focus groups- the same researchers conducted all the focus groups. At the end of the focus group, themes were fed back to participants, who were invited to comment. Focus groups were conducted at a large ambulance service. Audio recordings were transcribed. A second researcher was present during the focus group to take notes. Thematic saturation was reached. | The researchers reflect on the potential biases of the study in the limitations section. They acknowledge that by using discussion prompts, they may have steered the conversation away from topics that the participants would otherwise have talked about. Furthermore, they acknowledge that the fact that a single coder was responsible for coding 80% of the data alone may be a source of bias. | Participants gave informed consent and ethical approval was gained from an ethics committee. Resources were provided to the participants in case of experiencing psychological distress. | Transcripts were analysed using directed content analysis. Two reviewers independently coded 20% of the transcripts, and a single researcher coded the remaining data. Findings were validated through groups consensus. Quotations are provided for all sub-themes. Authors acknowledge the limitations of focus groups, and the potential for bias with one researcher coding 80% of the data. The authors also acknowledge how the discussion guide prompts may have altered the topic of discussion of participants from what they would otherwise have | Findings are presented as sub-themes, with quotations supporting each sub-theme in a separate table. Credibility of findings was enhanced by reviewing the focus group themes with participants at the end of the focus group. Furthermore, some of the data was reviewed by two researchers. | The findings are relevant to EMS organisations, specifically EMS leaders. Although Desired support mechanisms following difficult paediatric calls can be extrapolated to other types of traumatic events. |

For peer review only

| | | | | | | | | | |
|---|---|---|---|---|---|---|---|---|---|
| | | | traumatic incident exposure. Non participation was not discussed. | | | | talked about. Contradictory data are taken into account. | | |
| *An assessment of the need of police officials for trauma intervention programmes - a qualitative approach.* Boshoff et al. 2015[57] | Aims of study are clearly stated, and their importance justified. Qualitative methodology is appropriate due to nature of the aims, which is to explore subjective experiences in detail. | Focus groups were chosen as the method of data collection in order to 'allow the researcher to gain insight into participants' beliefs about and perceptions or accounts of a particular topic'. The researchers state: The focus groups allowed the researcher to interact systematically and simultaneously with several individuals. | 40 police officials. Participants were recruited via a purposive sampling method through the health and wellbeing departments, which were used as an intermediary between the researchers and participants. Police officials were purposefully chosen considering the relevance of the topic, specifically referring to their exposure to trauma, resultant symptoms of PTS and their participation in trauma intervention programmes. Non-participation was not discussed, other than reassuring participants that non-participation would not be sanctioned. | Setting for data collection was not outlined. Three focus groups were carried out, and semi-structured interviews were performed within the focus groups. Exact questions asked are provided by the researchers. The sessions were audio recorded, transcribed and analysed. Saturation of the data is not discussed. | Researchers do not critically examine their role, potential bias and influence of research question formulation or data collection. | Ethical approval was gained for this study from the North-West University's ethical committee . Informed consent was taken by the researchers. ' Participants were furthermore encouraged to withdraw from the focus groups at any time should they feel uncomfortable or experience any harm or emotional consequence as a result of participating in the study.' | The authors describe an 8 step method of thematic analysis, but do not explain how they will apply this method. It is not clear how the data is used to arrive at the themes generated. Sufficient data are presented to support the findings. Contradictory data are taken into account. | Findings are presented as sub-themes, supported by sufficient quotations. There is limited discussion for and against the researchers' arguments. No efforts are made to enhance credibility of findings. | The authors give a variety of 'preliminary indicators' for which a ' purposeful psycho-social trauma intervention programme' is indicated. The generalisability of this is limited to police forces. |
| *A preliminary investigation of post-traumatic stress symptoms among firefighters* | Aims of study are clearly stated, and their importance justified. Qualitative methodology is | One-to-one interviews were chosen but reasons for their use over focus groups were not | 11 Firefighters, 8 Station Officers, 4 Sub Officers, 4 Leading Firefighters, 2 Fire Control | ' One-to-one interviews were conducted in a private room with participants. They lasted or up to 90 | Researchers do not critically examine their role, potential bias and influence of research | No mention of informed consent, ethical approval or methods to ensure confidentiality. | 'The interviews were recorded and fully transcribed and the data were analysed by | Findings are presented as sub-headings of themes, with participant quotations | The authors provide recommendations for fire organisations. These |

| | | | | | | | | | |
|---|---|---|---|---|---|---|---|---|---|
| Haslam et al. 2003[60] | appropriate due to nature of the aims, which is to explore subjective experiences in detail. | explained. The interview schedule was developed in conjunction with psychologists working within the fire service, as well using relevant literature. | Officers and 2 Area Divisional Officers. No information provided on how participants were recruited. 'The sample was selected to cover the range of positions in the service and the proportion of respondents in each position broadly reflects the profile of the service.' | minutes. The interview questions were stated by the researchers. Open-ended questions would be used to explore participants' feelings towards incidents. ;The interviews were recorded and fully transcribed and the data were analysed by sorting verbatim material into emergent themes as described by Dey (1993).'' Choice of data collection methods or study setting was not justified. Saturation of data was not discussed. | question formulation or data collection. The researchers acknowledge that their small sample size may not be representative. | | sorting verbatim material into emergent themes as described by Dey (1993). A second researcher independently checked the analysis to ensure analysis reliability' There is little transparency to show how the themes were developed from the primary data. Use of a second researcher enhances credibility. Direct participant quotations are sufficiently used to support the authors' interpretations. Contradictory data are taken into account. | supporting the authors' comments. Credibility is enhanced by the use of two analysts. | recommendations are concerned with efforts to improve staff wellbeing. |
| *A qualitative study about experiences and emotions of emergency medical technicians and out-of-hospital emergency nurses after performing cardiopulmonary resuscitation resulting in death* Fernández-Aedo et al. 2017[47] | Aims of study are clearly stated, and their importance justified. Qualitative methodology is appropriate due to nature of the aims, which is to explore subjective experiences in detail. | Both interviews and focus groups were used as methods of data collection, the reason for their use was not explained. | 7 EMTs and 6 nurses. Snowball sampling was used to recruit the participants. As a prerequisite for their inclusion in this study, the health workers were required to have carried out at least 5 CPR techniques resulting in death over their entire professional career. No volunteer staff | Questions asked during the interviews were ' developed based on the reviewed literature and experts' opinions', but the authors do not provide a topic guide or examples of other prompts. ' The assignment of participants to' either individual semi-structured interviews or focus groups 'was | Researchers do not critically examine their role, potential bias and influence of research question formulation or data collection. | ' The study was approved by the Ethics Committee of the University of the Basque Country.' 'echnique was based on their availability and preferences. Participants gave their written consent to participate in the study and to be recorded in audio and/or video format.' | A detailed description of the analytical process is provided. 'To ensure the quality of the interpretation and guarantee the reliability of the information obtained, a triangulation between all researchers involved in the interviews was also carried out. Any disagreement | Themes are not provided with any participant quotations. 'To ensure the quality of the interpretation and guarantee the reliability of the information obtained, a triangulation between all researchers involved in the interviews was also carried out. Any disagreement | The authors recommend greater training for health professionals when ' notifying bad news and providing psychological support to the family members of the deceased ' No recommendations for research are made. |

For peer review only

| | | | | | | | | | |
|---|---|---|---|---|---|---|---|---|---|
| | | | was included in the study. ' In order to ensure heterogeneity, the study included healthcare professionals of different ages, both genders and varying years of experience, working at different institutions.' Non participation was not discussed. | based on their availability and preferences.' 'A total of 3 health emergency technicians and 3 nurses were interviewed individually for a total of 11---35 min by two of the researchers involved in the study. The focus group was comprised by 4 EMTs and 3 nurses, and it was used to triangulate the information obtained during the individual interviews. The group session lasted 76 min and was carried out by two researchers, one acting as a moderator and the other as an observer.' Data saturation was reached. | | | was resolved by consensus.' No participant quotations are provided to support the authors' interpretations. Contradictory data re not taken into account. | was resolved by consensus.' Other than the use of multiple researchers in the analytic process, no other methods to increase credibility are discussed. | |
| *Experiences of and actions towards worries among ambulance nurses in their professional life: A critical incident study* Svensson et al. 2008[48] | Aims of study are clearly stated, and their importance justified. Qualitative methodology is appropriate due to nature of the aims, which is to explore subjective experiences in detail. | There is no justification for the choice of semi-structured interviews as a method of data collection. | 25 ambulance nurses. Participants were strategically selected from three ambulance services based on 'socio-demographic and professional characteristics such as age, | The authors explain the structure of the interviews, giving examples of the open-ended questions which they ask to participants at the start of the interview. The authors say the | 'As both the researchers and the nurses were familiar with the environment in which the study was conducted, the risk of misunderstanding during the interviews was minimised.' | 'The managers of the ambulance service involved gave approval for the study to be conducted in their department.' The authors say ethical board approval is not required in Sweden under | 'The interviews were also read through several times before categorizing them into sub-categories, in order to further improve security. The incidents were analysed according to | Findings are arranged into themes and sub-themes, and participant quotations are included for each theme to support the authors' conclusions. Credibility was enhanced via the | The authors recommend further research: 'This phenomenon should be studied more in-depth in order to map how the interaction with colleagues influences the worry among |

For peer review only

| | | | | | | | | | |
|---|---|---|---|---|---|---|---|---|---|
| | | | education level, sex and years in the profession'. Non participation is not discussed. | interviews took place where it best suited the participants, and three interviews were conducted over the phone. Researchers explain their use of interviews: 'Interviews were chosen as the data collection method, which allowed the respondents to describe their thoughts in more detail with the help of follow-up questions.' The authors also justify the use of the CIT method. Interviews were audio recorded and transcribed verbatim. Saturation of data was not discussed. | | certain circumstances. The authors describe how informed consent was taken from participants, including assurances that data will be treated confidentially. 'The study adhered to the principles outlined in the Declaration of Helsinki' | character and content. This step was repeated several times before the end result emerged. The categorizing of incidents into sub-categories was conducted in cooperation with the second researcher, experienced both in theory and practice, which minimised the risk of subjectivity (Andersson and Nilsson, 1964). Direct quotes from the interviews strengthened the accuracy of the study.' Contradictory data were taken into account, and sufficient are presented to support the findings. Saturation of data was not discussed. | use of two researchers when categorising incidents. Authors consider contradictory data in the discussion. | ambulance nurses.' Some recommendations are also made for employers of nurses. |
| *Exploring the nature of resilience in paramedic practice: A psycho-social study* Clompus et al. 2016[49] | Aims of study are clearly stated, and their importance justified. Qualitative methodology is appropriate due to nature of the aims, which is to explore subjective | The authors used free association narrative interviewing, a technique which involves a preliminary narrative interview, followed by a semi-structured | 'An advert, with a brief study outline, was placed in a regional Paramedic bulletin which was circulated electronically to staff with an invitation to | The researchers justify their use of using Free association narrative interviewing. Saturation was achieved after the 6th interview. Details of the exact two stages | Researchers do not critically examine their role, potential bias and influence of research question formulation or data collection. | ' Ethical approval was received from a (NHS) research ethics committee and a university in the SW of England. Confidentiality and anonymity was secured through the use of | Details of the FANI analytic process were given by the researchers. 'Any discrepancies were examined and discussed until consensus was reached. Trustworthiness | Themes are presented as sub-headings, and supported by the direct participant quotations. Member validation was carried out to improve credibility. | The authors relate the findings of the research to the theories of exiting organisational interventions, eg TRiM. The authors conclude that for front-line paramedics, ' applying |

For peer review only

| | | | | | | | | | |
|---|---|---|---|---|---|---|---|---|---|
| | experiences in detail. | interview. 'This enabled a deeper analysis of the affective and often unconscious aspects of paramedics' lives. ' | participate.' Participants had to fulfill the following criteria: -Grade of paramedic, technician or emergency care practitioner -Willing to volunteer their time<br><br>Three out of the 10 individuals became unavailable, but the authors do not explain why. | of the interviewing process were provided by the authors. interviews were audio recorded and transcribed, and carried out at a place of the participants' choosing. | | pseudonyms for participants, and all data were kept in a password protected personal computer with access limited to SC. Participants were made aware that they could withdraw at any time and that the anonymised data would be disseminated in various ways. Due to the potential distress that participation could inadvertently provoke, information on how to access counselling services was made available to all participants.' | and data credibility were established by several means including participants feeding into the study's aims, keeping contemporaneous notes, and sending interview transcripts and a summary of findings to each participant for verification'. The authors are not clear how many researchers are involved in the analytic process. For each theme, sufficient participant quotes are provided to support the authors' interpretations. Contradictory data were taken into account and discussed. | | interventions and reviewing support mechanisms would seem to be a pressing imperative.' |
| *First response emergency care – experiences described by firefighters* Abelsson et al. 2019[61] | Aims of study are clearly stated, and their importance justified. Qualitative methodology is appropriate due to nature of the aims, which is to explore subjective experiences in detail. | The authors justify their choice of group interviews, and why limits are set on group interviews: 'The group sizes of eight and nine participants were considered acceptable to moderate and managed by the researcher. | Authors don't explain how participants were recruited into the study. | 35 Firefighters. Setting for group interviews was not given. Participation numbers in the group interviews were provided and discussed. Justification for methods chosen was provided. A rough structure to the interviews was provided by | Researchers do not critically examine their role, potential bias and influence of research question formulation or data collection. | No approval has been sought from an ethics committee, which is in accordance with Swedish law. Consent was implied through participation in the study, following the delivery of verbal 'clear information' by the researchers. | The authors describe their method of text-driven, interpretive qualitative content analysis. This involved repeated readings of the transcribed interviews, and then identification of 'meaning units', then codes were derived, and | Findings are presented as four themes, with consistent use of primary quotations to support the researchers' arguments. The findings are discussed in relation to the original research question. | The researchers contrast their findings with other research in the field, such as in the case of spouses being used as sources of support by firefighters. They also recommend areas of future research, eg involving other strands of |

For peer review only

| | | | | | | | | | |
|---|---|---|---|---|---|---|---|---|---|
| | | Interaction in the pre-existing group is key to a successful group interview. In this study, all firefighters worked at the same fire station, which promoted interactions optimal for the research purpose' | | giving questions which were asked to participants. There is no mention of saturation of the data. The authors reflected in the 'limitations' section that group interviews could lead to participants being uncomfortable. | | Methods taken to protect confidentiality are described. | formed into sub-categories and categories. Sufficient data are present to support the findings. Contradictory data are discussed. It is not clear how many researchers were involved in the analytic process. There is no explicit mention of efforts to improve credibility. Authors acknowledge that 'participants in group interviews may experience a pressure within the group, resulting in similar opinions'. | | emergency service workers. |
| *Paramedics' experiences with death notification: a qualitative study* Douglas et al. 2013[50] | Aims of study are clearly stated, and their importance justified. Qualitative methodology is appropriate due to nature of the aims, which is to explore subjective experiences in detail. | The authors used focus groups as their method of data collection, but did not justify their use over other qualitative methods. | 28 paramedics. Participants were self-selected. Recruitment messages were delivered via 'departmental e-mail, flyers, and by word-of-mouth'. Authors don't explain why the participants selected were the most appropriate for the study, and non-participation is not discussed. | The authors provide the locations of the focus groups, and give examples of questions asked during the sessions. There is no explicit justification for why focus groups were chosen over other qualitative methods. Methods were modified during the study as follows: 'The question guide was adapted as | The researchers acknowledged that their choice of focus group location, as well as the presence of the supervisor in the focus group may have affected the answers given by participants. The researchers do not critically examine their own role. | Ethical board approval was gained, and written consent obtained. No information is provided on efforts to maintain participant confidentiality. | The authors describe an inductive approach to data analysis. Two researchers were involved in the analysis process. Themes were discussed with a further two authors, until consensus was reached. Credibility was enhanced via the use of member checking the participant responses with | The findings are presented as themes, supported by participant quotations and authors' interpretations. Credibility was enhanced by distributing results from the study to participants for the purpose of generating feedback. | The findings are discussed in relation to existing literature, and the authors make recommendations for practice, specifically regarding paramedic training. |

| | | | | | | | | | |
|---|---|---|---|---|---|---|---|---|---|
| | | | | required by the author and the facilitator after each session to clarify some questions based on the paramedics' responses. ' Focus groups were audiotaped and transcribed. Saturation was discussed: the authors kept recruiting participants until saturation was reached. | | | participants at the end of focus groups. Contradictory data are taken into account, and sufficient data are presented to support the findings. | | |
| *Peer-support: a coping strategy for nurses working at the Emergency Ambulance Service* Carvello et al. 2019[51] | Aims of study are clearly stated, and their importance justified. Qualitative methodology is appropriate due to nature of the aims, which is to explore subjective experiences in detail. | The authors used semi-structured interviews. The authors explain their choice of questions result from pre-existing literature, but do not justify the use of interviews over other qualitative methods eg focus groups | 'Participants were recruited on a voluntary basis from an emergency medical service in the north of Italy.' The sampling was 'non-probabilistic', according to the following criteria: nurses working at the emergency ambulance service "118" in a hospital in northern Italy, who had experience in the extra-hospital emergency. Non-participation is not discussed. | Data collection was at an italian emergency service organization called 118. The justification given by the researchers for choosing this location was to choose an environment familiar to the participants so that they would feel at ease. Semi-structured interviews are carried out with participants. The authors explain how they chose their questions, by taking inspiration from similar literature, but choice of | Researchers do not critically examine their role, potential bias and influence of research question formulation or data collection. | Ethical approval was gained from the Bioethical Committee of the University of Bologna. Informed consent was gained from participants before participation. Efforts to protect confidentiality are not discussed. | 'The interviews were conducted and analyzed by all the researchers after having been faithfully transcribed on digital text documents, reporting in brackets some relevant non-verbal gestures, and after having evaluated the nodes and relationships generated by the nVivo qualitative research software12.' Although sufficient data are presented to support the findings, it is unclear how the | Findings are presented as themes. Authors discuss arguments for and against certain issues raised. One example of this is the inclusion of quotations of participants who prefer talking to a peer supporter, and those who prefer talking to a professional therapist. No mention is made of efforts to discuss the credibility of findings. Contradictory data are taken into account and discussed. | The authors relate their findings to their aims. They propose that a peer support program should be introduced in the ambulance service. No recommendations for further research are made. |

For peer review only

| | | | | | | | | | |
|---|---|---|---|---|---|---|---|---|---|
| | | | | interviews over other qualitative methods is not explained. 'The interviews were audio-recorded and conducted anonymously'. Saturation of data is not discussed. | | | themes were generated from the data. | | |
| *Experiences among firefighters and police officers of responding to out-of hospital cardiac arrest in a dual dispatch programme in Sweden: an interview study* Hasselqvist-Ax et al. 2019[63] | Aims of study are clearly stated, and their importance justified. Qualitative methodology is appropriate due to nature of the aims, which is to explore subjective experiences in detail. | The authors don't justify their use of semi-structured interviews over other qualitative methods. | 10 police officers, 12 firefighters. Participants were purposively sampled for knowledge of two or more cardiac arrest situations, and to collect as rich descriptions as possible. Three recruitment approaches were used: '1) an invitation letter from the researchers was presented to the main collaboration group for OHCA alarms in Stockholm County; 2) on the police report for cardiac arrest alarms there was a request to contact the researchers for a voluntary interview; 3) fire stations were directly contacted for recruitment of participants.' Non-participation | The authors provide an interview guide with 7 open-ended questions, which form the basis for the semi-structured interviews. Setting for interviews: all but three took place at regular work places (not known where the others took place). Critical interview technique (CIT) was chosen as the method for data collection. The authors don't justify their use of this technique, but they justify their sample size based on the recommended sample size for CIT. Interviews were recorded and transcribed. The authors state no modification of methods during study were necessary. Saturation was | All interviews were conducted by the same author, to increase the chance that they were conducted in a similar way. The authors list the researchers' relevant strengths and past experiences, demonstrating reflexivity. | Ethical approval was obtained. Written informed consent was obtained from the participants and information was given about the possibility to withdraw from the study without any reprisal. Participants were not entitled to financial remuneration or other benefits. | 'This was an interview study where data were analysed by using critical incident technique (CIT) and inductive qualitative content analysis.' The authors explain the CIT process step by step, and provide an example with a piece of interview data. To enhance credibility, the researchers discussed the analysis at each step of the process, ensuring all analyses were supported by data. Occasionally the views of participants are summarised without examples of quotations. Contradictory data are taken into account, but saturation of data is not discussed. | The findings are presented as themes. Although participant quotations were provided, the authors didn't provide quotations for all of their comments. Often, the views of participants were summarised without providing participant quotes. | The authors provide recommendations for swedish emergency organizations, such as giving indications for training of paramedics and firefighters. |

For peer review only

| | | | | | | | | | |
|---|---|---|---|---|---|---|---|---|---|
| | | | was not discussed. | discussed in relation to the critical incident technique- where 20 interviews (of 2-4 CIs per interview) provide sufficient data. The authors included 22 participants. | | | | | |
| *Working in prehospital emergency contexts: Stress, coping and support from the perspective of ambulance personnel* Oliveira et al. 2019[52] | Aims of study are clearly stated, and their importance justified. Qualitative methodology is appropriate due to nature of the aims, which is to explore subjective experiences in detail. | The authors don't explain why semi-structured interviews were chosen over other qualitative methods. | 14 ambulance personnel. Participants were recruited from seven institutions, mainly on the basis of 'having an active status on a rescue team and being willing to talk and share their experiences'. Details of how participants were recruited are not provided, and non-participation was not discussed. | Interviews took place at the participants' local professional organisation, and were all conducted by the first author. Semi-structured interviews were conducted with participants following an interview guide, details of which are provided by the authors. The choice of semi-structured interviews was justified as it allowed the authors 'to obtain in-depth information regarding participants' prehospital emergency experiences'. Interviews were audio-recorded and transcribed. Saturation of data was achieved. | The researchers acknowledge the possibility of researcher bias during the coding process, and therefore they discuss their analyses with other researchers. | The research project was approved by the Portuguese Red Cross and by the University's Ethics Committee Written consent to participate in the study and to audio-record the interview was obtained, as follows: 'We sent a cover letter to all selected structures, explaining the aim of the study, the procedures, the ethical issues guaranteed, the voluntary character of the participation and the possibility to withdraw at any time'. | The authors use Braun and Clark's analytic approach, and outline the process in detail. This includes explaining how themes were derived from the data. Data collection was performed until saturation was reached. Data was discussed between the researchers during coding, enhancing credibility. The researchers made efforts to reduce researcher bias: 'Furthermore, to reduce research bias, there was a concern to engage with other researchers to discuss the process of data analysis and to illustrate themes with verbatim descriptions from participants.' Sufficient data are | Findings are presented as themes. The researchers provide participant quotations to support their findings. Multiple analysts discussed the analytic process to reduce researcher bias. | The researchers make recommendations to ambulance organisations to improve the psychological wellbeing of their staff. The authors also provide multiple avenues of recommended further research. |

For peer review only

| | | | | | | | | | |
|---|---|---|---|---|---|---|---|---|---|
| | | | | | | | presented to support the findings, and contradictory data are taken into account. | | |
| *Exploring paramedic communication and emotional expression in the workplace after responding to emergency calls* Drewitz-Chesney et al. 2019[53] | Aims of study are clearly stated, and their importance justified. Qualitative methodology is appropriate due to nature of the aims, which is to explore subjective experiences in detail. | The authors don't explain why semi-structured interviews were chosen over other qualitative methods. | 8 paramedics. The recruitment strategy is explained in detail. Participants were recruited over facebook groups. 'Participants were sampled using convenience, then purposive sampling. Convenience sampling enabled initial recruitment. Snowball and quota sampling were the two forms of purposive sampling used.' Authors are not clear on which attributes are sampled for. The authors justified their choice of participants by saying that 'when sufficient information is gleaned from participants, a smaller sample size is required ' | Semi-structured interviews were performed with participants, but the researchers do not justify why interviews are chosen. Interviews were conducted using Skype, and participants could choose to interview via video or audio only. An interview guide is provided. Interviews were audio recorded and transcribed, and data collection was stopped once saturation was reached. | The recruitment messages mention that the researcher previously worked as a paramedic. The researchers acknowledge the possibility of confusion and bias that can arise from this, although this was minimised by the fact that the researcher didn't work with any of the participants. | 'This study received ethical approval from the University of Edinburgh's Usher Research Ethics Group. Each participant provided verbal and written consent. To minimise the risk of psychological impact, participants were never asked to recall specific calls or details. Participants were monitored for signs of distress during each interview (none were noted). BCEHS paramedics have access to three services offering support and counselling. These services were listed on their information letters. At the conclusion of each interview, participants were asked if they wanted a referral to any of the services, which | The authors used constructivist grounded theory in their analytic process. The authors are transparent about how themes are generated from the data.' Credibility was enhanced through triangulation, including interviewing the manager of the BCEHS CIS program, Marsha McCall, and Anonymous, a retired BCEHS paramedic, whom substantiated some participant data . Thick descriptions and a diverse sample contributed to credibility and transferability. An audit trail was maintained throughout the research process which enhanced dependability. Journaling and bracketing enriched reflexivity ' 'Each transcript was read at | Quotations are interspersed among the results which are presented as themes. Credibility was enhanced through the use of triangulation with leading members of the organisation. | The researchers consider their findings in relation to existing literature. They also make various recommendations for emergency service organisations, relating to post-incident organisational interventions that could protect paramedic wellbeing. |

For peer review only

| | | | | | | | | | |
|---|---|---|---|---|---|---|---|---|---|
| | | | | | | all participants declined.' | least three times to improve accuracy and familiarity'. Sufficient data are presented to support the findings, and contradictory data are taken into account. | | |
| *Mental Health in the UK Police Force: a Qualitative Investigation into the Stigma with Mental Illness* Edwards et al. 2020[58] | Aims of study are clearly stated, and their importance justified. Qualitative methodology is appropriate due to nature of the aims, which is to explore subjective experiences in detail. | The authors justify their use of open-ended questions in semi-structured interviews to facilitate meaningful discussion, although the choice of interviews other qualitative data collection methods is not justified. | Five police officers. 'Participants were recruited through a referral from a charity, personal network, police forum and contact from within the police force.' The authors outline the criteria for participant inclusion in the study. They do not however justify their choice for these criteria. Non-participation was not discussed. | Semi-structured individual interviews were conducted using a topic guide, of which the authors provide details. The authors justify their use of open-ended questions. Interviews were audio recorded and transcribed. Data saturation was not discussed. | Researchers do not critically examine their role, potential bias and influence of research question formulation or data collection. | Ethical board approval was obtained, as well as written consent from the participants. The researchers also had measures in place to support participants who experience distress due to participation in the study. | The authors used Braun and Clark's approach of thematic analysis. They outlined this process in detail, giving examples of how they arrived at themes from the data. It is unclear whether multiple researchers collaborated to compare coding. Participant quotations are consistently embedded within the authors' interpretations, and contradictory data are taken into account. The authors are transparent about how themes were generated. | Findings are presented as themes and sub-themes. The authors include contradictory data, and discuss this. Discussion takes place throughout the study within the results section, but there is also a separate discussion section. | The authors identify areas where further research would be valuable, such as barriers to help-seeking in male dominated professions. Recommendation s are also made to police organisations, such as highlighting a need to increase mental health awareness. |
| *Living in Critical Times: The Impact of Critical Incidents on Frontline Ambulance Personnel: A Qualitative Perspective* | Aims of study are clearly stated, and their importance justified. Qualitative methodology is appropriate due to nature of the aims, which is to | The authors don't justify why they chose individual interviews over other qualitative methods. They do however justify their use of some closed questions ( | 21 EMTs, 6 EMCs. The qualitative component of this study followed a quantitative Survey. Participants from the earlier study | Setting for data collection is not stated. The authors give examples of the topics discussed during the interview process. The interview | Researchers do not critically examine their role, potential bias and influence of research question formulation or data collection. | No mention of informed consent, ethical approval or methods to ensure confidentiality. | Thematic analysis was undertaken. 'A random sample of transcripts was read and coded by both authors in order in ensure good reliability and | Findings are presented as themes. For each theme, a range of relevant quotations are provided to support this theme. Credibility | The authors make recommendations to police organisations for post critical incident support for staff. |

| | | | | | | | | | |
|---|---|---|---|---|---|---|---|---|---|
| Gallagher et al. 2007[54] | explore subjective experiences in detail. | to elicit background information and to facilitate comparisons across participants. ) | were asked if they would be willing to participate in qualitative interviews. Non-participation is not discussed. | schedule used was created with the help of a literature review and the findings from the quantitative component of the study. No justification for the choice interview method is given. The interviews were audio recorded and transcribed. | | | validity.' It is not clear how the themes were derived from the data. The authors don't critically evaluate their own role in the analytic process. Contradictory data are taken into account- for example when giving examples of participant quotations with conflicting attitudes. The researchers don't critically examine their own role. Sufficient data are presented to support the findings. | of findings is not discussed. | |
| *Police officers, mental (ill-) health and spoiled identity* Bullock et al. 2018[59] | Aims of study are clearly stated, and their importance justified. Qualitative methodology is appropriate due to nature of the aims, which is to explore subjective experiences in detail. | Researchers justified their use of telephone interviews: '. While telephone interviews are often depicted as a less attractive alternative to face-to-face interviewing, telephones may allow respondents to feel relaxed, more able to disclose sensitive information and there is little evidence that they produce lower quality data ' | 52 police officers, two police staff, four Police Community Support Officers (PCSOs) (four) and one special constable. Participants were recruited from six police constabularies in England and Wales. ‘Individual participants were identified by virtue of their contribution to an online survey on the nature of work-related injury in which | The researchers explain in detail how the telephone interviews were conducted. Open questions were asked by the participants. Saturation of data is not discussed. ‘All interviews were digitally recorded, professionally transcribed and anonymized. ‘ | The researchers acknowledge that the nature of the interview is sensitive, and that the nature of telephone interviews would make participants more comfortable disclosing such information. | ‘ The nature of the interviewing was inevitably sensitive and mechanisms were put in place to mitigate that; for example, interviewees were provided with the contact details of sources of support.’ There is no mention of ethical board approval, informed or methods to protect patient confidentiality. | The authors use Braun and Clark's thematic analysis approach, and give a very brief overview of the approach, but they do not give examples of how themes were derived from the data. The researchers didn't critically examine their own role. Sufficient data are presented to support the findings, and contradictory data are taken into account. Data | The findings are presented as themes. For each theme, the authors use participant quotations as well as relevant literature to discuss the context surrounding the quotations. No efforts to enhance credibility are discussed. | The authors make recommendations to police organisations based on their findings surrounding stigma. No recommendations for research are made. |

For peer review only

| | | | | | | | | | |
|---|---|---|---|---|---|---|---|---|---|
| | | | they identified themselves as willing to participate in a full-length interview'. This study is part of a wider project. Non-participation was not discussed. | | | | saturation is not discussed. | | |
| *'You see a baby die and you're not fine:' a case study of stress and coping strategies in volunteer emergency medical technicians* Folwell et al. 2018[55] | Aims of study are clearly stated, and their importance justified. Qualitative methodology is appropriate due to nature of the aims, which is to explore subjective experiences in detail. | The authors justify their choice of in-depth interviewing technique as they 'hope to gain a deeper understanding of the lived experiences of voluntary EMTs'. | 25 EMTs. Participants were recruited from one county in a Western state. It is not clear how the participants were selected, and non-participation is not discussed. | Interviews were conducted in a private space of the participant's fire department. Justification for choice of interview setting was not provided. In-depth interviews were performed with participants, using an interview guide. This was chosen to "gain a deeper understanding of the lived experiences of voluntary EMTs". Saturation was reached by the end of the study. Interviews were audio recorded and transcribed. | 'The research team consisted of a female Caucasian professor who holds a doctorate and a male Caucasian undergraduate student with three years' experience as a volunteer EMT. While some of the interviews were conducted by both members of the research team, most interviews were conducted by the volunteer EMT'. Other than providing these details, Researchers do not critically examine their role, potential bias and influence of research question formulation or data collection. | Confidentiality was guaranteed by the authors as the transcripts were anonymised. There is no mention of ethical board approval or informed consent. | The authors describe the process of constant comparison analysis. Two researchers independently read all the transcripts, and collaborated to discuss findings. The authors are very transparent about the analytic process. Contradictory data were discussed, such as in the example of participants discussing the pros and cons of CISD. Sufficient data are presented to support the findings. | Findings are presented as themes and sub-themes. 'To enhance credibility, member validation was performed in which a summary of findings and initial interpretations of data were given to five participants to confirm the researchers accurately depicted viewpoints and experiences. To enrich transferability, we provided detailed descriptions of participants and research sites. To improve dependability, we used the same protocol for each interview and documented the process of data collection and analysis. To | The authors make four detailed recommendations to the specific EMS organisation with which the study was involved. The authors also make recommendations for future research. |

| | | | | | | | | | |
|---|---|---|---|---|---|---|---|---|---|
| | | | | | | | | address confirmability, we used direct quotes from participants to support the finding' | |

For peer review only

# Appendix A- Abbreviations and definitions

| Abbreviation | Meaning |
|---|---|
| NHS | National Health Service |
| PTSD | Post-Traumatic Stress Disorder |
| ESW | Emergency Service Worker |
| ESO | Emergency Service Organisation |
| EMS | Emergency Medical Service |
| CISD | Critical Incident Stress Debriefing |
| UK | United Kingdom |
| TRiM | Trauma Risk Management |
| GMB Union | General, Municipal, Boilermakers and Allied Trade Union |
| RTA | Reciprocal Translocation Analysis |
| CASP | Critical Appraisal Skills Programme |
| EMT | Emergency Medical Technician |

| Term | Definition |
|---|---|
| Psychological distress | 'the unique discomforting, emotional state experienced by an individual in response to a specific stressor or demand that results in harm, either temporary or permanent, to the person'[45] |
| Critical incident | 'any event with sufficient impact to produce significant emotional reactions in people now or later'.[9] |
| Supervisor | EMS frontline personnel responsible for overseeing a 'crew' of EMTs/paramedics. *Brown University- Roles And Responsibilities Of Supervisory Staff. [internet] 2021[cited 2021 June 1]. Available from: https://www.brown.edu/campus-life/health/ems/roles-and-responsibilities-supervisory-staff#:~:text=EMS%20Supervisors&text=The%20EMS%20Supervisor%20is%20the,and%20appropriate%20emergency%20vehicle%20operation.* |
| Mental health champion | A member of an organisation raising mental health awareness within the workplace[98] |

| Type of stigma | Definition |
|---|---|
| Anticipated stigma (Perceived stigma) | *'the extent to which people believe they personally will be viewed or treated in a stigmatizing way if their mental health problem or related help-seeking becomes known'* [77] |
| Treatment stigma | 'the stigma associated with seeking or receiving treatment for mental ill health'[76] |
| Internalized stigma | 'holding stigmatizing views about oneself'[76] |
| Public stigma | 'invalidating and unjustified beliefs (i.e., prejudices and endorsed stereotypes) about others'[83] |

For peer review only

# Appendix B: Literature search terms

(((mental AND (health OR well?being)) OR (trauma* OR *stress OR recover* OR PTSD OR post?traumatic?stress OR emotion* OR (critical AND incident) OR (traumatic AND incident) OR (traumatic AND event) OR experience* OR support* OR *support) AND (emergency service* OR emergency medical service* OR EMS OR first responder* OR ambulanc* OR paramedic* OR firefighter* OR fire service* fire department* OR police*) AND (qual* OR mixed?method* OR interview* OR focus?group*) NOT (quality OR emergency?department)

((emergency service* OR emergency medical service* OR EMS OR first responder* OR ambulanc* OR paramedic* OR firefighter* OR fire service* fire department* OR police*) AND (qual* OR mixed?method* OR interview* OR focus?group*) NOT (quality OR emergency?department)) AND ((help-seeking* OR stigma* OR mental* OR barriers OR (MeSH terms: help-seeking behaviour, social stigma, mental health, psychiatry, social support, mental disorders))

For peer review only


For peer review only - http://bmjopen.bmj.com/site/about/guidelines.xhtml

# Appendix C: Data extraction template

Citation
Reviewer
Country
Aims
Ethics – how ethical issues were addressed
Study setting- e.g. type of organisation

Relevant context to study setting

Socio-demographics of the country / region

Recruitment context (e.g. where people were recruited from)

Sampling- what sampling methods, what were inclusion and exclusion criteria,

Data quality rating

Participants- 'population described'

Participants- 'characteristics'- age, sex etc

Theoretical background

Proportion of sample exposed to critical incidents/ definition of critical incident/ anything relevant to the study matching my selection criteria

Definition of critical incidents/ something related

Data collection methods- data collection methods, role of researcher within setting...

For peer review only

Data analysis approach- how many researchers, how did they code, how were findings corroborated,

Themes identified in study (1st order interpretations)- Help-seeking

Themes identified in study (1st order interpretations) – Mental Health recovery

Data extracts related to key themes- Help-seeking

Data extracts related to key themes- Mental health recovery

Author explanation/interpretation of key themes (2nd order interpretations)- Help-seeking

Author explanation/interpretation of key themes (2nd order interpretations)- Mental health recovery

Recommendations made by authors (both outcomes; but specify)

Third order interpretations:

Other potentially relevant information

For peer review only

# Appendix D: Example literature quotations used to construct themes

| | | | |
|---|---|---|---|
| **Factors contributing to mental health recovery post traumatic incident exposure** | **Organisational** | **Time out/Downtime** | *They prefer support to be offered immediately after the call and find that downtime after a stressful call allows them to decompress and prepare for the rest of their shift: '…we knew we weren't going to get a call right, so we knew we had the two hours, so we watched a funny show and had a nap…But…like for me personally… that's exactly…what I needed.' Participant 4 from focus group 4.* Douglas et al. 2013<br><br>*. . . our supervisor took us out of service for a couple of hours and let us go have lunch, sat down and had lunch and just kind of relax and talk amongst ourselves, not even about the call, just about whatever, just to kind of relax. Before we went back on shift. Certainly we could have booked off the rest of the day, you know, on stress leave or whatever, but we all, found that just having, just being able to have a couple of hours to, kind of, you know, relax a little bit, that helps us a lot.' (Focus group #520–522)*<br>Halpern et al. 2008<br><br>*I like didn't want to be like I need to take a couple of hours off because I did not want to look bad in front of the supervisor.-Participant 2 from focus group 2.*<br>Douglas et al. 2013<br><br>*Unfortunately, paramedics often do not have adequate time to talk. Several participants expressed their frustration over 'calls waiting'. These calls are waiting to be dispatched as no crew is available. When BCEHS paramedics offload their patient(s) at the hospital, dispatch can immediately send them to a waiting call. This can limit paramedics' ability to discuss CIs. James desired:*<br><br>*…to be given the time uninterrupted, unbothered… Give us our time to decompress.*<br>Drewitz-Chesney et al. 2019 |
| | | **Supervisor** | *Depending on who it is . . . one supervisor, we took, we did a [critical call], and we took the last hour of the shift off. And he was making us fill out all these forms and, you know, telling us that if we wanted to take the next shift off that we had to go see our doctor and get a note for this and that. And you know, just made it more stress-(Focus group #520–522)*<br>Halpern et al. 2008<br>*'And so our supervisor was really, we have a great supervisor and he's, you know, asked us all specifically, "are you guys okay", you know. And the other crew went off on stress for the rest of the shift. We stayed, because we said, y ou know, we're okay. We just kind of dealt with the aftermath of everything. It was still a pretty stressful call but at least we had that option. And he had no problems, like, he said, go home. Whatever you guys need. So and that's a big thing.- (Focus group #520– 522)*<br>Halpern et al. 2008 |



For peer review only - http://bmjopen.bmj.com/site/about/guidelines.xhtml

| | | | |
|---|---|---|---|
| | | | *Supervisors, however, were perceived differently. Some paramedics reported feeling unsupported when their supervisors questioned why they were reacting to an event that they did not perceive as traumatic:*<br>*I wasn't really involved in a traumatic event because there was no loss of life, or you know what I mean, um, it, there wasn't really much sympathy the, um, I could, not that I really, um, oh, this wasn't, um, a huge accident, you weren't injured, you know, really why are you complaining?*<br>Regehr et al. 2007<br><br>*he's [supervisor] quite an old fashioned sort of police officer, not the bloke you would sort of want to go in and have a chat with about a sudden death you'd just been to ... If I went in and said 'Governor, can I have a chat about the sudden death?', he'd look at me as if I'd just asked to kill one of his children!*<br>Evans et al. 2013 |
| | | **Peer support network** | *Nurse 1 "Therefore, talking to a person who has the same skills, abilities and knowledge you have would probably be more meaningful".*<br>Carvello et al. 2019<br><br>*The majority of nurses is in favour of peer-supporters. The motivation is based on the fact that they recognise the peer-support as someone that can understand what nurses really mean when relating a traumatic event, being one of their colleagues*. Carvello et al. 2019 |
| | **Infor mal suppo rt** | **Colleague s and family** | *The more you talk about something, the more it becomes something you've told and your telling becomes part of the memory, as opposed to it being a really shiny, vivid thing inside your head*those images.*<br>Evans et al. 2013<br><br>*I don't think talking about it to people at work is the release, the escape I need ... it's speaking to people who I care about and who care for me and just having that comfort zone, that's what's important to me. [P16]*<br>Evans et al. 2013<br><br>*... I never tell my wife that, I would never tell her that because I just think that would have really put the frighteners on her. [P15]*<br>Evans et al. 2013 |
| | | **Regular partner** | *For me it's just always been that partner, because they're right there with you and they'll know what's going on, and you really want somebody that can understand what's happening.*<br>*Jessica et al. 2009*<br><br>*...for those of us that have regular partners, regular stations, rely on your partner… They're gonna know if something's up with you.*<br>Drewitz-Chesney et al. 2019<br><br>*Paramedics working with different partners may be unable to recognise changes as readily as they would in a regular partner. When paramedics are without a regular partner or are uncomfortable speaking with their* |

For peer review only

| | | | |
|---|---|---|---|
| | | | *current partner, they often don't discuss calls. Instead, some participants said they speak with long-time paramedic friends or coworkers with whom they have similarities, ie. age or training level.*<br>Drewitz-Chesney et al. 2019<br><br>*…if I'm working with my regular partner, those communication lines are very, very open and we can talk about the tough calls, how we're feeling, if there's anything we would have done different [sic] (Sean).*<br>Drewitz-Chesney et al. 2019<br><br>*I really envy the paramedics that have regular partners that they know, and they trust, and they can talk to (Krista).*<br>Drewitz-Chesney et al. 2019<br><br>*…I would have a regular partner and that partner would be someone who is just like family to me. We would just talk about everything, without even hesitating…'*<br>Drewitz-Chesney et al. 2019 |
| | | **Reassurance and validation** | *I remember going to the hospital and I remember I was disappointed because the parents weren't there at that time...And I don't know what it was. It was just something that made me feel like I just needed to talk to them. But I never ended up talking to them. So anyway that call definitely stands out as being...(Stuck with you.) Yeah.*<br>Halpern et al. 2009<br><br>*And it's weird, I looked in the newspaper. I saw his funeral announcement and I went to the funeral. (Oh okay.) I didn't talk to any of the family. I just went for my own, I don't know why ... I, for some reason, I guess I felt I needed to follow up and so I did. And I sent the family a condolence card and then a friend of mine ... met a friend of the family's friend and he had said, oh, his [relative] wants to talk to you ... So she called me and she wanted to know, like, you know, what happened with her [relative] and who was the last one to talk to him ... It's kind of funny because there are so many calls you do and you never think about them again. But this one, I thought about him a lot. I guess because he was so young and again, I really didn't think he was going to die. So I thought, okay, well, you know, he's at the trauma centre now and I had no idea that he was that badly injured. And then he died. So I think that freaked me out. I wasn't prepared for that. And I thought, oh my God, he's so young and his family wasn't there. I felt badly his family wasn't there and then. So that one stuck with me for a while.*<br><br>*Interviewer: "And talking to his [relative], did that help or …?"*<br><br>*It more, I felt it was helping them so it made me feel better, because she really, really was upset. And I felt she wanted, I think she felt a lot better after talking to me and hearing something about what happened. And so it made me feel better to give them some kind of closure.Pg 181-182*<br>Halpern et al. 2009 |



For peer review only - http://bmjopen.bmj.com/site/about/guidelines.xhtml

| | | | |
|---|---|---|---|
| | | | *. . . the day after that call, my supervisor kind of sensed that I wanted to talk to him and I don't know why, you know, he came to the hospital. But it was the next day and he was kind of like, so pull up a chair, sit down. Let's just talk. And it was like I wanted somebody just to say to me, okay, this is your time and you can talk right now and I'm not going to judge you. I'm not going to talk about anything. I'm going to let you just have your 10 minutes. And that made a huge difference. I have the utmost respect for my particular supervisor . . . Whether it was talking specifi cally about the call or just having a little bit of a "hey you're okay at your job", type comment. (Participant #122)*<br>Halpern et al. 2008 |
| **Factors influencing help-seeking behaviour** | **Nature of intervention delivery** | **Mandatory vs non-mandatory** | *We had counselling every six months ... and everybody used to go 'Oh I've got to see the counsellor this week', but I tell you what ... we all quite enjoyed it ... I was so much calmer after speaking to her but it's something I'd never have done had I not been made to do it. [P12]*<br>Evans et al. 2013<br><br>*My emotions are none of your business and if I wanted to share my emotions with you, I'm going to share [them] with someone I trust… Participant 1 from focus group 1.*<br>Douglas et al. 2013 |
| | | **Shared experiences with intervention provider** | *FRs in our study preferred a MH professional with experience as an FR or military veteran. Many also approved of a provider that "knew the job," either working with multiple FRs in the past, or even as a family member.*<br>Jones et al. 2020<br><br>*I've been to [therapy] a couple of times. . . . The guy that I got was excellent, but I only think it was excellent because he was prior military (P8, Firefighter ×22 years).*<br>Jones et al. 2020 (study 4)<br>*…someone I think understands what's going on, and has been through what I've done.- Participant 3 from focus group 4.*<br>Douglas et al. 2013 |


For peer review only - http://bmjopen.bmj.com/site/about/guidelines.xhtml

| | | | |
|---|---|---|---|
| | **Stigma as a help-seekingbarrier Specific barriers** | **'Macho culture'** | *I think there's a real element of machismo and masculinity in the police force and it's a bit, sort of a faux pas to admit that things have really affected you ... If I'd have come out and said 'ah you know, that really affected me badly, let's go and sit down and have a cup of tea and talk about it' I think you're straying into pink and fluffy territory there ... saying 'that made me feel sad' is a bit too far. [P3]*<br>Evans et al. 2013<br><br><br>*Everyone wants to be tough and strong. Maybe that was my downfall or problem at the time and I didn't want to admit that I needed any kind of help. I guess I didn't want to be perceived as weak. (Participant #110)*<br>Halpern et al. 2008<br><br>*… that's so ridiculous. You need to harden up if you want to do this job, and old people die.*<br>Drewitz-Chesney et al. 2019<br><br>*… in metro, there's a lot more...bravado and joking around about things…If you can get one on one with someone, they're usually a lot more receptive and a lot more empathetic- (Dennis).*<br>Drewitz-Chesney et al. 2019<br><br>*Unfortunately there's a massive stigma […] one of my sergeants the other week, there was mental health training coming up and his reaction to being put forward to go on the training was, 'Well what do I want to go and learn how to deal with a load of nutcases for?*<br>Bullock et al. 2018<br><br>*We are many women at the station so its easier for us to talk about what happened…You have to talk about what happened otherwise you can't go on. It's easier than on a station with only men, it's not necessary with any "macho style" so I think it's easier for the men (at our station) to talk about things you must talk about. (A female nurse describes her feelings.)*<br>Jonsson et al. 2003 |
| | | **Stigma and shame** | As an institution, they were very very good, in getting me better, at making me understand, but back at work that was a different ball game. You've got your colleagues who are still at work, still running around like idiots and they're like, oh fucking hell, you've had three months off, you know, I should have gone off with stress. (Participant 1).<br>Edwards et al. 2020<br><br>*There have been officers that are doing the shift that have shown that they can't deal with situations like that, and been very open about it*and they haven't got the respect from the shift, because the colleagues go 'well, you're on your own if you're working with her, because she'd back away' or whatever. So you don't want to be considered as one of those. [P7]*<br>Evans et al. 2013 |
| | | **Career concerns** | *I think that the stigma is you have to be very careful who you tell that it bothered you or you might get judged as weak or you might get fired" (P2, EMT/paramedic ×20 years).*<br>Jones et al. 2020<br><br>If you've a form of mental health illness you will not get on; you will not be promoted, …people will not want you on their section. (Participant 4).<br>Edwards et al. 2020 |



For peer review only - http://bmjopen.bmj.com/site/about/guidelines.xhtml

| | | | |
|---|---|---|---|
| | | | I feel scared to declare anything or do anything about anything because will it bite me later on in life? Will it prevent me from doing something in the police later on? Could it be used against me? … will it be used in a negative way later on?<br>Bullock et al. 2018 |
| | | **Confidentiality concerns** | *I know our department's very, very poor at keeping secrets. So if I put a crew out of service, I have to tell the communications center. Communications says, we're sitting this far apart, Hey X, I just put the 22 car out of service in stress. Every dispatcher in there hears it. Every call receiver in there hears it. So they hear it. I don't know if they say anything. I don't know if they go home and tell all their friends and family. But I don't like that system. There's no quiet way of doing it. (Participant #128)*<br>Halpern et al. 2008<br><br>*I think that the stigma is you have to be very careful who you tell that it bothered you or you might get judged as weak or you might get fired" (P2, EMT/paramedic ×20 years).*<br>Jones et al. 2020<br><br>*.. .to whom must I speak in the police if I can't trust anyone. So now I'm seeking professional help outside the police .*<br>Boshoff et al. 2015 |
| | **Mental health literacy** | **Emotional awareness** | *Some appreciated that difficulties in recognizing and admitting to distress pose significant barriers to accessing support. Recognizing the emotional impact of critical incidents may help to address these barriers.*<br>Halpern et al. 2009<br><br>*I didn't recognise it as what it was; I just thought I was grumpy…you don't see them creeping up, and in the end, the thing that tips you over the edge, the thing that makes your bottle overflow if you like can be something quite small because you've got used to dealing with stuff. (Participant 5).*<br>Edwards et al. 2020 |
| | | **Education and stigma** | *I think for, for everybody, is, here's my thought towards your process, is giving them the tools. People are going to be very tough and say, yeah, yeah, fine. But you know if you could somehow identify the emotions that go along with these calls that might be starting to put you on tilt...Then you can teach people to be aware of them and say, hey, you know what, it's okay to say, I need to talk to someone.*<br>Halpern et al. 2009<br><br>*In particular, it would appear from this study that teaching ambulance personnel about the emotional aspects surrounding different types of critical incident may diminish their confusion about which incidents they can expect to impact them.*<br>Halpern et al. 2009<br><br>*I guess that's the main two [barriers], pride and then denial. . . . But, it's just education. Just letting [FRs] know, look, these things are normal. It's going to happen to somebody" (P11, Firefighter ×8.5 years).*<br>Jones et al. 2020 |


For peer review only - http://bmjopen.bmj.com/site/about/guidelines.xhtml

| | | | |
|---|---|---|---|
| | | | *Knowing that other people are there dealing with that same stuff. You can bounce ideas off each other, see what's worked in their situations and what hasn't . . . you realize you're not crazy (P12, EMT/paramedic ×14 years).*<br>Jones et al. 2020<br><br>*If you could actually get people in in front of officers saying, 'I was one of those people that didn't believe stress could ever get to that level and it was ridiculous and you just needed to work harder', maybe officers would accept that from another officer more than just somebody standing in front of you training because it's getting officers to accept that actually it's okay, you're only human and your body and your mind can only take so much, and maybe they'd accept it more then.*<br>Bullock et al. 2018 |

For peer review only

For peer review only


For peer review only - http://bmjopen.bmj.com/site/about/guidelines.xhtml

BMJ Open

# Mental health and help-seeking among trauma-exposed emergency service staff: A qualitative evidence synthesis


| | |
|---|---|
| Journal: | *BMJ Open* |
| Manuscript ID | bmjopen-2020-047814.R2 |
| Article Type: | Original research |
| Date Submitted by the Author: | 15-Oct-2021 |
| Complete List of Authors: | Auth, Niklas; University of Birmingham, College of Medical and Dental Sciences<br>Booker, Matthew; University of Bristol, School of Social and Community Medicine<br>Wild, Jennifer; University of Oxford, Experimental Psychology<br>Riley, Ruth; University of Bristol, Social and Community Medicine; University of Birmingham, of Applied Health Research |
| <b>Primary Subject Heading</b>: | Occupational and environmental medicine |
| Secondary Subject Heading: | Mental health, Medical management, Qualitative research |
| Keywords: | QUALITATIVE RESEARCH, MENTAL HEALTH, OCCUPATIONAL & INDUSTRIAL MEDICINE, Health policy < HEALTH SERVICES ADMINISTRATION & MANAGEMENT |
| | |


SCHOLARONE™
Manuscripts

*I, the Submitting Author has the right to grant and does grant on behalf of all authors of the Work (as defined in the below author licence), an exclusive licence and/or a non-exclusive licence for contributions from authors who are: i) UK Crown employees; ii) where BMJ has agreed a CC-BY licence shall apply, and/or iii) in accordance with the terms applicable for US Federal Government officers or employees acting as part of their official duties; on a worldwide, perpetual, irrevocable, royalty-free basis to BMJ Publishing Group Ltd ("BMJ") its licensees and where the relevant Journal is co-owned by BMJ to the co-owners of the Journal, to publish the Work in this journal and any other BMJ products and to exploit all rights, as set out in our licence.*

*The Submitting Author accepts and understands that any supply made under these terms is made by BMJ to the Submitting Author unless you are acting as an employee on behalf of your employer or a postgraduate student of an affiliated institution which is paying any applicable article publishing charge ("APC") for Open Access articles. Where the Submitting Author wishes to make the Work available on an Open Access basis (and intends to pay the relevant APC), the terms of reuse of such Open Access shall be governed by a Creative Commons licence – details of these licences and which Creative Commons licence will apply to this Work are set out in our licence referred to above.*

*Other than as permitted in any relevant BMJ Author's Self Archiving Policies, I confirm this Work has not been accepted for publication elsewhere, is not being considered for publication elsewhere and does not duplicate material already published. I confirm all authors consent to publication of this Work and authorise the granting of this licence.*

For peer review only

# Mental health and help-seeking among trauma-exposed emergency service staff: A qualitative evidence synthesis

Word count: 6320

For peer review only

**Corresponding Author:**
Niklas Auth
University of Birmingham
Tel: 07475545757
Email: nma676@student.bham.ac.uk

Matthew Booker
GP & NIHR Clinical Lecturer in Primary Care (Urgent Care)
Centre for Academic Primary Care (CAPC)
Bristol Medical School
University of Bristol

Jennifer Wild
Associate Professor
Department of Experimental Psychology
Oxford Centre for Anxiety Disorders & Trauma
University of Oxford


For peer review only - http://bmjopen.bmj.com/site/about/guidelines.xhtml

Ruth Riley

Lecturer in Applied Health Research

College of Medical and Dental Sciences

University of Birmingham

For peer review only

## Abstract

**Objectives**

To identify factors and contexts that may contribute to mental health and recovery from psychological difficulties for emergency service workers (ESWs) exposed to occupational trauma, and barriers and facilitators to help-seeking behaviour among trauma-exposed ESWs.

**Background**



For peer review only - http://bmjopen.bmj.com/site/about/guidelines.xhtml

ESWs are at greater risk of stressor-related psychopathology than the general population. Exposure to occupational stressors and trauma contribute to the observed rates of post-trauma psychopathology in this occupational group with implications for workforce sustainability. Types of organisational interventions offered to trauma-exposed ESWs are inconsistent across the UK, with uncertainty around how to engage staff.

**Design**

Four databases (OVID MEDLINE, EMBASE, PsycINFO and SCOPUS) were systematically searched from January 1 1980 to March 2020, with citation tracking and reference chaining. A modified Critical Appraisal Skills Programme tool and quality appraisal prompts were used to identify fatally flawed studies. Qualitative studies of trauma-exposure in frontline ESWs were included, and data was extracted using a customised extraction table. Included studies were analysed using thematic synthesis.

**Results**

A qualitative evidence synthesis was conducted with 24 qualitative studies meeting inclusion criteria, as defined by the PerSPEcTiF framework. Fourteen descriptive themes emerged from this review, categorised into two overarching constructs: factors contributing to mental health (such as the need for downtime, peer support and reassurance) and factors influencing

For peer review only

help-seeking behaviour (such as stigma, the content, form and mandatory nature of interventions, and mental health literacy (i.e. emotional awareness, education).

## Conclusion

ESWs reported disconnect between the organisations' cultural positioning on trauma-related mental health, the reality of undertaking the role, and the perceived applicability and usefulness of trauma interventions. Following traumatic exposure, ESWs identify benefitting from recovery time and informal support from trusted colleagues. A culture which encourages help-seeking and open dialogue around mental health may reduce stigma and improve recovery from mental ill health associated with trauma exposure.

For peer review only

**Keywords:** Qualitative research, mental health, emergency service workers, thematic synthesis, psychological trauma, qualitative evidence synthesis

## Strengths and Limitations

-This is the first qualitative evidence synthesis of traumatisation and mental health help-seeking in ESWs.

- A user advisory group of ambulance management stakeholders and ESWs was involved in the design and purpose of this research.

-Findings are drawn from pre-Covid literature however core themes are omnirelevant.

- Study quality varied significantly and there is a predominance of ambulance service literature in included studies.

For peer review only

For peer review only

## Introduction

Emergency service workers (ESWs) consisting of members of the emergency medical services (EMS), the fire service and the police force, consistently experience poorer mental



For peer review only - http://bmjopen.bmj.com/site/about/guidelines.xhtml

health outcomes when compared to the general population. While subtle differences exist between occupational groups within emergency service organisations (ESOs), ESWs, also referred to as first responders, experience disproportionately higher rates of post-traumatic stress disorder (PTSD), anxiety, depression and psychological distress.[1-5] Suicide attempts by ESWs are considerably more prevalent than the estimated rate of 0.5% in the general population.[6] Exposure to traumatic events accounts for higher PTSD rates in the ESW cohort,[2] and is the second most commonly reported cause of poor mental health among UK ESWs in a 2019 survey, following excessive workload.[7] This review will consider the terms 'traumatic incident/event' synonymously with the term 'critical incident', which is defined as: 'any event with sufficient impact to produce significant emotional reactions in people now or later', as described by Mitchell and Everly.[8] The increased incidence of PTSD in ESW populations[1,2] is important to appreciate as PTSD is a risk factor for suicidal ideation and risky behaviours in civilian and military populations,[9,10] and increases suicidal risk in ESWs.[11,12] The wider impacts of mental ill health among ESWs include high rates of absenteeism and presenteeism, resulting in significant costs to emergency service organizations.[13]


For peer review only - http://bmjopen.bmj.com/site/about/guidelines.xhtml

ESOs employ a variety of programmes to prevent the development of mental ill health in trauma-exposed staff, of which main categories include stress management, psychotherapy and health promotion.[14] Single session critical incident stress debriefing following trauma-exposure is a psychological intervention with widespread historical and current use in ESOs.[15-17] However, by the early 2000s, a substantial body of evidence demonstrated that CISD was neutral at best and harmful at worst with respect to preventing PTSD; it appears that CISD interferes with natural recovery.[15,16,18-20] The following two interventions are emerging among UK ESOs, and are examples of prevention strategies hoped to replace traditional debriefing methods. Trauma Risk Management (TRiM) is a peer support system, delivered by trained volunteers within the organisation,[21] who assess trauma exposed individuals for risk of mental ill health.[22,23] The evidence surrounding TRiM's impact on users' mental health outcomes or for improving attitudes to mental ill health is inconclusive.[23-26] Schwartz rounds, best described as a cultural change initiative, also emerging in the UK,[27] allow multidisciplinary healthcare staff to share and discuss non-clinical aspects of their work, such as psychosocial, ethical and emotional issues.[28] As of May 2020, Schwartz rounds have been adopted by four UK ambulance trusts.[27] Schwartz rounds are reported to improve staff psychological wellbeing and increase 'empathy and compassion

For peer review only

for colleagues',[29] although they have not been formally evaluated. Other interventions provided by UK ESOs to support the emotional wellbeing of ESWs after attending to critical incidents include counselling and 24-hour helplines,[30-34] 'defusing' programmes[35] which require staff who attended a critical incident to discuss facts surrounding the event in a structured group format, and peer support networks.[36]

Despite the availability of interventions, ESWs experience barriers to mental health care with one third reporting that they experience mental health stigma, a rate that is higher than the general population.[37] The purpose of this review is to identify factors and contexts that may contribute to mental health and recovery from psychological difficulties experienced by emergency service workers (ESWs) exposed to occupational trauma. We were also interested in identifying barriers and facilitators to help-seeking behaviour among trauma-exposed ESWs.

When discussing methods aimed at improving mental health, 'protection' will be used in the context of trauma-exposed ESWs, while 'recovery' relates to ESWs experiencing psychological distress as a result of trauma-exposure. By identifying important contextual factors which help and hinder staff when they access support, and illuminating benefits and drawbacks of current organisational interventions, this review aims to offer qualitative


For peer review only - http://bmjopen.bmj.com/site/about/guidelines.xhtml

insights grounded in the perceptions of ESWs, which may help ESOs in decision-making about psychological support for their staff following traumatic incident exposure.

## Methods

Methodology

Qualitative evidence synthesis (QES) is a recognised method of integrating primary qualitative research findings in health and social care.[38] The methods of this QES are reported using the ENTREQ framework (Supplementary File 1). The research question and final search terms were ratified by a consensus panel of key stakeholders drawn from UK ambulance services and user advisory group of ESWs. This group contributed to the development and refinement of the review questions, search parameters and application of the review findings.


For peer review only - http://bmjopen.bmj.com/site/about/guidelines.xhtml

Eligibility criteria

In keeping with qualitative review guidance[38], the PerSPEcTiF framework was used to enhance description of inclusion criteria.[39] (Table 1)

**Table 1: The PerSPEcTiF question formulation framework[39]**

| Perspective | Setting | Phenomenon of interest/Problem | Environment | (Optional comparison, not applicable) | Time/timing | Findings |
|---|---|---|---|---|---|---|
| Emergency service workers | Emergency frontline ambulance, police or fire service work | Factors influencing mental wellbeing and help-seeking behaviour | Poor mental health outcomes and elevated rates of mental health stigma within the emergency services | | Following occupational exposure to traumatic event(s) | Emergency service workers' perceptions and experiences regarding the phenomenon of interest |

**Inclusion criteria:**

(* indicates further information below)

(1) Study participants were frontline ESWs (studies with mixed populations of eligible participants were included); (2) The study focus was work-related psychological distress*; (3) Data collection included primary qualitative interviews, focus groups or observational methods (this included mixed methods studies with qualitative components); (4) Analysis



For peer review only - http://bmjopen.bmj.com/site/about/guidelines.xhtml

focussed around participant attitudes towards: a) behaviour aimed at improving or protecting mental health after experiencing a traumatic event OR b) factors which ESWs find helpful or unhelpful for their mental health while experiencing work-related psychological distress; (5) Published in English and peer-reviewed.

No limits were applied to publication date or study location.

**Exclusion criteria:**

(1) Due to the unique nature of the traumatic events witnesses in this cohort,[40] studies investigating a military cohort were excluded; (2) Volunteer ESWs were not included.

*The term ‘psychological distress will be referred to according to Ridner’s definition (see Appendix A).[41]

Search strategy

Systematic searches of the following four databases were conducted by the primary author (NA) from January 1 1980 to March 2020: OVID MEDLINE, EMBASE, PsycINFO and


For peer review only - http://bmjopen.bmj.com/site/about/guidelines.xhtml

SCOPUS. The first three databases were searched together using the MEDLINE database. In order to avoid duplicates, the SCOPUS database was then searched with an additional filter to exclude MEDLINE results. One reviewer (NA) screened the 13381 article titles and/or abstracts identified by the search. The 42 full text articles identified in this process were then independently assessed for their eligibility criteria by two reviewers (NA and RR), with the aid of RefWorks reference management software. During this process, the two reviewers agreed to exclude 17 studies which did not meet the eligibility criteria. In cases of uncertainty surrounding eligibility criteria, there was a group discussion between all authors. One additional study was excluded on the grounds of low methodological study quality; this was agreed upon by all authors. (Figure 1)

To identify articles missed in the electronic database search, the following methods were employed: 1) Using 'related article' feature (when available), 2) Searching the titles of included studies in google scholar for citation tracking purposes, and 3) Manual searching of the references of relevant studies (reference mining).

For peer review only

Grey literature was searched during background research for context. Two combinations of search criteria (see appendix B) were entered into each database in order to locate relevant literature relating to help-seeking and mental health recovery. The search terms were developed during a process of trial and error using qualitative guidance,[42] other reviews in the field,[37,43] and virtual consultations with stakeholders and co-authors.

Quality appraisal

One reviewer (NA) assessed study quality using the CASP qualitative checklist.[44] Studies scoring less than five were further appraised independently by one of three reviewers (RR, MB, JW) to determine whether they should be included in this review based on their conceptual richness, drawing upon five quality appraisal prompts as recommended by Dixon-Woods et al.[45] One study was removed during this approach[46] and in keeping with this approach, 'signal' (the relevance of papers to the review's aims) was prioritised over 'noise' (the inverse of methodological quality).[45]


For peer review only - http://bmjopen.bmj.com/site/about/guidelines.xhtml

Data extraction

A customised data extraction template (See Appendix C) was created using qualitative data extraction guidance[42] and that of a similar review as a template.[43] In keeping with a thematic synthesis approach,[47] first order constructs (direct participant quotations) as well as the author's interpretations were extracted in separate sections to allow for a more comprehensive review.[42] Following Thomas and Harden,[47] all data relevant to the research aims were extracted into templates by one researcher (NA).

Data synthesis:

The analytical technique of thematic synthesis is an established method in the field of mental health to investigate barriers to help-seeking, and to synthesise qualitative evidence about participant experiences.[37,43,48] Thematic analysis uses inductive line-by-line coding and is focussed around: intervention need, appropriateness, acceptability and effectiveness.[49] It is therefore appropriate given that the purpose of the review is to inform UK ESO guidelines. The extracted text underwent line-by-line open coding, which allows new codes to emerge from the data, rather than imposing a pre-existing framework onto the extracted data.[47] Subsequently, first-level codes were assimilated according to their meanings, similarities and



For peer review only - http://bmjopen.bmj.com/site/about/guidelines.xhtml

differences. The codes were then arranged in a ‘hierarchical tree structure’ during which some codes were renamed as new meanings were generated. For example, some quotations initially coded as ‘stigma’ were later agreed to more appropriately reflect ‘macho culture’ or ‘career concerns’, with ‘stigma and shame’ relating specifically to shaming practices. This inductive process results in ‘descriptive themes’. Two reviewers (NA and RR) performed the initial coding process, but grouping into descriptive themes and interpretation of the data involved discussion between all four authors. To enhance transparency, primary quotations used to construct the themes are supplied in Appendix D. In the final stage of thematic synthesis, the descriptive themes were used to address the review questions. This process involves inferring the meaning behind the data. The final result of this process was the translation of the descriptive themes into implications for ESO wellbeing policy practice, which involved roundtable discussion between all four authors.

## Patient and public involvement statement

Patients were not involved the design, conduct, dissemination or reporting of this research. However, a user advisory group of ambulance management stakeholders and ESWs was consulted regarding the design and the purpose of the research and advised on questions to

For peer review only

include. We believed patient and public involvement to be of reduced relevance as the impetus for this review derived from the Association of Ambulance Chief Executives, who wished to address an evidence gap to inform policy decisions.

# Results

<<insert Figure 1 here>>

*Figure 1: Moher D, Liberati A, Tetzlaff J, Altman DG, The PRISMA Group (2009) Preferred Reporting Items for Systematic Reviews and Meta-Analyses: The PRISMA Statement. PLoS Med 6(7): e1000097*

For peer review only

Overview of study characteristics

24 studies were included in this review (Table 2). All but two studies[50,51] employed qualitative methodology only. For these two mixed methods studies, only data from sections related to their qualitative methodology were extracted. The majority of studies (16 of 24) employed a sample of ambulance personnel[50-65] followed by police officers[66-69] and lastly



For peer review only - http://bmjopen.bmj.com/site/about/guidelines.xhtml

firefighters.[70,71] The participants of one study, in addition to ESWs, included participants who did not match the selection criteria[70], and two studies included a mix of different types of ESWs.[72,73] These studies were still eligible as the participant quotations were labelled with an occupational identifier to make it possible to differentiate between eligible and non-eligible participants. The objectives of studies varied widely in terms of relevance to our review aims. Ten of the 24 studies included data relevant to protecting post-traumatic incident mental health,[51-53,55-58,61,65,73] and one study included data relevant to barriers and facilitators for help-seeking behaviour related to mental health.[72] The remaining 13 studies[50,54, 59,60,62-64,66-71] included data which were extracted for both of these outcomes.

**Study quality**:

Study quality varied significantly with data collection, data analysis and discussion of findings being adequately explained in the majority of studies. All studies provided adequate research aims, although fewer than half sufficiently justified the research design. [51,53,55, 59, 60,65,66, 67,69,71,72] The lowest scoring domains were recruitment and demonstrating reflexivity. In few studies the researchers adequately justified their selection of participants[51,55,57,67,72,73]

For peer review only

or critically examined their relationship with participants. [66,58,62,63,66,72,73] Four studies failed to present ethical considerations. [50,51,64,70]

The full CASP appraisal table of all included studies is provided in online supplementary file 2. Following CASP appraisal, six studies[49,55,57,61,64,70] initially identified as weaker quality underwent further independent quality appraisal by one of three reviewers (RR, MB, JW), during which process one study was excluded.[49]

This qualitative review identified 14 descriptive themes, which are arranged in groups of higher order themes. In turn, these are grouped into one of the following two overarching constructs: ‘factors contributing to mental health’ or ‘factors influencing mental health help-seeking behaviour’. Table 3 presents a summary of hierarchical thematic structure.

**Table 2: Study characteristics**

| Title of study (Authors, year) | Quality ranking against CASP criteria | Participants | Study aim(s) | Country | Method of data collection | Analysis method |
|---|---|---|---|---|---|---|
| What makes an incident critical for ambulance workers? Emotional outcomes and implications for intervention. Halpern et al. 2009 | 5/9 | N=60<br>4 supervisors, 54 front-line ambulance workers | To characterize critical incidents as well as elicit suggestions for interventions | Canada | Focus groups/ individual interviews | Ethnographic content analysis |
| Police officers' experiences of supportive and unsupportive social interactions | 6/9 | N=19<br>Police officers | (1) What are police officers’ experiences of supportive and unsupportive interactions following | England | Semi-structured interviews | Thematic analysis |



For peer review only

| | | | | | | |
|---|---|---|---|---|---|---|
| following traumatic incidents. Evans et al. 2013 | | | potentially traumatic incidents? (2) Do interactions differ on the basis of the context and source of support (i.e., at work with colleagues and supervisors, or outside of work with family and friends)? (3) How do supportive/unsupportive interactions facilitate/hinder the processing of traumatic incidents? | | | |
| Interventions for critical incident stress in emergency medical services: a qualitative study Halpern et al. 2008 | 6/9 | N=60 4 supervisors, 54 front-line ambulance workers | To explore and describe Emergency Medical Technicians' (EMTs) experiences of critical incidents and views about potential interventions, in order to facilitate development of interventions that take into account EMS culture | Canada | Focus groups/ individual interviews | Ethnographic content analysis |
| Barriers and Facilitators to Seeking Mental Health Care Among First Responders: "Removing the Darkness" Jones et al, 2020 | 9/9 | N=32 Twenty-five (78%) of the participants were active firefighters, 15 (47%) were certified EMTs, and 11 (34.4%) were certified EMTs/ paramedics. | To explore factors that influenced FRs' perceptions of mental health problems and engagement in MH services. | United states | Ethnographic individual interviews | Content analysis |
| Exposure to human tragedy, empathy, and trauma in ambulance paramedics. Regehr et al, 2002 | 6/9 | N=18 Paramedics | This mixed-methods study attempts to better understand factors that lead to higher levels of distress among paramedics within the theoretical framework of emotional and cognitive empathy. | Canada | Semi-structured interviews | Thematic analysis |
| The meaning of traumatic events as described by nurse sin ambulance service Jonsson et al. 2003 | 5/9 | N=362 240 EMTs, 122 registered nurses | The aim of this phenomenological study is to uncover the essence of traumatic events experienced by Swedish ambulance personnel. | Sweden | Written reports | Phenomenological analysis |
| Guilt, shame and need for a container: a study of post-traumatic stress among ambulance personnel | 4/9 | N=10 Ambulance nurses and ambulance technicians | The phenomenon approached in this study could be described as 'the way ambulance staff experience and handle traumatic events'. | Sweden | Individual interviews | Descriptive phenomenology |



For peer review only - http://bmjopen.bmj.com/site/about/guidelines.xhtml

| | | | | | | |
|---|---|---|---|---|---|---|
| Jonsson et al. 2004 | | | | | | |
| Situation Critical: High Demand, Low Control, and Low Support in Paramedic Organizations Regehr et al. 2007 | 7/9 | N=17 Paramedics | This mixed-methods study involving survey design and qualitative interviews seeks further to understand the factors related to these high levels of occupational stress. | Canada | Long interviews | Constant comparative method |
| Emergency Medical Services Provider Perspectives on Pediatric Calls: A Qualitative Study Jessica et al. 2019 | 8/9 | N=17 Paramedics and EMTs | This qualitative study was conducted to increase understanding about the difficulties of responding to pediatric calls and to obtain information about how organizations can better support EMS providers in managing potentially difficult calls. | United states | Focus groups | Directed content analysis |
| An assessment of the need of police officials for trauma intervention programmes - a qualitative approach. Boshoff et al. 2015 | 5/9 | N=40 Police officials | To conduct a qualitative situational analysis by exploring the experience and specific needs with regards to trauma and trauma intervention of police officials within the North-West Province's specialist units. | South Africa | Focus groups | Thematic analysis |
| A preliminary investigation of post-traumatic stress symptoms among firefighters Haslam et al. 2003 | 4/9 | N=31 11 firefighters, 8 station officers, 4 sub officers, 4 leading firefighters, 2 fire control officers, 2 area divisional officers | This preliminary study aimed to conduct an in-depth investigation of symptoms cited by fire service personnel and assess potential risk factors for mental health and PTSD. | England | Individual interviews | Thematic analysis |
| A qualitative study about experiences and emotions of emergency medical technicians and out-of-hospital emergency nurses after performing cardiopulmonary resuscitation resulting in death Fernández-Aedo et al. 2017 | 4/9 | N=13 7EMTs, 6 ambulance nurses | To explore the experiences, emotions and coping skills among emergency medical technicians and emergency nurses after performing out-of-hospital cardiopulmonary resuscitation maneuvers resulting in death. | Spain | Semi-structured individual interviews and focus groups | Thematic analysis |
| Experiences of and actions towards worries among ambulance nurses in their | 7/9 | N=25 Ambulance nurses | The purpose of this study was to describe critical incidents in which ambulance nurses experience worry in their | Sweden | Semi-structured individual interviews | Structural analysis/Critical incident technique |



For peer review only - http://bmjopen.bmj.com/site/about/guidelines.xhtml

| | | | | | | |
|---|---|---|---|---|---|---|
| professional life: A critical incident study Svensson et al. 2008 | | | professional life and the actions they take in order to prevent and cope with it. | | | |
| Exploring the nature of resilience in paramedic practice: A psycho-social study Clompus et al. 2016 | 5/9 | N=7 Paramedics or EMTs | The aim of this study was to explore the question of how paramedics 'survive' their work within the current healthcare climate. | England | Biographical narrative interviews and semi-structured interview with all participants | Thematic analysis |
| First response emergency care – experiences described by firefighters Abelsson et al. 2019 | 5/9 | N=35 Firefighters | The purpose of this paper is to describe firefighters' experiences of first response emergency care | Sweden | Group interviews | Interpretive qualitative content analysis |
| Paramedics' experiences with death notification: a qualitative study Douglas et al. 2013 | 5/9 | N=28 Paramedics | To explore paramedics' experiences and coping strategies with death notification in the field. | Canada | Focus groups | Inductive thematic analysis |
| Peer-support: a coping strategy for nurses working at the Emergency Ambulance Service Carvello et al. 2019 | 4/9 | N=14 Ambulance nurses | The aim of the study is to explore the experiences, the opinions and feelings of emergency medical service nursing staff in relation to the use of the peer supporting model. | Italy | Semi-structured interviews | Not made explicit |
| Experiences among firefighters and police officers of responding to out-of hospital cardiac arrest in a dual dispatch programme in Sweden: an interview study Hasselqvist-Ax et al. 2019 | 8/9 | N=22 10 police officers, 12 firefighters | The aim of this interview study was to explore firefighters' and police officers' experiences of saving lives in OHCA in a dual dispatch programme. | Sweden | Individual interviews | Critical incident technique |
| Working in prehospital emergency contexts: Stress, coping and support from the perspective of ambulance personnel Oliveira et al. 2019 | 8/9 | N=14 Ambulance personnel | The purpose of this paper is to explore, from this group perspective, sources of stress, coping strategies and support measures | Portugal | Semi-structured interviews | Thematic analysis |
| Exploring paramedic communication and emotional expression in the workplace after responding to emergency calls | 7/9 | N=8 Paramedics | The study aim was to learn about peer communication and emotional expression between paramedics in the workplace, after they respond to calls. | Canada | Semi-structured individual interviews | Constructivist grounded theory |

For peer review only

| | | | | | | |
|---|---|---|---|---|---|---|
| Drewitz-Chesney et al. 2019 | | | | | | |
| Mental Health in the UK Police Force: a Qualitative Investigation into the Stigma with Mental Illness Edwards et al. 2020 | 6/9 | N=5 Police officers | The study aims to explore institutional negativity and stigma in the police force towards mental ill health | United Kingdom | Semi-structured individual interviews | Thematic analysis |
| Living in Critical Times: The Impact of Critical Incidents on Frontline Ambulance Personnel: A Qualitative Perspective Gallagher et al. 2007 | 2/9 | N=27 21 EMTs, 6 EMCs | The principal aim of this second stage of the study was to ascertain, using qualitative methods, the impact of CIs on frontline staff by allowing them to tell their own stories. | Ireland | Individual interviews | Thematic analysis |
| *Police officers, mental (ill-) health and spoiled identity* Bullock et al. 2018 | 5/9 | N=59 52 police officers, 2 police staff, 4 PCSOs, 1 special constable | The processes through which some police officers with mental ill-health experience stigmatization. | England and Wales | Phone interviews | Thematic analysis |
| 'You see a baby die and you're not fine:' a case study of stress and coping strategies in volunteer emergency medical technicians Folwell et al. 2018 | 6/9 | N=25 EMTs | This study explores the lived experience of ESP involved in unsuccessful pediatric resuscitation efforts and how this experience affects them professionally and personally. | United States | Individual interviews | Constant comparative analysis |


For peer review only - http://bmjopen.bmj.com/site/about/guidelines.xhtml

Table 3: Summary of Themes

| Domain | Higher level theme | Descriptive themes |
| --- | --- | --- |
| Factors contributing to mental health | Organisational sources of support | -Time out/Downtime<br>-Supervisor<br>-Official peer support network |
| | Informal support | -Colleagues and family<br>-Regular partner<br>-Reassurance and validation |
| Factors influencing help-seeking behaviour | Nature of intervention delivery | -Mandatory vs non-mandatory<br>-Shared experiences with intervention provider |
| | Stigma as a barrier to help-seeking | -Macho culture<br>-Stigma and shame<br>-Career concerns<br>-Confidentiality concerns |
| | Mental health literacy | -Emotional awareness<br>-Education and stigma |

## 1. Factors contributing to mental health



For peer review only - http://bmjopen.bmj.com/site/about/guidelines.xhtml

The following themes describe factors which participants attribute to having a direct influence on protecting their mental health or facilitating recovery from mental ill health after traumatic incident exposure.

**Organisational sources of support**

Three themes were identified as being directly linked with systems put in place by the organisations employing ESWs and represent opportunities for mental health support following trauma exposure over which ESOs have control.

*Time-out/Downtime period*

A 'time-out' or 'downtime' period refers to a period of time following a stressful call in which ESWs are temporarily placed off duty, the availability of which was inconsistent across the studies. ESWs working in organisations in which they were offered downtime by their supervisors following certain calls reported that these breaks, ranging from 30 minutes to two hours,[53,56] were essential in order to allow them to 'decompress' in preparation for the remainder of the shift.[53,54] ESWs found particular comfort in casual conversation with their



For peer review only - http://bmjopen.bmj.com/site/about/guidelines.xhtml

colleagues during this time, which did not necessarily relate to the previous call.[53] During such discussion, humour could be employed by the group as a method of distraction and off-loading.[59] While the majority of ESWs preferred to be in the company of colleagues during this time,[55,59,60,63] some individuals chose to make use of organisation-provided exercise equipment in order to de-stress.[65]

When time-out opportunities were unavailable, ESWs describe rushing into the next call without having psychologically processed the previous call.[51] In such circumstances, paramedics reported difficulty giving their full attention to the next call, limiting their ability to provide life-saving care.[64] Contrary to the above findings, one ambulance worker preferred to be dispatched to another call immediately following a stressful call, due to the distraction this provided.[53]

*Supervisor*

ESWs have supervisors or line managers, whose roles include acting as a point of contact after a traumatic incident. In an ambulance setting, paramedics were appreciative of the 'genuine concern' shown by their managers or supervisors following a traumatic call.[52]



For peer review only - http://bmjopen.bmj.com/site/about/guidelines.xhtml

Concern was commonly expressed by asking paramedics how they were feeling, and providing them with an opportunity to talk.[52,53] Not all ESWs want to be approached by their supervisor immediately following the call, as illustrated by the following quote from a paramedic:

> *'I don't want you to come up and get in my face and say, are you okay? Just leave me alone. Okay. Ask me in a couple of days, am I okay with the call, sort of thing.'*[53]

Occasionally supervisors were responsible for taking an ambulance crew off duty after a call.[53] Even if downtime opportunities were available upon individual request, paramedics described not making use of the opportunity unless suggested or requested by the supervisor.[60] Conversely, a supervisor's influence may also dissuade paramedics from requesting temporary downtime, especially for newer paramedics who were fearful of perceived repercussions linked to an inability to cope.[60]



For peer review only - http://bmjopen.bmj.com/site/about/guidelines.xhtml

Paramedics described unsupportive supervisor responses, which could include failing to recognise the traumatising effect of an incident, applying disciplinary pressure after complicated calls,[51] or showing a lack of concern for paramedics' mental wellbeing.[53,62]

*Official peer support network*

The majority of ambulance nurses taking part in one study were in favour of peer supporters within their organisation whom the nurses described as being able to understand their distress due to their common experiences.[61] Despite the apparent popularity of the service in this organisation, peer support networks in other organisations were rarely used[59,64] with defusing occurring 'naturally within the halls' instead.[59] Participants in two studies expressed a hesitancy to make use of peer support opportunities for fear of being judged by colleagues as 'weak'.[61,64] Other concerns centred around the competence of peer supporters, their ability to maintain confidentliality,[61,64] and fear of overwhelming colleagues delivering the support.[61]

**Informal support**

In contrast to organisational factors, three themes emerged related to how informal social factors influence the protection of ESW mental health and recovery from mental ill health.



For peer review only - http://bmjopen.bmj.com/site/about/guidelines.xhtml

*Colleagues and family*

ESWs reported that they found it useful to talk with someone in an informal manner.[53,54,55,57,59,60,61,63,64,66,68,70,71] One firefighter described a need to 'vent a backpack', which fills up after each call.[71] Along a similar vein, one study reported suppression to be harmful in the long term since this coping style can obfuscate mental health conditions.[68] The main providers of such informal support were family members and work colleagues, but there were mixed findings in terms of preferences for support.

Many ESWs reported turning to their family members as a primary source of emotional support following difficult calls,[66,53,72,50,56,70,59] who were capable of 'selfless listening' without judgement,[66] and with whom ESWs felt more comfortable sharing emotional vulnerability compared to colleagues.[53,66] However, a number of ESWs reported avoiding talking to their family members about stressful calls out of a wish to protect them from the trauma they experienced,[56,58,59,66,70-72] although this did not apply to family members with a first responder/healthcare background, who were judged to be able to understand ESWs' traumatic experiences.[57-59] For similar reasons, ESWs were willing to talk to certain colleagues about traumatic calls. The informal sharing of vulnerability was


For peer review only - http://bmjopen.bmj.com/site/about/guidelines.xhtml

reserved for colleagues with whom ESWs shared a bond of trust[57,58,63,66] and for those more likely to empathise and understand the emotional impact of the event.[50,53,55-58] Sharing experiences with trusted colleagues provided an opportunity for reflection and to hear different interpretations of the event. The risks of disclosure included reliving distressing events, and potential for feelings to be invalidated when partners felt differently about the event.[58]

*Regular partner*

ESWs described how having a regular work partner helped their ability to process traumatic events encountered on the job.[51,53,63,72]A trusting relationship between partners facilitated comfortable sharing of vulnerability following traumatic calls. [53,63] Having shared the experience, partners could emotionally support colleagues by allowing them to talk about the call and provide reassurance.[51,72] Having a regular partner could however be a negative influence in the case of an unsympathetic relationship, such as partners who respond insensitively to disclosures of vulnerability.[63] Due to the potential stigma arising from the disclosure of vulnerability within earshot of colleagues, the process of 'defusing' between

For peer review only

partners, following a call, commonly takes place within the private space of the ambulance, when returning to base and while awaiting the next call. [63]

*Reassurance and validation*

Reassurance, provided by colleagues indicating that they would have acted in the same way[52,54,58] or by receiving praise for their actions from their supervisor,[53] were valued by ESWs following traumatic incidents, especially those involving fatalities.[53] Reviewing the technical aspects of calls with other ESWs provided reassurance that the final outcome was unavoidable.[71] In cases of suicide, learning about the preceding circumstances could provide closure for some ESWs.[53] Following fatal accidents, paramedics also described needing to visit family members in hospital or to attend funerals.[52]

## 2. Factors influencing help-seeking behaviour

The following themes reflect the barriers and facilitators to help-seeking following occupational traumatic exposure.



For peer review only - http://bmjopen.bmj.com/site/about/guidelines.xhtml

**Nature of intervention delivery**

Two themes emerged related to how the method in which a formal intervention is offered can influence attitudes towards engagement.

*Mandatory vs non-mandatory*

The decision to employ optional or mandatory organisational mental health support for ESWs following traumatic calls was raised in several of the studies and often depended on the timing of delivery following the incident. Some participants resisted mandatory organisational mental health support following traumatic calls; police officers expressed a need to '*feel in control of the decision to talk'* due to the stigma surrounding any disclosure of vulnerability.[66] Mandatory interventions for ESWs could lead to a rejection of the intervention,[53,60] as illustrated by the following paramedic quote:

> *'My emotions are none of your business and if I wanted to share my emotions with you, I'm going to share [them] with someone I trust…*[60]



For peer review only - http://bmjopen.bmj.com/site/about/guidelines.xhtml

Others however believed mandatory interventions would reduce stigma associated with their use,[60,66] and prevent delays to help-seeking due to the stigma associated with disclosing vulnerability.[53] Police officers who were initially reluctant to participate felt 'calmer' and expressed gratitude after attending a mandatory counselling service.[66] EMS staff in one study suggested limiting mandatory support to certain types of incident, such as those involving children.[53]

*Shared experiences with intervention provider*

Therapists with a background in the emergency services or the military, or trained peers, were preferred by ESWs[72] because of the belief that they are more likely to understand their problems and experiences.[60] This finding was observed across the emergency services:

> *'Many [participants] also approved of a provider that "knew the job," either working with multiple FRs in the past, or even as a family member.'*[72]



For peer review only - http://bmjopen.bmj.com/site/about/guidelines.xhtml

## Stigma as a help-seeking barrier

*'Macho' culture*

Stigma associated with the disclosure of emotional vulnerability related to traumatic calls, and mental health issues.[50,53,54,59,60,62,63,64,66,68,70] was identified across the emergency services. A 'macho' attitude and culture acted as a key barrier to disclosure where there was an expectation to *'deal with it'*.[63,68] Disclosure of vulnerability in such a culture was perceived as a weakness and responders were viewed as unable to cope with the demands of the job as a first responder.[53,59,60,63,66,69,70] Revealing one's feelings was perceived to be emasculating and prevented ESWs from talking about their feelings and seeking support[60,66,70] as demonstrated by this quote from a police officer:

> 'I *think there's a real element of machismo and masculinity in the police force and it's a bit, sort of a faux pas to admit that things have really affected you ... If I'd have come out and said ''ah you know, that really affected me badly, let's go and sit down and have a cup of tea and talk about it'' I think you're straying into pink and fluffy territory there ... saying 'that made me feel sad' is a bit too far'*.[66]



For peer review only - http://bmjopen.bmj.com/site/about/guidelines.xhtml

Stigmatising attitudes held by senior organisational members were influential as they prevented ESWs from contacting their supervisors to seek support.[66,69] Discussing stress in this culture was described as 'taboo'.[64] ESWs often avoided talking to their colleagues about their emotions following traumatic calls.[60,62] Police officers described how 'tough' colleagues working in such a culture have died by suicide.[68] Some organisations described a contrasting culture in which openness about emotional vulnerability was regarded as a strength.[71]

Two studies identified a connection between elements of the 'macho' culture described, and the gender of ESWs. Swedish ambulance nurses reported that organisations where there was a higher proportion of women fostered a culture of openness with respect to sharing vulnerability.[50] Of a small sample of seven paramedics, Clompus et al. noted that female participants were more likely than male participants to have made use of formal mental health support mechanisms.[59] The authors attribute the gender bias in accessing support in this study as being related to *'masculinised paramedic culture'*.[59]

*Stigma and shame*



For peer review only - http://bmjopen.bmj.com/site/about/guidelines.xhtml

This review identified that the fear of being shamed by being labelled as '*malingerers*'[68] or '*the lazy and the lame*'[69] resulted in presenteeism when officers remained on active duty although mentally unwell. Mental health stigma appears related to the belief that affected individuals are less competent in their responsibilities as an ESW as well as being unreliable.[69] Police officers described a common belief that *'you're on your own'* working a shift with a colleague who has been open about the emotional impact of traumatic calls.[66] ESWs who have been open about their mental health diagnoses describe being labelled as '*mad*'[67] or a '*crazy guy*'.[72] Such attitudes may result in shame and the avoidance of help-seeking.

*Career concerns*

Four studies report that police officers delay help-seeking for mental illness due to concerns about the perceived impact of disclosure on their careers [66-69] Officers believed that being labelled with a mental health condition would obstruct career progression.[67-69] Officers feared being removed from 'public-facing operational roles', and/or feared a reduction in pay which related to being removed from frontline duities.[68] Fear of involuntary dismissal due to disclosure and help-seeking was also reported by study participants from the fire service.[70]



For peer review only - http://bmjopen.bmj.com/site/about/guidelines.xhtml

*Confidentiality concerns*

Concerns regarding confidentiality were a barrier for ESWs to formal and informal help-seeking behaviour. Formal support services were viewed with suspicion by police officers [67] while other officers felt they might be monitored or labelled as 'weak' if they sought a referral to wellbeing services.[66] Concerns about confidentiality were also raised by firefighters, who therefore requested preference for an anonymous counselling service outside of the brigade.[70] In the emergency medical services, EMTs expressed concerns about loss of confidentiality through the organisation-provided peer support network;[64] similar concerns were expressed by EMTs towards critical incident stress debriefing (CISD).[65] There are also perceived risks of confidentiality breaches in informal settings as described by one paramedic:

> *I think that the stigma is you have to be very careful who you tell that it bothered you or you might get judged as weak or you might get fired.*[72]


For peer review only - http://bmjopen.bmj.com/site/about/guidelines.xhtml

Confidentiality concerns could also indirectly influence help-seeking or the provision of support. For example, ambulance supervisors wishing to put their crew on a time-out after a traumatic call could be dissuaded by knowing this information could be disclosed to dispatchers.[53]

**Mental health literacy**

*Emotional awareness*

Participants and authors of the included studies recognised a need for more training and education about mental health related issues for ESWs and family members and supervisors, who may be in a better position to detect behavioural changes associated with mental ill health and could facilitate help-seeking.[52,53,64,72] Such education should focus on increasing ESWs' ability to detect emotional or behavioural changes within themselves, which could facilitate help-seeking behaviour.[52,64,64,67,72] ESWs expressed a desire to be informed about the types of emotions which could be triggered by work-related traumatic incidents, [52,64] which may reduce shame associated with help-seeking.[72] Studies revealed that an inability to recognise milder mental health symptoms acted as a barrier to help-seeking with participants



For peer review only - http://bmjopen.bmj.com/site/about/guidelines.xhtml

writing them off as being 'grumpy',[68] and not recognising and admitting to emotional distress.[52] Additionally, participants were sometimes unaware of the support services available to them[63,67,72] and were unaware of the benefits of seeking help.[72]

*Education and Stigma*

While stigma may indirectly change through improving general mental health awareness, authors also emphasised the value of education to reduce organisational stigma.[53] ESWs recommend such education to be delivered regularly in 'brief and efficient' classes of small groups by a peer from outside of the organisation.[72] Having an awareness of work-related mental health problems among colleagues appeared to be an important facilitator of help-seeking.[69,72] Experienced ESWs were regarded as being influential in reducing perceived stigma by giving permission to other responders to 'open up'.[71] Police officers in one organisation therefore advocated for mental health 'champions'; colleagues, preferably leaders, who could model vulnerability by openly disclosing their mental illness and work-related distress .[69]

For peer review only

# Discussion

This review synthesised 24 primary studies investigating ESW attitudes to help-seeking and protection of mental health and recovery from mental ill health following trauma exposure. The synthesis generated 14 themes relating to 'factors contributing to mental health' and 'factors influencing mental health help-seeking behaviour'. Despite being grouped separately for increased clarity, these overarching constructs are interconnected. Both constructs explore the influence that senior organisational members have on the mental health of ESWs within their organisation. The influence could be positive, such as delivering educational sessions about mental ill health to reduce stigma, or negative, such as the finding that ESWs may not be given downtime after a critical incident. Another interconnecting area relates to the themes of 'macho culture' and 'stigma and shame', as these stigmatising attitudes contribute towards a hesitancy to use official peer support networks.

<u>Help-seeking: culture/stigma</u>

The findings of this review support quantitative findings that fears of a breach in confidentiality related to accessing help for mental ill health and associated career



For peer review only - http://bmjopen.bmj.com/site/about/guidelines.xhtml

repercussions pose significant stigma-related barriers to help-seeking for ESWs.[37] This is important as any delays in help-seeking can compound or exacerbate mental ill health.[74,75] Concerns about the impact of mental health disclosure on career progression, professional identity and competence have also been found in military personnel.[37,43,66,76,77]

Expressing emotional vulnerability, being labelled with a mental health condition and seeking help was equated with a perception of weakness, which contributed to a 'macho' culture. Similar social norms have been described in the military, [23,77] which is male dominated with high rates of mental health stigma.[37] There are, however, many other similarities between the two settings which are likely to be influential, such as 'norms and values that place a premium on self-reliance in the face of obstacles'.[37] Furthermore, these findings may reflect evidence that women are more likely to seek help about their mental health,[78] and corresponds with the literature suggesting that men in male-dominated professions are less likely than women to seek mental health support,[79] and experience higher rates of suicide.[80] Whilst women experience a reduced risk of suicide in male-dominated professions, they experience a slightly increased risk in female-dominated professions,[80] highlighting the complexity of using occupational gender ratios to predict mental health help-seeking behaviours and mental health outcomes. Occupations dominated by hyper-masculine

For peer review only

stereotypes may also disadvantage men who do not identify with these values, therefore discouraging the very demographic who would challenge them.[81] Recruiting a more diverse, emotionally literate and aware workforce, with more women may challenge macho work cultures which prevent ESWs from disclosing their vulnerability.[81]

This review's finding of anticipated stigma acting as a barrier to help-seeking, is supported by the findings from a systematic review which found that *'stigma can potentially lead to delayed presentation in mental health care'* for ESWs,[37] and have an adverse influence on help-seeking in civilian populations. [76] These findings are also consistent with a qualitative review of a military setting.[43]

In terms of attitudes to mental ill, our findings are consistent with Goffman who argued that stigma is defined in and enacted through socially constructed norms. The norms of what is stigmatising and what isn't are socially constructed within a large variety of contexts and have the potential to shift.[82] An effective way of challenging stigma associated with mental illness and changing negative perceptions is through a strategy of increasing contact [23,83-85] in which there is equal status, the opportunity for individuals to get to know

For peer review only

each other, information which challenges negative stereotypes, active co-operation and pursuit of a mutual goal. Such approaches may be helpful to tackle the stigma and culture which prevent ESWs from seeking help. Elements of these factors can already be recognised in organisational strategies such as Mental Health Champions and Schwartz rounds.

**Help-seeking: Education**

This review identifies a demand for improving the mental health literacy of ESWs and describes the type of education ESWs believe would be appropriate. Mental health education has been shown to be effective at changing attitudes towards mental health disorders when aimed at large populations, smaller at-risk groups or at the individual level.[86,87] Anti-stigma education was introduced in UK emergency services as part of the 'Blue Light Programme',[7] and demonstrated that achieving anti-stigma change at the employee level requires sustained education efforts over a number of years.[7]

The literature suggests that certain types of events, such as paediatric fatalities, events involving multiple casualties or suicides, have a higher traumatising potential than others.[50,70,88] The evidence suggests however that the process of developing a post-trauma mental health disorder among ESWs is highly individual, relating to the personal history,

For peer review only


For peer review only - http://bmjopen.bmj.com/site/about/guidelines.xhtml

situation, and perspective of the exposed individual,[65] and ESWs can experience trauma in different ways depending on how they contextualise the victim. [50] Questionnaires taking into account individual factors or risk factors, such as the ESW's propensity to dwell,[89] are therefore a useful resource for predicting post-trauma psychopathology for exposed individuals.[90]

**Protecting mental health: Social support and downtime**

Consistent with the wider literature, review participants identified the importance of social support following exposure to traumatic incidents.[25,43,91,92] Downtime was positively valued by ESWs, yet is not commonly granted to ESWs in practice.[92,93] The types of mental health outcome affected by post-incident downtime is disputed. Carlier et al. identified 'insufficient time allowed by the employer to resolve the trauma' was correlated with higher PTSD scores in police officers three months post-trauma, but not at 12 months.[94] Having insufficient recovery time following traumatic calls has been correlated with higher emotional exhaustion[95] and psychological distress.[96] These results are in contrast with the findings of a cross-sectional quantitative survey of the psychological consequences of downtime in 217



For peer review only - http://bmjopen.bmj.com/site/about/guidelines.xhtml

ambulance workers, which revealed increasing periods of downtime, up to and including one day, to be significantly associated with lower depression scores, but not with symptoms of post-traumatic stress, burnout and stress-related physical symptoms.[97]

**Application of review findings to organisational interventions, policy and research**

This review was initiated in collaboration with key ambulance management stakeholders who expressed a need for research to guide decisions about wellbeing interventions for frontline staff, criteria which were expanded by this review to also include fire and police organisations. Our findings have implications for three organisational interventions which are in use or available for use within ESOs.

1) *Trauma risk management (TRiM)*

Consistent with qualitative findings of navy personnel regarding the implementation of TRiM, [98] this review identified a perception among ESWs that peer-support programs are relevant and suitable for their needs, as well as concerns regarding

For peer review only

confidentiality and competence of practitioners of peer support programs. Peer supporters are not however regarded as possessing the same professional competence/credibility of a professional mental health professional. Of note, while review participants expressed concerns about being judged for being perceived as 'weak' by peers in a peer-support system, such a concern was not detected among naval officers towards TRiM,[98] despite widespread stigma towards help-seeking and mental health issues in the military.[43]

2) *Mental Health Champions*

We identified that awareness of colleagues' mental health challenges could be an important facilitator for help-seeking. The 'Blue light champions' role[99] therefore appears to be a potentially effective method for improving attitudes, although quantitative evaluation linking these roles with ESW mental health outcomes and culture change is lacking. Staff satisfaction surveys for these roles reveal a lack of support from management and insufficient time available to dedicate to the role.[100]

3) *Schwartz Rounds*



For peer review only - http://bmjopen.bmj.com/site/about/guidelines.xhtml

In terms of challenging organisational culture which prohibits help-seeking due to stigma, Schwartz Rounds offer staff the opportunity to disclose their vulnerability whilst fostering a connectedness to other staff. However, Schwartz Rounds have not been evaluated and it is unclear whether they reduce stigma, facilitate help-seeking behaviour, or alter mental health outcomes. Further evaluation of Schwartz Rounds is required.

**A note about Covid**

This is the first review to qualitatively synthesise the barriers and facilitators to help-seeking in the emergency services, and frontline staff's experience of what may protect and what may hinder mental health after traumatic incident exposure. The search was conducted in March 2020, before the emergence of qualitative literature relating to the Covid pandemic, offering a summary of evidence preceding this significant historical benchmark. Qualitative literature relating to ESW mental health in the context of Covid-19 necessitates analysis in its own right, due to the distinct psychological stressors brought on by the pandemic, such as fear of infecting family members.[101] Healthcare workers experience different mental health pressures



For peer review only - http://bmjopen.bmj.com/site/about/guidelines.xhtml

For peer review only

during pandemic-working,[102] and should therefore be studied in a separate context to police and fire workers.

## Strengths and limitations

A core strength of this review is the analysis, which includes multiple perspectives on the topic, comprising of a primary care and ambulance clinician, a medical student, and two applied health researchers. A user advisory group including ESWs was consulted regarding the design and purpose of the research and advised on questions to include, broadening its applicability. Limitations include the focus on only English language studies conducted in western countries with the exception of one study.[67] As such, findings may not generalise to other countries. Study quality varied significantly with research design commonly being inadequately explained. It should be recognised that while the review has applicability to ambulance fire and police services, the majority of studies meeting the eligibility criteria were drawn from an ambulance service background. Only one reviewer performed the initial screen of studies. However, a second reviewer was recruited to independently assess potentially relevant studies against eligibility criteria. Although the included studies are

For peer review only

drawn from pre-Covid literature, the findings are omnirelevant to the issues of traumatisation and mental health help-seeking in ESWs.

## Implications for further research to help inform policy

Following traumatic calls, ESWs will likely benefit from a recovery period during which they may wish to access informal support from their colleagues. ESOs should be aware of the therapeutic effects of informal support in the post-incident setting and facilitate its availability since ESWs are unlikely to request downtime themselves. It may also be useful for supervisors to consider that the manner in which they approach ESWs for welfare concerns influences their help-seeking behaviours. It may be important to consider that ESWs report trusting professional relationships, such as regular work-partners, as being psychologically protective against the psychological consequences of occupational trauma experiences. ESWs may benefit from education enhancing their ability to recognise pathological emotional and behavioural changes associated with traumatic incident exposure for themselves and colleagues, and to



For peer review only - http://bmjopen.bmj.com/site/about/guidelines.xhtml

able to suggest accessing formal organisational resources and interventions as appropriate. ESOs may wish to consider that mental health champion-type roles, providing staff in such roles are adequately supported, are regarded by ESWs as valuable tools for challenging mental health stigma. Our findings may provide insights into how engagement between ESWs and official peer support networks could be improved, by focussing on promoting anti-stigma and by targeting barriers to help-seeking.

## Conclusion

Our review identified barriers and facilitators to help-seeking which may assist emergency medical organisations in improving staff engagement with organisational wellbeing interventions. This is in keeping with the organisation's responsibility to dismantle barriers to help-seeking and reduce stigma related to mental ill health and vulnerability. Our review identified the importance of organisational cultures in which it was safe to be vulnerable and need for supportive and compassionate leadership.



For peer review only - http://bmjopen.bmj.com/site/about/guidelines.xhtml

For peer review only

## Footnotes

**Acknowledgements:** This work was initially written as a dissertation. We would like to thank Justin Waring for being a valued source of support throughout the dissertation. Special thanks to Anna Parry for her vital contributions in the early stages of the project, and involving key ambulance stakeholders during the process of developing the project aims. We would also like to thank Marcus Auth for his assistance provided in the form of advice regarding structure of the paper, and formulating the abstract.



For peer review only - http://bmjopen.bmj.com/site/about/guidelines.xhtml

**Contributors:** NA is the main author involved in all stages of the design, analysis and drafting and writing of the paper. RR was involved in the conception of the project's scope, design, and interpretation of results, providing important supervision and mentorship throughout. RR, MB and JW assisted with drafting the article and revising it critically for important intellectual content, providing methodological advice, quality appraisal by independently appraising lower CASP scoring studies, and final approval of the version to be published. All authors have approved the final version of the paper ahead of publication.

**Funding:** This research received no specific grant from any funding agency in the public, commercial or not-for-profit sectors.

**Competing interests:** None declared.

**Provenance and peer review:** Not commissioned; externally peer reviewed.

**Ethics approval:** Research ethics approval not applicable.

**Data sharing statement:** No additional data are available.

**Figure legend:** Figure 1. Preferred Reporting Items for Systematic Reviews and Meta-Analyses (PRISMA) flow diagram.

# References

1. Jones S. Describing the Mental Health Profile of First Responders: A Systematic Review. J Am Psychiatr Nurses Assoc 2017 May;23(3):200-214.
2. Lima Ed P, Assuncao AA. Prevalence and factors associated with Posttraumatic Stress Disorder (PTSD) in emergency workers: a systematic literature review. Rev bras epidemiol 2011 Jun;14(2):217-230.
3. Berger W, Coutinho E, Figueira I, Marques-Portella C, Luz M, Neylan T, et al. Rescuers at risk: a systematic review and meta-regression analysis of the worldwide current prevalence and correlates of PTSD in rescue workers. Soc Psychiatry Psychiatr Epidemiol 1206;47(6):1001-1011
4. Stanley IH, Hom MA, Joiner TE. A systematic review of suicidal thoughts and behaviors among police officers, firefighters, EMTs, and paramedics. Clin Psychol Rev 1603;44:25-44.
5. Petrie K, Milligan-Saville J, Gayed A, Deady M, Phelps A, Dell L, et al. Prevalence of PTSD and common mental disorders amongst ambulance personnel: a systematic review and meta-analysis. Soc Psychiatry Psychiatr Epidemiol 2018 Sep;53(9):897-909.
6. Substance Abuse and Mental Health Services Administration. First Responders: Behavioral Health Concerns, Emergency Response, and Trauma. [internet]. 2018 May. [cited 2019 Oct 14]. Available from: https://www.samhsa.gov/sites/default/files/dtac/supplementalresearchbulletin-firstresponders-may2018.pdf
7. Mind. Wellbeing and mental health support in the emergency services. [internet]. 2019. [cited 2019 November 5]. Available from: https://www.mind.org.uk/media-a/4572/20046_mind-blue-light-programme-legacy-report-v12_online.pdf
8. Mitchell, J.T., & Everly, G.S. (1995). Critical Incident Stress debriefings: An operations manual for the prevention of traumatic stress among emergency service



For peer review only - http://bmjopen.bmj.com/site/about/guidelines.xhtml

and disaster workers (2nd ed. revised). Ellicott City, MD: Chevron Publishing Company.

9. Jakupcak, M., Cook, J., Imel, Z., Fontana, A., Rosenheck, R., & McFall, M. Posttraumatic stress disorder as a risk factor for suicidal ideation in Iraq and Afghanistan War veterans. Journal Of Traumatic Stress 2009 July;22(4), 303-306. doi: 10.1002/jts.20423
10. Tarrier N, Gregg L. Suicide risk in civilian PTSD patients. Social Psychiatry and Psychiatric Epidemiology. 2004;39(8).
11. Maia DB, Marmar CR, Metzler T, Nóbrega A, Berger W, Mendlowicz MV, et al. Post-traumatic stress symptoms in an elite unit of Brazilian police officers: Prevalence and impact on psychosocial functioning and on physical and mental health. J Affect Disord 2007;97(1):241-245.
12. Case R, Alabakis J, Bowles K-A, Smith K: High-risk occupations—Suicide: an Evidence Check rapid review brokered by the Sax Institute (www.saxinstitute.org.au) for the NSW Ministry of Health, 2020.
13. Mind. The case for support. [internet]. 2017. [cited 2019 November 6]. Available from: https://www.mind.org.uk/media-a/4510/blp-blueprint_case-for-support.pdf
14. Antony J, Brar R, Khan P, Ghassemi M, Nincic V, Sharpe J et al. Interventions for the prevention and management of occupational stress injury in first responders: a rapid overview of reviews. Systematic Reviews. 2020;9(1).
15. Smith A, Roberts K. Interventions for post-traumatic stress disorder and psychological distress in emergency ambulance personnel: a review of the literature. Emergency Medicine Journal 2003 /01/01;20(1):75-78.
16. Carlier IVE, Lamberts RD, Van Uchelen AJ, Gersons BPR. Disaster-related post-traumatic stress in police officers: a field study of the impact of debriefing. Stress Med 1998 9807 9807;14(3):143-148.
17. Sattler DN, Boyd B, Kirsch J. Trauma-exposed Firefighters: Relationships among Posttraumatic Growth, Posttraumatic Stress, Resource Availability, Coping and Critical Incident Stress Debriefing Experience. Stress Health 2014;30(5):356-365.
18. Tuckey MR. Group critical incident stress debriefing with emergency services personnel: a randomized controlled trial. Anxiety, Stress & Coping 2014;27(1):38-55.

For peer review only

19. Regel S. Post-trauma support in the workplace: the current status and practice of critical incident stress management (CISM) and psychological debriefing (PD) within organizations in the UK. Occupational Medicine 2007 709 0;57(6):411-416.
20. Rose S, Bisson J, Churchill R, Wessely S. Psychological debriefing for preventing post traumatic stress disorder (PTSD). Cochrane Database of Systematic Reviews. 2002;.
21. Whybrow D, Jones N, Greenberg N. Promoting organizational well-being: a comprehensive review of Trauma Risk Management. Occup Med (Lond) 2015 /06/01;65(4):331-336.
22. Hunt E, Jones N, Hastings V, Greenberg N. TRiM: an organizational response to traumatic events in Cumbria Constabulary. Occup Med (Lond) 2013 /12/01;63(8):549-555.
23. Gould M, Greenberg N, Hetherton J. Stigma and the military: Evaluation of a PTSD psychoeducational program. Journal of Traumatic Stress 2007;20(4):505-515.
24. Jones N, Burdett H, Green K, Greenberg N. Trauma Risk Management (TRiM): Promoting Help Seeking for Mental Health Problems Among Combat-Exposed U.K. Military Personnel. *Psychiatry*. 2017;80(3):236-251. doi:10.1080/00332747.2017.1286894
25. Shakespeare-Finch J, Rees A, Armstrong D. Social Support, Self-efficacy, Trauma and Well-Being in Emergency Medical Dispatchers. Soc Indic Res 1509;123(2):549-565.
26. Greenberg N. A cluster randomized controlled trial to determine the efficacy of Trauma Risk Management (TRiM) in a military population. J Trauma Stress ;23(4):430-437.
27. The Point of Care Foundation. List of Schwartz Rounds sites. [internet] [cited 2020 May 19]. Available from: https://www.pointofcarefoundation.org.uk/our-work/schwartz-rounds/list-of-sites/
28. Taylor C, Xyrichis A, Leamy M, Reynolds E, Maben J. Can Schwartz Center Rounds support healthcare staff with emotional challenges at work, and how do they compare with other interventions aimed at providing similar support? A systematic review and scoping reviews. BMJ Open. 2018;8(10):e024254.
29. Maben J, Taylor C, Dawson J, Leamy M, Mccarthy I, Reynolds E, et al. A realist informed mixed-methods evaluation of Schwartz Center Rounds® in England. Health Services and Delivery Research 1101;6(37).



For peer review only - http://bmjopen.bmj.com/site/about/guidelines.xhtml

30. HMICFRS. Fire and rescue- Staffordshire. [internet] 2019 [cited 2020 April 5]. Available from: https://www.justiceinspectorates.gov.uk/hmicfrs/frs-assessment/frs-2018/staffordshire/people/
31. HMICFRS. Fire and rescue- Buckinghamshire. [internet] 2019 [cited 2020 April 5]. Available from: https://www.justiceinspectorates.gov.uk/hmicfrs/frs-assessment/frs-2018/buckinghamshire/
32. HMICFRS. Fire and rescue- Derbyshire. [internet] 2019 [cited 2020 April 5]. Available from: https://www.justiceinspectorates.gov.uk/hmicfrs/frs-assessment/frs-2018/derbyshire/
33. HMICFRS. Police- Dfyed-Powes. [internet] 2019 [cited 2020 April 5]. Available from: https://www.justiceinspectorates.gov.uk/hmicfrs/peel-assessments/peel-2018/dyfed-powys/legitimacy/detailed-findings/#question-3
34. HMICFRS. Police- Cumbria. [internet] 2019 [cited 2020 April 5]. Available from: https://www.justiceinspectorates.gov.uk/hmicfrs/peel-assessments/peel-2018/cumbria/legitimacy/detailed-findings/#question-1
35. Her Majesty's Inspectorate of Constabulary and Fire & Rescue Services (HMICFRS). Fire and rescue- Gloucestershire. [internet] 2019 [cited 2020 April 5]. Available from: https://www.justiceinspectorates.gov.uk/hmicfrs/frs-assessment/frs-2018/gloucestershire/people/
36. UNISON. Linc Peer Support. [internet] 2016 [cited 2020 April 20]. Available from: https://lasunison.com/linc-peer-support
37. Haugen PT, McCrillis AM, Smid GE, Nijdam MJ. Mental health stigma and barriers to mental health care for first responders: A systematic review and meta-analysis. Journal of Psychiatric Research. 2017(10):125-126.
38. Flemming K, Noyes J. Qualitative Evidence Synthesis: Where Are We at?. International Journal of Qualitative Methods. 2021;20:160940692199327.
39. Booth A, Noyes J, Flemming K, Moore G, Tunçalp Ö, Shakibazadeh E. Formulating questions to explore complex interventions within qualitative evidence synthesis. BMJ Global Health. 2019;4(Suppl 1):e001107.
40. Litz BT. Distinct Trauma Types in Military Service Members Seeking Treatment for Posttraumatic Stress Disorder. J Trauma Stress 2018;31(2):286-296.
41. Ridner SH. Psychological distress: concept analysis. J Adv Nurs. 2004 ;45(5):536-546.



For peer review only - http://bmjopen.bmj.com/site/about/guidelines.xhtml

42. Noyes J & Lewin S. Chapter 5: Extracting qualitative evidence. In: Noyes J, Booth A, Hannes K, Harden A, Harris J, Lewin S, Lockwood C (editors), Supplementary Guidance for Inclusion of Qualitative Research in Cochrane Systematic Reviews of Interventions. Version 1 (updated August 2011). Cochrane Collaboration Qualitative Methods Group, 2011. Available from URL http://cqrmg.cochrane.org/supplemental-handbook-guidance .

43. Coleman SJ, Stevelink SAM, Hatch SL, Denny JA, Greenberg N. Stigma-related barriers and facilitators to help seeking for mental health issues in the armed forces: a systematic review and thematic synthesis of qualitative literature. 1708;47(11):1880-1892.

44. CASP. Qualitative checklist. [internet] 2018 [cited 2020 March 5]. Available from: https://casp-uk.net/wp-content/uploads/2018/03/CASP-Qualitative-Checklist-2018_fillable_form.pdf

45. Dixon-Woods M, Cavers D, Agarwal S, Annandale E, Arthur A, Harvey J, et al. Conducting a critical interpretive review of the literature on access to healthcare by vulnerable groups. 2006.

46. Sommerfeld A, Wagner S, Harder H, Schmidt G. Behavioral Health and Firefighters: An Intervention and Interviews with Canadian Firefighters. Journal of Loss and Trauma. 2017;22(4):307-324.

47. Thomas J. Methods for the thematic synthesis of qualitative research in systematic reviews. BMC Medical Research Methodology ;8:45-46.

48. Thomas J, Sutcliffe K, Harden A, Oakley A, Oliver S, Rees R, Brunton G, Kavanagh J (2003) Children and Healthy Eating: A systematic review of barriers and facilitators. London: EPPI-Centre, Social Science Research Unit, Institute of Education, University of London.

49. Barnett-Page E, Thomas J. Methods for the synthesis of qualitative research: a critical review. BMC Medical Research Methodology. 2009;9(1).

50. Regehr C, Goldberg G, Hughes J. Exposure to human tragedy, empathy, and trauma in ambulance paramedics. American Journal of Orthopsychiatry. 2002;72(4):505-513.

51. Regehr C, Millar D. Situation critical: High demand, low control, and low support in paramedic organizations. Traumatology. 2007;13(1):49-58.

52. Halpern J, Gurevich M, Schwartz B, Brazeau P. What makes an incident critical for ambulance workers? Emotional outcomes and implications for intervention. Work & Stress. 2009;23(2):173-189.



For peer review only - http://bmjopen.bmj.com/site/about/guidelines.xhtml

53. Halpern J, Gurevich M, Schwartz B, Brazeau P. Interventions for critical incident stress in emergency medical services: a qualitative study. Stress and Health. 2009;25(2):139-149.
54. Jonsson A, Segesten K. The meaning of traumatic events as described by nurses in ambulance service. Accident and Emergency Nursing. 2003;11(3):141-152.
55. Jonsson A, Segesten K. Guilt, shame and need for a container: a study of post-traumatic stress among ambulance personnel. Accident and Emergency Nursing. 2004;12(4):215-223.
56. Jeruzal J, Boland L, Frazer M, Kamrud J, Myers R, Lick C et al. Emergency Medical Services Provider Perspectives on Pediatric Calls: A Qualitative Study. Prehospital Emergency Care. 2019;23(4):501-509.
57. Fernández-Aedo I, Pérez-Urdiales I, Unanue-Arza S, García-Azpiazu Z, Ballesteros-Peña S. A qualitative study about experiences and emotions of emergency medical technicians and out-of-hospital emergency nurses after performing cardiopulmonary resuscitation resulting in death. Enfermería Intensiva (English ed). 2017;28(2):57-63.
58. Svensson A, Fridlund B. Experiences of and actions towards worries among ambulance nurses in their professional life: A critical incident study. International Emergency Nursing. 2008;16(1):35-42.
59. Clompus S, Albarran J. Exploring the nature of resilience in paramedic practice: A psycho-social study. International Emergency Nursing. 2016;28:1-7.
60. Douglas L, Cheskes S, Feldman M, Ratnapalan S. Paramedics' experiences with death notification: a qualitative study. Journal of Paramedic Practice. 2012;4(9):533-539.
61. Carvello M, Zanotti F, Rubbi I, Bacchetti S, Artioli G, Bonacaro A. Peer-support: a coping strategy for nurses working at the Emergency Ambulance Service. *Acta Biomed.* 2019;90(11-S):29-37
62. Oliveira A, Neto F, Teixeira F, Maia Â. Working in prehospital emergency contexts. International Journal of Workplace Health Management. 2019;12(6):469-482.
63. Drewitz-Chesney C. Exploring paramedic communication and emotional expression in the workplace after responding to emergency calls. Australasian Journal of Paramedicine. 2019;16.
64. Gallagher S, McGilloway S. Living in critical times: The impact of critical incidents on frontline ambulance personnel--a qualitative perspective. *Int J Emerg Ment Health.* 2007;9(3):215-223.


For peer review only - http://bmjopen.bmj.com/site/about/guidelines.xhtml

65. Folwell A, Kauer T. ‘You see a baby die and you’re not fine:’ a case study of stress and coping strategies in volunteer emergency medical technicians. Journal of Applied Communication Research. 2018;46(6):723-743.
66. Evans R, Pistrang N, Billings J. Police officers’ experiences of supportive and unsupportive social interactions following traumatic incidents. European Journal of Psychotraumatology. 2013;4(1):19696.
67. Strydom H, Botha K, Boshoff P. AN ASSESSMENT OF THE NEED OF POLICE OFFICIALS FOR TRAUMA INTERVENTION PROGRAMMES – A QUALITATIVE APPROACH. Social Work/Maatskaplike Werk. 2015;51(2).
68. Edwards A, Kotera Y. Mental Health in the UK Police Force: a Qualitative Investigation into the Stigma with Mental Illness. International Journal of Mental Health and Addiction. 2020;.
69. Bullock K, Garland J. Police officers, mental (ill-)health and spoiled identity. Criminology & Criminal Justice. 2017;18(2):173-189.
70. Haslam C, Mallon K. A preliminary investigation of post-traumatic stress symptoms among firefighters. Work & Stress. 2003;17(3):277-285.
71. Abelsson A. First response emergency care – experiences described by firefighters. International Journal of Emergency Services. 2019;8(3):247-258.
72. Jones S, Agud K, McSweeney J. Barriers and Facilitators to Seeking Mental Health Care Among First Responders: “Removing the Darkness”. Journal of the American Psychiatric Nurses Association. 2019;26(1):43-54.
73. Hasselqvist-Ax I, Nordberg P, Svensson L, Hollenberg J, Joelsson-Alm E. Experiences among firefighters and police officers of responding to out-of-hospital cardiac arrest in a dual dispatch programme in Sweden: an interview study. BMJ Open. 2019;9(11):e030895.
74. Fridgen G, Aston J, Gschwandtner U, Pflueger M, Zimmermann R, Studerus E, et al. Help-seeking and pathways to care in the early stages of psychosis. Soc Psychiatry Psychiatr Epidemiol 1307;48(7):1033-1043.
75. Gronholm PC. Mental health-related stigma and pathways to care for people at risk of psychotic disorders or experiencing first-episode psychosis: a systematic review. Psychol Med ;47(11):1867-1880.
76. Clement S, Schauman O, Graham T, Maggioni F, Evans-Lacko S, Bezborodovs N, et al. What is the impact of mental health-related stigma on help-seeking? A systematic review of quantitative and qualitative studies. Psychol Med 2015 101 0;45(1):11-27.


For peer review only - http://bmjopen.bmj.com/site/about/guidelines.xhtml

77. Sharp M, Fear NT, Rona RJ, Wessely S, Greenberg N, Jones N, et al. Stigma as a Barrier to Seeking Health Care Among Military Personnel With Mental Health Problems. Epidemiol Rev 2015 /01/01;37(1):144-162.

78. Australian Bureau of Statistics. National Survey of Mental Health and Wellbeing: Summary of Results, Australia, 2007. ABS cat. no. 4326.0. Canberra: ABS; 2008.

79. Milner A, Scovelle A, King T. Treatment-seeking differences for mental health problems in male- and non-male-dominated occupations: evidence from the HILDA cohort. Epidemiology and Psychiatric Sciences. 2018;28(6):630-637.

80. Milner A, King T. Men's work, women's work and suicide: a retrospective mortality study in Australia. Australian and New Zealand Journal of Public Health. 2018;43(1):27-32.

81. Peters K, Ryan M, Haslam S. Marines, medics, and machismo: Lack of fit with masculine occupational stereotypes discourages men's participation. British Journal of Psychology. 2014;106(4):635-655.

82. E. Goffman. Stigma: Notes on the management of spoiled identity. Prentice-Hall, Englewood Cliffs, NJ (1963)

83. Dickstein BD, Vogt DS, Handa S, Litz BT. Targeting Self-Stigma in Returning Military Personnel and Veterans: A Review of Intervention Strategies. Military Psychology 2010 April 2,;22(2):224-236.

84. Norms, Committee on the Science of Changing Behavioral Health Social, Board on Behavioral C, Education, Division of Behavioral and Social Sciences and, National Academies of Sciences, Engineering. Approaches to Reducing Stigma. : National Academies Press (US); 2016.

85. Like Minds. THE POWER OF CONTACT- Project to Counter Stigma and Discrimination Associated with Mental Illness. [internet] 2005 [cited 2020 April 6]. Available from: https://www.likeminds.org.nz/assets/National-Plans/1power-of-contact.pdf

86. Jorm AF, Barney LJ, Christensen H, Highet NJ, Kelly CM, Kitchener BA. Research on Mental Health Literacy: What we know and what we Still Need to know. Aust N Z J Psychiatry 2006 January 1,;40(1):3-5.

87. Jorm AF. Mental Health Literacy: Empowering the Community to Take Action for Better Mental Health. Am Psychol ;67(3):231-244.



For peer review only - http://bmjopen.bmj.com/site/about/guidelines.xhtml

88. Jacobsson A, Backteman-Erlanson S, Brulin C, Hörnsten Å. Experiences of critical incidents among female and male firefighters. International Emergency Nursing. 2015;23(2):100-104.
89. Wild J, Smith KV, Thompson E, Béar F, Lommen MJJ, Ehlers A. A prospective study of pre-trauma risk factors for post-traumatic stress disorder and depression. Psychological Medicine. Cambridge University Press; 2016;46(12):2571–82.
90. Halpern J, Maunder R, Schwartz B, Gurevich M. The critical incident inventory: characteristics of incidents which affect emergency medical technicians and paramedics. BMC Emergency Medicine 101 0;12:10.
91. Brewin CR, Andrews B, Valentine JD. Meta-Analysis of Risk Factors for Posttraumatic Stress Disorder in Trauma-Exposed Adults. J Consult Clin Psychol 10 00;68(5):748-766.
92. Gouweloos-Trines J, Tyler MP, Giummarra MJ, Kassam-Adams N, Landolt MA, Kleber RJ, et al. Perceived support at work after critical incidents and its relation to psychological distress: a survey among prehospital providers. Emerg Med J 1221;34(12):816
93. Alexander D, Klein S. Ambulance personnel and critical incidents: impact of accident and emergency work on mental health and emotional well-being. British Journal of Psychiatry 101 0(178):76-81.
94. CARLIER I, LAMBERTS R, GERSONS B. Risk Factors for Posttraumatic Stress Symptomatology in Police Officers: A Prospective Analysis. The Journal of Nervous &amp Mental Disease. 1997;185(8):498-506.
95. Alexander D, Klein S. Ambulance personnel and critical incidents: impact of accident and emergency work on mental health and emotional well-being. British Journal of Psychiatry 101 0(178):76-81.
96. Mitchell AM. Critical incident stress debriefing: implications for best practice. Disaster Management & Response 2003;1(2):46-52.
97. Halpern J. Downtime after critical incidents in emergency medical technicians/paramedics. BioMed Research International ;2014:483140-483141.
98. Greenberg N, Langston V, Iversen AC, Wessely S. The acceptability of 'Trauma Risk Management' within the UK Armed Forces. Occup Med (Lond) 2011 /05/01;61(3):184-189.
99. Mind. Mind Blue light champions. [internet] 2019 [cited 2020 Feb 19]. Available from: https://www.mind.org.uk/media-a/4843/blp-role-description.pdf



For peer review only - http://bmjopen.bmj.com/site/about/guidelines.xhtml

100. Mind. Blue Light Programme Strand 1 (Part 2). [internet] 2016 [cited 2020 May 5]. Available from: https://www.mind.org.uk/media-a/4864/strand-1-part-2.pdf
101. Cai H, Tu B, Ma J, Chen L, Fu L, Jiang Y et al. Psychological impacts and coping strategies of front-line medical staff during COVID-19 outbreak in Hunan, China. Medical Science Monitor. 2020;26.
102. Vagni M, Maiorano T, Giostra V, Pajardi D. Coping With COVID-19: Emergency Stress, Secondary Trauma and Self-Efficacy in Healthcare and Emergency Workers in Italy. Frontiers in Psychology. 2020;11.

For peer review only

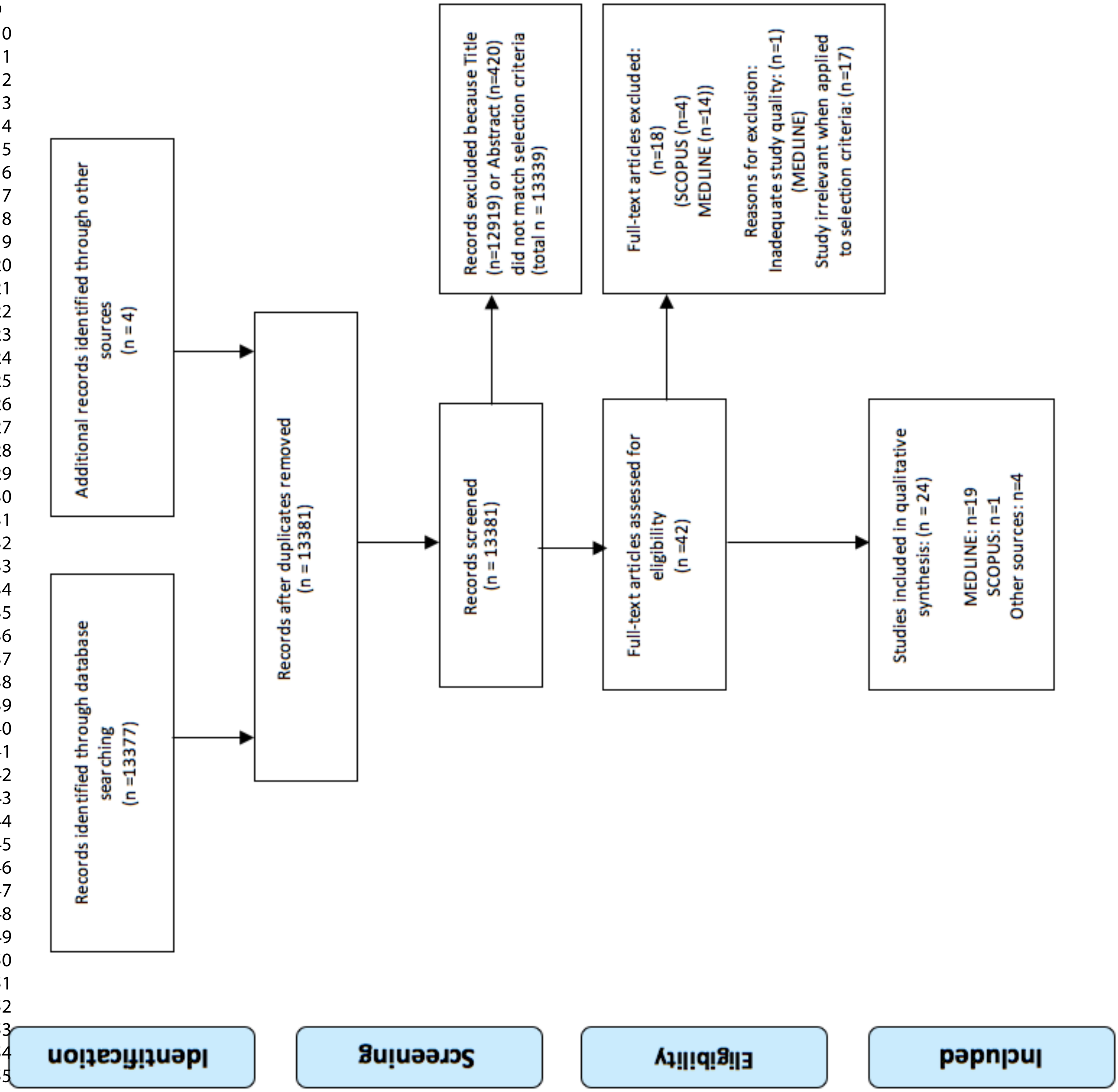
Identification
Screening
Eligibility
Included
Records identified through database searching (n =13377)
Additional records identified through other sources (n =4)
Records after duplicates removed (n = 13381)
Records screened (n = 13381)
Records excluded because Title (n=12919) or Abstract (n=420) did not match selection criteria (total n = 13339)
Full-text articles assessed for eligibility (n =42)
Full-text articles excluded: (n=18) (SCOPUS (n=4) MEDLINE (n=14))
Reasons for exclusion: Inadequate study quality: (n=1) (MEDLINE) Study irrelevant when applied to selection criteria: (n=17)
Studies included in qualitative synthesis: (n = 24)
MEDLINE: n=19 SCOPUS: n=1 Other sources: n=4

**Online supplementary file 1: ENTREQ checklist (Enhancing transparency in reporting the synthesis of qualitative research**

| No | Item | Guide and description |
|---|---|---|
| 1 | Aims | -to identify factors and contexts that may contribute to mental health and recovery from psychological difficulties for emergency service workers (ESWs) exposed to occupational trauma<br>-to identify barriers and facilitators to help-seeking behaviour among trauma-exposed ESWs. |
| 2 | Synthesis Methodology | Thematic synthesis |
| 3 | Approach to searching | Pre-planned comprehensive search strategies, combining database searching with manual search methods. |
| 4 | Inclusion criteria | Articles were eligible for inclusion provided they met the following criteria:<br>(1) Study participants were frontline ESWs (studies with mixed populations of eligible participants were included); (2) The study focus was work-related psychological distress*; (3) Data collection included primary qualitative interviews, focus groups or observational methods (this included mixed methods studies with qualitative components); (4) Analysis focussed around participant attitudes towards: a) behaviour aimed at improving or protecting mental health after experiencing a traumatic event OR b) factors which ESWs find helpful or unhelpful for their mental health while experiencing work-related psychological distress; (5) Published in English and peer-reviewed.<br>No limits were applied to publication date or study location.<br>Exclusion criteria:<br>(1) Due to the unique nature of the traumatic events witnesses in this cohort,[40] studies investigating a military cohort were excluded; (2) Volunteer ESWs were not included. |
| 5 | Data sources | OVID MEDLINE, EMBASE, PsycINFO, SCOPUS. To identify articles missed in the electronic database search, the following |

For peer review only

| | | |
|---|---|---|
| | | methods were also employed:<br>-Using 'related article' feature (when available).<br>-Searching the titles of included studies in google scholar for citation tracking purposes.<br>-Manual searching of the references of relevant studies (reference chaining).<br>Grey literature was searched during background research for context but not to locate eligible studies. |
| 6 | Electronic search strategy | The two combinations of search criteria (see Appendix A) were entered into each database. Figure 1 provides an overview of the database search. |
| 7 | Study screening methods | One reviewer (NA) screened the 13381 article titles and/or abstracts identified by the search. The 42 full text articles identified in this process were then independently assessed for their eligibility criteria by two reviewers (NA and RR) (Figure 1). |
| 8 | Study characteristics | Study characteristics are presented in Table 1. |
| 9 | Study selection results | PRISMA guidance was used to construct a flow diagram displaying the database searching process (Figure 1). Of the 13381 records identified once duplicates were removed, the full text of 42 articles were screened, and 24 studies were included in this qualitative synthesis. |
| 10 | Rationale for appraisal | All potentially relevant studies were quality appraised by one reviewer (NA), using the Critical Appraisal Skills Program (CASP) guidelines. Studies meeting less than 5 criteria were then subject to further scrutiny, in the form of five quality appraisal prompts developed by Dixon-Woods et al. (see 'quality appraisal' section). This second stage of appraisal was carried out independently by by RR, MB or JW, and decisions relating to the inclusion of these studies in the review were made following thorough communication between reviewers and referring to pre-determined quality prompts. |
| 11 | Appraisal terms | Critical Appraisal Skills Program (CASP) guidelines and quality appraisal prompts (see 'quality appraisal' section) were used to quality appraise all included studies. |

For peer review only

| | | |
|---|---|---|
| 12 | Appraisal process | Quality assessment was carried out primarily by one reviewer (NA), with independent verification from RR, MB or JW for ambiguous studies. |
| 13 | Appraisal results | CASP scores of included studies are provided in Table 1. Full study quality assessments are available for review if required. |
| 14 | Data extraction | A data extraction template (Appendix B) was created for this review by one reviewer (NA). Sections for first, second and third order constructs are included in the extraction template, which was filled manually by one reviewer (NA). |
| 15 | Software | RefWorks reference management software. |
| 16 | Number of reviewers | Two reviewers were involved in the coding and analysis (NA, RR). |
| 17 | Coding | Two reviewers carried out line-by-line coding, grouping of codes and generation of descriptive themes (NA and RR). |
| 18 | Study comparison | During primary readings of the studies, overarching concepts relating to the research aims were noted. The generation of new codes altered pre-existing codes. |
| 19 | Derivation of themes | Initial coding was carried out in correspondence between two reviewers (NA and RR), and was grounded in the extracted data. Thematic synthesis of initial codes was an inductive approach, and involved roundtable discussion between all four authors. |
| 20 | Quotations | Appendix C provides example literature quotations from included studies which were used to construct themes. |
| 21 | Synthesis output | This qualitative review generated 14 descriptive themes. These are grouped into either 'Factors contributing to the protection of mental health' or 'factors influencing mental health help-seeking behaviour'. Table 2 presents a summary of themes. These descriptive themes were applied to the context of UK emergency service organisations to produce implications for practice and research. |

Tong A. Enhancing transparency in reporting the synthesis of qualitative research: ENTREQ. BMC Medical Research Methodology ;12(1):181-182.

For peer review only

# Manuscript online supplementary files

Online Supplementary File 1: ENTREQ checklist (submitted separately)

**Online Supplementary File 2: CASP appraisal table**

For peer review only

**Online Supplementary File 2: CASP appraisal table**

| Study title (Authors, year) | Aims & Methods | Research design | Sampling | Data collection | Reflexivity | Ethical issues | Data analysis | Discussion of findings | Value |
|---|---|---|---|---|---|---|---|---|---|
| *What makes an incident critical for ambulance workers? Emotional outcomes and implications for intervention.* Halpern et al. 2009[42] | Research aim clearly stated, introduction uses relevant literature to successfully explain demand for the research. Qualitative methods are appropriate for investigating complicated emotions, which could not be explored in equal depth via quantitative methods. | Interviews and focus groups seem appropriate methods to address the research aims, but the authors don't specify the reasons for their choice to use both methods. | 4 supervisors, 54 front-line ambulance workers. Researchers state that participants were recruited from a specific cohort at a mandatory training conference, but no detail is given about what information was provided to the participants to entice them to enter the study. The authors considered representation of all job levels and both genders | The setting of the data collection away from the workplace was stated but no justification was given for the choice of study setting or methods used. Detail was given about the length of interviews and focus groups, and the main topics of questions asked during interviews and focus groups were stated. The researchers stated they had reached saturation by the end of the study. Data obtained was in the form of | Researchers do not acknowledge how their characteristics may have impacted on the results. The researchers acknowledge that self-selection or reporting bias may have contributed to the discrepancy in findings between men and women. | Ethical approval was obtained from a research ethics board, and participants signed a consent form. No further information was given regarding any ethical safeguards in place. Lack of description of how confidentiality was maintained. | The authors described how ethnographic content analysis was used to generate thematic codes, with a constant comparative method throughout. Three authors were involved in the coding process, although only two of these authors listened to the audiotapes. Initial and final codes are described, and contradictory data are taken into account. Sufficient data are | Findings are explicitly stated and are relevant to the researchers' aims. The only measure of credibility described was the use of three analysts during the coding process. Findings are discussed with reference to wider literature, and implications for interventions for critical incident stress are made. | The authors identify promising post-incident interventions which they believe should be further researched. Wider applicability of the study findings appear to not extend beyond the ambulance cohort. The authors provide implications for interventions for critical incident stress. |

| | | | | | | | | | |
|---|---|---|---|---|---|---|---|---|---|
| | | | during recruitment. | audio recordings, written transcripts and written notes of non-verbal communication information as observed by the researchers. | | | presented to support the findings. The authors describe how textual data was not analysed for focus groups. | | |
| *Police officers' experiences of supportive and unsupportive social interactions following traumatic incidents.* Evans et al. 2013[56] | Research aims made clear in objectives section. Overall aim 'was to understand the types of support processes that might promote resilience'. Methods are appropriate as experiences, and other subjective outcomes are best measured qualitatively. | Semi-structured interview design appears reasonable to explore the proposed aims, but choice of qualitative study design not directly justified. | 19 Police officers. A snowballing approach was used to recruit officers, which allowed detection of information-rich participants. By limiting participants to those who have served over 2 years, the chance of traumatic incident exposure was increased. Limiting participants to those without diagnosed PTSD was appropriate as the study measured resilience-promoting investigations. Non-participation was not discussed. | An interview schedule was used for the semi-structured interviews, although only the broad focus of the schedule was provided by the authors. Interviews were audio-recorded and transcribed verbatim. No information was given regarding interview setting, and data saturation was not discussed. No justification for choice of methods. | Researchers acknowledge how their role as researchers could have affected the data, giving social desirability bias as an example. | A National Health Service ethics committee granted ethical approval, and participants provided informed consent. No information is provided regarding efforts to maintain confidentiality. | The process of thematic analysis was described in detail. All themes generated can be linked with relevant first order data. Contradictory data is taken into account and present within the themes. Three authors were involved in the analytic process to minimise bias. One author had the main role in the analysis, while two others cross-referenced certain parts of their work in order to increase credibility. All 3 researchers reached a consensus on unclear issues before the final themes were decided upon. | Themes are clearly summarised, with relevant quotations to support each theme. Findings are discussed in relation to the research aims. Three researchers were involved in the analytic process. | The researchers provide detailed recommendations for further quantitative research. Furthermore, the researchers recommend similar qualitative research in a population that has a history of PTSD, so that comparisons can be made. |
| *Interventions for critical incident stress in emergency medical services: a qualitative study* | Clear statement of study aims, with overall study purpose ' to facilitate development of interventions that | Explanations were provided for how the focus groups and semi-structured interviews were conducted. E.g | 4 supervisors, 54 front-line ambulance workers. Participants were sampled to ensure all job levels and | The setting of the data collection away from the workplace was stated but not explained. Detail was given about | Researchers do not critically examine their role, potential bias and influence of research | Ethical approval was obtained from a research ethics board, and participants signed a consent form. No further | The authors described how ethnographic content analysis was used to generate thematic codes, using a | The findings are clearly presented, using relevant participant quotations to support the authors | The research conclusions appear relevant to all EMS organisations which operate in the format of |

| | | | | | | | | | |
|---|---|---|---|---|---|---|---|---|---|
| Halpern et al. 2008[43] | take into account EMS culture.' Examining experiences adequately requires qualitative methodology. | flexible interview structure ' to permit the elaboration of more in-depth or emotionally significant data' and focus group size of 4-8 members 'to maximize interactive data'. No justification given for choice of interviews and focus groups. | genders were represented. Researchers state that participants were recruited from a specific cohort at a mandatory training conference, but no detail is given about what information was provided to the participants to entice them to enter the study. The sampling process was iterative- preliminary analysis informed subsequent sampling decisions. No further participants were recruited once saturation was reached. | the length of interviews and focus groups, and the main topics of questions asked during interviews and focus groups were stated. The researchers stated they had reached saturation by the end of the study. Data obtained was in the form of audio recordings, written transcripts and written notes of non-verbal communication information as observed by the researchers. | question formulation or data collection. | information was given regarding any ethical safeguards in place. | constant comparative method to categorise themes. Initial and final codes are described. The authors explain why textual data was not analysed for focus groups. Member validation was carried out 6 months post initial data collection. Contradictory data are presented and discussed, and sufficient data are presented to support the findings. | interpretations. 3 analysts were used during the data analysis process. Member validation was carried out 6 months after data was gathered. Contradictory viewpoints are taken into account and discussed. | using supervisors. The authors provide suggestions for interventions which are of relevance to EMS organisations. The authors recommend that the interventions of:<br>1. Supervisor support<br>2. Timeout period post-incident<br><br>should be further researched. |
| *Barriers and Facilitators to Seeking Mental Health Care Among First Responders: "Removing the Darkness"* Jones et al, 2020[62] | Aims are clearly stated and the study's importance justified. The authors aims include exploring organisational culture and perceptions of individuals, for which qualitative research is appropriate. | The decision to use individual ethnographic interviews the qualitative method of choice is well explained. The authors also explain why they chose a 'community-based approach'. | 32 firefighters and/or EMTs/paramedics.<br><br>This was a convenience sample. The principal investigator facilitated recruitment by developing relationships with community partners who each recruited participants from | Ethnographic ndividual interviews were conducted with participants by the principal investigator. An interview guide was used and attached, which was developed by the PI based on the study aim and input from a qualitative methods expert, | The principal investigator acknowledged how being married to a firefighter/paramedic promoted buy in from the community during the recruitment stage. | Ethical board approval was gained. 'Prior to starting the interview, the PI reviewed the study information sheet and completed the consent process with each participant. ' Resources for further emotional support were provided to participants in case of emotional | Content analysis and constant comparison methods were used during the analytic process. Themes which emerged during data analysis were checked with participants in later interviews. Two researchers independently analysed the transcripts, and discussed the process until | Findings are presented as themes, with participant quotations included for each theme to support the author's interpretations. Credibility was enhanced by the use of more than one analyst, and member validation. Findings are discussed in relation to the | The authors make implications for practice, identifying 'need for improving education and awareness regarding duty-related MH problems.' The authors also make implications for nursing practice, and implications for future research. |

For peer review only

| | | | | | | | | | |
|---|---|---|---|---|---|---|---|---|---|
| | | | their own organisations. They did this by distributing e-mails on behalf of the principal investigator. Reasons for non-participation: 'individuals not answering or returning calls (all were called twice), scheduling conflicts' | along with feedback from community partners to strengthen trustworthiness and validity. Interviews were audio recorded and transcribed. Field notes were also conducted by the authors. Data collection setting was decided on the basis of convenience for participants. Data saturation was reached. | | distress experienced during the study. | consensus was reached. (on 80% of top level codes). Exploration of new themes was stopped when saturation was reached. participant quotations are provided throughout to support the author's interpretations. The researchers acknowledge a bias risk due to the principal investigator conducting all the interviews. Sufficient data are presented to support the findings, and contradictory data are reported (such as positive and negative experiences of therapists). | original research question. | |
| *Exposure to human tragedy, empathy, and trauma in ambulance paramedics.* Regehr et al, 2002[40] | Aims of study are clearly stated, and their importance justified. Qualitative methods are appropriate because the authors want to explore experiences in depth. | Choice of qualitative study design not explicitly justified. | 18 paramedics. 'Purposive sampling was used to ensure that participants represented a wide range of experiences in terms of length of time with the service and types of events encountered.' Non participation | A semistructured interview guide was used to conduct interview, and the authors provided examples of guide topics. These interviews were audio-recorded and transcribed, and field notes were taken. The authors acknowledge they | Researchers do not critically examine their role, potential bias and influence of research question formulation or data collection. No mention of changing the research design as the study progressed. | No mention of ethical approval, or consent gained from participants. No attempt is made to explain how confidentiality was maintained. | Nvivo was used to aid in data analysis. Open coding was used initially to generate broad categories. Towards the later stages of the process, selective coding was used to develop a meaningful narrative of the experiences of the | Findings are presented in sections headed by themes. Author's comments are supported by first order interpretations. Triangulation with members of emergency service organization was carried out | The authors acknowledge that the study is 'not intended to reflect the views of all paramedics in all organizations; rather, it describes a particular phenomenon experienced and described by one group of paramedics and |

| | | | | | | | | | |
|---|---|---|---|---|---|---|---|---|---|
| | | | was not discussed. | required a larger than normal sample size to achieve saturation. No information is provided for the setting of data collection.<br><br>'Other sources of data included the notes recording the interviewer's impressions.' | | | workers. Interviewers also recorded field notes. Triangulation with members of emergency service organization was carried out throughout the research, and analysis of data was discussed. Two members of the research team developed the coding tree together. A third member reviewed the open and selective coding. It is not clear how the themes were derived from the data, but sufficient data are presented to support the findings. Contradictory data are included in the findings and discussed. | throughout the research, and analysis of data was discussed. Two members of the research team developed the coding tree together. A third member reviewed the open and selective coding. | points to some interesting avenues for further consideration. These include future attempts to measure cognitive and emotional empathy in paramedics and assess the impact of these strategies on posttraumatic and depressive symptoms and on social supports.' |
| *The meaning of traumatic events as described by nurse sin ambulance service* Jonsson et al. 2003[44] | Aims of study are clearly stated, and their importance justified. Qualitative methodology is appropriate due to nature of the aims, which is to explore subjective experiences in detail. | A phenomenologica l approach was used, and the reason for its us thoroughly explained. The use of self-reports over the use of interviews was not justified (the authors said that interviews and self-reports are | 240 medical technicians and 122 registered ambulance nurses. The authors asked 500 participants of another study to write down their experiences with traumatic events. No detail is given how the sample of the | The question the participants were asked to answer was: 'Write down and describe a situation which you experienced as a traumatic event''. From the data returned by the participants, the authors selected 25% of the written | Researchers do not critically examine their role, potential bias and influence of research question formulation or data collection. No mention of changing the research design as the study progressed. | Ethical approval was granted. Consent was implied through willingness to participate in the study, but no information is given regarding a signed consent form. In addition, participants were assured that they could withdraw | Van Kaam's scientific explication was used to analyse the written stories. Detail was provided about the steps of this thematic analytic process. Only 25% of the written stories were further analysed, and | The findings are in the form of themes, supported by first order data and the author's interpretations. The findings are related to the original research question in the discussion. There is no mention of any efforts to increase the | The authors recommend specific areas for further research, limited to the ambulance sector. The organisational interventions recommended by the authors are likewise limited to the ambulance sector. |

For peer review only

| | | | | | | | | | |
|---|---|---|---|---|---|---|---|---|---|
| | | the most common methods in empiric-phenomenologica l studies. | other study was recruited. Non participation is not discussed. | accounts to further analyse. These written accounts were strategically selected based on having the fullest description of a traumatic event. Details of the analytic process are provided. Saturation of data is not discussed. | | from the study at any time. The questionnaire was not labelled with the name of the participant. Confidentiality was guaranteed by eliminating names or other identifying characteristics from the essays. | were strategically selected for being more data-rich. There is no information to reveal how many researchers are involved in the analytic process. Sufficient data is presented to support the findings. There is no mention of contradictory data being taken into account. The researcher does not critically appraise their own potential bias. | credibility of the findings. | |
| *Guilt, shame and need for a container: a study of post-traumatic stress among ambulance personnel* Jonsson et al. 2004[45] | Aims of study are clearly stated, and their importance justified. Qualitative methodology is appropriate due to nature of the aims, which is to explore subjective experiences in detail. | Descriptive phenomenology was 'chosen for its potential to grasp the meaning of such phenomena through the description of lived experiences.' | 10 Ambulance nurses or ambulance technicians. Insufficient information on how participants were The only relevant information is that participants were strategically selected to obtain a 'variation of sex, age, educational background, and experience as ambulance staff.' | Setting for interviews was described but not justified. Interviews were open in structure, beginning with the question "tell about a traumatic event that you have experienced". 'do you mean?" or "How did you feel about that?". The interviews, taking about an hour and a half each, were audio-taped and transcribed word by word.' Reason for choice of interview as method of data collection not | Researchers do not critically examine their role, potential bias and influence of research question formulation or data collection. | The study was described, the extent of participation was explained, and potential risks were explored with the participants, who were assured that they had the right to withdraw from the study at any time. The study was approved by the Ethical committee at Göteborg University. To protect participant confidentiality, no quotations were provided. | The authors provide details about the 5 steps of the analytic process, with the latter steps being repeated once the author was familiar with existing philosophy. Due to the authors not using participant quotations (to protect participant confidentiality), it is not possible to determine whether the author's findings are supported by first order interpretations. Contradictory data are discussed, but | Findings are displayed under relevant sub-headings. In the discussion section, there is some debate for and against the researcher's arguments. No attempts made to demonstrate efforts to enhance credibility. | The results can be applied to emergency medical organisations, however caution should be taken due to the small sample size and lack of information about participant demographics. The authors recommend for the insights gained by this study to be ' distributed to all ambulance managers and other relevant personnel categories. ' The authors make specific |

For peer review only

| | | | | | | | | | |
|---|---|---|---|---|---|---|---|---|---|
| | | | | explained. Saturation of data not discussed. | | | without the direct participant quotations. | | recommendations for management. |
| *Situation Critical: High Demand, Low Control, and Low Support in Paramedic Organizations* Regehr et al. 2007[41] | Aims of study are clearly stated, and their importance justified. Qualitative methodology is appropriate due to nature of the aims, which is to explore subjective experiences in detail. | Long interview design was chosen 'to explore in detail the experiences of paramedics in their organizations including their roles, the demands placed on them, the control over the working environment that they experience, and the supports that they receive.' | 17 paramedics. Paramedics were purposively sampled from the sample of participants partaking in the quantitative component of the study. Purposive sampling was used to ensure that participants represented a wide range of experiences in terms of length of time with the services and types of work experiences encountered. | A long interview method was used to collect data. The interviews followed an interview guide (not provided), were audio recorded and transcribed verbatim. The interviewer made their own notes during the interview. Setting of data collection not provided, saturation of data not discussed. | Researchers do not critically examine their role, potential bias and influence of research question formulation or data collection. | No mention of ethical approval, or consent gained from participants. No attempt is made to explain how confidentiality was maintained. | Data analysis commenced with open coding that captured a broad range of perspectives, whereas axial and selective coding facilitated the structuring of a coding framework. After the initial coding framework was developed, the transcribed interviews were imported into N*VIVO, a computer generated data analysis system, and the interview data were coded by multiple coders and subjected to detailed thematic analysis. In the final stage of analysis, constant comparative method of data analysis was implemented to compare categories and themes across respondents. Furthermore, paramedic organisations were consulted when creating the | Findings are presented in themed subheadings. Authors claim to have enhanced credibility through ' prolonged engagement and persistent observation'. No mention of more than one analyst, and qualitative findings were triangulated with quantitative results. | The authors make recommendations for EMS organisations, based on a mixture of their quantitative and qualitative results. |

| | | | | | | | | | |
|---|---|---|---|---|---|---|---|---|---|
| | | | | | | | research questions, and throughout the research project to guide it with feedback. Sufficient data are presented to support the findings, and contradictory data taken into account. | | |
| *Emergency Medical Services Provider Perspectives on Pediatric Calls: A Qualitative Study* Jessica et al. 2019[46] | Aims of study are clearly stated, and their importance justified. Qualitative methodology is appropriate due to nature of the aims, which is to explore subjective experiences in detail | The use of focus groups was not explicitly justified. | 17 EMS providers. ' Paramedics and EMTs were recruited for the study through invitations circulated via weekly staff emails, flyers posted at ambulance bases, the agency's social media page, and e-mails from the EMS chaplain. Interested participants were instructed to contact a single study investigator by phone or email 'Selection criteria were applied to the participants in order to sample for the most relevant participants- participants had to be working for over a year in order to increase chance of | The process of focus group conduction was thoroughly described. A semi-structured guide was used by the researcher to conduct the focus groups- the same researchers conducted all the focus groups. At the end of the focus group, themes were fed back to participants, who were invited to comment. Focus groups were conducted at a large ambulance service. Audio recordings were transcribed. A second researcher was present during the focus group to take notes. Thematic saturation was reached. | The researchers reflect on the potential biases of the study in the limitations section. They acknowledge that by using discussion prompts, they may have steered the conversation away from topics that the participants would otherwise have talked about. Furthermore, they acknowledge that the fact that a single coder was responsible for coding 80% of the data alone may be a source of bias. | Participants gave informed consent and ethical approval was gained from an ethics committee. Resources were provided to the participants in case of experiencing psychological distress. | Transcripts were analysed using directed content analysis. Two reviewers independently coded 20% of the transcripts, and a single researcher coded the remaining data. Findings were validated through groups consensus. Quotations are provided for all sub-themes. Authors acknowledge the limitations of focus groups, and the potential for bias with one researcher coding 80% of the data. The authors also acknowledge how the discussion guide prompts may have altered the topic of discussion of participants from what they would otherwise have | Findings are presented as sub-themes, with quotations supporting each sub-theme in a separate table. Credibility of findings was enhanced by reviewing the focus group themes with participants at the end of the focus group. Furthermore, some of the data was reviewed by two researchers. | The findings are relevant to EMS organisations, specifically EMS leaders. Although Desired support mechanisms following difficult paediatric calls can be extrapolated to other types of traumatic events. |

For peer review only

| | | | | | | | | | |
|---|---|---|---|---|---|---|---|---|---|
| | | | traumatic incident exposure. Non participation was not discussed. | | | | talked about. Contradictory data are taken into account. | | |
| *An assessment of the need of police officials for trauma intervention programmes - a qualitative approach.* Boshoff et al. 2015[57] | Aims of study are clearly stated, and their importance justified. Qualitative methodology is appropriate due to nature of the aims, which is to explore subjective experiences in detail. | Focus groups were chosen as the method of data collection in order to 'allow the researcher to gain insight into participants' beliefs about and perceptions or accounts of a particular topic'. The researchers state: The focus groups allowed the researcher to interact systematically and simultaneously with several individuals. | 40 police officials. Participants were recruited via a purposive sampling method through the health and wellbeing departments, which were used as an intermediary between the researchers and participants. Police officials were purposefully chosen considering the relevance of the topic, specifically referring to their exposure to trauma, resultant symptoms of PTS and their participation in trauma intervention programmes. Non-participation was not discussed, other than reassuring participants that non-participation would not be sanctioned. | Setting for data collection was not outlined. Three focus groups were carried out, and semi-structured interviews were performed within the focus groups. Exact questions asked are provided by the researchers. The sessions were audio recorded, transcribed and analysed. Saturation of the data is not discussed. | Researchers do not critically examine their role, potential bias and influence of research question formulation or data collection. | Ethical approval was gained for this study from the North-West University's ethical committee . Informed consent was taken by the researchers. ' Participants were furthermore encouraged to withdraw from the focus groups at any time should they feel uncomfortable or experience any harm or emotional consequence as a result of participating in the study.' | The authors describe an 8 step method of thematic analysis, but do not explain how they will apply this method. It is not clear how the data is used to arrive at the themes generated. Sufficient data are presented to support the findings. Contradictory data are taken into account. | Findings are presented as sub-themes, supported by sufficient quotations. There is limited discussion for and against the researchers' arguments. No efforts are made to enhance credibility of findings. | The authors give a variety of 'preliminary indicators' for which a ' purposeful psycho-social trauma intervention programme' is indicated. The generalisability of this is limited to police forces. |
| *A preliminary investigation of post-traumatic stress symptoms among firefighters* | Aims of study are clearly stated, and their importance justified. Qualitative methodology is | One-to-one interviews were chosen but reasons for their use over focus groups were not | 11 Firefighters, 8 Station Officers, 4 Sub Officers, 4 Leading Firefighters, 2 Fire Control | ' One-to-one interviews were conducted in a private room with participants. They lasted or up to 90 | Researchers do not critically examine their role, potential bias and influence of research | No mention of informed consent, ethical approval or methods to ensure confidentiality. | 'The interviews were recorded and fully transcribed and the data were analysed by | Findings are presented as sub-headings of themes, with participant quotations | The authors provide recommendations for fire organisations. These |

For peer review only

| | | | | | | | | | |
|---|---|---|---|---|---|---|---|---|---|
| Haslam et al. 2003[60] | appropriate due to nature of the aims, which is to explore subjective experiences in detail. | explained. The interview schedule was developed in conjunction with psychologists working within the fire service, as well using relevant literature. | Officers and 2 Area Divisional Officers. No information provided on how participants were recruited. 'The sample was selected to cover the range of positions in the service and the proportion of respondents in each position broadly reflects the profile of the service.' | minutes. The interview questions were stated by the researchers. Open-ended questions would be used to explore participants' feelings towards incidents. ;The interviews were recorded and fully transcribed and the data were analysed by sorting verbatim material into emergent themes as described by Dey (1993).'' Choice of data collection methods or study setting was not justified. Saturation of data was not discussed. | question formulation or data collection. The researchers acknowledge that their small sample size may not be representative. | | sorting verbatim material into emergent themes as described by Dey (1993). A second researcher independently checked the analysis to ensure analysis reliability' There is little transparency to show how the themes were developed from the primary data. Use of a second researcher enhances credibility. Direct participant quotations are sufficiently used to support the authors' interpretations. Contradictory data are taken into account. | supporting the authors' comments. Credibility is enhanced by the use of two analysts. | recommendations are concerned with efforts to improve staff wellbeing. |
| *A qualitative study about experiences and emotions of emergency medical technicians and out-of-hospital emergency nurses after performing cardiopulmonary resuscitation resulting in death* Fernández-Aedo et al. 2017[47] | Aims of study are clearly stated, and their importance justified. Qualitative methodology is appropriate due to nature of the aims, which is to explore subjective experiences in detail. | Both interviews and focus groups were used as methods of data collection, the reason for their use was not explained. | 7 EMTs and 6 nurses. Snowball sampling was used to recruit the participants. As a prerequisite for their inclusion in this study, the health workers were required to have carried out at least 5 CPR techniques resulting in death over their entire professional career. No volunteer staff | Questions asked during the interviews were ' developed based on the reviewed literature and experts' opinions', but the authors do not provide a topic guide or examples of other prompts. ' The assignment of participants to' either individual semi-structured interviews or focus groups 'was | Researchers do not critically examine their role, potential bias and influence of research question formulation or data collection. | ' The study was approved by the Ethics Committee of the University of the Basque Country.' 'echnique was based on their availability and preferences. Participants gave their written consent to participate in the study and to be recorded in audio and/or video format.' | A detailed description of the analytical process is provided. 'To ensure the quality of the interpretation and guarantee the reliability of the information obtained, a triangulation between all researchers involved in the interviews was also carried out. Any disagreement | Themes are not provided with any participant quotations. 'To ensure the quality of the interpretation and guarantee the reliability of the information obtained, a triangulation between all researchers involved in the interviews was also carried out. Any disagreement | The authors recommend greater training for health professionals when ' notifying bad news and providing psychological support to the family members of the deceased ' No recommendations for research are made. |

For peer review only

| | | | | | | | | | |
|---|---|---|---|---|---|---|---|---|---|
| | | | was included in the study. ‘ In order to ensure heterogeneity, the study included healthcare professionals of different ages, both genders and varying years of experience, working at different institutions.’ Non participation was not discussed. | based on their availability and preferences.’ ‘A total of 3 health emergency technicians and 3 nurses were interviewed individually for a total of 11---35 min by two of the researchers involved in the study. The focus group was comprised by 4 EMTs and 3 nurses, and it was used to triangulate the information obtained during the individual interviews. The group session lasted 76 min and was carried out by two researchers, one acting as a moderator and the other as an observer.’ Data saturation was reached. | | | was resolved by consensus.' No participant quotations are provided to support the authors' interpretations. Contradictory data re not taken into account. | was resolved by consensus.' Other than the use of multiple researchers in the analytic process, no other methods to increase credibility are discussed. | |
| *Experiences of and actions towards worries among ambulance nurses in their professional life: A critical incident study* Svensson et al. 2008[48] | Aims of study are clearly stated, and their importance justified. Qualitative methodology is appropriate due to nature of the aims, which is to explore subjective experiences in detail. | There is no justification for the choice of semi-structured interviews as a method of data collection. | 25 ambulance nurses. Participants were strategically selected from three ambulance services based on ‘socio-demographic and professional characteristics such as age, | The authors explain the structure of the interviews, giving examples of the open-ended questions which they ask to participants at the start of the interview. The authors say the | ‘As both the researchers and the nurses were familiar with the environment in which the study was conducted, the risk of misunderstanding during the interviews was minimised.' | ‘The managers of the ambulance service involved gave approval for the study to be conducted in their department.’ The authors say ethical board approval is not required in Sweden under | 'The interviews were also read through several times before categorizing them into sub-categories, in order to further improve security. The incidents were analysed according to | Findings are arranged into themes and sub-themes, and participant quotations are included for each theme to support the authors' conclusions. Credibility was enhanced via the | The authors recommend further research: 'This phenomenon should be studied more in-depth in order to map how the interaction with colleagues influences the worry among |

| | | | | | | | | | |
|---|---|---|---|---|---|---|---|---|---|
| | | | education level, sex and years in the profession'. Non participation is not discussed. | interviews took place where it best suited the participants, and three interviews were conducted over the phone. Researchers explain their use of interviews: 'Interviews were chosen as the data collection method, which allowed the respondents to describe their thoughts in more detail with the help of follow-up questions.' The authors also justify the use of the CIT method. Interviews were audio recorded and transcribed verbatim. Saturation of data was not discussed. | | certain circumstances. The authors describe how informed consent was taken from participants, including assurances that data will be treated confidentially. 'The study adhered to the principles outlined in the Declaration of Helsinki' | character and content. This step was repeated several times before the end result emerged. The categorizing of incidents into sub-categories was conducted in cooperation with the second researcher, experienced both in theory and practice, which minimised the risk of subjectivity (Andersson and Nilsson, 1964). Direct quotes from the interviews strengthened the accuracy of the study.' Contradictory data were taken into account, and sufficient are presented to support the findings. Saturation of data was not discussed. | use of two researchers when categorising incidents. Authors consider contradictory data in the discussion. | ambulance nurses.' Some recommendations are also made for employers of nurses. |
| *Exploring the nature of resilience in paramedic practice: A psycho-social study* Clompus et al. 2016[49] | Aims of study are clearly stated, and their importance justified. Qualitative methodology is appropriate due to nature of the aims, which is to explore subjective | The authors used free association narrative interviewing, a technique which involves a preliminary narrative interview, followed by a semi-structured | 'An advert, with a brief study outline, was placed in a regional Paramedic bulletin which was circulated electronically to staff with an invitation to | The researchers justify their use of using Free association narrative interviewing. Saturation was achieved after the 6th interview. Details of the exact two stages | Researchers do not critically examine their role, potential bias and influence of research question formulation or data collection. | ' Ethical approval was received from a (NHS) research ethics committee and a university in the SW of England. Confidentiality and anonymity was secured through the use of | Details of the FANI analytic process were given by the researchers. 'Any discrepancies were examined and discussed until consensus was reached. Trustworthiness | Themes are presented as sub-headings, and supported by the direct participant quotations. Member validation was carried out to improve credibility. | The authors relate the findings of the research to the theories of exiting organisational interventions, eg TRiM. The authors conclude that for front-line paramedics, ' applying |

For peer review only

| | | | | | | | | | |
|---|---|---|---|---|---|---|---|---|---|
| | experiences in detail. | interview. 'This enabled a deeper analysis of the affective and often unconscious aspects of paramedics' lives. ' | participate.' Participants had to fulfill the following criteria: -Grade of paramedic, technician or emergency care practitioner -Willing to volunteer their time<br><br>Three out of the 10 individuals became unavailable, but the authors do not explain why. | of the interviewing process were provided by the authors. interviews were audio recorded and transcribed, and carried out at a place of the participants' choosing. | | pseudonyms for participants, and all data were kept in a password protected personal computer with access limited to SC. Participants were made aware that they could withdraw at any time and that the anonymised data would be disseminated in various ways. Due to the potential distress that participation could inadvertently provoke, information on how to access counselling services was made available to all participants.' | and data credibility were established by several means including participants feeding into the study's aims, keeping contemporaneous notes, and sending interview transcripts and a summary of findings to each participant for verification'. The authors are not clear how many researchers are involved in the analytic process. For each theme, sufficient participant quotes are provided to support the authors' interpretations. Contradictory data were taken into account and discussed. | | interventions and reviewing support mechanisms would seem to be a pressing imperative.' |
| *First response emergency care – experiences described by firefighters* Abelsson et al. 2019[61] | Aims of study are clearly stated, and their importance justified. Qualitative methodology is appropriate due to nature of the aims, which is to explore subjective experiences in detail. | The authors justify their choice of group interviews, and why limits are set on group interviews: 'The group sizes of eight and nine participants were considered acceptable to moderate and managed by the researcher. | Authors don't explain how participants were recruited into the study. | 35 Firefighters. Setting for group interviews was not given. Participation numbers in the group interviews were provided and discussed. Justification for methods chosen was provided. A rough structure to the interviews was provided by | Researchers do not critically examine their role, potential bias and influence of research question formulation or data collection. | No approval has been sought from an ethics committee, which is in accordance with Swedish law. Consent was implied through participation in the study, following the delivery of verbal 'clear information' by the researchers. | The authors describe their method of text-driven, interpretive qualitative content analysis. This involved repeated readings of the transcribed interviews, and then identification of 'meaning units', then codes were derived, and | Findings are presented as four themes, with consistent use of primary quotations to support the researchers' arguments. The findings are discussed in relation to the original research question. | The researchers contrast their findings with other research in the field, such as in the case of spouses being used as sources of support by firefighters. They also recommend areas of future research, eg involving other strands of |

For peer review only

| | | | | | | | | | |
|---|---|---|---|---|---|---|---|---|---|
| | | Interaction in the pre-existing group is key to a successful group interview. In this study, all firefighters worked at the same fire station, which promoted interactions optimal for the research purpose' | | giving questions which were asked to participants. There is no mention of saturation of the data. The authors reflected in the 'limitations' section that group interviews could lead to participants being uncomfortable. | | Methods taken to protect confidentiality are described. | formed into sub-categories and categories. Sufficient data are present to support the findings. Contradictory data are discussed. It is not clear how many researchers were involved in the analytic process. There is no explicit mention of efforts to improve credibility. Authors acknowledge that 'participants in group interviews may experience a pressure within the group, resulting in similar opinions'. | | emergency service workers. |
| *Paramedics' experiences with death notification: a qualitative study* Douglas et al. 2013[50] | Aims of study are clearly stated, and their importance justified. Qualitative methodology is appropriate due to nature of the aims, which is to explore subjective experiences in detail. | The authors used focus groups as their method of data collection, but did not justify their use over other qualitative methods. | 28 paramedics. Participants were self-selected. Recruitment messages were delivered via 'departmental e-mail, flyers, and by word-of-mouth'. Authors don't explain why the participants selected were the most appropriate for the study, and non-participation is not discussed. | The authors provide the locations of the focus groups, and give examples of questions asked during the sessions. There is no explicit justification for why focus groups were chosen over other qualitative methods. Methods were modified during the study as follows: 'The question guide was adapted as | The researchers acknowledged that their choice of focus group location, as well as the presence of the supervisor in the focus group may have affected the answers given by participants. The researchers do not critically examine their own role. | Ethical board approval was gained, and written consent obtained. No information is provided on efforts to maintain participant confidentiality. | The authors describe an inductive approach to data analysis. Two researchers were involved in the analysis process. Themes were discussed with a further two authors, until consensus was reached. Credibility was enhanced via the use of member checking the participant responses with | The findings are presented as themes, supported by participant quotations and authors' interpretations. Credibility was enhanced by distributing results from the study to participants for the purpose of generating feedback. | The findings are discussed in relation to existing literature, and the authors make recommendations for practice, specifically regarding paramedic training. |

| | | | | | | | | | |
|---|---|---|---|---|---|---|---|---|---|
| | | | | required by the author and the facilitator after each session to clarify some questions based on the paramedics' responses. ' Focus groups were audiotaped and transcribed. Saturation was discussed: the authors kept recruiting participants until saturation was reached. | | | participants at the end of focus groups. Contradictory data are taken into account, and sufficient data are presented to support the findings. | | |
| *Peer-support: a coping strategy for nurses working at the Emergency Ambulance Service* Carvello et al. 2019[51] | Aims of study are clearly stated, and their importance justified. Qualitative methodology is appropriate due to nature of the aims, which is to explore subjective experiences in detail. | The authors used semi-structured interviews. The authors explain their choice of questions result from pre-existing literature, but do not justify the use of interviews over other qualitative methods eg focus groups | 'Participants were recruited on a voluntary basis from an emergency medical service in the north of Italy.' The sampling was 'non-probabilistic', according to the following criteria: nurses working at the emergency ambulance service "118" in a hospital in northern Italy, who had experience in the extra-hospital emergency. Non-participation is not discussed. | Data collection was at an italian emergency service organization called 118. The justification given by the researchers for choosing this location was to choose an environment familiar to the participants so that they would feel at ease. Semi-structured interviews are carried out with participants. The authors explain how they chose their questions, by taking inspiration from similar literature, but choice of | Researchers do not critically examine their role, potential bias and influence of research question formulation or data collection. | Ethical approval was gained from the Bioethical Committee of the University of Bologna. Informed consent was gained from participants before participation. Efforts to protect confidentiality are not discussed. | 'The interviews were conducted and analyzed by all the researchers after having been faithfully transcribed on digital text documents, reporting in brackets some relevant non-verbal gestures, and after having evaluated the nodes and relationships generated by the nVivo qualitative research software12.' Although sufficient data are presented to support the findings, it is unclear how the | Findings are presented as themes. Authors discuss arguments for and against certain issues raised. One example of this is the inclusion of quotations of participants who prefer talking to a peer supporter, and those who prefer talking to a professional therapist. No mention is made of efforts to discuss the credibility of findings. Contradictory data are taken into account and discussed. | The authors relate their findings to their aims. They propose that a peer support program should be introduced in the ambulance service. No recommendations for further research are made. |

For peer review only

| | | | | | | | | | |
|---|---|---|---|---|---|---|---|---|---|
| | | | | interviews over other qualitative methods is not explained. 'The interviews were audio-recorded and conducted anonymously'. Saturation of data is not discussed. | | | themes were generated from the data. | | |
| *Experiences among firefighters and police officers of responding to out-of hospital cardiac arrest in a dual dispatch programme in Sweden: an interview study* Hasselqvist-Ax et al. 2019[63] | Aims of study are clearly stated, and their importance justified. Qualitative methodology is appropriate due to nature of the aims, which is to explore subjective experiences in detail. | The authors don't justify their use of semi-structured interviews over other qualitative methods. | 10 police officers, 12 firefighters. Participants were purposively sampled for knowledge of two or more cardiac arrest situations, and to collect as rich descriptions as possible. Three recruitment approaches were used: '1) an invitation letter from the researchers was presented to the main collaboration group for OHCA alarms in Stockholm County; 2) on the police report for cardiac arrest alarms there was a request to contact the researchers for a voluntary interview; 3) fire stations were directly contacted for recruitment of participants.' Non-participation | The authors provide an interview guide with 7 open-ended questions, which form the basis for the semi-structured interviews. Setting for interviews: all but three took place at regular work places (not known where the others took place). Critical interview technique (CIT) was chosen as the method for data collection. The authors don't justify their use of this technique, but they justify their sample size based on the recommended sample size for CIT. Interviews were recorded and transcribed. The authors state no modification of methods during study were necessary. Saturation was | All interviews were conducted by the same author, to increase the chance that they were conducted in a similar way. The authors list the researchers' relevant strengths and past experiences, demonstrating reflexivity. | Ethical approval was obtained. Written informed consent was obtained from the participants and information was given about the possibility to withdraw from the study without any reprisal. Participants were not entitled to financial remuneration or other benefits. | 'This was an interview study where data were analysed by using critical incident technique (CIT) and inductive qualitative content analysis.' The authors explain the CIT process step by step, and provide an example with a piece of interview data. To enhance credibility, the researchers discussed the analysis at each step of the process, ensuring all analyses were supported by data. Occasionally the views of participants are summarised without examples of quotations. Contradictory data are taken into account, but saturation of data is not discussed. | The findings are presented as themes. Although participant quotations were provided, the authors didn't provide quotations for all of their comments. Often, the views of participants were summarised without providing participant quotes. | The authors provide recommendations for swedish emergency organizations, such as giving indications for training of paramedics and firefighters. |

| | | | | | | | | | |
|---|---|---|---|---|---|---|---|---|---|
| | | | was not discussed. | discussed in relation to the critical incident technique- where 20 interviews (of 2-4 CIs per interview) provide sufficient data. The authors included 22 participants. | | | | | |
| *Working in prehospital emergency contexts: Stress, coping and support from the perspective of ambulance personnel* Oliveira et al. 2019[52] | Aims of study are clearly stated, and their importance justified. Qualitative methodology is appropriate due to nature of the aims, which is to explore subjective experiences in detail. | The authors don't explain why semi-structured interviews were chosen over other qualitative methods. | 14 ambulance personnel. Participants were recruited from seven institutions, mainly on the basis of 'having an active status on a rescue team and being willing to talk and share their experiences'. Details of how participants were recruited are not provided, and non-participation was not discussed. | Interviews took place at the participants' local professional organisation, and were all conducted by the first author. Semi-structured interviews were conducted with participants following an interview guide, details of which are provided by the authors. The choice of semi-structured interviews was justified as it allowed the authors 'to obtain in-depth information regarding participants' prehospital emergency experiences'. Interviews were audio-recorded and transcribed. Saturation of data was achieved. | The researchers acknowledge the possibility of researcher bias during the coding process, and therefore they discuss their analyses with other researchers. | The research project was approved by the Portuguese Red Cross and by the University's Ethics Committee Written consent to participate in the study and to audio-record the interview was obtained, as follows: 'We sent a cover letter to all selected structures, explaining the aim of the study, the procedures, the ethical issues guaranteed, the voluntary character of the participation and the possibility to withdraw at any time'. | The authors use Braun and Clark's analytic approach, and outline the process in detail. This includes explaining how themes were derived from the data. Data collection was performed until saturation was reached. Data was discussed between the researchers during coding, enhancing credibility. The researchers made efforts to reduce researcher bias: 'Furthermore, to reduce research bias, there was a concern to engage with other researchers to discuss the process of data analysis and to illustrate themes with verbatim descriptions from participants.' Sufficient data are | Findings are presented as themes. The researchers provide participant quotations to support their findings. Multiple analysts discussed the analytic process to reduce researcher bias. | The researchers make recommendations to ambulance organisations to improve the psychological wellbeing of their staff. The authors also provide multiple avenues of recommended further research. |

For peer review only

| | | | | | | | | | |
|---|---|---|---|---|---|---|---|---|---|
| | | | | | | | presented to support the findings, and contradictory data are taken into account. | | |
| *Exploring paramedic communication and emotional expression in the workplace after responding to emergency calls* Drewitz-Chesney et al. 2019[53] | Aims of study are clearly stated, and their importance justified. Qualitative methodology is appropriate due to nature of the aims, which is to explore subjective experiences in detail. | The authors don't explain why semi-structured interviews were chosen over other qualitative methods. | 8 paramedics. The recruitment strategy is explained in detail. Participants were recruited over facebook groups. 'Participants were sampled using convenience, then purposive sampling. Convenience sampling enabled initial recruitment. Snowball and quota sampling were the two forms of purposive sampling used.' Authors are not clear on which attributes are sampled for. The authors justified their choice of participants by saying that 'when sufficient information is gleaned from participants, a smaller sample size is required ' | Semi-structured interviews were performed with participants, but the researchers do not justify why interviews are chosen. Interviews were conducted using Skype, and participants could choose to interview via video or audio only. An interview guide is provided. Interviews were audio recorded and transcribed, and data collection was stopped once saturation was reached. | The recruitment messages mention that the researcher previously worked as a paramedic. The researchers acknowledge the possibility of confusion and bias that can arise from this, although this was minimised by the fact that the researcher didn't work with any of the participants. | 'This study received ethical approval from the University of Edinburgh's Usher Research Ethics Group. Each participant provided verbal and written consent. To minimise the risk of psychological impact, participants were never asked to recall specific calls or details. Participants were monitored for signs of distress during each interview (none were noted). BCEHS paramedics have access to three services offering support and counselling. These services were listed on their information letters. At the conclusion of each interview, participants were asked if they wanted a referral to any of the services, which | The authors used constructivist grounded theory in their analytic process. The authors are transparent about how themes are generated from the data.' Credibility was enhanced through triangulation, including interviewing the manager of the BCEHS CIS program, Marsha McCall, and Anonymous, a retired BCEHS paramedic, whom substantiated some participant data . Thick descriptions and a diverse sample contributed to credibility and transferability. An audit trail was maintained throughout the research process which enhanced dependability. Journaling and bracketing enriched reflexivity ' 'Each transcript was read at | Quotations are interspersed among the results which are presented as themes. Credibility was enhanced through the use of triangulation with leading members of the organisation. | The researchers consider their findings in relation to existing literature. They also make various recommendations for emergency service organisations, relating to post-incident organisational interventions that could protect paramedic wellbeing. |

| | | | | | | | | | |
|---|---|---|---|---|---|---|---|---|---|
| | | | | | | all participants declined.' | least three times to improve accuracy and familiarity'. Sufficient data are presented to support the findings, and contradictory data are taken into account. | | |
| *Mental Health in the UK Police Force: a Qualitative Investigation into the Stigma with Mental Illness* Edwards et al. 2020[58] | Aims of study are clearly stated, and their importance justified. Qualitative methodology is appropriate due to nature of the aims, which is to explore subjective experiences in detail. | The authors justify their use of open-ended questions in semi-structured interviews to facilitate meaningful discussion, although the choice of interviews other qualitative data collection methods is not justified. | Five police officers. 'Participants were recruited through a referral from a charity, personal network, police forum and contact from within the police force.' The authors outline the criteria for participant inclusion in the study. They do not however justify their choice for these criteria. Non-participation was not discussed. | Semi-structured individual interviews were conducted using a topic guide, of which the authors provide details. The authors justify their use of open-ended questions. Interviews were audio recorded and transcribed. Data saturation was not discussed. | Researchers do not critically examine their role, potential bias and influence of research question formulation or data collection. | Ethical board approval was obtained, as well as written consent from the participants. The researchers also had measures in place to support participants who experience distress due to participation in the study. | The authors used Braun and Clark's approach of thematic analysis. They outlined this process in detail, giving examples of how they arrived at themes from the data. It is unclear whether multiple researchers collaborated to compare coding. Participant quotations are consistently embedded within the authors' interpretations, and contradictory data are taken into account. The authors are transparent about how themes were generated. | Findings are presented as themes and sub-themes. The authors include contradictory data, and discuss this. Discussion takes place throughout the study within the results section, but there is also a separate discussion section. | The authors identify areas where further research would be valuable, such as barriers to help-seeking in male dominated professions. Recommendations are also made to police organisations, such as highlighting a need to increase mental health awareness. |
| *Living in Critical Times: The Impact of Critical Incidents on Frontline Ambulance Personnel: A Qualitative Perspective* | Aims of study are clearly stated, and their importance justified. Qualitative methodology is appropriate due to nature of the aims, which is to | The authors don't justify why they chose individual interviews over other qualitative methods. They do however justify their use of some closed questions ( | 21 EMTs, 6 EMCs. The qualitative component of this study followed a quantitative Survey. Participants from the earlier study | Setting for data collection is not stated. The authors give examples of the topics discussed during the interview process. The interview | Researchers do not critically examine their role, potential bias and influence of research question formulation or data collection. | No mention of informed consent, ethical approval or methods to ensure confidentiality. | Thematic analysis was undertaken. 'A random sample of transcripts was read and coded by both authors in order in ensure good reliability and | Findings are presented as themes. For each theme, a range of relevant quotations are provided to support this theme. Credibility | The authors make recommendations to police organisations for post critical incident support for staff. |

| | | | | | | | | | |
|---|---|---|---|---|---|---|---|---|---|
| Gallagher et al. 2007[54] | explore subjective experiences in detail. | to elicit background information and to facilitate comparisons across participants. ) | were asked if they would be willing to participate in qualitative interviews. Non-participation is not discussed. | schedule used was created with the help of a literature review and the findings from the quantitative component of the study. No justification for the choice interview method is given. The interviews were audio recorded and transcribed. | | | validity.' It is not clear how the themes were derived from the data. The authors don't critically evaluate their own role in the analytic process. Contradictory data are taken into account- for example when giving examples of participant quotations with conflicting attitudes. The researchers don't critically examine their own role. Sufficient data are presented to support the findings. | of findings is not discussed. | |
| *Police officers, mental (ill-) health and spoiled identity* Bullock et al. 2018[59] | Aims of study are clearly stated, and their importance justified. Qualitative methodology is appropriate due to nature of the aims, which is to explore subjective experiences in detail. | Researchers justified their use of telephone interviews: '. While telephone interviews are often depicted as a less attractive alternative to face-to-face interviewing, telephones may allow respondents to feel relaxed, more able to disclose sensitive information and there is little evidence that they produce lower quality data ' | 52 police officers, two police staff, four Police Community Support Officers (PCSOs) (four) and one special constable. Participants were recruited from six police constabularies in England and Wales. 'Individual participants were identified by virtue of their contribution to an online survey on the nature of work-related injury in which | The researchers explain in detail how the telephone interviews were conducted. Open questions were asked by the participants. Saturation of data is not discussed. 'All interviews were digitally recorded, professionally transcribed and anonymized. ' | The researchers acknowledge that the nature of the interview is sensitive, and that the nature of telephone interviews would make participants more comfortable disclosing such information. | ' The nature of the interviewing was inevitably sensitive and mechanisms were put in place to mitigate that; for example, interviewees were provided with the contact details of sources of support.' There is no mention of ethical board approval, informed or methods to protect patient confidentiality. | The authors use Braun and Clark's thematic analysis approach, and give a very brief overview of the approach, but they do not give examples of how themes were derived from the data. The researchers didn't critically examine their own role. Sufficient data are presented to support the findings, and contradictory data are taken into account. Data | The findings are presented as themes. For each theme, the authors use participant quotations as well as relevant literature to discuss the context surrounding the quotations. No efforts to enhance credibility are discussed. | The authors make recommendations to police organisations based on their findings surrounding stigma. No recommendations for research are made. |

For peer review only

| | | | | | | | | | |
|---|---|---|---|---|---|---|---|---|---|
| | | | they identified themselves as willing to participate in a full-length interview'. This study is part of a wider project. Non-participation was not discussed. | | | | saturation is not discussed. | | |
| *'You see a baby die and you're not fine:' a case study of stress and coping strategies in volunteer emergency medical technicians* Folwell et al. 2018[55] | Aims of study are clearly stated, and their importance justified. Qualitative methodology is appropriate due to nature of the aims, which is to explore subjective experiences in detail. | The authors justify their choice of in-depth interviewing technique as they 'hope to gain a deeper understanding of the lived experiences of voluntary EMTs'. | 25 EMTs. Participants were recruited from one county in a Western state. It is not clear how the participants were selected, and non-participation is not discussed. | Interviews were conducted in a private space of the participant's fire department. Justification for choice of interview setting was not provided. In-depth interviews were performed with participants, using an interview guide. This was chosen to "gain a deeper understanding of the lived experiences of voluntary EMTs". Saturation was reached by the end of the study. Interviews were audio recorded and transcribed. | 'The research team consisted of a female Caucasian professor who holds a doctorate and a male Caucasian undergraduate student with three years' experience as a volunteer EMT. While some of the interviews were conducted by both members of the research team, most interviews were conducted by the volunteer EMT'. Other than providing these details, Researchers do not critically examine their role, potential bias and influence of research question formulation or data collection. | Confidentiality was guaranteed by the authors as the transcripts were anonymised. There is no mention of ethical board approval or informed consent. | The authors describe the process of constant comparison analysis. Two researchers independently read all the transcripts, and collaborated to discuss findings. The authors are very transparent about the analytic process. Contradictory data were discussed, such as in the example of participants discussing the pros and cons of CISD. Sufficient data are presented to support the findings. | Findings are presented as themes and sub-themes. 'To enhance credibility, member validation was performed in which a summary of findings and initial interpretations of data were given to five participants to confirm the researchers accurately depicted viewpoints and experiences. To enrich transferability, we provided detailed descriptions of participants and research sites. To improve dependability, we used the same protocol for each interview and documented the process of data collection and analysis. To | The authors make four detailed recommendations to the specific EMS organisation with which the study was involved. The authors also make recommendations for future research. |

| | | | | | | | | | |
|---|---|---|---|---|---|---|---|---|---|
| | | | | | | | | address confirmability, we used direct quotes from participants to support the finding' | |

For peer review only

# Appendix A- Abbreviations and definitions

| Abbreviation | Meaning |
|---|---|
| NHS | National Health Service |
| PTSD | Post-Traumatic Stress Disorder |
| ESW | Emergency Service Worker |
| ESO | Emergency Service Organisation |
| EMS | Emergency Medical Service |
| CISD | Critical Incident Stress Debriefing |
| UK | United Kingdom |
| TRiM | Trauma Risk Management |
| GMB Union | General, Municipal, Boilermakers and Allied Trade Union |
| RTA | Reciprocal Translocation Analysis |
| CASP | Critical Appraisal Skills Programme |
| EMT | Emergency Medical Technician |

| Term | Definition |
|---|---|
| Psychological distress | 'the unique discomforting, emotional state experienced by an individual in response to a specific stressor or demand that results in harm, either temporary or permanent, to the person'[41] |
| Critical incident | 'any event with sufficient impact to produce significant emotional reactions in people now or later'.[9] |
| Supervisor | EMS frontline personnel responsible for overseeing a 'crew' of EMTs/paramedics. *Brown University- Roles And Responsibilities Of Supervisory Staff. [internet] 2021[cited 2021 June 1]. Available from: https://www.brown.edu/campus-life/health/ems/roles-and-responsibilities-supervisory-staff#:~:text=EMS%20Supervisors&text=The%20EMS%20Supervisor%20is%20the,and%20appropriate%20emergency%20vehicle%20operation.* |
| Mental health champion | A member of an organisation raising mental health awareness within the workplace[99] |

| Type of stigma | Definition |
|---|---|
| Anticipated stigma (Perceived stigma) | *'the extent to which people believe they personally will be viewed or treated in a stigmatizing way if their mental health problem or related help-seeking becomes known'* [77] |
| Treatment stigma | 'the stigma associated with seeking or receiving treatment for mental ill health'[76] |
| Internalized stigma | 'holding stigmatizing views about oneself'[76] |
| Public stigma | 'invalidating and unjustified beliefs (i.e., prejudices and endorsed stereotypes) about others'[83] |


For peer review only - http://bmjopen.bmj.com/site/about/guidelines.xhtml

## Appendix B: Literature search terms

(((mental AND (health OR well?being)) OR (trauma* OR *stress OR recover* OR PTSD OR post?traumatic?stress OR emotion* OR (critical AND incident) OR (traumatic AND incident) OR (traumatic AND event) OR experience* OR support* OR *support) AND (emergency service* OR emergency medical service* OR EMS OR first responder* OR ambulanc* OR paramedic* OR firefighter* OR fire service* fire department* OR police*) AND (qual* OR mixed?method* OR interview* OR focus?group*) NOT (quality OR emergency?department)

((emergency service* OR emergency medical service* OR EMS OR first responder* OR ambulanc* OR paramedic* OR firefighter* OR fire service* fire department* OR police*) AND (qual* OR mixed?method* OR interview* OR focus?group*) NOT (quality OR emergency?department)) AND ((help-seeking* OR stigma* OR mental* OR barriers OR (MeSH terms: help-seeking behaviour, social stigma, mental health, psychiatry, social support, mental disorders))

For peer review only



For peer review only - http://bmjopen.bmj.com/site/about/guidelines.xhtml

# Appendix C: Data extraction template

Citation
Reviewer
Country
Aims
Ethics – how ethical issues were addressed
Study setting- e.g. type of organisation

Relevant context to study setting

Socio-demographics of the country / region

Recruitment context (e.g. where people were recruited from)

Sampling- what sampling methods, what were inclusion and exclusion criteria,

Data quality rating

Participants- 'population described'

Participants- 'characteristics'- age, sex etc

Theoretical background

Proportion of sample exposed to critical incidents/ definition of critical incident/ anything relevant to the study matching my selection criteria

Definition of critical incidents/ something related

Data collection methods- data collection methods, role of researcher within setting...

For peer review only

Data analysis approach- how many researchers, how did they code, how were findings corroborated,

Themes identified in study (1st order interpretations)- Help-seeking

Themes identified in study (1st order interpretations) – Mental Health recovery

Data extracts related to key themes- Help-seeking

Data extracts related to key themes- Mental health recovery

Author explanation/interpretation of key themes (2nd order interpretations)- Help-seeking

Author explanation/interpretation of key themes (2nd order interpretations)- Mental health recovery

Recommendations made by authors (both outcomes; but specify)

Third order interpretations:

Other potentially relevant information

For peer review only

# Appendix D: Example literature quotations used to construct themes

| | | | |
|---|---|---|---|
| **Factors contributing to mental health recovery post traumatic incident exposure** | **Organisational** | **Time out/Downtime** | *They prefer support to be offered immediately after the call and find that downtime after a stressful call allows them to decompress and prepare for the rest of their shift: '…we knew we weren't going to get a call right, so we knew we had the two hours, so we watched a funny show and had a nap…But…like for me personally… that's exactly…what I needed.' Participant 4 from focus group 4.* Douglas et al. 2013<br><br>*. . . our supervisor took us out of service for a couple of hours and let us go have lunch, sat down and had lunch and just kind of relax and talk amongst ourselves, not even about the call, just about whatever, just to kind of relax. Before we went back on shift. Certainly we could have booked off the rest of the day, you know, on stress leave or whatever, but we all, found that just having, just being able to have a couple of hours to, kind of, you know, relax a little bit, that helps us a lot.' (Focus group #520–522)*<br>Halpern et al. 2008<br><br>*I like didn't want to be like I need to take a couple of hours off because I did not want to look bad in front of the supervisor.-Participant 2 from focus group 2.*<br>Douglas et al. 2013<br><br>*Unfortunately, paramedics often do not have adequate time to talk. Several participants expressed their frustration over 'calls waiting'. These calls are waiting to be dispatched as no crew is available. When BCEHS paramedics offload their patient(s) at the hospital, dispatch can immediately send them to a waiting call. This can limit paramedics' ability to discuss CIs. James desired:*<br><br>*…to be given the time uninterrupted, unbothered… Give us our time to decompress.*<br>Drewitz-Chesney et al. 2019 |
| | | **Supervisor** | *Depending on who it is . . . one supervisor, we took, we did a [critical call], and we took the last hour of the shift off. And he was making us fill out all these forms and, you know, telling us that if we wanted to take the next shift off that we had to go see our doctor and get a note for this and that. And you know, just made it more stress-(Focus group #520–522)*<br>Halpern et al. 2008<br>*'And so our supervisor was really, we have a great supervisor and he's, you know, asked us all specifically, "are you guys okay", you know. And the other crew went off on stress for the rest of the shift. We stayed, because we said, y ou know, we're okay. We just kind of dealt with the aftermath of everything. It was still a pretty stressful call but at least we had that option. And he had no problems, like, he said, go home. Whatever you guys need. So and that's a big thing.- (Focus group #520– 522)*<br>Halpern et al. 2008 |


For peer review only - http://bmjopen.bmj.com/site/about/guidelines.xhtml

| | | | |
|---|---|---|---|
| | | | *Supervisors, however, were perceived differently. Some paramedics reported feeling unsupported when their supervisors questioned why they were reacting to an event that they did not perceive as traumatic:*<br>*I wasn't really involved in a traumatic event because there was no loss of life, or you know what I mean, um, it, there wasn't really much sympathy the, um, I could, not that I really, um, oh, this wasn't, um, a huge accident, you weren't injured, you know, really why are you complaining?*<br>Regehr et al. 2007<br><br>*he's [supervisor] quite an old fashioned sort of police officer, not the bloke you would sort of want to go in and have a chat with about a sudden death you'd just been to … If I went in and said 'Governor, can I have a chat about the sudden death?', he'd look at me as if I'd just asked to kill one of his children!*<br>Evans et al. 2013 |
| | | **Peer support network** | *Nurse 1 "Therefore, talking to a person who has the same skills, abilities and knowledge you have would probably be more meaningful".*<br>Carvello et al. 2019<br><br>*The majority of nurses is in favour of peer-supporters. The motivation is based on the fact that they recognise the peer-support as someone that can understand what nurses really mean when relating a traumatic event, being one of their colleagues.* Carvello et al. 2019 |
| | **Informal support** | **Colleagues and family** | *The more you talk about something, the more it becomes something you've told and your telling becomes part of the memory, as opposed to it being a really shiny, vivid thing inside your head*those images.*<br>Evans et al. 2013<br><br>*I don't think talking about it to people at work is the release, the escape I need … it's speaking to people who I care about and who care for me and just having that comfort zone, that's what's important to me. [P16]*<br>Evans et al. 2013<br><br>*… I never tell my wife that, I would never tell her that because I just think that would have really put the frighteners on her. [P15]*<br>Evans et al. 2013 |
| | | **Regular partner** | *For me it's just always been that partner, because they're right there with you and they'll know what's going on, and you really want somebody that can understand what's happening.*<br>*Jessica et al. 2009*<br><br>*…for those of us that have regular partners, regular stations, rely on your partner… They're gonna know if something's up with you.*<br>Drewitz-Chesney et al. 2019<br><br>*Paramedics working with different partners may be unable to recognise changes as readily as they would in a regular partner. When paramedics are without a regular partner or are uncomfortable speaking with their* |



For peer review only - http://bmjopen.bmj.com/site/about/guidelines.xhtml

| | | | |
|---|---|---|---|
| | | | *current partner, they often don't discuss calls. Instead, some participants said they speak with long-time paramedic friends or coworkers with whom they have similarities, ie. age or training level.*<br>Drewitz-Chesney et al. 2019<br><br>*…if I'm working with my regular partner, those communication lines are very, very open and we can talk about the tough calls, how we're feeling, if there's anything we would have done different [sic] (Sean).*<br>Drewitz-Chesney et al. 2019<br><br>*I really envy the paramedics that have regular partners that they know, and they trust, and they can talk to (Krista).*<br>Drewitz-Chesney et al. 2019<br><br>*…I would have a regular partner and that partner would be someone who is just like family to me. We would just talk about everything, without even hesitating…'*<br>Drewitz-Chesney et al. 2019 |
| | | **Reassurance and validation** | *I remember going to the hospital and I remember I was disappointed because the parents weren't there at that time...And I don't know what it was. It was just something that made me feel like I just needed to talk to them. But I never ended up talking to them. So anyway that call definitely stands out as being...(Stuck with you.) Yeah.*<br>Halpern et al. 2009<br><br>*And it's weird, I looked in the newspaper. I saw his funeral announcement and I went to the funeral. (Oh okay.) I didn't talk to any of the family. I just went for my own, I don't know why …I, for some reason, I guess I felt I needed to follow up and so I did. And I sent the family a condolence card and then a friend of mine …met a friend of the family's friend and he had said, oh, his [relative] wants to talk to you …So she called me and she wanted to know, like, you know, what happened with her [relative] and who was the last one to talk to him …It's kind of funny because there are so many calls you do and you never think about them again. But this one, I thought about him a lot. I guess because he was so young and again, I really didn't think he was going to die. So I thought, okay, well, you know, he's at the trauma centre now and I had no idea that he was that badly injured. And then he died. So I think that freaked me out. I wasn't prepared for that. And I thought, oh my God, he's so young and his family wasn't there. I felt badly his family wasn't there and then. So that one stuck with me for a while.*<br><br>*Interviewer: "And talking to his [relative], did that help or …?"*<br><br>*It more, I felt it was helping them so it made me feel better, because she really, really was upset. And I felt she wanted, I think she felt a lot better after talking to me and hearing something about what happened. And so it made me feel better to give them some kind of closure.Pg 181-182*<br>Halpern et al. 2009 |



For peer review only - http://bmjopen.bmj.com/site/about/guidelines.xhtml

| | | | |
|---|---|---|---|
| | | | *. . . the day after that call, my supervisor kind of sensed that I wanted to talk to him and I don't know why, you know, he came to the hospital. But it was the next day and he was kind of like, so pull up a chair, sit down. Let's just talk. And it was like I wanted somebody just to say to me, okay, this is your time and you can talk right now and I'm not going to judge you. I'm not going to talk about anything. I'm going to let you just have your 10 minutes. And that made a huge difference. I have the utmost respect for my particular supervisor . . . Whether it was talking specifi cally about the call or just having a little bit of a "hey you're okay at your job", type comment. (Participant #122)*<br>Halpern et al. 2008 |
| **Factors influencing help-seeking behaviour** | **Nature of intervention delivery** | **Mandatory vs non-mandatory** | *We had counselling every six months … and everybody used to go 'Oh I've got to see the counsellor this week', but I tell you what … we all quite enjoyed it … I was so much calmer after speaking to her but it's something I'd never have done had I not been made to do it. [P12]*<br>Evans et al. 2013<br><br>*My emotions are none of your business and if I wanted to share my emotions with you, I'm going to share [them] with someone I trust… Participant 1 from focus group 1.*<br>Douglas et al. 2013 |
| | | **Shared experiences with intervention provider** | *FRs in our study preferred a MH professional with experience as an FR or military veteran. Many also approved of a provider that "knew the job," either working with multiple FRs in the past, or even as a family member.*<br>Jones et al. 2020<br><br>*I've been to [therapy] a couple of times. . . . The guy that I got was excellent, but I only think it was excellent because he was prior military (P8, Firefighter ×22 years).*<br>Jones et al. 2020 (study 4)<br>*…someone I think understands what's going on, and has been through what I've done.- Participant 3 from focus group 4.*<br>Douglas et al. 2013 |



For peer review only - http://bmjopen.bmj.com/site/about/guidelines.xhtml

| | | | |
|---|---|---|---|
| | **Stigma as a help-seekingbarrier Specific barriers** | **'Macho culture'** | *I think there's a real element of machismo and masculinity in the police force and it's a bit, sort of a faux pas to admit that things have really affected you ... If I'd have come out and said 'ah you know, that really affected me badly, let's go and sit down and have a cup of tea and talk about it' I think you're straying into pink and fluffy territory there ... saying 'that made me feel sad' is a bit too far. [P3]*<br>Evans et al. 2013<br><br>*Everyone wants to be tough and strong. Maybe that was my downfall or problem at the time and I didn't want to admit that I needed any kind of help. I guess I didn't want to be perceived as weak. (Participant #110)*<br>Halpern et al. 2008<br><br>*… that's so ridiculous. You need to harden up if you want to do this job, and old people die.*<br>Drewitz-Chesney et al. 2019<br><br>*… in metro, there's a lot more...bravado and joking around about things…If you can get one on one with someone, they're usually a lot more receptive and a lot more empathetic- (Dennis).*<br>Drewitz-Chesney et al. 2019<br><br>*Unfortunately there's a massive stigma […] one of my sergeants the other week, there was mental health training coming up and his reaction to being put forward to go on the training was, 'Well what do I want to go and learn how to deal with a load of nutcases for?*<br>Bullock et al. 2018<br><br>*We are many women at the station so its easier for us to talk about what happened…You have to talk about what happened otherwise you can't go on. It's easier than on a station with only men, it's not necessary with any "macho style" so I think it's easier for the men (at our station) to talk about things you must talk about. (A female nurse describes her feelings.)*<br>Jonsson et al. 2003 |
| | | **Stigma and shame** | As an institution, they were very very good, in getting me better, at making me understand, but back at work that was a different ball game. You've got your colleagues who are still at work, still running around like idiots and they're like, oh fucking hell, you've had three months off, you know, I should have gone off with stress. (Participant 1).<br>Edwards et al. 2020<br><br>*There have been officers that are doing the shift that have shown that they can't deal with situations like that, and been very open about it*and they haven't got the respect from the shift, because the colleagues go 'well, you're on your own if you're working with her, because she'd back away' or whatever. So you don't want to be considered as one of those. [P7]*<br>Evans et al. 2013 |
| | | **Career concerns** | *I think that the stigma is you have to be very careful who you tell that it bothered you or you might get judged as weak or you might get fired" (P2, EMT/paramedic ×20 years).*<br>Jones et al. 2020<br><br>If you've a form of mental health illness you will not get on; you will not be promoted, …people will not want you on their section. (Participant 4).<br>Edwards et al. 2020 |


For peer review only - http://bmjopen.bmj.com/site/about/guidelines.xhtml

| | | | |
|---|---|---|---|
| | | | I feel scared to declare anything or do anything about anything because will it bite me later on in life? Will it prevent me from doing something in the police later on? Could it be used against me? … will it be used in a negative way later on?<br>Bullock et al. 2018 |
| | | **Confidentiality concerns** | *I know our department's very, very poor at keeping secrets. So if I put a crew out of service, I have to tell the communications center. Communications says, we're sitting this far apart, Hey X, I just put the 22 car out of service in stress. Every dispatcher in there hears it. Every call receiver in there hears it. So they hear it. I don't know if they say anything. I don't know if they go home and tell all their friends and family. But I don't like that system. There's no quiet way of doing it. (Participant #128)*<br>Halpern et al. 2008<br><br>*I think that the stigma is you have to be very careful who you tell that it bothered you or you might get judged as weak or you might get fired" (P2, EMT/paramedic ×20 years).*<br>Jones et al. 2020<br><br>*.. .to whom must I speak in the police if I can't trust anyone. So now I'm seeking professional help outside the police .*<br>Boshoff et al. 2015 |
| | **Mental health literacy** | **Emotional awareness** | *Some appreciated that difficulties in recognizing and admitting to distress pose significant barriers to accessing support. Recognizing the emotional impact of critical incidents may help to address these barriers.*<br>Halpern et al. 2009<br><br>*I didn't recognise it as what it was; I just thought I was grumpy…you don't see them creeping up, and in the end, the thing that tips you over the edge, the thing that makes your bottle overflow if you like can be something quite small because you've got used to dealing with stuff. (Participant 5).*<br>Edwards et al. 2020 |
| | | **Education and stigma** | *I think for, for everybody, is, here's my thought towards your process, is giving them the tools. People are going to be very tough and say, yeah, yeah, fine. But you know if you could somehow identify the emotions that go along with these calls t hat might be starting to put you on tilt…Then you can teach people to be aware of them and say, hey, you know what, it's okay to say, I need to talk to someone.*<br>Halpern et al. 2009<br><br>*In particular, it would appear from this study that teaching ambulance personnel about the emotional aspects surrounding different types of critical incident may diminish their confusion about which incidents they can expect to impact them.*<br>Halpern et al. 2009<br><br>*I guess that's the main two [barriers], pride and then denial. . . . But, it's just education. Just letting [FRs] know, look, these things are normal. It's going to happen to somebody" (P11, Firefighter ×8.5 years).*<br>Jones et al. 2020 |


For peer review only - http://bmjopen.bmj.com/site/about/guidelines.xhtml

| | | | |
|---|---|---|---|
| | | | *Knowing that other people are there dealing with that same stuff. You can bounce ideas off each other, see what's worked in their situations and what hasn't . . . you realize you're not crazy (P12, EMT/paramedic ×14 years).* Jones et al. 2020<br><br>*If you could actually get people in in front of officers saying, 'I was one of those people that didn't believe stress could ever get to that level and it was ridiculous and you just needed to work harder', maybe officers would accept that from another officer more than just somebody standing in front of you training because it's getting officers to accept that actually it's okay, you're only human and your body and your mind can only take so much, and maybe they'd accept it more then.* Bullock et al. 2018 |

For peer review only

For peer review only



For peer review only - http://bmjopen.bmj.com/site/about/guidelines.xhtml
